# Copper-catalyzed trichloromethylative carbonylation of ethylene†

**DOI:** 10.1039/d3sc05530b

**Published:** 2023-12-14

**Authors:** Youcan Zhang, Bing-Hong Teng, Xiao-Feng Wu

**Affiliations:** a School of Chemistry and Chemical Engineering, Liaoning Normal University 850 Huanghe Road Dalian 116029 China; b College of Chemistry and Chemical Engineering, Shanghai University of Engineering Science Shanghai 201620 China; c Dalian National Laboratory for Clean Energy, Dalian Institute of Chemical Physics, Chinese Academy of Sciences 116023 Dalian Liaoning China xwu2020@dicp.ac.cn; d Leibniz-Institut Für Katalyse e.V. Albert-Einstein-Straβe 29a 18059 Rostock Germany

## Abstract

Difunctionalization of alkenes is an efficient strategy for the synthesis of complex compounds from readily available starting materials. Herein, we developed a copper-catalyzed visible-light-mediated trichloromethylative carbonylation of ethylene by employing commercially available CCl_4_ and CO as trichloromethyl and carbonyl sources, respectively. With this protocol, various nucleophiles including amines, phenols, and alcohols can be rapidly transformed into β-trichloromethyl carboxylic acid derivatives with good functional-group tolerance. Bis-vinylated γ-trichloromethyl amides can also be obtained by adjusting the pressure of carbon monoxide and ethylene. In addition, this photocatalytic system can be successfully applied in the late-stage functionalization of bioactive molecules and pharmaceutical derivatives as well.

## Abstract 2

A copper-catalyzed trichloromethylative carbonylation of ethylene under visible light irradiation by employing inexpensive and readily available CCl_4_ and CO as C1 sources for trichloromethyl and carbonyl has been developed.

## Graphical abstract

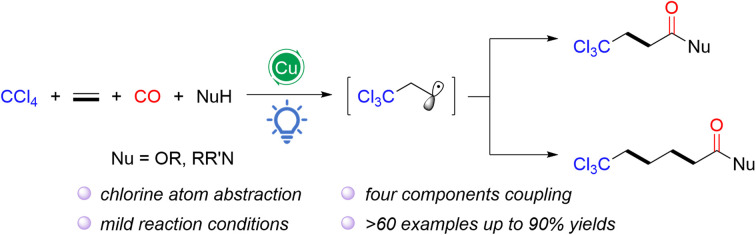

Polychloroalkyl motifs are well known as key components to alter the biological activity of organic compounds, which are widely distributed in natural products, pharmaceuticals, and bioactive molecules.^1–3^ During the last few decades, thousands of natural products containing C–Cl bonds have been discovered, especially those embedding the trichloromethyl skeleton, such as desenamide A,^4,5^ desenpyridine,^6,7^ sintokamide A,^8,9^ and callyspongiamide B,^10^ which have shown excellent biological activities in antibiotics and antitumor applications (Fig. 1a). Additionally, the trichloromethyl motif can also be employed as a versatile building block for organic transformations.^11–13^ Therefore, the incorporation of the trichloromethyl group into organic compounds has attracted considerable attention from chemists. Conventionally, trichloromethylation strategies employ base-promoted deprotonation of CHCl_3_ (ref. ^14–16^) or deprotection of TMS-CCl_3_ (ref. ^17–19^) to form trichloromethyl anions, which are then applied as coupling partners. Meanwhile, the Kharasch reaction considered as an alternative method of trichloromethylation proceeds through the addition of trichloromethyl radicals to alkenes *via* C–H cleavage of CHCl_3_ or C–X bond activation of CXCl_3_ (X = Cl, Br)^20,21^ (Fig. 1b). In recent years, the catalytic functionalization of C–Cl or C–H bonds of polychloromethanes (CCl_4_ and CHCl_3_) with alkenes and other coupling partners has emerged as a powerful trichloromethylation protocol to construct trichloromethyl functionalized compounds in a single step (Fig. 1b).^22–25^ However, these methods often reacted at high temperatures and require a stoichiometric amount of oxidant. Accordingly, redesign of redox-neutral reactions for preparing functionalized trichloromethyl-containing compounds under mild conditions remains urgently needed.

**Fig. 1:**
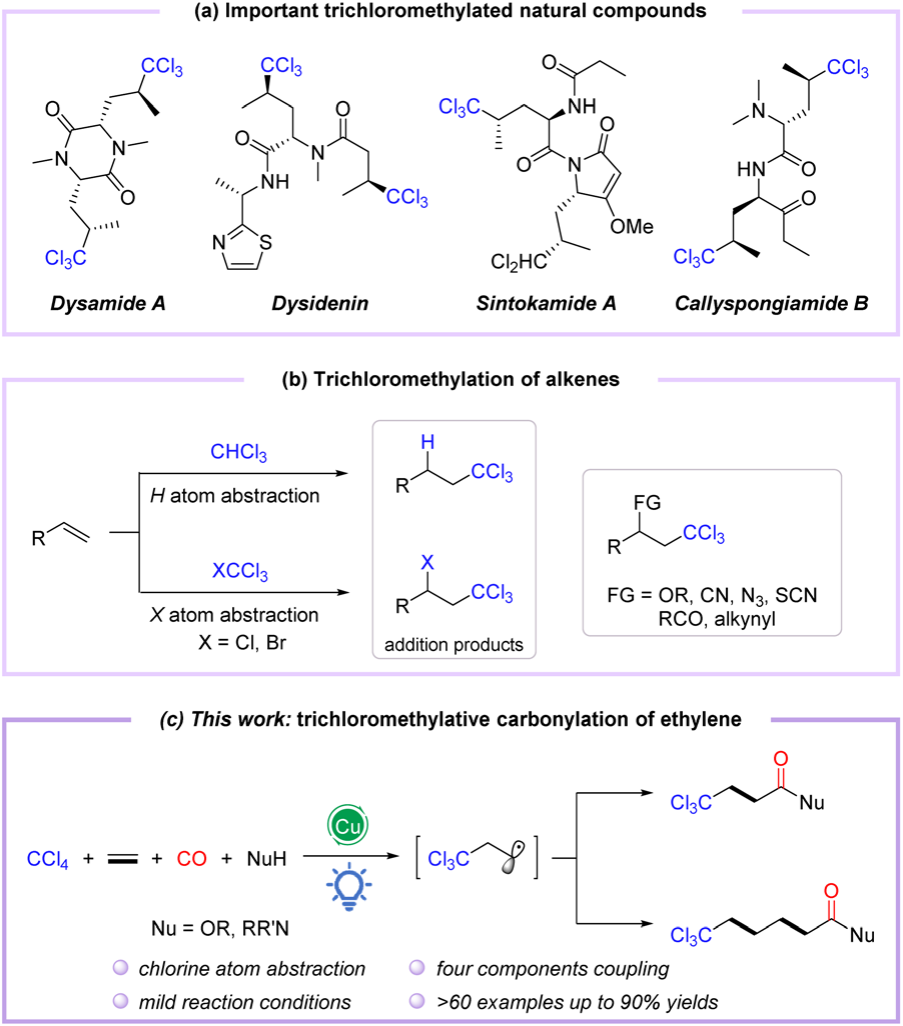
(a) Important trichloromethylated natural compounds. (b) Trichloromethylation of alkenes. (c) Copper-catalyzed visible-light-induced trichloromethylation/carbonylation of alkenes.

The radical relay-type vicinal difunctionalization of alkenes is one of the most attractive strategies to rapidly incorporate diversely functionalized molecular backbones.^26–29^ However, examples of trichloromethylative difunctionalization of alkenes employing CCl_4_ as a versatile trichloromethyl radical precursor under external oxidant-free conditions are still rarely reported.^30,31^ On the other hand, amides and esters are powerful molecular backbones ubiquitous in compounds that display biological properties,^32–35^ especially in complex molecules with a trichloromethyl group. In this context, divergent syntheses from simple and readily available substrates to obtain diverse trichloromethyl-containing carbonylated compounds have received much attention. To date, only one report on palladium-catalyzed trichloromethylative carbonylation of alkenes in ethanol to synthesize β-trichloromethyl esters in 12–60% yields has been reported by Tsuji and coworkers in 1985.^36^

Catalytic carbonylation represents a direct and efficient tool; in particular, difunctionalized carbonylation based on alkenes allows the simultaneous introduction of two building units, including the carbonyl group, enabling the synthesis of functionalized carbonylated compounds.^37–40^ Among them, visible light-induced radical carbonylation has received much attention because it can avoid certain disadvantages of conventional thermal based methods and promote the formation of radicals under relatively mild conditions.^41–44^ The trichloromethyl group has been well installed in alkenes as a trichloromethyl radical equivalent, generated by visible-light-induced Cl-atom abstraction of CCl_4_.^45–47^ Nevertheless, trichloromethylative difunctionalization is still challenging, primarily due to the weak C–X bond of CCl_4_, which induces side reactions such as addition, coupling, and elimination to compete with the target reaction. In view of our continuing research interest in transition metal-catalyzed and photo-induced difunctionalization of alkenes by the radical pathway,^48–50^ we envisioned the trichloromethylative carbonylation of alkenes to construct a series of trichloromethyl-modified carboxylic acid derivatives under mild photocatalytic conditions in which CCl_4_ was selected as the trichloromethyl radical precursor (Fig. 1c). In this strategy, the insertion of CO (carbon monoxide) and the oxidation of acyl radicals are the key steps during the photocatalytic process.

To explore this multicomponent reaction, screening studies were initiated in ethylene and CO atmospheres with CCl_4_ and aniline as the trichloromethyl precursor and nucleophile, respectively (Table 1). After systematic screening of reaction conditions, the desired β-trichloromethyl amide 3a could be smoothly provided in 77% isolated yield when the reaction was performed in the presence of a photoredox catalyst *in situ* generated from Cu(OTf)_2_ and bpy (bipyridine), using K_2_CO_3_ as the base in MeCN upon irradiation with 456 nm blue LEDs at room temperature (25–30 °C) for 24 h under ethylene (1 bar) and CO (50 bar) atmospheres (Table 1, entry 1). Other N-ligands were subsequently examined, such as 1,10-phen (1,10-phenanthroline) and 4,4′-diOMe-2,2′-bpy (4,4′-dimethoxy-2,2′-bipyridine) delivered similar yields, and lower conversion was found when 6,6′-diMe-2,2′-bpy (6,6′-dimethyl-2,2′-bipyridine) was selected as the ligand (Table 1, entries 2–4). Undesirable, none of the other copper precursors could improve the yield of the targeted product (Table 1, entries 5–6). Next, different bases were also investigated; among them with the inorganic bases Na_2_CO_3_ and K_3_PO_4_, slightly reduced yields were observed (Table 1, entries 7–8), whereas the organic base triethylamine resulted in a drastic decrease in yield (Table 1, entry 9). In addition, reaction solvent screening revealed that MeCN was the best solvent among the evaluated solvents (Table 1, entries 10–12). Using well prepared copper complexes with different ligands instead of Cu(TfO)_2_ and bpy were tested as well, and the desired carbonylation product can be obtained in all the cases (Table S2,† entries 7–10).^51–53^ Control experiments indicated that a copper salt, ligand, base, and blue light were all indispensable, although the desired product was still detectable in the absence of the ligand at elevated temperature (Table 1, entries 13–16, for more details, see the ESI†).

**Table 1:** Study of reaction conditions^a^

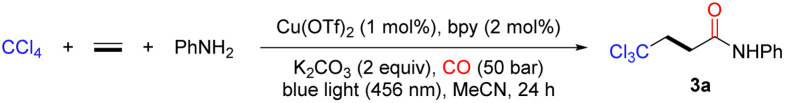		
Entry	Deviation from standard conditions	Yield (%)^a^
1	None	80 (77)^b^
2	1,10-Phen instead of bpy	78
3	4,4′-diOMe-2,2′-bpy instead of bpy	76
4	6,6′-diMe-2,2′-bpy instead of bpy	60
5	Cu(OAc)_2_ instead of Cu(OTf)_2_	75
6	CuI instead of Cu(OTf)_2_	76
7	Na_2_CO_3_ instead of K_2_CO_3_	79
8	K_3_PO_4_ instead of K_2_CO_3_	76
9	Et_3_N instead of K_2_CO_3_	22
10	PhCF_3_ instead of MeCN	53
11	1,4-Dioxane instead of MeCN	65
12	DCE instead of MeCN	57
13	No bpy	24
14	No Cu(OTf)_2_, no K_2_CO_3_, or no light	n.d.
15	No light, 40 °C	56
16	No light, 80 °C	76

a Reaction conditions: CCl_4_ (1.0 mmol), ethylene (1 bar), PhNH_2_ (0.2 mmol), Cu(OTf)_2_ (1 mol%), bpy (2 mol%), and K_2_CO_3_ (2.0 equiv.) in MeCN (1.0 mL) at 25–30 °C for 24 h under CO (50 bar). Yields were determined by GC-FID analysis using *n*-hexadecane as the internal standard.

b Isolated yield. n.d.: not detected.

With the optimal reaction conditions in hand, the general substrate scope of this copper-catalyzed photo-induced multicomponent trichloromethylation/carbonylation was systematically explored. As shown in Scheme 1, various nucleophiles including amines, phenols, and alcohols were successively investigated. Arylamines with different functional groups on the benzene ring, including electron-donating (isopropyl, methoxy, methylthio, phenoxy, and acetamido) and electron-withdrawing (trifluoromethyl, trifluoromethoxy, acetyl, and cyano), smoothly participate in carbonylation, delivering the corresponding products 3a–3m in 25–90% yields. Among them, the scope of arylamines was sensitive to steric and electronic changes of functional groups, and large steric hindrance and electron-donating functional groups significantly inhibit the reaction; in contrast, electron-withdrawing functional groups promote efficient conversion of substrates. Notably, aryl halides (3g and 3h) or aryl nitriles (3l) are well compatible with catalytic systems. Moderate yields were obtained when this protocol was extended to substrates with the pyridine motif (3n and 3o). Secondary arylamines such as *N*-methylaniline and indoline also proceeded smoothly, while 3q was obtained in lower yield, likely due to its steric hindrance. Benzylamine, even NH_3_ were converted successfully as well, afforded the desired β-trichloromethylamides 3r and 3s in 39% and 70% yields, respectively.

**Scheme 1:**
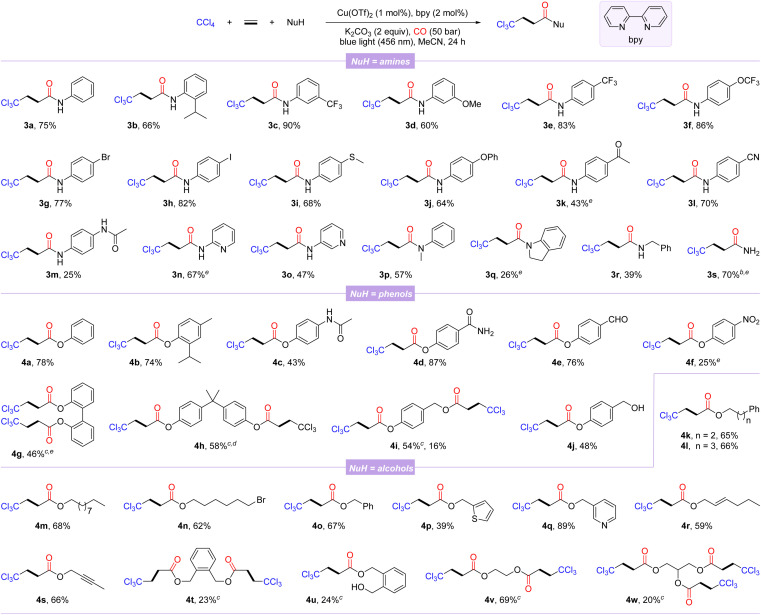
Scope of nucleophiles. [a] Reaction conditions: CCl_4_ (2.0 mmol, 5.0 equiv.), ethylene (1 bar), NuH (0.4 mmol, 1.0 equiv.), Cu(OTf)_2_ (1 mol%), bpy (2 mol%), and K_2_CO_3_ (0.8 mmol, 2.0 equiv.) in MeCN (2.0 mL) at 25–30 °C for 24 h under CO (50 bar); isolated yields. [b] NH_3_ (0.4 mmol, 0.4 M in dioxane). [c] NuH (0.2 mmol). [d] MeCN (3.0 mL). [e] 95% purity.

Next, several β-trichloromethyl esters 4a–4f were prepared from the corresponding phenols. The steric hindrance of phenols had an insignificant effect on the carbonylation procedure (4a-4b). Phenols with an acetamido, carbamoyl, aldehyde, or nitro group were tolerated well, albeit with relatively low yields of 4c and 4f. The reaction was also amenable to diphenols, furnishing the corresponding double carbonylation products 4g and 4h in 46% and 58% yields, respectively. In addition, 4-(hydroxymethyl)phenol with two hydroxyl groups could selectively lead to the double-carbonylation product 4i or simultaneously to the mono-carbonylated and double-carbonylated products 4i and 4j by controlling the reaction loading. Finally, we also examined a variety of alcohols as the nucleophilic partners, among which alcohols with distinct carbon chain lengths and remote bromine atoms could be successfully converted to the corresponding esterification products 4k–4n in 62–68% yields. Benzyl alcohols and heterocyclic units of thiophene and pyridine replacing phenyl behave well in this reaction (4o–4q). Allyl alcohol and alkynyl alcohol could generate the corresponding esters 4r and 4s in good yields with the C–C unsaturated bonds intact. Notably, alcohols with two reaction sites were suitable substrates, yielding the corresponding products 4t–4v, while the mono-carbonylation product 4u was also observed, probably due to the influence of steric hindrance. Even glycerol with trihydroxyl groups could participate in the reaction, albeit the tricarbonylated product 4w was obtained with a lower yield.

To further validate the practicality and generality of this procedure, the late-stage functionalization of several natural products, pharmaceutical derivatives, and bioactive molecules was performed. As shown in Scheme 2, aminoglutethimide, sulfalene, and anilines modified with menthol and camphorsulfonyl chloride were well matched to furnish the desired products 5a–5d in 39–85% yields, which indicated that the weakly nucleophilic sulfonamide is also a potential reactive site, whereas the amide functional group exhibits no reactivity. Additionally, our protocol could be extended to O-nucleophilic complex molecules with equal ease to advanced β-trichloromethyl esters derived from raspberry ketone (5e), estrogen (5f), fluorescein (5g), diacetonefructose (5h), DL-menthol (5i), epiandrosterone (5j), pregnenolone (5k), and estradiol benzoate (5l) in moderate to high yields (58–81%). As we expected, ezetimibe with two potential hydroxyl reactive sites also successfully accomplished double carbonylation to acquire the trichloromethylated carbonylation product 5m in 65% yield. Satisfactorily, scaling up the carbonylation involving aniline and phenol to 2.0 mmol, respectively, supplied the desired products 3a and 4a smoothly unhindered even with halving the catalytic amounts of copper salt and ligand. Unexpectedly, the scale-up reaction extended to phenylpropanol with a markedly lower yield of the target product 4l. It is also worth mentioning that as analogues of ethylene, but-3-en-1-ylbenzene and styrene were also tested, but only compounds based on a non-carbonylation reaction were detected.

**Scheme 2:**
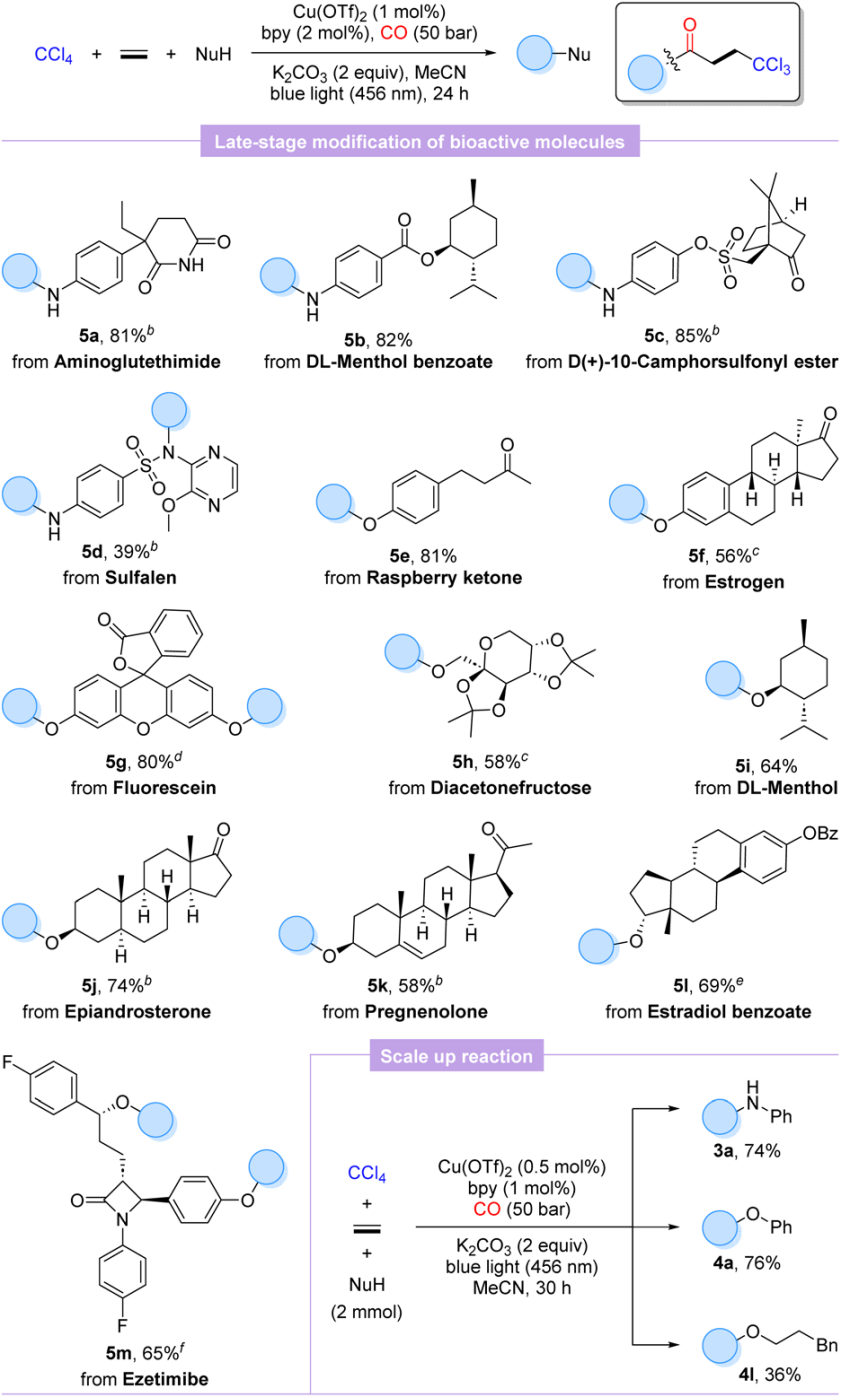
Late-stage modification of bioactive molecules and scale up reaction. [a]Reaction conditions: CCl_4_ (2.0 mmol, 5.0 equiv.), ethylene (1 bar), NuH (0.4 mmol, 1.0 equiv.), Cu(OTf)_2_ (1 mol%), bpy (2 mol%), and K_2_CO_3_ (0.8 mmol, 2.0 equiv.) in MeCN (2.0 mL) at 25–30 °C for 24 h under CO (50 bar); isolated yields. [b] CCl_4_ (1.0 mmol), NuH (0.2 mmol), and K_2_CO_3_ (0.4 mmol). [c] MeCN (3.0 mL). [d] NuH (0.2 mmol) and MeCN (3.0 mL). [e] CCl_4_ (0.5 mmol), NuH (0.1 mmol), and K_2_CO_3_ (0.2 mmol). [f] CCl_4_ (1.0 mmol), NuH (0.1 mmol), Cu(OTf)_2_ (2 mol%), bpy (4 mol%), and K_2_CO_3_ (0.4 mmol). Scale up reaction (2.0 mmol level): CCl_4_ (10.0 mmol, 5.0 equiv.), ethylene (1 bar), NuH (2.0 mmol, 1.0 equiv.), Cu(OTf)_2_ (0.5 mol%), bpy (1 mol%), and K_2_CO_3_ (4.0 mmol, 2.0 equiv.) in MeCN (10.0 mL) at 25–30 °C for 30 h under CO (50 bar); isolated yields.

Inspired by the polymerization reaction of CO with ethylene to produce polyketones,^54–56^ we are keenly interested in exploring the photo-induced trichloromethylative carbonylation of ethylene for the synthesis of carbon chain-extending remotely trichloromethyl-modified aliphatic carboxylic acid derivatives. We conjectured that radical carbonylation transformations involving bis- or even multi-ethylene relays could be achieved by increasing the amount of ethylene. To confirm our hypothesis, the model reaction was probed in terms of parameters such as ligand, pressure of CO and ethylene (for details, see the ESI†). After identifying the optimal conditions, we then evaluated the substrate scope of this transformation with the access of amines to γ-trichloromethyl amides (6a–6i). As shown in Scheme 3, anilines with diverse functional groups, including methoxy, *tert*-butyl, trifluoromethyl, methylthio, and trifluoromethoxy, at the *meta*- or *para*-positions furnished the desired products 6a–6g in moderate yields. Notably, the radical-relay coupling of anilines bearing a Br or I atom posed no problems, affording 6h and 6i in 44% and 46% yields, respectively.

**Scheme 3:**
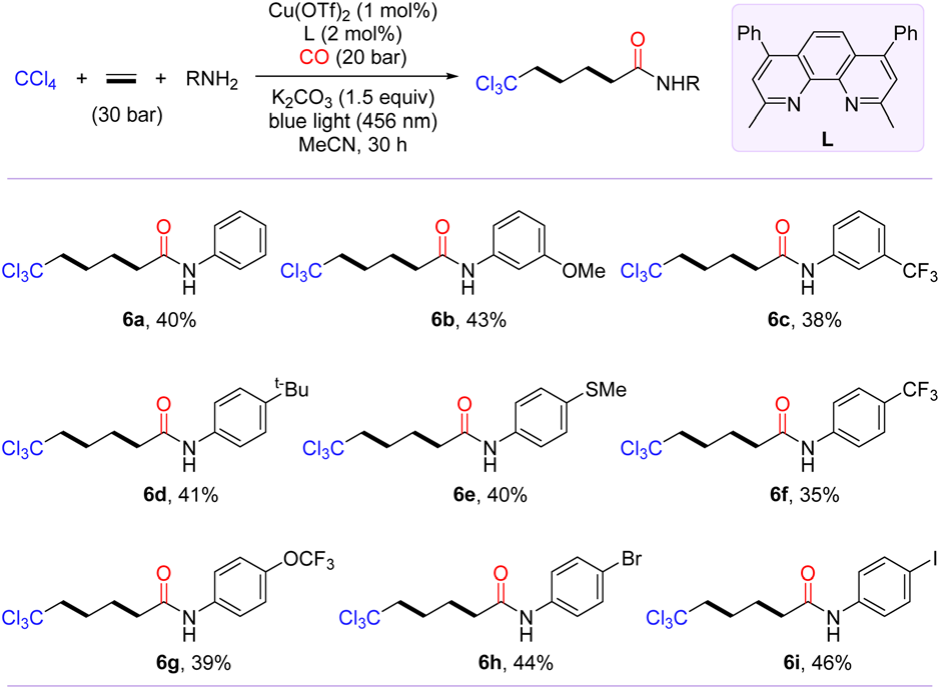
Scope of bis-vinylative carbonylation. [a] Reaction conditions: CCl_4_ (2.0 mmol, 5.0 equiv.), ethylene (30 bar), NuH (0.4 mmol, 1.0 equiv.), Cu(OTf)_2_ (1 mol%), bpy (2 mol%), and K_2_CO_3_ (0.6 mmol, 1.5 equiv.) in MeCN (2.0 mL) at 25–30 °C for 30 h under CO (20 bar); isolated yields.

Based on the results of the above experiments and previous reports, a plausible radical reaction pathway is proposed, as shown in Scheme 4. The catalytic cycle initiates with the *in situ* generation of active Cu^I^Ln species, which reduces CCl_4_*via* single electron transfer under light irradiation to form a trichloromethyl radical and Cu^II^LnX species. Then, the trichloromethyl radical is added to ethylene to give the crucial β-trichloromethyl-substituted carbon radical A. Subsequently, radical A can be transformed into acyl copper intermediate D in two routes: (1) carbon radical A traps CO to form acyl intermediate B, which rapidly recombines with the Cu^II^LnX species to provide intermediate D; (2) carbon radical A is captured by Cu^II^LnX to form Cu(iii) alkyl complex C, which undergoes coordination/insertion of carbon monoxide to give intermediate D. Finally, intermediate D interacts with a nucleophilic reagent in the presence of a base to obtain the desired β-trichloromethyl carboxylic acid derivatives and the active Cu^I^Ln species for the next catalytic cycle. On the other hand, β-trichloromethyl-substituted carbon radical A can be further added to ethylene to form γ-trichloromethyl-substituted carbon radical E, which then undergoes the same pathway as β-trichloromethyl-substituted carbon radical A to eventually give γ-trichloromethyl amides. Products based on the oligomerization and polymerization of ethylene can also be detected.

**Scheme 4:**
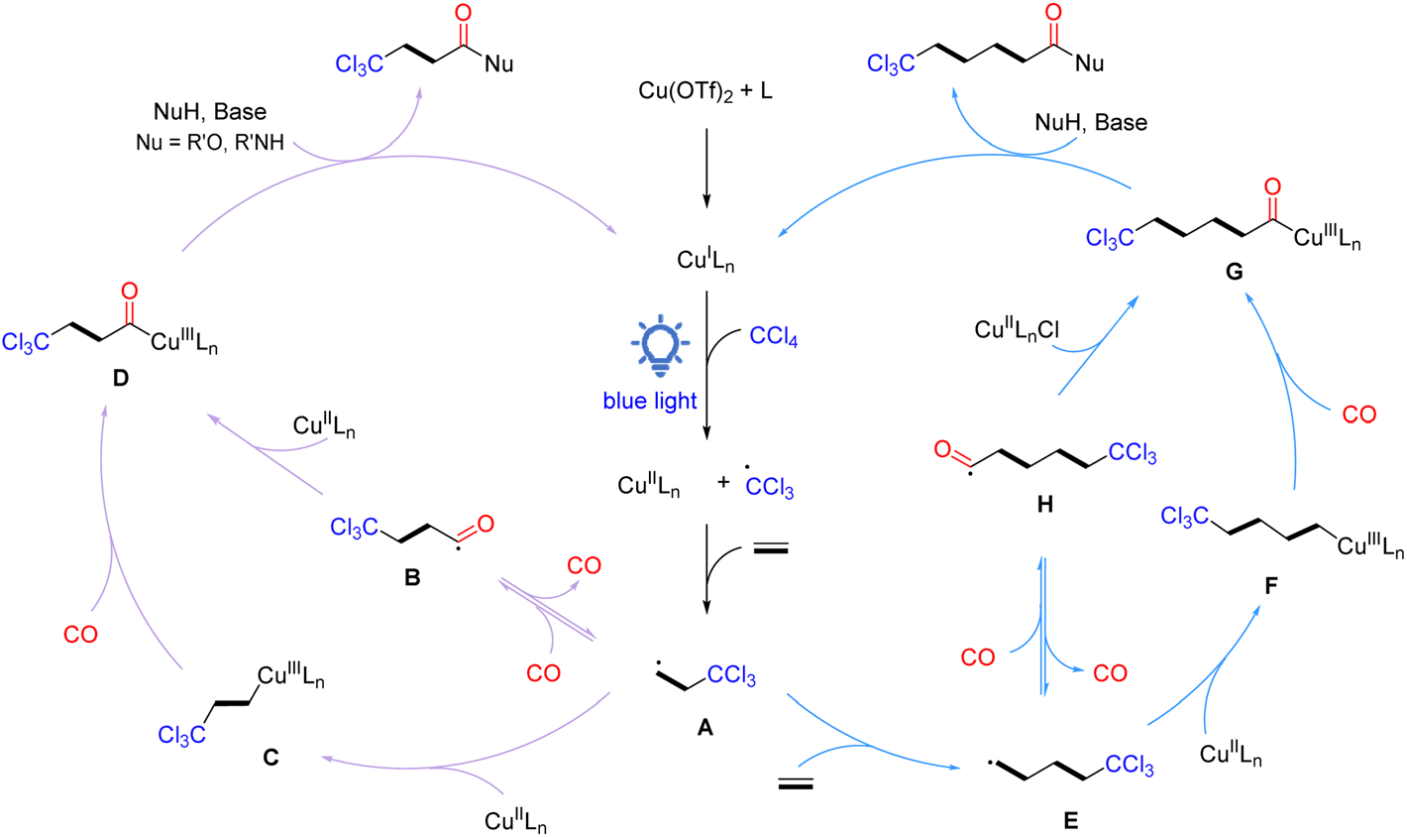
Proposed reaction mechanism.

## Conclusions

In summary, we have developed a copper-catalyzed trichloromethylative carbonylation of ethylene under visible light irradiation by employing inexpensive and readily available CCl_4_ and CO as C1 sources for trichloromethyl and carbonyl, respectively. With this photocatalytic system, a series of functionalized nucleophiles including amines, alcohols, and phenols can be efficiently converted into the corresponding β-trichloromethyl carboxylic acid derivatives. Significantly, this process could be applied for the late-stage functionalization of several complex molecules, in addition to allowing the selectively controlled generation of bis-vinylated γ-trichloromethyl amides.

## Data availability

The data underlying this study are available in the published article and its ESI.†

## Author contributions

X. F. W. and Y. Z. conceived and designed the experiments. Y. Z. and B. H. T. performed the experiments. Y. Z. and X. F. W. wrote the manuscript. X. F. W. directed the research.

## Conflicts of interest

There are no conflicts to declare.

## Supplementary Material

SC-015-D3SC05530B-s001

## References

[cit1] Vaillancourt F. H., Yeh E., Vosburg D. A., Garneau-Tsodikova S., Walsh C. T. (2006). Chem. Rev..

[cit2] Wagner C., Omari M. E., König G. M. (2009). J. Nat. Prod..

[cit3] Jeschke P. (2010). Pest Manage. Sci..

[cit4] Su J.-Y., Zhong Y.-L., Zheng L.-M., Wei S., Wong Q.-W., Mak T. C. W., Zhou Z.-Y. (1993). J. Nat. Prod..

[cit5] Durow A. C., Long G. C., O'Connell S. J., Willis C. L. (2006). Org. Lett..

[cit6] Kazlauskas R., Lidgard R. O., Wells R. J., Vetter W. (1977). Tetrahedron Lett..

[cit7] MacMillan J. B., Trousdale E. K. (2000). Org. Lett..

[cit8] Gu Z. H., Zakarian A. (2010). Angew. Chem., Int. Ed..

[cit9] Sadar M. D., Williams D. E., Mawji N. R., Patrick B. O., Wikanta T., Chasanah E., Irianto H. E., Soest R. V., Andersen R. J. (2008). Org. Lett..

[cit10] Kapojos M. M., Abdjul D. B., Yamazaki H., Ohshiro T., Rotinsulu H., Wewengkang D. S., Sumilat D. A., Tomoda H., Namikoshi M., Uchida R. (2018). Bioorg. Med. Chem. Lett..

[cit11] Shimakoshi H., Luo Z., Inaba T., Hisaeda Y. (2016). Dalton Trans..

[cit12] Kazuo S., Hiroshi K., Toshiaki Y. (1966). Bull. Chem. Soc. Jpn..

[cit13] Joyce R. M., Hanford W. E., Harmon J. (1948). J. Am. Chem. Soc..

[cit14] Aggarwal V. K., Mereu A. (2000). J. Org. Chem..

[cit15] Li Y., Zheng T., Wang W., Xu W., Ma Y., Zhang S., Wang H., Sun Z. (2012). Adv. Synth. Catal..

[cit16] Gupta M. K., Li Z., Snowden T. S. (2014). Org. Lett..

[cit17] Fujita M., Hiyama T. (1985). J. Am. Chem. Soc..

[cit18] Wu N., Wahl B., Woodward S., Lewis W. (2014). Chem. – Eur. J..

[cit19] Kister J., Mioskowski C. (2007). J. Org. Chem..

[cit20] Kharasch M. S., Jensen E. V., Urry W. H. (1945). Science.

[cit21] Tsuji J., Sato K., Nagashima H. (1985). Tetrahedron.

[cit22] Huang F., Luo W., Zhou J. (2023). Chinese J. Org. Chem..

[cit23] Nef R. K., Su Y.-L., Liu S., Rosado M., Zhang X., Doyle M. P. (2019). J. Am. Chem. Soc..

[cit24] Su Y.-L., Tram L., Wherritt D., Arman H., Griffith W. P., Doyle M. P. (2020). ACS Catal..

[cit25] Liang Y.-Y., Huang J., Ouyang X.-H., Qin J.-H., Song R.-J., Li J.-H. (2021). Chem. Commun..

[cit26] Sivaguru P., Ning Y., Bi X. (2021). Chem. Rev..

[cit27] Whyte A., Torelli A., Mirabi B., Zhang A., Lautens M. (2020). ACS Catal..

[cit28] Coppola G. A., Pillitteri S., Van der Eycken E. V., You S.-L., Sharma U. K. (2022). Chem. Soc. Rev..

[cit29] Sun J., Wang L., Zheng G., Zhang Q. (2023). Org. Chem. Front..

[cit30] Wang Q., Wang M., Wu Q., Ma M., Zhao B. (2022). Org. Lett..

[cit31] Kusakabe M., Nagao K., Ohmiya H. (2021). Org. Lett..

[cit32] Liu B., Hu F., Shi B.-F. (2015). ACS Catal..

[cit33] Otera J. and Nishikido J., Esterification: Methods, Reactions, and Applications, Wiley-VCH, Weinheim, 2010

[cit34] Lundberg H., Tinnis F., Selander N., Adolfsson H. (2014). Chem. Soc. Rev..

[cit35] Dunetz J. R., Magano J., Weisenburger G. A. (2016). Org. Process Res. Dev..

[cit36] Tsuji J., Sato K., Nagashima H. (1985). Tetrahedron.

[cit37] Wu X.-F. , Han B., Ding K. and Liu Z., The Chemical Transformations of C1 Compounds, John Wiley & Sons, 2022

[cit38] Peng J.-B., Wu F.-P., Wu X.-F. (2019). Chem. Rev..

[cit39] Xu J.-X., Yuan Y., Wu X.-F. (2023). Coord. Chem. Rev..

[cit40] Gabriele B. , Carbon Monoxide in Organic Synthesis: Carbonylation Chemistry, Wiley-VCH, Verlag, 2021

[cit41] Lu B., Zhang Z., Jiang M., Liang D., He Z.-W., Bao F.-S., Xiao W.-J., Chen J.-R. (2023). Angew. Chem., Int. Ed..

[cit42] Zhu J., Xu T., Lu P., Chen W., Lu W. (2022). Mol. Catal..

[cit43] Sumino S., Fusano A., Fukuyama T., Ryu I. (2014). Acc. Chem. Res..

[cit44] Cheung K. P. S., Sarkar S., Gevorgyan V. (2022). Chem. Rev..

[cit45] Wu C., Hui X., Zhang D., Zhang M., Zhu Y., Wang S. (2022). Green Chem..

[cit46] Wu X., Xiang C., Yu J.-T. (2023). Eur. J. Org Chem..

[cit47] Arora S., Singh T., Mondal U., Singh A. (2023). Eur. J. Org Chem..

[cit48] Zhang Y., Geng H.-Q., Wu X.-F. (2021). Angew. Chem., Int. Ed..

[cit49] Zhang Y., Wu X.-F. (2020). Chem. Commun..

[cit50] Zhang Y., Yuan Y., Geng H.-Q., Xu J.-X., Wu X.-F. (2022). J. Catal..

[cit51] Keiko K.-O., Kazuma H., Shinya Y., Yoshihiro K. (2010). Bull. Chem. Soc. Jpn..

[cit52] Michaels H., Rinderle M., Freitag R., Benesperi I., Edvinsson T., Socher R., Gagliardi A., Freitag M. (2020). Chem. Sci..

[cit53] Saygili Y., Söderberg M., Pellet N., Giordano F., Cao Y., Muñoz-Garcia A. B., Zakeeruddin S. M., Vlachopoulos N., Pavone M., Boschloo G., Kavan L., Moser J.-E., Grätzel M., Hagfeldt A., Freitag M. (2016). J. Am. Chem. Soc..

[cit54] Morgen T. O., Baur M., Göttker-Schnetmann I., Mecking S. (2020). Nat. Commun..

[cit55] Chen S.-Y., Ren B.-H., Li S.-H., Song Y.-H., Jiao S., Zou C., Chen C., Lu X.-B., Liu Y. (2022). Angew. Chem., Int. Ed..

[cit56] Zhu L., Gaire S., Ziegler C. J., Jia L. (2022). ChemCatChem.

