# Supplementary material for: Copper-catalyzed trichloromethylative carbonylation of ethylene
Source: Chem Sci. 2023 Dec 14;15(4):1418–23. doi: 10.1039/d3sc05530b (PMC10806816; doi:10.1039/d3sc05530b)
Supplement: SC-015-D3SC05530B-s001 [file SC-015-D3SC05530B-s001.pdf]

Electronic Supplementary Material (ESI) for Chemical Science.
This journal is © The Royal Society of Chemistry 2023

Supporting Information

Copper-Catalyzed Trichloromethylative Carbonylation of Ethylene

Youcan Zhang,†[b,c] Bing-Hong Teng,†[a,c] and Xiao-Feng Wu*[c,d]

[a] School of Chemistry and Chemical Engineering, Liaoning Normal University, 850 Huanghe Road, Dalian 116029, China; [b] College of Chemistry and Chemical Engineering, Shanghai University of Engineering Science, Shanghai 201620, China; [c] Dalian National Laboratory for Clean Energy, Dalian Institute of Chemical Physics, Chinese Academy of Sciences, 116023 Dalian, Liaoning, China; [d] Leibniz-Institut für Katalyse e.V., Albert-Einstein-Straβe 29a, 18059 Rostock, Germany

* E-Mail: xwu2020@dicp.ac.cn

Contents

1. General conditions 2

2. General procedures 3

   2.1 General Procedure: trichloromethylative carbonylation of ethylene 3

   2.2 Supplementary Tables 4

3. Analytical data 7

5. ^{1}H-, ^{13}C- and ^{19}F-NMR spectra copy of products 25

1. General conditions

Unless stated otherwise, all reactions were carried out under a carbon monoxide or nitrogen atmosphere. All reagents and solvents were purchased from Adamas-beta, Energy Chemical, Sigma-Aldrich, Bidepharm, TCI, or Alfa Aesar and used without purification. Anhydrous solvents were purchased from Energy Chemical and used as received. NMR spectra were recorded at ambient temperature using Bruker Avance III 400 MHz NMR and Bruker AVANCE III HD 700MHz NMR spectrometers. Chemical shifts (ppm) are given relative to solvent: references for $CDCl_3$ were 7.26 ppm (^1H NMR) and 77.0 ppm (^{13}C NMR); CD_3OD were 3.31 ppm (^1H NMR) and 49.0 ppm (^{13}C NMR); DMSO-d_6 were 2.50 ppm (^1H NMR) and 39.5 ppm (^{13}C NMR). Multiplets were assigned as s (singlet), br.s (broad singlet), d (doublet), t (triplet), q (quartet), dd (doublet of doublets), dt (doublet of triplets), td (triplet of doublets) and m (multiplet). GC-yields were calculated using hexadecane as an internal standard. HRMS data was obtained with Micromass HPLC-Q-TOF mass spectrometer (ESI) or Agilent 6540 Accurate-MS spectrometer (Q-TOF). Analytical thin layer chromatography (TLC) was carried out using pre-coated (0.20 mm thickness) silica gel plates with F254 indicator. The products were isolated from the reaction mixture by column chromatography on silica gel 200-300 mesh. Because of the high toxicity of carbon monoxide, all of the reactions should be performed in an autoclave. The laboratory should well-equipped with a CO detector and alarm system. For blue light irradiation, two Kessil PR160L-456 nm blue lamps (19 V DC 40 W Max) were placed ca. 7 cm from the reaction vial inside the autoclave at room temperature.

2. General procedures

2.1 General Procedure: trichloromethylative carbonylation of ethylene

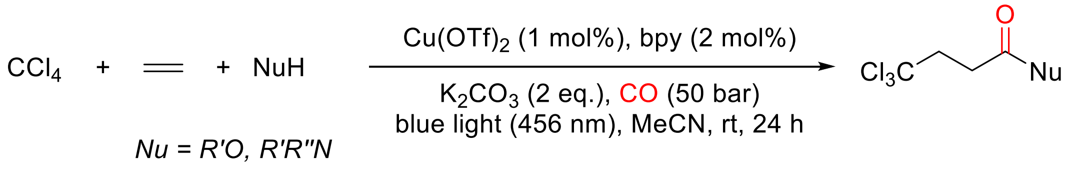

To each screw-cap vial (4 ml) equipped with a septum, a small cannula, and a stirring bar was added $Cu(OTf)_2$ (1.5 mg, 1 mol%), bpy (1.3 mg, 2 mol%), K_2CO_3 (110.6 mg, 0.8 mmol, 2.0 equiv.), and NuH (0.4 mmol, if the nucleophile is solid). The vials then were purged with argon three times before added dry MeCN (2 mL), CCl_4 (200 uL, 2 mmol, 5.0 equiv.), and NuH (0.4 mmol, if the nucleophile is liquid). These vials were transferred into a 500 mL photoautoclav. The closed autoclave was flushed one time with nitrogen (~ 5 bar), two times with CO (~ 5 bar), and a pressure of ethylene (1 bar) and CO (50 bar) were charged. The autoclave was then placed on a magnetic stirrer. The reaction mixture was stirred while being irradiated with 40 w blue light (Kessil PR160L-456 nm, intensity = 50) at room temperature (25-30 ^{o}C) for 24 h. After irradiation, the light was turned off and the pressure was released carefully. The organic phase was removed under reduced pressure and the crude products were purified by column chromatography on silica gel (eluent: pentane/ethyl acetate = 40:1 to pentane/ethyl acetate/ dichloromethane = 1:1:1).

Notably: the substrate expansion reactions were performed on a 0.4 mmol scale unless otherwise noted.

2 mmol scale: To a screw-cap vial (15 ml) equipped with a septum, a small cannula, and a stirring bar was added $Cu(OTf)_2$ (3.6 mg, 0.5 mol%), bpy (3.2 mg, 1 mol%), K_2CO_3 (552.8 mg, 4 mmol, 2.0 equiv.), and NuH (2.0 mmol, if the nucleophile is solid). The vials then were purged with argon three times before added dry MeCN (10 mL), CCl_4 (1.0 mL, 10 mmol, 5.0 equiv.), and NuH (2.0 mmol, if the nucleophile is liquid). These vials were transferred into a 500 mL photoautoclav. The closed autoclave was flushed one time with nitrogen (~ 5 bar), two times with CO (~ 5 bar), and a pressure of ethylene (1 bar) and CO (50 bar) were charged. The autoclave was then placed on a magnetic stirrer. The reaction mixture was stirred while being irradiated with 40 w blue light (Kessil PR160L-456 nm, intensity = 50) at room temperature (25-30 ^{o}C) for 30 h. After irradiation, the light was turned off and the pressure was released carefully. The organic phase was removed under reduced pressure and the crude products were purified by column chromatography on silica gel (eluent: pentane/ethyl acetate = 30:1 to pentane/ethyl acetate/ dichloromethane = 10:1:1).

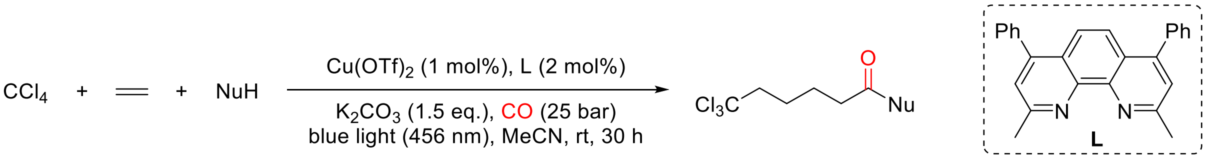

To each screw-cap vial (4 ml) equipped with a septum, a small cannula, and a stirring bar was added $Cu(OTf)_2$ (1.5 mg, 1 mol%), L (3.0 mg, 2 mol%), K_2CO_3 (82.9 mg, 0.6 mmol, 1.5 equiv.), and NuH (0.4 mmol, if the nucleophile is solid). The vials then were purged with argon three times before added dry MeCN (2 mL), CCl_4 (100 uL, 1 mmol, 2.5 equiv.), and NuH (0.4 mmol, if the nucleophile is liquid). These vials were transferred into

a 500 mL photoautoclav. The closed autoclave was flushed one time with nitrogen (~ 5 bar), two times with CO (~ 5 bar), and a pressure of ethylene (35 bar) and CO (25 bar) were charged. The autoclave was then placed on a magnetic stirrer. The reaction mixture was stirred while being irradiated with 40 w blue light (Kessil PR160L-456 nm, intensity = 50) at room temperature (25-30 °C) for 24 h. After irradiation, the light was turned off and the pressure was released carefully. The organic phase was removed under reduced pressure and the crude products were purified by column chromatography on silica gel (eluent: pentane/ethyl acetate = 40:1 to pentane/ethyl acetate/ dichloromethane = 6:1:1).

2.2 Supplementary Tables

Table S1. Screening of catalyst and base[a]

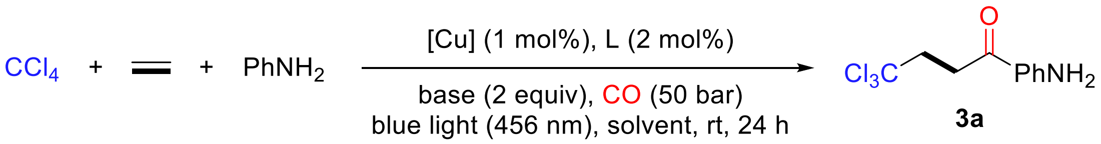

3a

Entry	Catalyst	Ligand	Base	Solvent	Yield (%)[a]
1[c]	$Cu(OTf)_2$	BINAP	K_2CO_3	MeCN	44
2[c]	$Cu(OTf)_2$	1,10-Phen	K_2CO_3	MeCN	55
3	$Cu(OTf)_2$	1,10-Phen	K_2CO_3	MeCN	78
4	$Cu(OTf)_2$	bpy	K_2CO_3	MeCN	80 (77)[b]
5	$Cu(OTf)_2$	4,4'-diOMe-2,2'-bpy	K_2CO_3	MeCN	76
6	$Cu(OTf)_2$	6,6'-diMe-2,2'-bpy	K_2CO_3	MeCN	60
7	$Cu(OTf)_2$	2,9-diMe-1,10-Phen	K_2CO_3	MeCN	43
8	$Cu(OAc)_2$	bpy	K_2CO_3	MeCN	75
9	CuI	bpy	K_2CO_3	MeCN	76
10	$Cu(OTf)_2$	bpy	Na_2CO_3	MeCN	79
11	$Cu(OTf)_2$	bpy	K_3PO_4	MeCN	76
12	$Cu(OTf)_2$	bpy	Et_3N	MeCN	22
13	$Cu(OTf)_2$	bpy	K_2CO_3	$PhCF_3$	53
14	$Cu(OTf)_2$	bpy	K_2CO_3	1,4-dioxane	65
15	$Cu(OTf)_2$	bpy	K_2CO_3	DCE	57

[a] Reaction conditions: CCl_4 (1.0 mmol), ethylene (1 bar), $PhNH_2$ (0.2 mmol), [Cu] (1 mol%), Ligand (2 mol%), and Base (2.0 equiv.) in solvent (1.0 mL) at rt (25-30 °C) for 24 h under CO (50 bar). Yields were determined by GC-FID analysis using n-hexadecane as internal standard. [b] Yield of isolated product. [c] CO (40 bar).

Table S2. Optimization of reaction conditions[a]

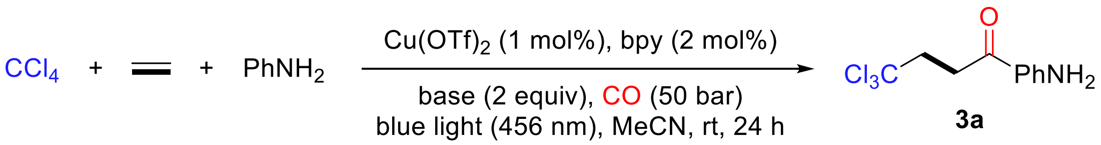

Entry	Variations from the standard conditions	Yield (%)[a]
1	0.5 mmol of CCl_4 added	74
2	$Cu(OTf)_2$ (2.5 mol%), bpy (5 mol%) added	76
3	1.5 equiv of K_2CO_3 added	78
4	1.2 equiv of K_2CO_3 added	76
5	no bpy	24
6	no $Cu(OTf)_2$, no K_2CO_3, or no light	n.r.
7	$[Cu(dmp)_2]Cl$ instead of $Cu(OTf)_2$ and bpy	66
8	$[Cu(dmp)_2Cl]Cl$ instead of $Cu(OTf)_2$ and bpy	62
9	$[Cu(dmp)XantPhos]PF_6$ instead of $Cu(OTf)_2$ and bpy	50
10	$[Cu(bpy)_2](OTf)_2$ instead of $Cu(OTf)_2$ and bpy	70
11	no light, 40 °C	56
12	no light, 80 °C	76

[a] Reaction conditions: CCl_4 (1.0 mmol), ethylene (1 bar), $PhNH_2$ (0.2 mmol), $Cu(OTf)_2$ (1 mol%), bpy (2 mol%), and K_2CO_3 (2.0 equiv.) in MeCN (1.0 mL) at rt (25-30 °C) for 24 h under CO (50 bar). Yields were determined by GC-FID analysis using n-hexadecane as internal standard.

Table S3. Optimization of reaction conditions[a]

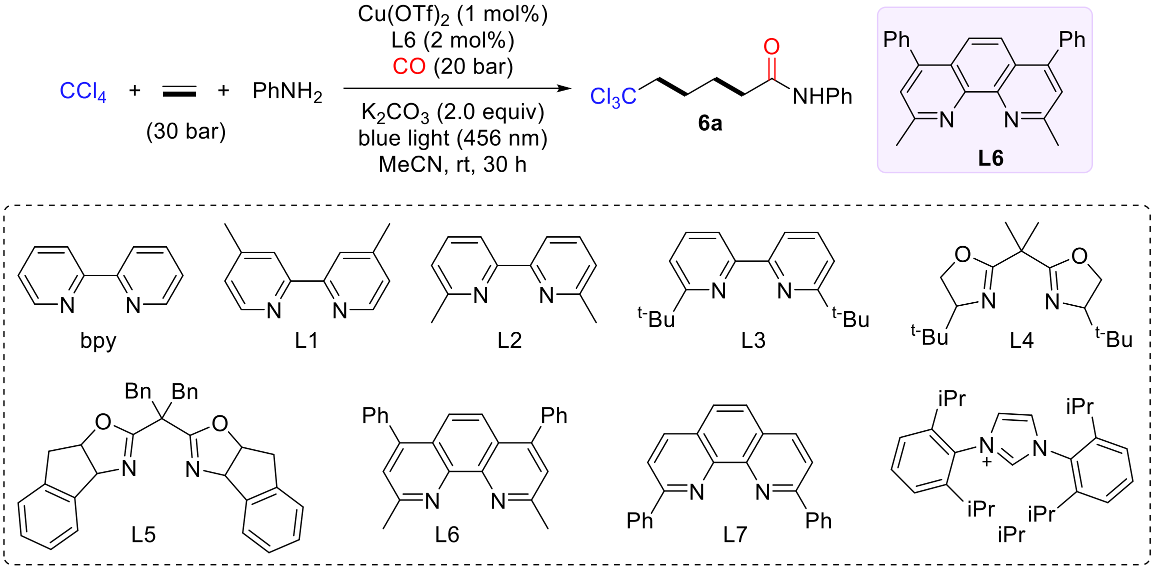

Entry	Variations from the standard conditions	Yield (%)[a]
1	none	36
2	Bpy instead of L6	22
3	L1 instead of L6	21

4	L2 instead of L6	22
5	L3 instead of L6	27
6	L4 instead of L6	32
7	L5 instead of L6	35
8	L7 instead of L6	37
9	iPrCuCl instead of $Cu(OTf)_2$ and L6	34

[a] Reaction conditions: CCl_4 (1.0 mmol), ethylene (30 bar), $PhNH_2$ (0.2 mmol), $Cu(OTf)_2$ (1 mol%), bpy (2 mol%), and K_2CO_3 (2.0 equiv.) in MeCN (1.0 mL) at rt (25-30 °C) for 24 h under CO (20 bar). Yields were determined by GC-FID analysis using n-hexadecane as internal standard.

Table S4. Optimization of reaction conditions[a]

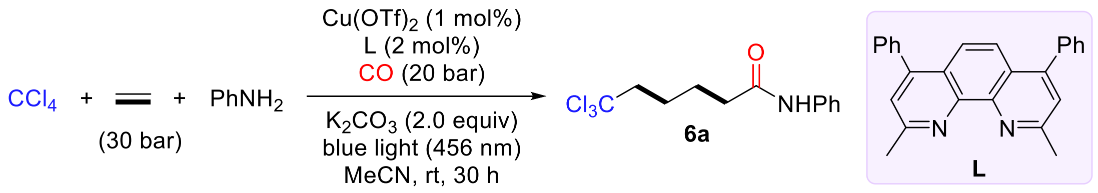

Entry	Variations from the standard conditions	Yield (%)[a]
1	ethylene = (30 bar), CO (10 bar)	25
2	ethylene = (30 bar), CO (15 bar)	32
3	ethylene = (30 bar), CO (30 bar)	27
4	ethylene = (35 bar), CO (30 bar)	23
5	ethylene = (40 bar), CO (30 bar)	18
6[c]	ethylene = (30 bar), CO (20 bar)	39
7[c]	0.5 mmol of CCl_4 added	43 (41)[b]
8[c]	0.4 mmol of CCl_4 added	40
9[c]	0.6 mmol of CCl_4 added	42

[a] Reaction conditions: CCl_4 (1.0 mmol), ethylene (30 bar), $PhNH_2$ (0.2 mmol), $Cu(OTf)_2$ (1 mol%), bpy (2 mol%), and K_2CO_3 (2.0 equiv.) in MeCN (1.0 mL) at rt (25-30 °C) for 24 h under CO (20 bar). Yields were determined by GC-FID analysis using n-hexadecane as internal standard. [b] Yield of isolated product.

3. Analytical data

4,4,4-Trichloro-*N*-phenylbutanamide (**3a**)

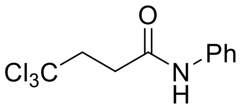

79.9 mg, light yellow solid, yield: 75%. Eluent: pentane/ethyl acetate/dichloromethane = 10/1/1.

^{1}H NMR (400 MHz, $CDCl_3$) δ 7.49 (d, *J* = 7.7 Hz, 2H), 7.45 (s, 1H), 7.32 (t, *J* = 7.9 Hz, 2H), 7.13 (t, *J* = 7.4 Hz, 1H), 3.31 – 3.03 (m, 2H), 2.95 – 2.70 (m, 2H).

^{13}C NMR (101 MHz, $CDCl_3$) δ 168.1, 137.4, 129.1, 124.7, 120.0, 98.9, 50.1, 34.3.

HR-MS (ESI-TOF) m/z: $[M+H]^+$ calcd. for $C_{10}H_{11}Cl_3NO$ 265.9901, found 265.9902.

4,4,4-Trichloro-*N*-(2-isopropylphenyl)butanamide (**3b**)

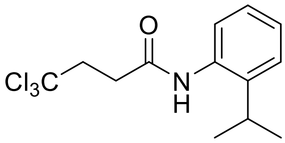

41.1 mg, light yellow solid, yield: 66%. Eluent: pentane/ethyl acetate/dichloromethane = 10/1/1.

^{1}H NMR (400 MHz, $CDCl_3$) δ 7.68 (s, 1H), 7.39 (d, *J* = 8.4 Hz, 1H), 7.26 (d, *J* = 8.1 Hz, 1H), 7.17 (t, *J* = 6.8 Hz, 1H), 7.11 (t, *J* = 7.6 Hz, 1H), 3.14 – 3.07 (m, 2H), 3.08-2.96 (m, 1H), 2.87 – 2.76 (m, 2H), 1.18 (d, *J* = 6.9 Hz, 6H).

^{13}C NMR (101 MHz, $CDCl_3$) δ 169.0, 141.8, 133.4, 126.7, 126.2, 125.73, 125.67, 99.0, 50.2, 33.6, 27.8, 23.1.

HR-MS (ESI-TOF) m/z: $[M+H]^+$ calcd. for $C_{13}H_{17}Cl_3NO$ 308.0370, found 308.0375.

4,4,4-Trichloro-*N*-(3-(trifluoromethyl)phenyl)butanamide (**3c**)

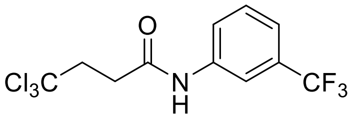

121.7 mg, light yellow solid, yield: 90%. Eluent: pentane/ethyl acetate/dichloromethane = 10/1/1.

^{1}H NMR (400 MHz, CD_3OD) δ 8.01 (t, *J* = 2.1 Hz, 1H), 7.74 (dt, *J* = 8.2, 1.4 Hz, 1H), 7.46 (t, *J* = 8.0 Hz, 1H), 7.34 (d, *J* = 8.0 Hz, 1H), 4.87 (s, 1H), 3.18 – 3.10 (m, 2H), 2.94 – 2.87 (m, 2H).

^{13}C NMR (101 MHz, CD_3OD) δ 171.2, 140.6, 132.1 (q, *J* = 32.2 Hz), 130.6, 125.43 (q, *J* = 271.4 Hz), 124.0, 121.3 (q, *J* = 4.1 Hz), 117.3 (q, *J* = 4.2 Hz), 100.4, 51.2, 34.7.

^{19}F NMR (376 MHz, CD_3OD) δ -64.2.

HR-MS (ESI-TOF) m/z: $[M+H]^+$ calcd. for $C_{11}H_{10}Cl_3F_3NO$ 333.9775, found 333.9781.

4,4,4-Trichloro-*N*-(3-methoxyphenyl)butanamide (**3d**)

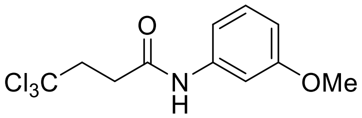

71.7 mg, light yellow oil, yield: 60%. Eluent: pentane/ethyl acetate/dichloromethane = 10/1/1 to 6/1/1.

^{1}H NMR (400 MHz, $CDCl_3$) δ 7.95 (s, 1H), 7.23 (t, *J* = 2.3 Hz, 1H), 7.18 (t, *J* = 8.2 Hz, 1H), 6.97 (dd, *J* = 7.9, 2.0 Hz, 1H), 6.66 (dd, *J* = 8.3, 2.5 Hz, 1H), 3.75 (s, 3H), 3.17 – 3.09 (m, 2H), 2.86 – 2.78 (m, 2H).

^{13}C NMR (101 MHz, $CDCl_3$) δ 168.5, 160.0, 138.6, 129.7, 112.3, 110.3, 106.1, 98.9, 55.2, 50.0, 34.1.

HR-MS (ESI-TOF) m/z: $[M+H]^+$ calcd. for $C_{11}H_{13}Cl_3NO_2$ 296.0006, found 293.0009.

4,4,4-Trichloro-*N*-(4-(trifluoromethyl)phenyl)butanamide (**3e**)

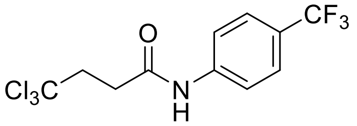

110.8 mg, light yellow solid, yield: 83%. Eluent: pentane/ethyl acetate/dichloromethane = 10/1/1.

^{1}H NMR (400 MHz, DMSO-d_6) δ 10.49 (s, 1H), 7.80 (d, *J* = 8.5 Hz, 2H), 7.64 (d, *J* = 8.6 Hz, 2H), 3.20 – 3.07 (m, 2H), 2.93 – 2.77 (m, 2H).

^{13}C NMR (101 MHz, DMSO-d_6) δ 168.9, 142.5, 126.0 (q, *J* = 3.8 Hz), 124.3 (q, *J* = 271.2 Hz), 123.3 (q, *J* = 32.0 Hz), 118.9, 99.7, 49.4, 33.5.

^{19}F NMR (376 MHz, DMSO-d_6) δ -60.5.

HR-MS (ESI-TOF) m/z: $[M+H]^+$ calcd. for $C_{11}H_{10}Cl_3F_3NO$ 333.9775, found 333.9776.

4,4,4-Trichloro-*N*-(4-(trifluoromethoxy)phenyl)butanamide (**3f**)

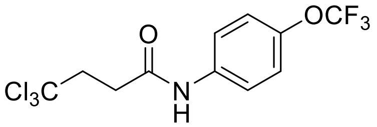

120.3 mg, light yellow solid, yield: 86%. Eluent: pentane/ethyl acetate/dichloromethane = 10/1/1.

^{1}H NMR (400 MHz, $CDCl_3$) δ 8.29 (s, 1H), 7.48 (d, *J* = 9.0 Hz, 2H), 7.12 (d, *J* = 8.6 Hz, 2H), 3.20 – 3.01 (m, 2H), 2.95 – 2.78 (m, 2H).

^{13}C NMR (101 MHz, $CDCl_3$) δ 169.0, 145.6 (q, *J* = 2.1 Hz), 135.9, 121.7, 120.4 (q, *J* = 257.9 Hz), 98.7, 50.0, 34.0.

^{19}F NMR (376 MHz, $CDCl_3$) δ -58.2.

HR-MS (ESI-TOF) m/z: $[M+H]^+$ calcd. for $C_{11}H_{10}Cl_3F_3NO_2$ 349.9724, found 349.9724.

4,4,4-Trichloro-*N*-(4-bromophenyl)butanamide (**3g**)

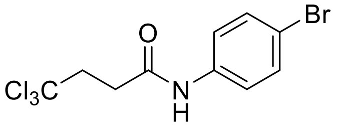

106.7 mg, light yellow solid, yield: 77%. Eluent: pentane/ethyl acetate/dichloromethane = 10/1/1.

^{1}H NMR (400 MHz, DMSO-d_6) δ 10.26 (s, 1H), 7.57 (d, *J* = 8.9 Hz, 2H), 7.46 (d, *J* = 8.8 Hz, 2H), 3.18 – 3.05 (m, 2H), 2.89 – 2.73 (m, 2H).

^{13}C NMR (101 MHz, DMSO-d_6) δ 168.3, 138.3, 131.5, 120.9, 114.8, 99.7, 49.5, 33.4.

HR-MS (ESI-TOF) m/z: $[M+H]^+$ calcd. for $C_{10}H_{10}BrCl_3NO$ 343.9006, found 343.9004.

4,4,4-Trichloro-*N*-(4-iodophenyl)butanamide (**3h**)

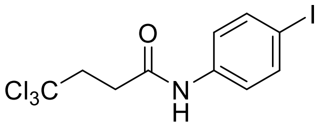

128.2 mg, light yellow solid, yield: 82%. Eluent: pentane/ethyl acetate/dichloromethane = 10/1/1.

^{1}H NMR (400 MHz, DMSO-d_6) δ 10.23 (s, 1H), 7.62 (d, *J* = 8.7 Hz, 2H), 7.43 (d, *J* = 8.8 Hz, 2H), 3.18 – 3.02 (m, 2H), 2.88 – 2.72 (m, 2H).

^{13}C NMR (101 MHz, DMSO-d_6) δ 168.3, 138.8, 137.3, 121.2, 99.7, 86.6, 49.5, 33.4.

HR-MS (ESI-TOF) m/z: $[M+H]^+$ calcd. for $C_{10}H_{10}ICl_3NO$ 391.8867, found 391.8871.

4,4,4-Trichloro-*N*-(4-(methylthio)phenyl)butanamide (**3i**)

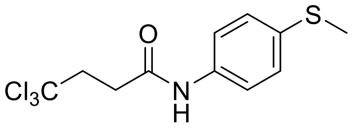

84.5 mg, light yellow solid, yield: 68%. Eluent: pentane/ethyl acetate/dichloromethane = 8/1/1.

^{1}H NMR (400 MHz, $CDCl_3$) δ 8.00 (s, 1H), 7.37 (d, *J* = 8.5 Hz, 2H), 7.17 (d, *J* = 8.6 Hz, 2H), 3.16 – 3.05 (m, 2H), 2.85 – 2.76 (m, 2H), 2.43 (s, 3H).

^{13}C NMR (101 MHz, $CDCl_3$) δ 168.5, 134.8, 134.2, 127.6, 121.0, 98.8, 50.0, 34.0, 16.4.

HR-MS (ESI-TOF) m/z: $[M+H]^+$ calcd. for $C_{11}H_{13}Cl_3NOS$ 311.9778, found 311.9785.

4,4,4-Trichloro-*N*-(4-phenoxyphenyl)butanamide (**3j**)

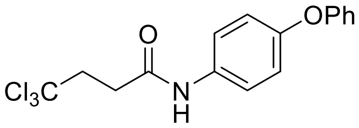

91.1 mg, light yellow oil, yield: 64%. Eluent: pentane/ethyl acetate/dichloromethane = 10/1/1 to 8/1/1.

^{1}H NMR (400 MHz, $CDCl_3$) δ 8.01 (s, 1H), 7.43 (d, *J* = 8.9 Hz, 2H), 7.32 (dd, *J* = 8.6, 7.3 Hz, 2H), 7.09 (t, *J* = 7.4 Hz, 1H), 7.00 – 6.91 (m, 4H), 3.19 – 3.11 (m, 2H), 2.88 – 2.79 (m, 2H).

^{13}C NMR (101 MHz, $CDCl_3$) δ 168.5, 157.2, 153.9, 132.8, 129.7, 123.2, 122.1, 119.4, 118.5, 98.9, 50.1, 34.0.

HR-MS (ESI-TOF) m/z: $[M+H]^+$ calcd. for $C_{16}H_{15}Cl_3NO_2$ 358.0163, found 358.0171.

4,4,4-Trichloro-*N*-(4-acetylphenyl)butanamide (**3k**)

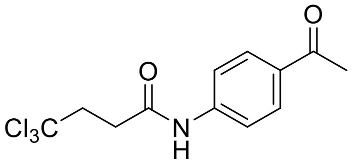

52.5 mg, light yellow solid, yield: 43%, 95% purity. Eluent: pentane/ethyl acetate/dichloromethane = 10/1/1 to 6/1/1.

^{1}H NMR (400 MHz, DMSO-d_6) δ 10.47 (s, 1H), 7.92 (d, *J* = 8.6 Hz, 2H), 7.72 (d, *J* = 8.8 Hz, 1H), 3.18 – 3.07 (m, 2H), 2.97 – 2.80 (m, 2H), 2.52 (s, 3H).

^{13}C NMR (1101 MHz, DMSO-d_6) δ 196.4, 168.8, 143.3, 131.7, 129.5, 118.3, 99.7, 49.4, 33.5, 26.4.

HR-MS (ESI-TOF) m/z: $[M+H]^+$ calcd. for $C_{12}H_{13}Cl_3NO_2$ 308.0006, found 308.0008.

4,4,4-Trichloro-*N*-(4-cyanophenyl)butanamide (**3l**)

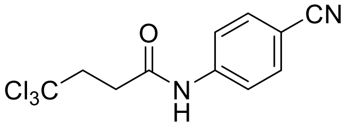

81.2 mg, light yellow solid, yield: 70%. Eluent: pentane/ethyl acetate/dichloromethane = 10/1/1 to 6/1/1.

^{1}**H NMR** (400 MHz, DMSO-d_6) δ 10.55 (s, 1H), 7.85 – 7.66 (m, 4H), 3.19 – 3.05 (m, 2H), 2.91 – 2.75 (m, 2H).

^{13}**C NMR** (101 MHz, DMSO-d_6) δ 169.1, 143.1, 133.2, 119.0, 119.0, 105.0, 99.6, 49.3, 33.5.

HR-MS (ESI-TOF) m/z: $[M+H]^+$ calcd. for $C_{11}H_{10}Cl_3N_2O$ 290.9853, found 290.9853.

4,4,4-Trichloro-*N*-(4-acetamidophenyl)butanamide (**3m**)

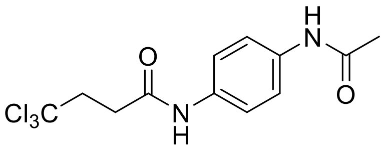

32.6 mg, light yellow solid, yield: 25%. Eluent: pentane/ethyl acetate/dichloromethane = 1/1/1 to 1/2/1.

^{1}**H NMR** (400 MHz, DMSO-d_6) δ 10.06 (s, 1H), 9.86 (s, 1H), 7.59 – 7.37 (m, 4H), 3.19 – 3.00 (m, 2H), 2.84 – 2.71 (m, 2H), 2.01 (s, 3H).

^{13}**C NMR** (101 MHz, DMSO-d_6) δ 167.9, 167.8, 134.8, 134.3, 119.5, 119.4, 99.8, 49.7, 33.2, 23.9.

HR-MS (ESI-TOF) m/z: $[M+H]^+$ calcd. for $C_{12}H_{14}Cl_3N_2O_2$ 323.0115, found 323.0120.

4,4,4-Trichloro-*N*-(pyridin-2-yl)butanamide (**3n**)

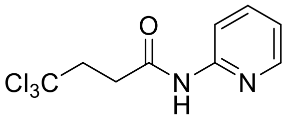

71.5 mg, light yellow solid, yield: 67%, 95% purity. Eluent: pentane/ethyl acetate/dichloromethane = 5/1/1 to 3/1/1.

^{1}**H NMR** (400 MHz, $CDCl_3$) δ 9.98 (s, 1H), 8.31 (dd, *J* = 4.9, 1.8 Hz, 1H), 8.22 (d, *J* = 8.4 Hz, 1H), 7.72 (td, *J* = 8.4, 7.9, 1.9 Hz, 1H), 7.06 (dd, *J* = 7.4, 5.0 Hz, 1H), 3.29 – 3.05 (m, 2H), 2.99 – 2.76 (m, 2H).

^{13}**C NMR** (101 MHz, $CDCl_3$) δ 168.9, 151.7, 147.4, 138.8, 119.9, 114.8, 98.8, 49.7, 34.0.

HR-MS (ESI-TOF) m/z: $[M+H]^+$ calcd. for $C_9H_{10}Cl_3N_2O$ 266.9853, found 266.9860.

4,4,4-Trichloro-*N*-(pyridin-3-yl)butanamide (**3o**)

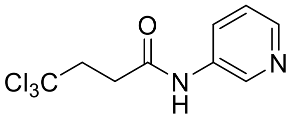

50.8 mg, light yellow solid, yield: 47%. Eluent: pentane/ethyl acetate/dichloromethane = 1/1/1 to 1/2/1.

^{1}**H NMR** (400 MHz, $CDCl_3$) δ 9.47 (s, 1H), 8.64 (d, *J* = 2.6 Hz, 1H), 8.33 (dd, *J* = 4.8, 1.5 Hz, 1H), 8.18 (dt, *J* = 8.4, 2.0 Hz, 1H), 7.30 (dd, *J* = 8.4, 4.8 Hz, 1H), 3.24 – 3.09 (m, 2H), 2.97 – 2.82 (m, 2H).

^{13}**C NMR** (101 MHz, $CDCl_3$) δ 169.3, 144.7, 140.8, 135.4, 127.8, 124.1, 98.8, 49.9, 33.9.

HR-MS (ESI-TOF) m/z: $[M+H]^+$ calcd. for $C_9H_{10}Cl_3N_2O$ 266.9853, found 266.9859.

4,4,4-Trichloro-*N*-methyl-*N*-phenylbutanamide (**3p**)

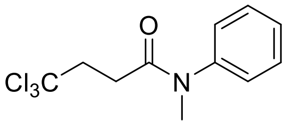

64.5 mg, light yellow oil, yield: 57%. Eluent: pentane/ethyl acetate/dichloromethane = 10/1/1.

^{1}**H NMR** (400 MHz, $CDCl_3$) δ 7.43 (t, *J* = 7.6 Hz, 2H), 7.35 (t, *J* = 7.4 Hz, 1H), 7.23 – 7.13 (m, 2H), 3.26 (s, 3H), 3.14 – 2.97 (m, 2H), 2.58 – 2.44 (m, 2H).

13C NMR (101 MHz, $CDCl_3$) δ 169.9, 143.3, 129.9, 128.1, 127.1, 99.0, 50.5, 37.4, 31.3.

HR-MS (ESI-TOF) m/z: $[M+H]^+$ calcd. for $C_{11}H_{13}Cl_3NO$ 280.0057, found 280.0063.

4,4,4-Trichloro-1-(indolin-1-yl)butan-1-one (**3q**)

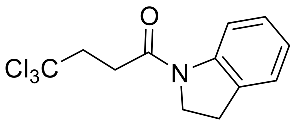

30.7 mg, light yellow solid, yield: 26%, 95% purity. Eluent: pentane/ethyl acetate/dichloromethane = 15/1/1.

1H NMR (400 MHz, $CDCl_3$) δ 8.20 (d, *J* = 8.0 Hz, 1H), 7.21 (t, *J* = 7.9 Hz, 2H), 7.04 (t, *J* = 7.7 Hz, 1H), 4.11 (t, *J* = 8.5 Hz, 2H), 3.28 – 3.13 (m, 4H), 2.95 – 2.82 (m, 2H).

13C NMR (101 MHz, $CDCl_3$) δ 167.9, 142.7, 131.0, 127.6, 124.6, 123.9, 116.9, 99.2, 49.8, 47.9, 33.0, 28.0.

HR-MS (ESI-TOF) m/z: $[M+Na]^+$ calcd. for $C_{12}H_{13}Cl_3NO$ 292.0057, found 292.0061.

4,4,4-Trichloro-*N*-benzylbutanamide (**3r**)

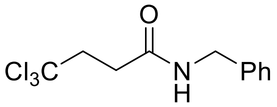

43.3 mg, light yellow oil, yield: 39%. Eluent: pentane/ethyl acetate/dichloromethane = 10/1/1 to 5/1/1.

1H NMR (400 MHz, $CDCl_3$) δ 7.34 – 7.24 (m, 3H), 7.24 – 7.18 (m, 2H), 6.64 (t, *J* = 5.8 Hz, 1H), 4.35 (d, *J* = 5.7 Hz, 2H), 3.10 – 2.96 (m, 2H), 2.73 – 2.58 (m, 2H).

13C NMR (101 MHz, $CDCl_3$) δ 169.9, 137.8, 128.6, 127.6, 127.5, 99.0, 50.2, 43.6, 33.0.

HR-MS (ESI-TOF) m/z: $[M+H]^+$ calcd. for $C_{11}H_{13}Cl_3NO$ 280.0057, found 280.0059.

4,4,4-Trichlorobutanamide (**3s**)

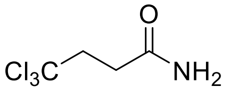

53.4 mg, colorless solid, yield: 70%, 95% purity. Eluent: pentane/ethyl acetate/dichloromethane = 1/1/1 to 1/2/1.

1H NMR (400 MHz, $CDCl_3$) δ 6.45 (s, 1H), 6.17 (s, 1H), 3.10 – 2.91 (m, 2H), 2.77 – 2.60 (m, 2H).

13C NMR (101 MHz, $CDCl_3$) δ 173.0, 98.8, 50.0, 32.4.

HR-MS (ESI-TOF) m/z: $[M+H]^+$ calcd. for $C_4H_7Cl_3NO$ 189.9588, found 189.9592.

4,4,4-Trichlorophenylbutanoate (**4a**)

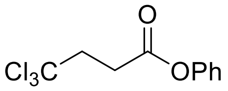

83.8 mg, light yellow oil, yield: 78%. Eluent: pentane/ethyl acetate = 30/1 to 20/1.

1H NMR (400 MHz, $CDCl_3$) δ 7.45 – 7.32 (m, 2H), 7.30 – 7.18 (m, 1H), 7.14 – 7.04 (m, 2H), 3.23 – 3.12 (m, 2H), 3.12 – 3.03 (m, 2H).

13C NMR (101 MHz, $CDCl_3$) δ 169.5, 150.4, 129.5, 126.0, 121.3, 98.3, 49.7, 31.6.

HR-MS (ESI-TOF) m/z: $[M+H]^+$ calcd. for $C_{10}H_{10}Cl_3O_2$ 266.9741, found 266.9739.

4,4,4-Trichloro-2-isopropyl-4-methylphenylbutanoate (**4b**)

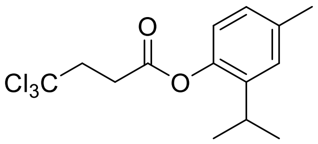

96.1 mg, light yellow oil, yield: 74%. Eluent: pentane/ethyl acetate = 50/1 to 30/1.

^{1}H NMR (400 MHz, $CDCl_3$) δ 7.24 (d, *J* = 7.9 Hz, 1H), 7.08 (d, *J* = 8.0 Hz, 1H), 6.89 – 6.81 (m, 1H), 3.27 – 3.18 (m, 2H), 3.16 – 3.08 (m, 2H), 3.00 (p, *J* = 6.9 Hz, 1H), 2.35 (s, 3H), 1.24 (d, *J* = 7.0 Hz, 6H).

^{13}C NMR (101 MHz, $CDCl_3$) δ 169.8, 147.6, 136.8, 136.6, 127.4, 126.5, 122.4, 98.4, 49.7, 31.4, 27.1, 23.0, 20.8.

HR-MS (ESI-TOF) m/z: $[M+H]^+$ calcd. for $C_{14}H_{18}Cl_3O_2$ 323.0367, found 323.0369.

4,4,4-trichloro-4-acetamidophenylbutanoate (**4c**)

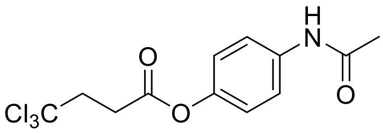

55.6 mg, light yellow solid, yield: 43%. Eluent: pentane/ethyl acetate/dichloromethane = 4/1/1 to 2/1/1.

^{1}H NMR (400 MHz, $CDCl_3$) δ 8.07 (s, 1H), 7.48 (d, *J* = 8.9 Hz, 2H), 6.99 (d, *J* = 8.9 Hz, 2H), 3.24 – 3.11 (m, 2H), 3.10 – 3.00 (m, 2H), 2.11 (s, 3H).

^{13}C NMR (101 MHz, $CDCl_3$) δ 169.9, 168.8, 146.4, 135.9, 121.6, 121.0, 98.3, 49.6, 31.5, 24.2.

HR-MS (ESI-TOF) m/z: $[M+H]^+$ calcd. for $C_{12}H_{13}Cl_3NO_3$ 323.9956, found 323.9962.

4,4,4-trichloro-4-carbamoylphenylbutanoate (**4d**)

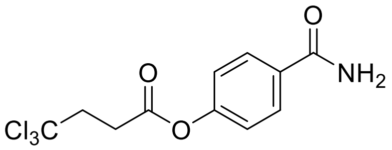

107.5 mg, light yellow solid, yield: 87%. Eluent: Eluent: pentane/ethyl acetate/dichloromethane = 2/1/1 to 1/1/1.

^{1}H NMR (400 MHz, DMSO-d_6) δ 8.02 (s, 1H), 7.95 (d, *J* = 8.7 Hz, 2H), 7.41 (s, 1H), 7.25 (d, *J* = 8.6 Hz, 2H), 3.25 – 3.16 (m, 2H), 3.11 – 3.00 (m, 2H).

^{13}C NMR (101 MHz, DMSO-d_6) δ 169.3, 167.2, 152.5, 132.1, 129.0, 121.5, 99.0, 49.1, 31.2.

HR-MS (ESI-TOF) m/z: $[M+H]^+$ calcd. for $C_{11}H_{11}Cl_3NO_3$ 309.9799, found 309.9805.

4,4,4-trichloro-4-formylphenylbutanoate (**4e**)

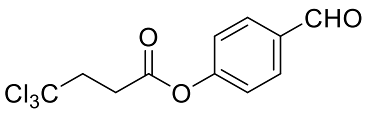

89.5 mg, light yellow oil, yield: 76%. Eluent: pentane/ethyl acetate = 20/1 to 10/1.

^{1}H NMR (400 MHz, $CDCl_3$) δ 9.99 (s, 1H), 8.01 – 7.83 (m, 2H), 7.35 – 7.24 (m, 2H), 3.22 – 3.15 (m, 2H), 3.14 – 3.08 (m, 2H).

^{13}C NMR (101 MHz, $CDCl_3$) δ 190.7, 168.9, 154.9, 134.1, 131.2, 122.1, 98.1, 49.4, 31.5.

HR-MS (ESI-TOF) m/z: $[M+H]^+$ calcd. for $C_{11}H_{10}Cl_3O_3$ 294.9690, found 294.9695.

4,4,4-trichloro-4-nitrophenylbutanoate (**4f**)

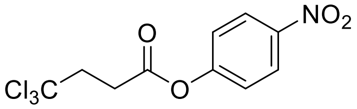

31.7 mg, light yellow oil, yield: 25%, 95% purity. Eluent: pentane/ethyl acetate = 15/1 to 10/1.

^{1}H NMR (400 MHz, $CDCl_3$) δ 8.32 – 8.24 (m, 2H), 7.35 – 7.27 (m, 2H), 3.22 – 3.16 (m, 2H), 3.16 – 3.09 (m, 2H).

^{13}C NMR (101 MHz, $CDCl_3$) δ 168.8, 155.0, 145.5, 125.3, 122.3, 98.0, 49.4, 31.5.

HR-MS (ESI-TOF) m/z: $[M+H]^+$ calcd. for $C_{10}H_9Cl_3NO_4$ 311.9592, found 311.9598.

[1,1'-Biphenyl]-2,2'-diyl bis(4,4,4-trichlorobutanoate) (**4g**)

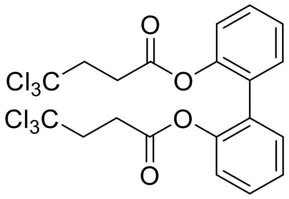

49.4 mg, light yellow oil, yield: 46% (0.2 mmol scale), 95% purity. Eluent: pentane/ethyl acetate = 20/1 to 10/1.

^{1}H NMR (400 MHz, $CDCl_3$) δ 7.48 – 7.40 (m, 2H), 7.37 – 7.31 (m, 4H), 7.19 (d, *J* = 8.2 Hz, 2H), 2.88 – 2.82 (m, 4H), 2.81 – 2.74 (m, 4H).

^{13}C NMR (101 MHz, $CDCl_3$) δ 169.3, 147.9, 131.3, 130.2, 129.3, 126.3, 122.3, 98.3, 49.4, 31.3.

HR-MS (ESI-TOF) m/z: $[M+H]^+$ calcd. for $C_{20}H_{17}Cl_6O_4$ 530.9253, found 530.9249.

Propane-2,2-diylbis(4,1-phenylene) bis(4,4,4-trichlorobutanoate) (**4h**)

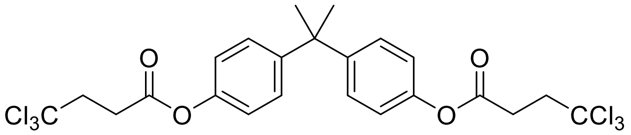

66.3 mg, colorless solid, yield: 58% (0.2 mmol scale). Eluent: pentane/ethyl acetate = 20/1 to 10/1.

^{1}H NMR (400 MHz, $CDCl_3$) δ 7.27 – 7.19 (m, 4H), 7.05 – 6.94 (m, 4H), 3.21 – 3.12 (m, 4H), 3.10 – 3.01 (m, 4H), 1.67 (s, 6H).

^{13}C NMR (101 MHz, $CDCl_3$) δ 169.6, 148.3, 148.0, 127.8, 120.7, 98.4, 49.7, 42.5, 31.6, 30.9.

HR-MS (ESI-TOF) m/z: $[M+H]^+$ calcd. for $C_{23}H_{23}Cl_6O_4$ 572.9722, found 572.9724.

4-((4,4,4-Trichlorobutanoyl)oxy)benzyl 4,4,4-trichlorobutanoate (**4i**)

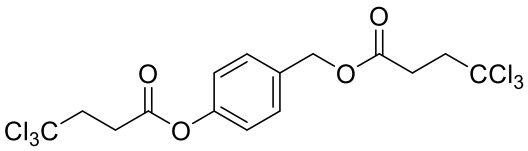

50.8 mg, light yellow oil, yield: 54% (0.2 mmol scale). Eluent: pentane/ethyl acetate = 20/1 to 10/1.

^{1}H NMR (400 MHz, $CDCl_3$) δ 7.43 – 7.37 (m, 2H), 7.15 – 7.09 (m, 2H), 5.15 (s, 2H), 3.21 – 3.15 (m, 2H), 3.11 – 3.05 (m, 4H), 2.89 – 2.83 (m, 2H).

^{13}C NMR (101 MHz, $CDCl_3$) δ 170.7, 169.5, 150.4, 133.3, 129.7, 121.6, 98.4, 98.3, 66.1, 49.7, 49.6, 31.5, 31.4.

HR-MS (ESI-TOF) m/z: $[M+NH_4]^+$ calcd. for $C_{15}H_{18}Cl_6NO_4$ 485.9362, found 485.9359.

4,4,4-trichloro-4-(hydroxymethyl)phenylbutanoate (**4j**)

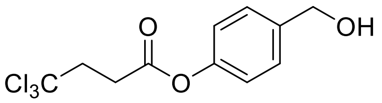

57.1 mg, light yellow oil, yield: 48% (and isolated **4i**, 30.1 mg, 16%). Eluent: pentane/ethyl acetate = 20/1 to 3/1.

^{1}H NMR (400 MHz, $CDCl_3$) δ 7.35 (d, *J* = 8.3 Hz, 2H), 7.07 (d, *J* = 8.4 Hz, 2H), 4.62 (s, 2H), 3.21 – 3.12 (m, 2H), 3.10 – 3.03 (m, 2H), 2.37 (s, 1H).

^{13}C NMR (101 MHz, $CDCl_3$) δ 169.7, 149.6, 138.8, 128.0, 121.3, 98.3, 64.4, 49.6, 31.5.

HR-MS (ESI-TOF) m/z: $[M+H]^+$ calcd. for $C_{11}H_{12}Cl_3O_3$ 296.9847, found 296.9837.

4,4,4-Trichloro-3-phenylpropylbutanoate (**4k**)

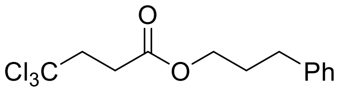

80.5 mg, light yellow oil, yield: 65%. Eluent: pentane/ethyl acetate = 30/1 to 20/1.

^{1}H NMR (400 MHz, $CDCl_3$) δ 7.34 – 7.28 (m, 2H), 7.25 – 7.17 (m, 3H), 4.17 (t, *J* = 6.5 Hz, 2H), 3.12 – 3.02 (m, 2H), 2.73 (t, *J* = 7.7 Hz, 2H), 2.76 – 2.69 (m, 2H), 2.07 – 1.95 (m, 2H).

^{13}C NMR (101 MHz, $CDCl_3$) δ 170.9, 140.9, 128.4, 128.3, 126.0, 98.6, 64.4, 49.8, 32.1, 31.4, 30.0.

HR-MS (ESI-TOF) m/z: $[M+ NH_4]^+$ calcd. for $C_{13}H_{19}Cl_3NO_2$ 326.0476, found 326.0470.

4,4,4-Trichloro-4-phenylbutylbutanoate (**4l**)

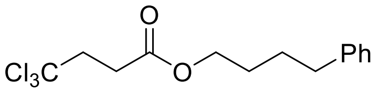

85.5 mg, light yellow oil, yield: 66%. Eluent: pentane/ethyl acetate = 30/1 to 20/1.

^{1}H NMR (400 MHz, $CDCl_3$) δ 7.35 – 7.27 (m, 2H), 7.25 – 7.13 (m, 3H), 4.21 – 4.09 (m, 2H), 3.13 – 3.03 (m, 2H), 2.85 – 2.78 (m, 2H), 2.71 – 2.61 (m, 2H), 1.77 – 1.65 (m, 4H).

^{13}C NMR (101 MHz, $CDCl_3$) δ 171.0, 141.8, 128.3, 125.8, 98.6, 64.9, 49.8, 35.4, 31.4, 28.1, 27.6.

HR-MS (ESI-TOF) m/z: $[M+H]^+$ calcd. for $C_{14}H_{18}Cl_3O_2$ 323.0367, found 323.0366.

4,4,4-Trichloro-decylbutanoate (**4m**)

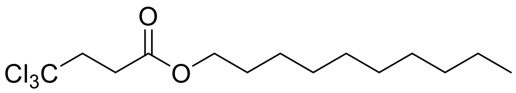

90.7 mg, light yellow oil, yield: 68%. Eluent: pentane/ethyl acetate = 30/1 to 20/1.

^{1}H NMR (400 MHz, $CDCl_3$) δ 4.10 (t, *J* = 6.8 Hz, 2H), 3.10 – 2.99 (m, 2H), 2.83 – 2.74 (m, 2H), 1.68 – 1.58 (m, 2H), 1.37 – 1.23 (m, 14H), 0.87 (t, *J* = 6.8 Hz, 3H).

^{13}C NMR (101 MHz, $CDCl_3$) δ 171.0, 98.6, 65.2, 49.9, 31.8, 31.5, 29.48, 29.46, 29.3, 29.2, 28.5, 25.8, 22.6, 14.1.

HR-MS (ESI-TOF) m/z: $[M+H]^+$ calcd. for $C_{14}H_{26}Cl_3O_2$ 331.0993, found 331.0997.

4,4,4-Trichloro-6-bromohexylbutanoate (**4n**)

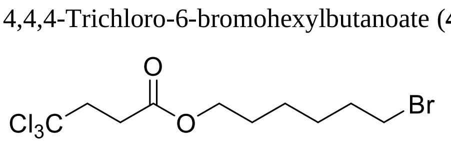

87.7 mg, light yellow oil, yield: 62%. Eluent: pentane/ethyl acetate = 30/1 to 20/1.

^{1}H NMR (400 MHz, $CDCl_3$) δ 4.10 (t, *J* = 6.7 Hz, 2H), 3.39 (t, *J* = 6.7 Hz, 2H), 3.08 – 2.99 (m, 2H), 2.82 – 2.73 (m, 2H), 1.85 (p, *J* = 6.9 Hz, 2H), 1.65 (p, *J* = 6.9 Hz, 2H), 1.52 – 1.42 (m, 2H), 1.42 – 1.32 (m, 2H).

^{13}C NMR (101 MHz, $CDCl_3$) δ 171.0, 98.5, 64.9, 49.8, 33.6, 32.5, 31.4, 28.3, 27.7, 25.0.

HR-MS (ESI-TOF) m/z: $[M+H]^+$ calcd. for $C_{10}H_{17}BrCl_3O_2$ 352.9472, found 352.9475.

4,4,4-Trichloro-benzylbutanoate (**4o**)

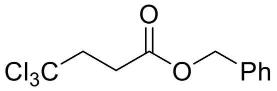

75.2 mg, light yellow oil, yield: 67%. Eluent: pentane/ethyl acetate = 30/1 to 20/1.

^{1}H NMR (400 MHz, $CDCl_3$) δ 7.45 – 7.30 (m, 5H), 5.18 (s, 2H), 3.18 – 3.05 (m, 2H), 2.95 – 2.79 (m, 2H).

^{13}C NMR (101 MHz, $CDCl_3$) δ 170.8, 135.4, 128.6, 128.4, 128.3, 98.5, 66.8, 49.8, 31.4.

HR-MS (ESI-TOF) m/z: $[M+H]^+$ calcd. for $C_{11}H_{12}Cl_3O_2$ 280.9897, found 280.9896.

Thiophen-2-ylmethyl 4,4,4-trichlorobutanoate (**4p**)

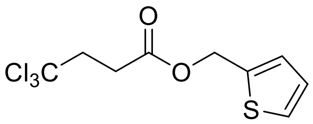

44.6 mg, light yellow oil, yield: 39%. Eluent: pentane/ethyl acetate = 30/1 to 20/1.

^{1}H NMR (400 MHz, $CDCl_3$) δ 7.34 (dd, *J* = 5.1, 1.2 Hz, 1H), 7.12 (d, *J* = 3.6 Hz, 1H), 7.00 (dd, *J* = 5.1, 3.5 Hz, 1H), 5.32 (s, 2H), 3.11 – 3.04 (m, 2H), 2.88 – 2.81 (m, 2H).

^{13}C NMR (101 MHz, $CDCl_3$) δ 170.7, 137.3, 128.5, 127.1, 126.9, 98.4, 61.0, 49.7, 31.4.

HR-MS (ESI-TOF) m/z: $[M+H]^+$ calcd. for $C_9H_{10}Cl_3O_2S$ 286.9462, found 286.9457.

Pyridin-3-ylmethyl 4,4,4-trichlorobutanoate (**4q**)

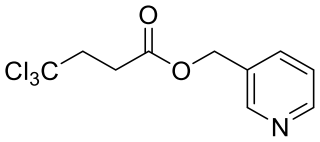

100.7 mg, light yellow oil, yield: 89%. Eluent: pentane/ethyl acetate/dichloromethane = 5/1/1 to 2/1/1.

^{1}H NMR (400 MHz, $CDCl_3$) δ 8.64 (d, *J* = 2.3 Hz, 1H), 8.60 (dd, *J* = 4.9, 1.7 Hz, 1H), 7.71 (dt, *J* = 7.8, 2.0 Hz, 1H), 7.32 (dd, *J* = 7.8, 4.9 Hz, 1H), 5.19 (s, 2H), 3.15 – 3.00 (m, 2H), 2.93 – 2.81 (m, 2H).

^{13}C NMR (101 MHz, $CDCl_3$) δ 170.6, 149.7, 149.6, 136.0, 131.0, 123.3, 98.3, 64.1, 49.6, 31.2.

HR-MS (ESI-TOF) m/z: $[M+H]^+$ calcd. for $C_{10}H_{11}Cl_3NO_2$ 281.9850, found 281.9857.

(*E*)-Hex-2-en-1-yl 4,4,4-trichlorobutanoate (**4r**)

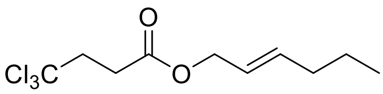

64.8 mg, light yellow oil, yield: 59%. Eluent: pentane/ethyl acetate = 30/1 to 20/1.

^{1}H NMR (400 MHz, $CDCl_3$) δ 5.85 – 5.69 (m, 1H), 5.63 – 5.48 (m, 1H), 4.62 – 4.42 (m, 2H), 3.10 – 2.98 (m, 2H), 2.88 – 2.73 (m, 2H), 2.11 – 1.97 (m, 2H), 1.40 (h, *J* = 7.4 Hz, 2H), 0.89 (t, *J* = 7.4 Hz, 3H).

^{13}C NMR (101 MHz, $CDCl_3$) δ 170.8, 137.0, 123.4, 98.6, 65.9, 49.9, 34.2, 31.5, 22.0, 13.6.

HR-MS (ESI-TOF) m/z: $[M+H]^+$ calcd. for $C_{10}H_{16}Cl_3O_2$ 273.0210, found 273.0216.

But-2-yn-1-yl 4,4,4-trichlorobutanoate (**4s**)

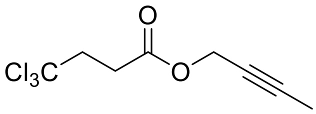

64.1 mg, colorless oil, yield: 66%. Eluent: pentane/ethyl acetate = 30/1 to 20/1.

^{1}H NMR (400 MHz, $CDCl_3$) δ 4.68 (q, *J* = 2.4 Hz, 2H), 3.10 – 3.01 (m, 2H), 2.87 – 2.79 (m, 2H), 1.85 (t, *J* = 2.4 Hz, 3H).

^{13}C NMR (101 MHz, $CDCl_3$) δ 170.4, 98.4, 83.6, 72.7, 53.3, 49.7, 31.3, 3.6.

HR-MS (ESI-TOF) m/z: $[M+H]^+$ calcd. for $C_8H_{10}Cl_3O_2$ 242.9741, found 242.9749.

1,2-Phenylenebis(methylene) bis(4,4,4-trichlorobutanoate) (**4t**)

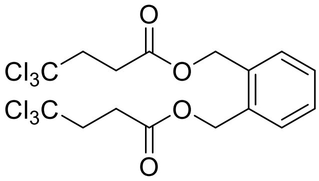

21.9 mg, light yellow oil, yield: 23% (0.2 mmol scale). Eluent: pentane/ethyl acetate = 20/1 to 5/2.

^{1}H NMR (400 MHz, $CDCl_3$) δ 7.46 – 7.41 (m, 2H), 7.41 – 7.35 (m, 2H), 5.27 (s, 4H), 3.12 – 3.02 (m, 4H), 2.91 – 2.80 (m, 4H).

^{13}C NMR (101 MHz, $CDCl_3$) δ 170.7, 134.1, 130.1, 129.0, 98.4, 64.3, 49.7, 31.4.

HR-MS (ESI-TOF) m/z: $[M+NH_4]^+$ calcd. for $C_{16}H_{20}Cl_6NO_4$ 499.9518, found 499.9524, $[M+Na]^+$ calcd. for $C_{16}H_{16}Cl_6NaO_4$ 504.9072, found 504.9071.

4,4,4-Trichloro-2-(hydroxymethyl)benzyl butanoate (**4u**)

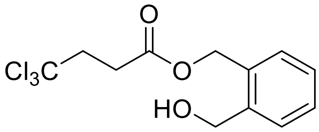

15.0 mg, light yellow oil, yield: 24% (0.2 mmol scale). Eluent: pentane/ethyl acetate = 20/1 to 5/2.

^{1}H NMR (400 MHz, $CDCl_3$) δ 7.46 – 7.39 (m, 2H), 7.38 – 7.31 (m, 2H), 5.30 (s, 2H), 4.77 (s, 2H), 3.11 – 3.02 (m, 2H), 2.90 – 2.80 (m, 2H), 2.06 (s, 1H).

^{13}C NMR (101 MHz, $CDCl_3$) δ 170.9, 139.2, 133.4, 129.9, 129.1, 128.8, 128.2, 98.4, 64.5, 62.9, 49.7, 31.5.

HR-MS (ESI-TOF) m/z: $[M+H]^+$ calcd. for $C_{12}H_{14}Cl_3O_3$ 311.0003, found 311.0002, $[M+Na]^+$ calcd. for $C_{12}H_{13}Cl_3NaO_3$ 332.9822, found 332.9827.

Ethane-1,2-diyl bis(4,4,4-trichlorobutanoate) (**4v**)

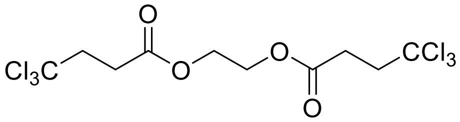

56.1 mg, light yellow oil, yield: 69% (0.2 mmol scale). Eluent: pentane/ethyl acetate = 10/1 to 8/1.

^{1}H NMR (400 MHz, $CDCl_3$) δ 4.34 (s, 4H), 3.13 – 2.99 (m, 4H), 2.89 – 2.74 (m, 4H).

^{13}C NMR (101 MHz, $CDCl_3$) δ 170.8, 98.4, 62.5, 49.7, 31.3.

HR-MS (ESI-TOF) m/z: [M+ NH_4]$^+$ calcd. for $C_{10}H_{16}Cl_6NO_4$ 423.9205, found 423.9213, [M+Na]$^+$ calcd. for $C_{10}H_{12}Cl_6NaO_4$ 428.8759, found 428.8763.

Propane-1,2,3-triyl tris(4,4,4-trichlorobutanoate) (**4w**)

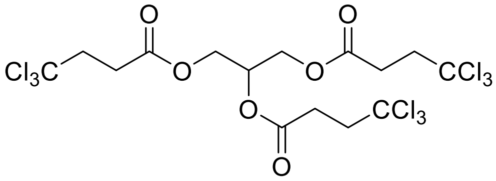

24.5 mg, light yellow oil, yield: 20% (0.2 mmol scale). Eluent: pentane/ethyl acetate = 20:1 to 10:1.

^{1}H NMR (400 MHz, $CDCl_3$) δ 5.40 – 5.27 (m, 1H), 4.41 (dd, *J* = 12.0, 4.1 Hz, 2H), 4.23 (dd, *J* = 12.1, 5.9 Hz, 2H), 3.14 – 3.00 (m, 6H), 2.90 – 2.76 (m, 6H).

^{13}C NMR (101 MHz, $CDCl_3$) δ 170.6, 170.3, 98.3, 69.5, 62.6, 49.6, 31.3, 31.2.

HR-MS (ESI-TOF) m/z: [M+ NH_4]$^+$ calcd. for $C_{15}H_{21}Cl_9NO_6$ 625.8560, found 625.8561.

4,4,4-Trichloro-*N*-(4-(3-ethyl-2,6-dioxopiperidin-3-yl)phenyl)butanamide (**5a**)

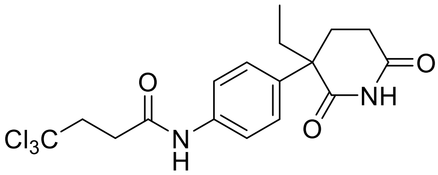

66.1 mg, colorless solid, yield: 81% (0.2 mmol scale). Eluent: pentane/ethyl acetate/dichloromethane = 4/1/1 to 2/1/1.

^{1}H NMR (400 MHz, DMSO-*d*$_6$) δ 10.83 (s, 1H), 10.19 (s, 1H), 7.59 (d, *J* = 8.3 Hz, 2H), 7.22 (d, *J* = 8.3 Hz, 2H), 3.11 (dd, *J* = 8.9, 6.0 Hz, 2H), 2.80 (dd, *J* = 9.0, 6.0 Hz, 2H), 2.51 – 2.41 (m, 1H), 2.36 – 2.25 (m, 1H), 2.21 – 2.08 (m, 2H), 1.92 – 1.71 (m, 2H), 0.74 (t, *J* = 7.3 Hz, 3H).

^{13}C NMR (101 MHz, DMSO-*d*$_6$) δ 175.8, 172.8, 168.2, 137.9, 134.3, 126.7, 119.3, 99.8, 49.8, 49.6, 33.3, 32.2, 29.1, 26.0, 8.9.

HR-MS (ESI-TOF) m/z: [M+H]$^+$ calcd. for $C_{17}H_{20}Cl_3N_2O_3$ 405.0534, found 405.0536.

(1*R*,2*R*,5*R*)-5-Isopropyl-2-methylcyclohexyl 4-(4,4,4-trichlorobutanamido)benzoate (**5b**)

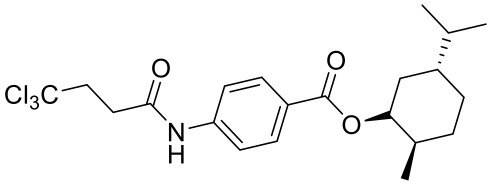

148.7 mg, light yellow solid, yield: 82%. Eluent: pentane/ethyl acetate/dichloromethane = 10/1/1 to 7/1/1.

^{1}H NMR (400 MHz, $CDCl_3$) δ 8.60 (s, 1H), 7.98 (d, *J* = 8.5 Hz, 2H), 7.63 (d, *J* = 8.4 Hz, 2H), 4.89 (td, *J* = 10.8, 4.3 Hz, 1H), 3.20 – 3.08 (m, 2H), 2.94 – 2.81 (m, 2H), 2.13 – 2.03 (m, 1H), 1.97 – 1.86 (m, 1H), 1.77 – 1.64 (m, 2H), 1.59 – 1.44 (m, 2H), 1.16 – 1.03 (m, 2H), 0.97 – 0.84 (m, 1H), 0.89 (d, *J* = 6.8 Hz, 6H), 0.76 (d, *J* = 6.9 Hz, 3H).

^{13}C NMR (101 MHz, $CDCl_3$) δ 168.8, 165.9, 141.9, 130.7, 126.2, 119.1, 98.7, 75.0, 49.9, 47.1, 40.9, 34.2, 31.4, 26.4, 23.6, 21.9, 20.6, 16.5.

HR-MS (ESI-TOF) m/z: [M+H]$^+$ calcd. for $C_{21}H_{29}Cl_3NO_3$ 448.1208, found 448.1213.

4-(4,4,4-Trichlorobutanamido)phenyl ((1*S*,4*R*)-7,7-dimethyl-2-oxobicyclo[2.2.1]heptan-1-yl)methanesulfonate (**5c**)

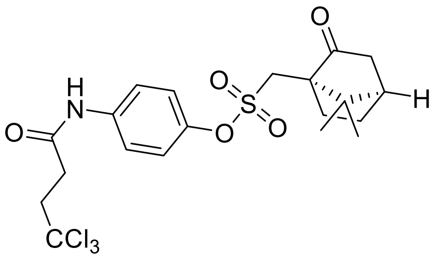

84.3 mg, light yellow oil, yield: 85% (0.2 mmol scale). Eluent: pentane/ethyl acetate/dichloromethane = 5/1/1 to 3/1/1.

^{1}H NMR (400 MHz, $CDCl_3$) δ 8.23 (s, 1H), 7.48 (d, *J* = 9.0 Hz, 2H), 7.16 (d, *J* = 8.9 Hz, 2H), 3.76 (d, *J* = 14.9 Hz, 1H), 3.19 (d, *J* = 15.0 Hz, 1H), 3.15 – 3.05 (m, 2H), 2.87 – 2.75 (m, 2H), 2.53 – 2.35 (m, 2H), 2.14 (t, *J* = 4.5 Hz, 1H), 2.11 – 2.04 (m, 1H), 1.95 (d, *J* = 18.6 Hz, 1H), 1.78 – 1.68 (m, 1H), 1.49 – 1.40 (m, 1H), 1.11 (s, 3H), 0.88 (s, 3H).

^{13}C NMR (101 MHz, $CDCl_3$) δ 214.3, 168.5, 144.7, 136.8, 122.5, 121.2, 98.9, 58.0, 49.9, 48.0, 47.5, 42.7, 42.4, 33.8, 26.7, 25.1, 19.7, 19.6.

HR-MS (ESI-TOF) m/z: $[M+H]^+$ calcd. for $C_{20}H_{25}Cl_3NO_5S$ 496.0514, found 496.0520.

4,4,4-Trichloro-*N*-(3-methoxypyrazin-2-yl)-*N*-((4-(4,4,4-trichlorobutanamido)phenyl)sulfonyl)butanamide (**5d**)

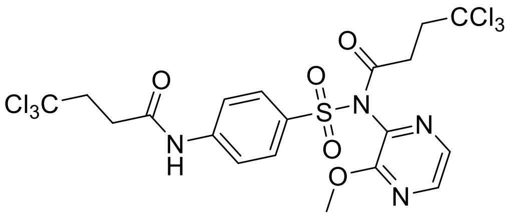

49.2 mg, light yellow solid, yield: 39% (0.2 mmol scale). Eluent: pentane/ethyl acetate/dichloromethane = 5/1/1 to 3/1/1.

^{1}H NMR (400 MHz, DMSO-d_6) δ 8.53 (d, *J* = 2.6 Hz, 1H), 8.39 (d, *J* = 2.7 Hz, 1H), 8.07 – 7.96 (m, 2H), 7.93 – 7.81 (m, 2H), 4.04 (s, 3H), 3.21 – 3.08 (m, 2H), 2.96 (t, *J* = 6.9 Hz, 2H), 2.92 – 2.87 (m, 2H), 2.42 (t, *J* = 7.0 Hz, 2H).

^{13}C NMR (101 MHz, DMSO-d_6) δ 169.2, 168.6, 157.6, 144.2, 144.1, 136.5, 134.0, 131.5, 130.4, 118.3, 99.6, 98.6, 54.6, 49.3, 47.9, 33.5, 32.5.

HR-MS (ESI-TOF) m/z: $[M+H]^+$ calcd. for $C_{19}H_{19}Cl_6N_4O_5S$ 624.9202, found 624.9208.

4,4,4-Trichloro-4-(3-oxobutyl)phenyl butanoate (**5e**)

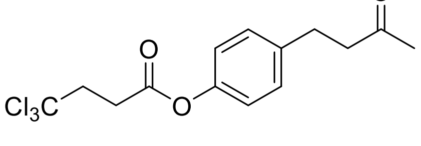

109.6 mg, light yellow oil, yield: 81%. Eluent: pentane/ethyl acetate = 20:1 to 10:1.

^{1}H NMR (400 MHz, $CDCl_3$) δ 7.22 – 7.13 (m, 2H), 7.04 – 6.94 (m, 2H), 3.19 – 3.10 (m, 2H), 3.08 – 2.99 (m, 2H), 2.86 (t, *J* = 7.5 Hz, 2H), 2.73 (t, *J* = 7.2 Hz, 2H), 2.11 (s, 3H).

^{13}C NMR (101 MHz, $CDCl_3$) δ 207.3, 169.5, 148.5, 138.7, 129.2, 121.1, 98.3, 49.5, 44.8, 31.4, 29.9, 28.8.

HR-MS (ESI-TOF) m/z: $[M+H]^+$ calcd. for $C_{14}H_{16}Cl_3O_3$ 337.0160, found 337.0156.

(8*R*,9*S*,13*S*,14*S*)-13-Methyl-17-oxo-7,8,9,11,12,13,14,15,16,17-decahydro-6*H*-cyclopenta[*a*]phenanthren-3-yl 4,4,4-trichlorobutanoate (**5f**)

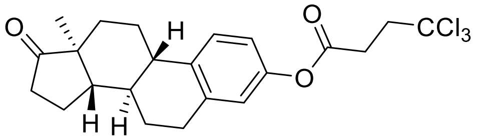

47.6 mg, colorless oil, yield: 78%. Eluent: pentane/ethyl acetate = 20:1 to 15:1.

^{1}H NMR (400 MHz, $CDCl_3$) δ 7.29 (d, *J* = 8.5 Hz, 1H), 6.86 (dd, *J* = 8.5, 2.6 Hz, 1H), 6.82 (d, *J* = 2.5 Hz, 1H), 3.21 – 3.14 (m, 2H), 3.09 – 3.02 (m, 2H), 2.95 – 2.87 (m, 2H), 2.50 (dd, *J* = 18.7, 8.7 Hz, 1H), 2.44 – 2.36 (m, 1H), 2.33 – 2.23 (m, 1H), 2.19 – 1.92 (m, 4H), 1.69 – 1.41 (m, 6H), 0.90 (s, 3H).

^{13}C NMR (101 MHz, $CDCl_3$) δ 220.5, 169.7, 148.2, 138.0, 137.5, 126.3, 121.2, 118.4, 98.3, 50.3, 49.6, 47.8, 44.0, 37.8, 35.7, 31.5, 31.4, 29.3, 26.2, 25.6, 21.4, 13.7.

HR-MS (ESI-TOF) m/z: $[M+H]^+$ calcd. for $C_{22}H_{26}Cl_3O_3$ 443.0942, found 443.0941.

3-Oxo-3*H*-spiro[isobenzofuran-1,9'-xanthene]-3',6'-diyl bis(4,4,4-trichlorobutanoate) (**5g**)

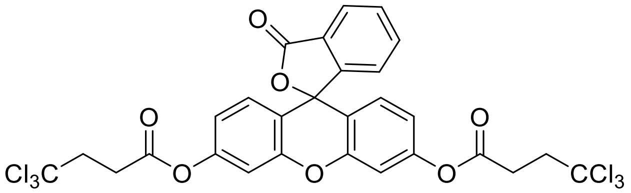

108.3 mg, light yellow solid, yield: 80% (0.2 mmol scale). Eluent: pentane/ethyl acetate/dichloromethane = 10/1/1 to 6/1/1.

^{1}H NMR (400 MHz, $CDCl_3$) δ 8.03 (dd, *J* = 7.1, 1.4 Hz, 1H), 7.73 – 7.59 (m, 2H), 7.18 (dd, *J* = 7.4, 1.2 Hz, 1H), 7.13 – 7.07 (m, 2H), 6.87 – 6.80 (m, 4H), 3.20 – 3.13 (m, 4H), 3.11 – 3.04 (m, 4H).

^{13}C NMR (101 MHz, $CDCl_3$) δ 169.0, 168.9, 152.6, 151.6, 151.4, 135.3, 130.1, 129.0, 125.9, 125.2, 123.9, 117.5, 116.6, 110.2, 98.2, 81.3, 49.4, 31.5.

HR-MS (ESI-TOF) m/z: $[M+H]^+$ calcd. for $C_{28}H_{19}Cl_6O_7$ 676.9256, found 676.9252.

((3a*S*,5a*R*,8a*R*,8b*S*)-2,2,7,7-Tetramethyltetrahydro-3a*H*-bis([1,3]dioxolo)[4,5-b:4',5'-d]pyran-3a-yl)methyl 4,4,4-trichlorobutanoate (**5h**)

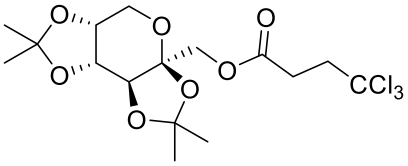

100.5 mg, light yellow oil, yield: 58%. Eluent: pentane/ethyl acetate = 10/1.

^{1}H NMR (400 MHz, $CDCl_3$) δ 4.58 (dd, *J* = 7.9, 2.6 Hz, 1H), 4.43 (d, *J* = 11.6 Hz, 1H), 4.27 (d, *J* = 2.6 Hz, 1H), 4.21 (dd, *J* = 7.9, 1.7 Hz, 1H), 4.04 (d, *J* = 11.6 Hz, 1H), 3.87 (dd, *J* = 13.0, 1.9 Hz, 1H), 3.74 (d, *J* = 13.0 Hz, 1H), 3.10 – 3.00 (m, 2H), 2.88 – 2.80 (m, 2H), 1.51 (s, 3H), 1.45 (s, 3H), 1.38 (s, 3H), 1.31 (s, 3H).

^{13}C NMR (101 MHz, $CDCl_3$) δ 170.3, 109.0, 108.7, 101.3, 98.3, 70.6, 70.5, 69.9, 65.8, 61.2, 49.7, 31.2, 26.4, 25.8, 25.1, 24.0.

HR-MS (ESI-TOF) m/z: $[M+H]^+$ calcd. for $C_{16}H_{24}Cl_3O_7$ 433.0582, found 433.0588.

(1*R*,2*R*,5*R*)-5-Isopropyl-2-methylcyclohexyl 4,4,4-trichlorobutanoate (**5i**)

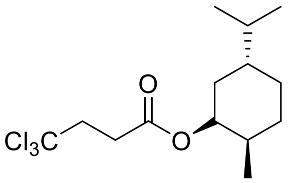

84.1 mg, light yellow oil, yield: 64%. Eluent: pentane/ethyl acetate = 30/1.

^{1}H NMR (400 MHz, $CDCl_3$) δ 4.72 (td, *J* = 10.8, 4.4 Hz, 1H), 3.11 – 2.98 (m, 2H), 2.83 – 2.72 (m, 2H), 2.03 – 1.95 (m, 1H), 1.90 – 1.79 (m, 1H), 1.74 – 1.64 (m, 2H), 1.55 – 1.43 (m, 1H), 1.43 – 1.34 (m, 1H), 1.11 – 1.02 (m, 1H), 1.01 – 0.93 (m, 1H), 0.93 – 0.85 (m, 1H), 0.90 (dd, *J* = 6.8, 1.6 Hz, 6H), 0.76 (d, *J* = 6.9 Hz, 3H).

^{13}C NMR (101 MHz, $CDCl_3$) δ 170.6, 98.7, 75.0, 49.9, 46.9, 40.8, 34.1, 31.7, 31.3, 26.3, 23.4, 22.0, 20.7, 16.3.

HR-MS (ESI-TOF) m/z: $[M+H]^+$ calcd. for $C_{14}H_{27}NCl_3O_2$ 346.1102, found 346.1106; $[M+Na]^+$ calcd. for $C_{14}H_{23}NaCl_3O_2$ 351.0656, found 351.0656.

(3*S*,5*S*,8*R*,9*S*,10*S*,13*S*,14*S*)-10,13-Dimethyl-17-oxohexadecahydro-1*H*-cyclopenta[*a*]phenanthren-3-yl 4,4,4-trichlorobutanoate (**5j**)

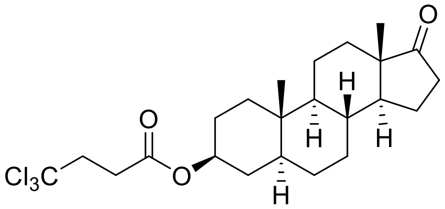

68.2 mg, colorless solid, yield: 74% (0.2 mmol scale). Eluent: pentane/ethyl acetate/dichloromethane = 20/1/1 to 15/1/1.

^{1}H NMR (400 MHz, $CDCl_3$) δ 4.74 (tt, *J* = 10.9, 4.9 Hz, 1H), 3.11 – 2.99 (m, 2H), 2.84 – 2.73 (m, 2H), 2.43 (dd, *J* = 19.2, 8.8 Hz, 1H), 2.07 (dt, *J* = 18.8, 9.0 Hz, 1H), 1.98 – 1.89 (m, 1H), 1.87 – 1.72 (m, 4H), 1.70 – 1.61 (m, 2H), 1.59 – 1.46 (m, 3H), 1.43 – 1.20 (m, 7H), 1.10 – 0.93 (m, 2H), 0.86 (s, 6H), 0.72 (td, *J* = 11.9, 11.3, 3.9 Hz, 1H).

^{13}C NMR (101 MHz, $CDCl_3$) δ 221.0, 170.4, 98.6, 74.2, 54.2, 51.3, 49.9, 47.7, 44.5, 36.6, 35.7, 35.5, 34.9, 33.8, 31.8, 31.4, 30.7, 28.2, 27.3, 21.7, 20.4, 13.7, 12.1.

HR-MS (ESI-TOF) m/z: $[M+H]^+$ calcd. for $C_{23}H_{34}Cl_3O_3$ 463.1568, found 463.1561.

(3*S*,8*S*,9*S*,10*R*,13*S*,14*S*,17*S*)-17-Acetyl-10,13-dimethyl-2,3,4,7,8,9,10,11,12,13,14,15,16,17-tetradecahydro-1*H*-cyclopenta[*a*]phenanthren-3-yl 4,4,4-trichlorobutanoate (**5k**)

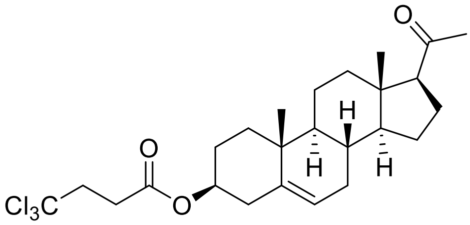

57.3 mg, colorless oil, yield: 58% (0.2 mmol scale). Eluent: pentane/ethyl acetate/dichloromethane = 20/1/1 to 15/1/1.

^{1}H NMR (400 MHz, $CDCl_3$) δ 5.36 (dt, *J* = 3.4, 1.7 Hz, 1H), 4.71 – 4.56 (m, 1H), 3.11 – 2.96 (m, 2H), 2.82 – 2.72 (m, 2H), 2.52 (t, *J* = 8.9 Hz, 1H), 2.32 (d, *J* = 7.9 Hz, 2H), 2.22 – 2.13 (m, 1H), 2.10 (s, 3H), 2.07 – 1.94 (m,

2H), 1.91 – 1.82 (m, 2H), 1.71 – 1.54 (m, 5H), 1.54 – 1.42 (m, 3H), 1.28 – 1.08 (m, 4H), 1.01 (s, 3H), 0.61 (s, 3H).

^{13}C NMR (101 MHz, $CDCl_3$) δ 209.4, 170.4, 139.4, 122.5, 98.6, 74.6, 63.6, 56.8, 49.9, 49.8, 43.9, 38.7, 37.9, 36.9, 36.5, 31.8, 31.76, 31.74, 31.5, 27.6, 24.4, 22.8, 21.0, 19.2, 13.2.

HR-MS (ESI-TOF) m/z: $[M+H]^+$ calcd. for $C_{25}H_{36}Cl_3O_3$ 489.1725, found 489.1732.

(8*R*,9*S*,13*S*,14*S*,17*R*)-13-Methyl-17-((4,4,4-trichlorobutanoyl)oxy)-7,8,9,11,12,13,14,15,16,17-decahydro-6*H*-cyclopenta[*a*]phenanthren-3-yl benzoate (**5l**)

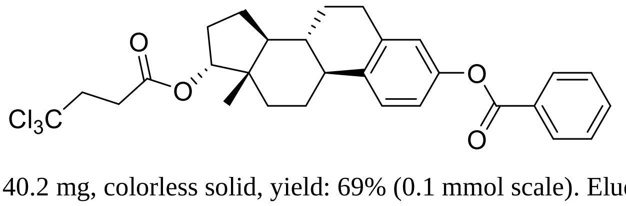

40.2 mg, colorless solid, yield: 69% (0.1 mmol scale). Eluent: pentane/ethyl acetate = 20/1 to 10/1.

^{1}H NMR (400 MHz, $CDCl_3$) δ 8.20 (d, *J* = 7.0 Hz, 2H), 7.63 (t, *J* = 7.6 Hz, 1H), 7.51 (t, *J* = 7.6 Hz, 2H), 7.34 (d, *J* = 8.5 Hz, 1H), 6.98 (dd, *J* = 8.4, 2.6 Hz, 1H), 6.94 (d, *J* = 2.4 Hz, 1H), 4.83 – 4.70 (m, 1H), 3.16 – 3.06 (m, 2H), 2.96 – 2.88 (m, 2H), 2.88 – 2.79 (m, 2H), 2.39 – 2.18 (m, 3H), 1.97 – 1.87 (m, 2H), 1.83 – 1.73 (m, 1H), 1.64 – 1.58 (m, 1H), 1.57 – 1.49 (m, 2H), 1.48 – 1.40 (m, 2H), 1.39 – 1.26 (m, 2H), 0.87 (s, 3H).

^{13}C NMR (101 MHz, $CDCl_3$) δ 171.0, 165.4, 148.7, 138.1, 137.7, 133.4, 130.1, 129.6, 128.5, 126.4, 121.6, 118.7, 98.6, 83.4, 49.9, 49.7, 43.9, 42.9, 38.2, 36.8, 31.6, 29.5, 27.5, 27.0, 26.0, 23.2, 12.1.

HR-MS (ESI-TOF) m/z: $[M+H]^+$ calcd. for $C_{29}H_{32}Cl_3O_4$ 549.1361, found 549.1362.

(*R*)-1-(4-Fluorophenyl)-3-((3*R*,4*S*)-1-(4-fluorophenyl)-2-oxo-4-(4-((4,4,4-trichlorobutanoyl)oxy)phenyl)azetidin-3-yl)propyl 4,4,4-trichlorobutanoate (**5m**)

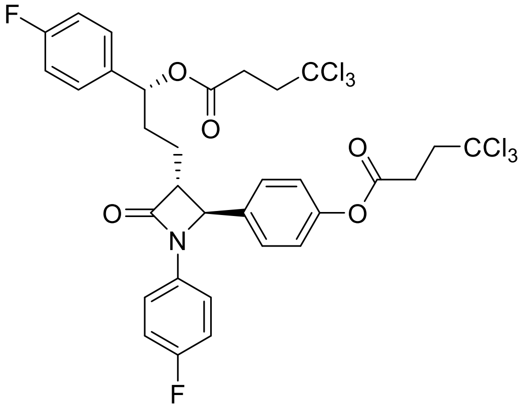

49.5 mg, light yellow oil (0.1 mmol scale), yield: 65%. Eluent: pentane/ethyl acetate/dichloromethane = 10/1/1 to 5/1/1.

^{1}H NMR (400 MHz, $CDCl_3$) δ 7.37 – 7.32 (m, 2H), 7.31 – 7.25 (m, 2H), 7.24 – 7.18 (m, 2H), 7.16 – 7.11 (m, 2H), 7.07 – 7.00 (m, 2H), 6.97 – 6.90 (m, 2H), 5.74 (t, *J* = 6.8 Hz, 1H), 4.61 (d, *J* = 2.4 Hz, 1H), 3.22 – 3.13 (m, 2H), 3.11 – 2.96 (m, 5H), 2.90 – 2.72 (m, 2H), 2.15 – 2.02 (m, 2H), 1.97 – 1.78 (m, 2H).

^{13}C NMR (101 MHz, $CDCl_3$) δ 170.2, 169.5, 166.5, 162.5 (d, *J* = 247.3 Hz), 159.0 (d, *J* = 243.8 Hz), 150.5, 135.2, 135.1 (d, *J* = 3.3 Hz), 133.6 (d, *J* = 2.7 Hz), 128.3 (d, *J* = 8.2 Hz), 127.0, 122.4, 118.3 (d, *J* = 7.9 Hz), 115.9 (d, *J* = 22.7 Hz), 115.7 (d, *J* = 21.6 Hz), 98.4, 98.2, 75.6, 60.6, 60.1, 49.63, 49.60, 33.4, 31.5, 24.9.

^{19}F NMR (376 MHz, $CDCl_3$) δ -113.2, -117.6.

HR-MS (ESI-TOF) m/z: $[M+NH_4]^+$ calcd. for $C_{32}H_{31}Cl_6F_2N_2O_5$ 771.0327, found 771.0321, $[M+Na]^+$ calcd. for $C_{32}H_{27}Cl_6F_2NNaO_5$ 775.9881, found 775.9882.

6,6,6-Trichloro-*N*-phenylhexanamide (**6a**)

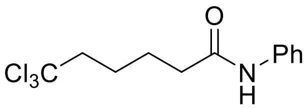

47.1 mg, light yellow solid, yield: 40%. Eluent: pentane/ethyl acetate/dichloromethane = 15/1/1 to 8/1/1.

^{1}H NMR (400 MHz, $CDCl_3$) δ 7.75 (s, 1H), 7.51 (d, *J* = 7.9 Hz, 2H), 7.30 (t, *J* = 7.8 Hz, 2H), 7.10 (t, *J* = 7.4 Hz, 1H), 2.75 – 2.64 (m, 2H), 2.40 (t, *J* = 6.7 Hz, 2H), 1.87 – 1.74 (m, 4H).

^{13}C NMR (101 MHz, $CDCl_3$) δ 170.8, 137.7, 128.9, 124.4, 120.1, 99.7, 54.7, 37.0, 26.0, 24.1.

HR-MS (ESI-TOF) m/z: $[M+H]^+$ calcd. for $C_{12}H_{15}Cl_3NO$ 294.0214, found 294.0221.

6,6,6-Trichloro-*N*-(3-methoxyphenyl)hexanamide (**6b**)

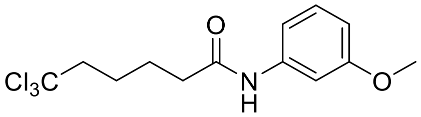

55.8 mg, light yellow oil, yield: 43%. Eluent: pentane/ethyl acetate/dichloromethane = 15/1/1 to 6/1/1.

^{1}H NMR (700 MHz, $CDCl_3$) δ 7.63 (s, 1H), 7.29 (t, *J* = 2.3 Hz, 1H), 7.19 (t, *J* = 8.1 Hz, 1H), 6.98 (d, *J* = 7.6 Hz, 1H), 6.65 (dd, *J* = 8.1, 2.7 Hz, 1H), 3.77 (s, 3H), 2.68 (t, *J* = 7.3 Hz, 2H), 2.40 (t, *J* = 6.8 Hz, 2H), 1.87 – 1.78 (m, 4H).

^{13}C NMR (176 MHz, $CDCl_3$) δ 170.7, 160.1, 139.0, 129.6, 112.0, 110.0, 105.8, 99.7, 55.2, 54.7, 37.1, 26.0, 24.0.

HR-MS (ESI-TOF) m/z: $[M+H]^+$ calcd. for $C_{13}H_{17}Cl_3NO_2$ 324.0319, found 324.0321.

6,6,6-Trichloro-*N*-(3-(trifluoromethyl)phenyl)hexanamide (**6c**)

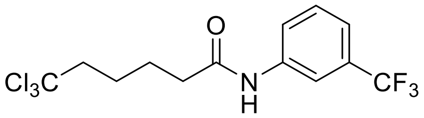

55.1 mg, light yellow oil, yield: 38%. Eluent: pentane/ethyl acetate/dichloromethane = 15/1/1 to 8/1/1.

^{1}H NMR (400 MHz, $CDCl_3$) δ 7.81 (s, 1H), 7.70 (d, *J* = 8.4 Hz, 2H), 7.42 (t, *J* = 7.9 Hz, 1H), 7.35 (d, *J* = 7.9 Hz, 1H), 2.74 – 2.66 (m, 2H), 2.49 – 2.41 (m, 2H), 1.89 – 1.80 (m, 4H).

^{13}C NMR (101 MHz, $CDCl_3$) δ 171.0, 138.2, 131.4 (q, *J* = 32.5 Hz), 129.5, 123.8 (q, *J* = 272.4 Hz), 123.0, 120.9 (q, *J* = 3.6 Hz), 116.6 (q, *J* = 3.7 Hz), 99.6, 54.7, 37.0, 26.0, 23.9.

^{19}F NMR (376 MHz, $CDCl_3$) δ -62.7.

HR-MS (ESI-TOF) m/z: $[M+H]^+$ calcd. for $C_{13}H_{14}Cl_3F_3NO$ 362.0088, found 362.0093.

6,6,6-Trichloro-*N*-(4-(*tert*-butyl)phenyl)hexanamide (**6d**)

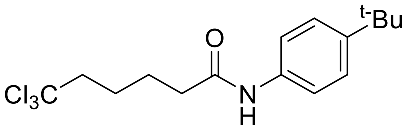

57.5 mg, light yellow solid, yield: 41%. Eluent: pentane/ethyl acetate/dichloromethane = 15/1/1 to 8/1/1.

^{1}H NMR (400 MHz, $CDCl_3$) δ 7.65 (s, 1H), 7.43 (d, *J* = 8.6 Hz, 2H), 7.32 (d, *J* = 8.7 Hz, 2H), 2.69 (t, *J* = 7.3 Hz, 2H), 2.40 (t, *J* = 6.7 Hz, 2H), 1.91 – 1.77 (m, *J* = 4.8 Hz, 4H), 1.30 (s, 9H).

^{13}C NMR (101 MHz, $CDCl_3$) δ 170.6, 147.3, 135.1, 125.7, 119.8, 99.7, 54.7, 37.0, 34.3, 31.3, 26.0, 24.2.

HR-MS (ESI-TOF) m/z: $[M+H]^+$ calcd. for $C_{16}H_{23}Cl_3NO$ 350.0840, found 350.0846.

6,6,6-Trichloro-*N*-(4-(methylthio)phenyl)hexanamide (**6e**)

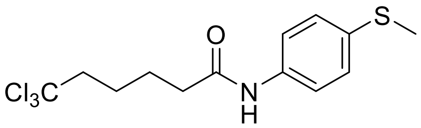

54.5 mg, light yellow solid, yield: 40%. Eluent: pentane/ethyl acetate/dichloromethane = 15/1/1 to 7/1/1.

^{1}H NMR (700 MHz, $CDCl_3$) δ 7.68 (s, 1H), 7.42 (d, *J* = 8.3 Hz, 2H), 7.20 (d, *J* = 8.6 Hz, 2H), 2.71 – 2.64 (m, 2H), 2.44 (s, 3H), 2.39 (t, *J* = 6.9 Hz, 2H), 1.87 – 1.76 (m, 4H).

^{13}C NMR (176 MHz, $CDCl_3$) δ 170.7, 135.3, 133.7, 127.8, 120.7, 99.6, 54.7, 36.9, 26.0, 24.0, 16.5.

HR-MS (ESI-TOF) m/z: $[M+H]^+$ calcd. for $C_{13}H_{17}Cl_3NOS$ 340.0091, found 340.0094.

6,6,6-Trichloro-*N*-(4-(trifluoromethyl)phenyl)hexanamide (**6f**)

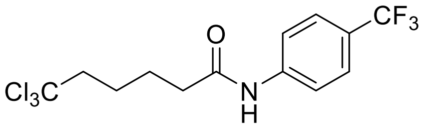

50.8 mg, light yellow oil, yield: 35%. Eluent: pentane/ethyl acetate/dichloromethane = 15/1/1 to 8/1/1.

^{1}H NMR (700 MHz, $CDCl_3$) δ 7.64 (d, *J* = 8.4 Hz, 2H), 7.56 (d, *J* = 8.4 Hz, 2H), 7.52 (s, 1H), 2.75 – 2.68 (m, 2H), 2.46 (t, *J* = 6.7 Hz, 2H), 1.90 – 1.82 (m, 4H).

^{13}C NMR (176 MHz, $CDCl_3$) δ 170.8, 140.8, 126.3 (q, *J* = 3.6 Hz), 126.0, 124.0 (q, *J* = 271.8 Hz), 119.4, 99.6, 54.7, 37.1, 26.0, 23.9.

^{19}F NMR (376 MHz, $CDCl_3$) δ -62.1.

HR-MS (ESI-TOF) m/z: $[M+H]^+$ calcd. for $C_{13}H_{14}Cl_3F_3NO$ 362.0088, found 362.0082.

6,6,6-Trichloro-*N*-(4-(trifluoromethoxy)phenyl)hexanamide (**6g**)

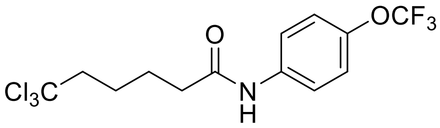

59.1 mg, light yellow oil, yield: 39%. Eluent: pentane/ethyl acetate/dichloromethane = 15/1/1 to 8/1/1.

^{1}H NMR (700 MHz, $CDCl_3$) δ 7.82 – 7.67 (m, 1H), 7.53 (d, *J* = 8.7 Hz, 2H), 7.15 (d, *J* = 8.5 Hz, 2H), 2.73 – 2.64 (m, 2H), 2.42 (t, *J* = 6.8 Hz, 2H), 1.87 – 1.78 (m, 4H).

^{13}C NMR (176 MHz, $CDCl_3$) δ 170.8, 145.3, 136.3, 121.7, 121.2, 120.4 (q, *J* = 257.0 Hz), 99.6, 54.7, 36.9, 26.0, 24.0.

^{19}F NMR (376 MHz, $CDCl_3$) δ -58.1.

HR-MS (ESI-TOF) m/z: $[M+H]^+$ calcd. for $C_{13}H_{14}Cl_3F_3NO_2$ 378.0037, found 378.0040.

6,6,6-Trichloro-*N*-(4-bromophenyl)hexanamide (**6h**)

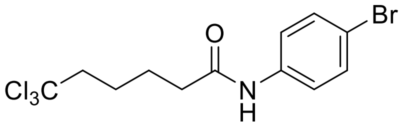

65.7 mg, light yellow oil, yield: 44%. Eluent: pentane/ethyl acetate/dichloromethane = 15/1/1 to 8/1/1.

^{1}H NMR (400 MHz, $CDCl_3$) δ 7.75 (s, 1H), 7.39 (s, 4H), 2.75 – 2.60 (m, 2H), 2.39 (t, *J* = 6.6 Hz, 2H), 1.90 – 1.70 (m, *J* = 4.9 Hz, 4H).

^{13}C NMR (101 MHz, $CDCl_3$) δ 170.8, 136.8, 131.9, 121.6, 117.0, 99.6, 54.7, 36.9, 26.0, 24.0.

HR-MS (ESI-TOF) m/z: $[M+H]^+$ calcd. for $C_{12}H_{14}ICl_3NO$ 371.9319, found 371.9322.

6,6,6-Trichloro-*N*-(4-iodophenyl)hexanamide (**6i**)

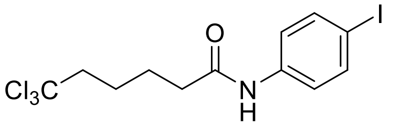

77.4 mg, light yellow solid, yield: 46%. Eluent: pentane/ethyl acetate/dichloromethane = 15/1/1 to 8/1/1.

^{1}H NMR (700 MHz, $CDCl_3$) δ 7.67 (s, 1H), 7.58 (d, *J* = 8.4 Hz, 2H), 7.27 (d, *J* = 8.4 Hz, 2H), 2.71 – 2.65 (m, 2H), 2.39 (t, *J* = 6.9 Hz, 2H), 1.86 – 1.76 (m, 4H).

^{13}C NMR (176 MHz, $CDCl_3$) δ 170.8, 137.8, 137.5, 121.8, 99.6, 87.6, 54.7, 37.0, 26.0, 23.9.

HR-MS (ESI-TOF) m/z: $[M+H]^+$ calcd. for $C_{12}H_{14}ICl_3NO$ 419.9180, found 419.9182.

5. ^{1}H-, ^{13}C- and ^{19}F-NMR spectra copy of products

3a

5598-yzhang-3-12.10.fid — PROTON CDCl3 {D:\NMR400\DNL0604} nmr-new 29400.13MHz

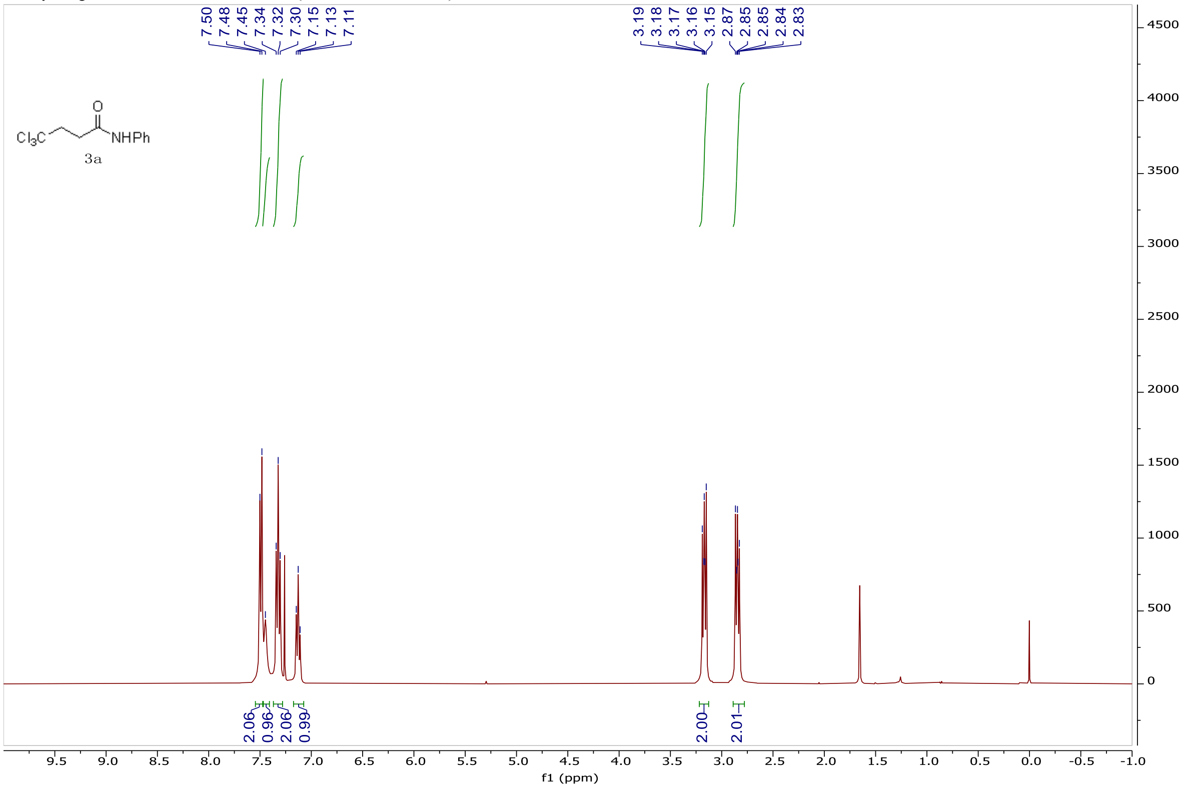

5598-yzhang-3-12.11.fid — C13CPD CDCl3 {D:\NMR400\DNL0604} nmr-new 29100.62MHz

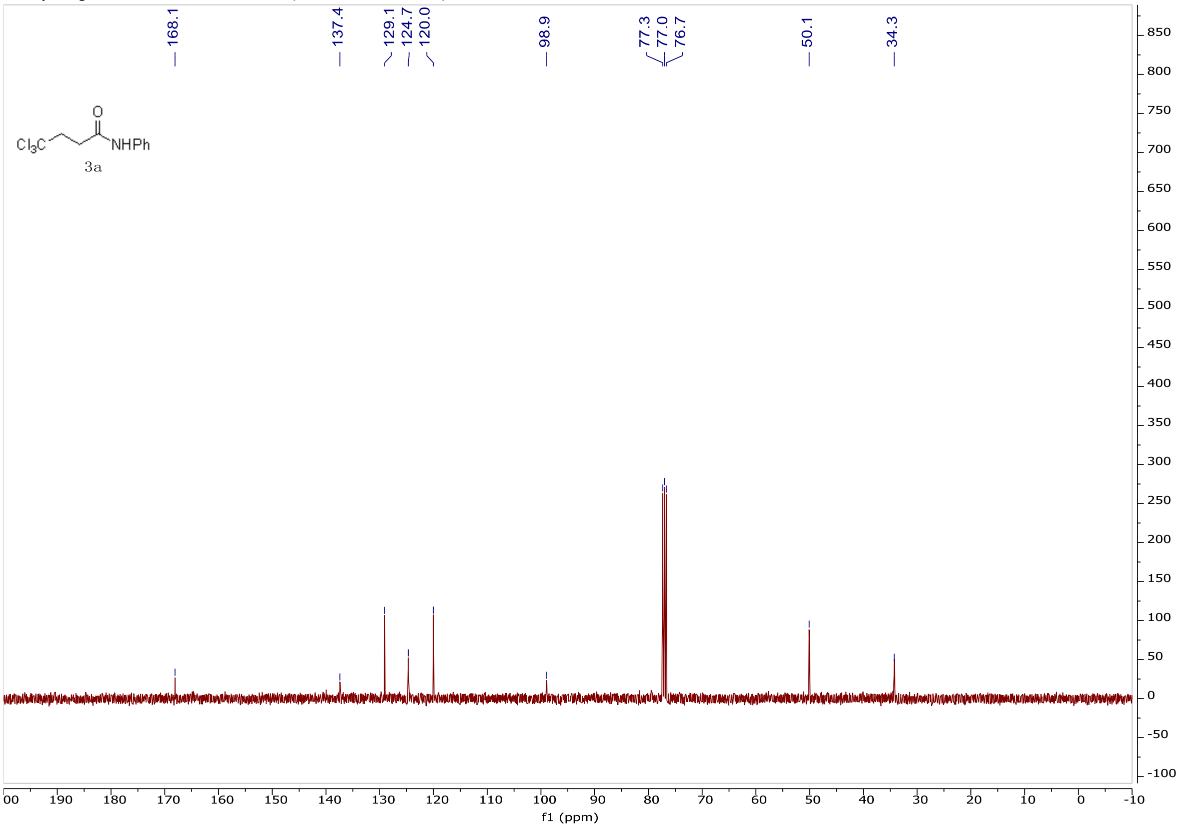

3b

7346-yzhang-3-316.10.fid — PROTON CDCl3 {D:\NMR400\DNL0604} nmr-new 30400.13MHz

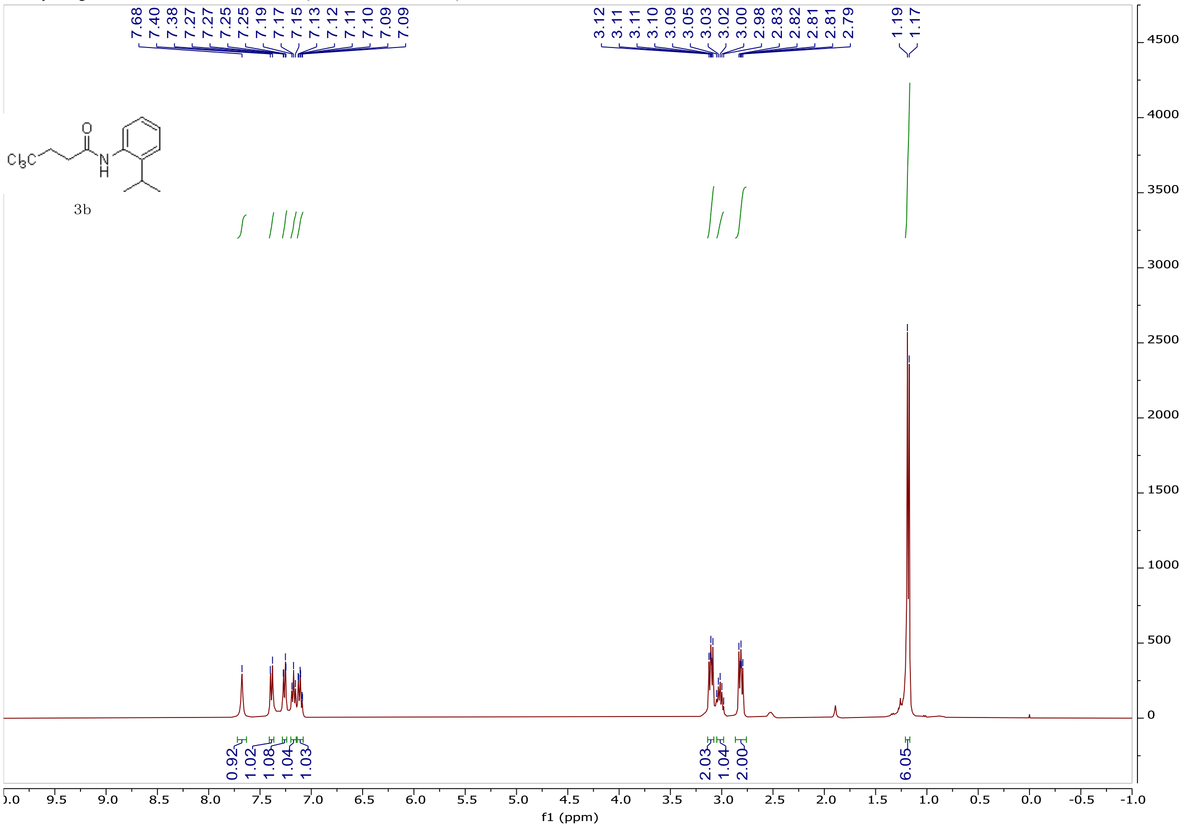

7346-yzhang-3-316.11.fid — C13CPD CDCl3 {D:\NMR400\DNL0604} nmr-new 30100.62MHz

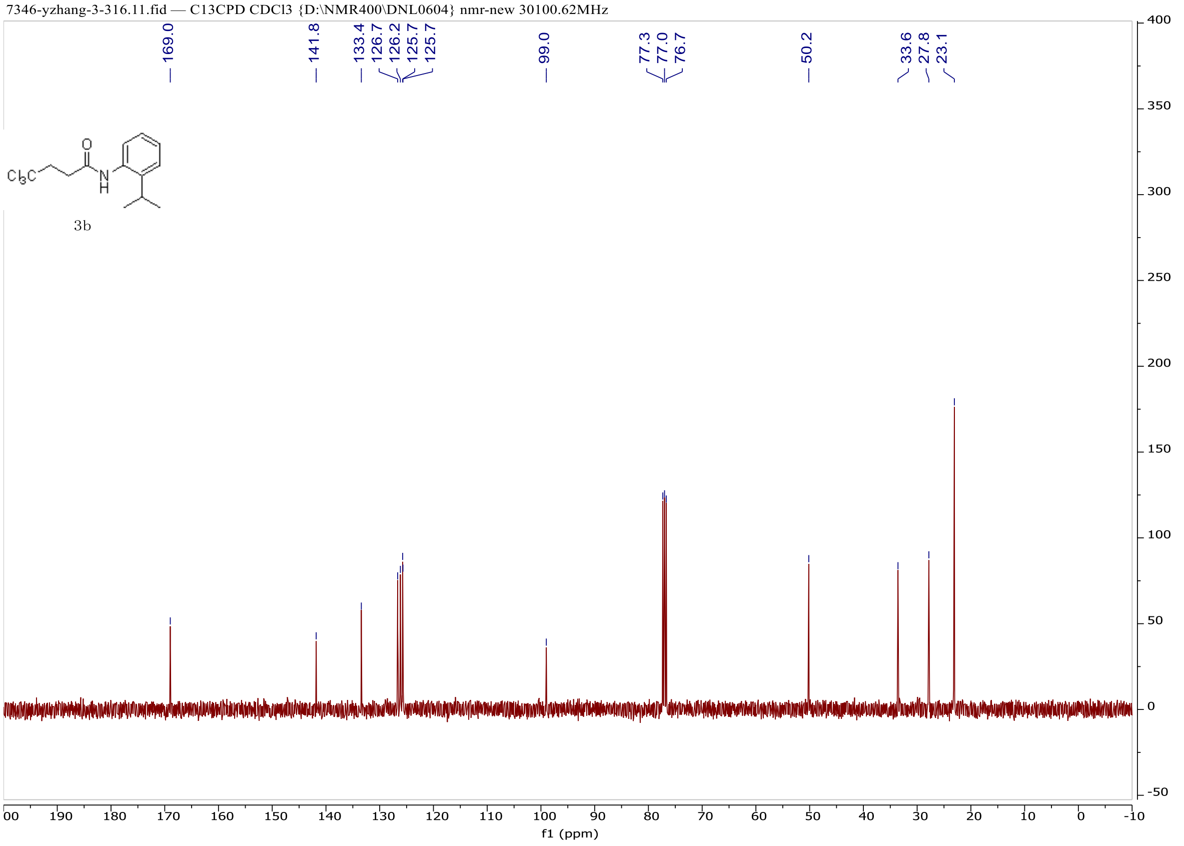

3c

6476-YZhang-3-178.10.fid — PROTON MeOD {D:\NMR400\DNL0604} nmr-new 29400.13MHz

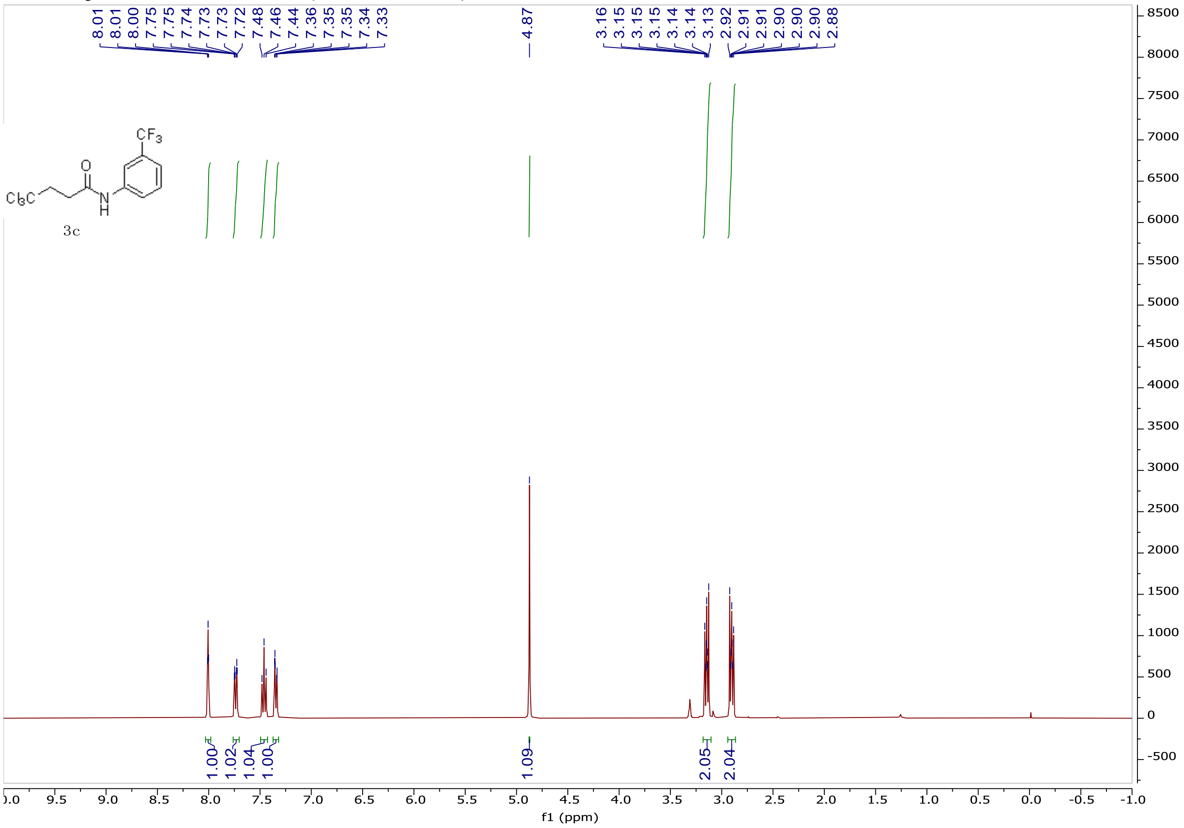

6476-YZhang-3-178.11.fid — C13CPD MeOD {D:\NMR400\DNL0604} nmr-new 29100.62MHz

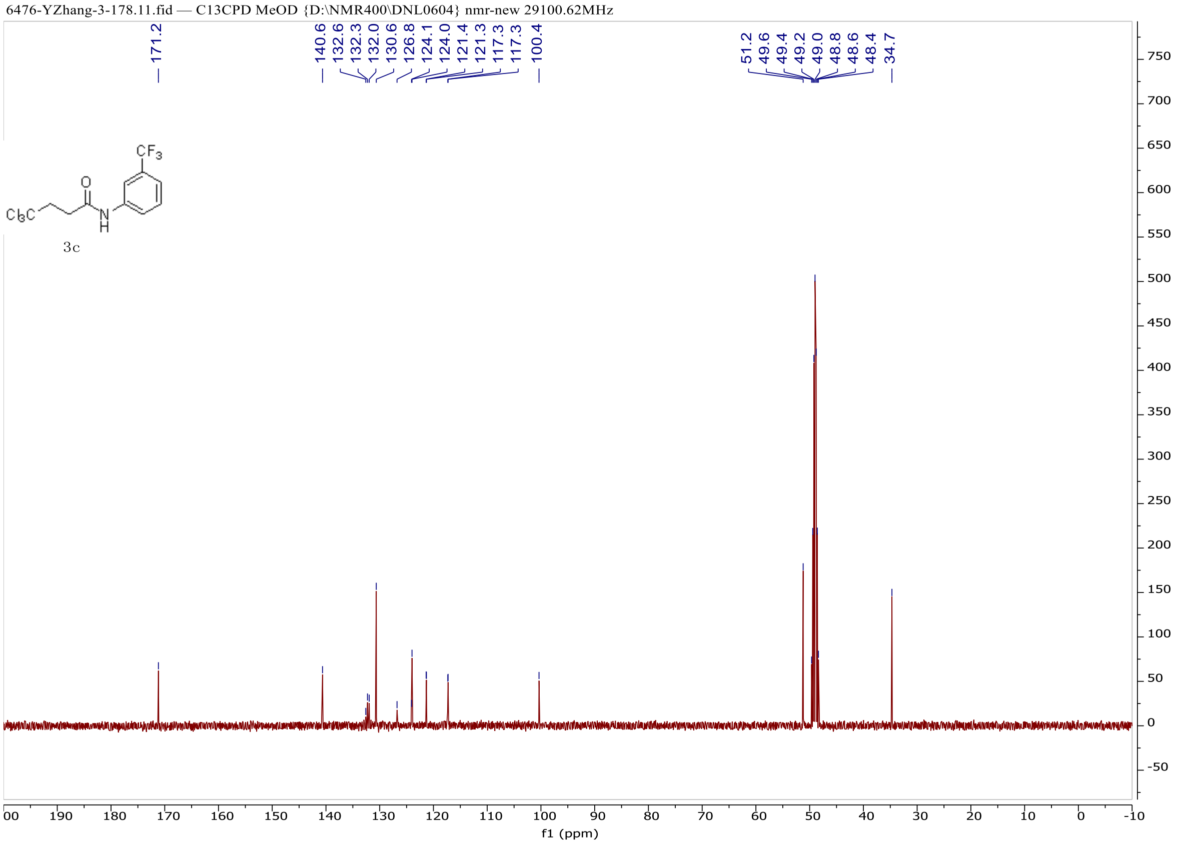

6476-YZhang-3-178.12.fid — F19CPD MeOD {D:\NMR400\DNL0604} nmr-new 29376.46MHz

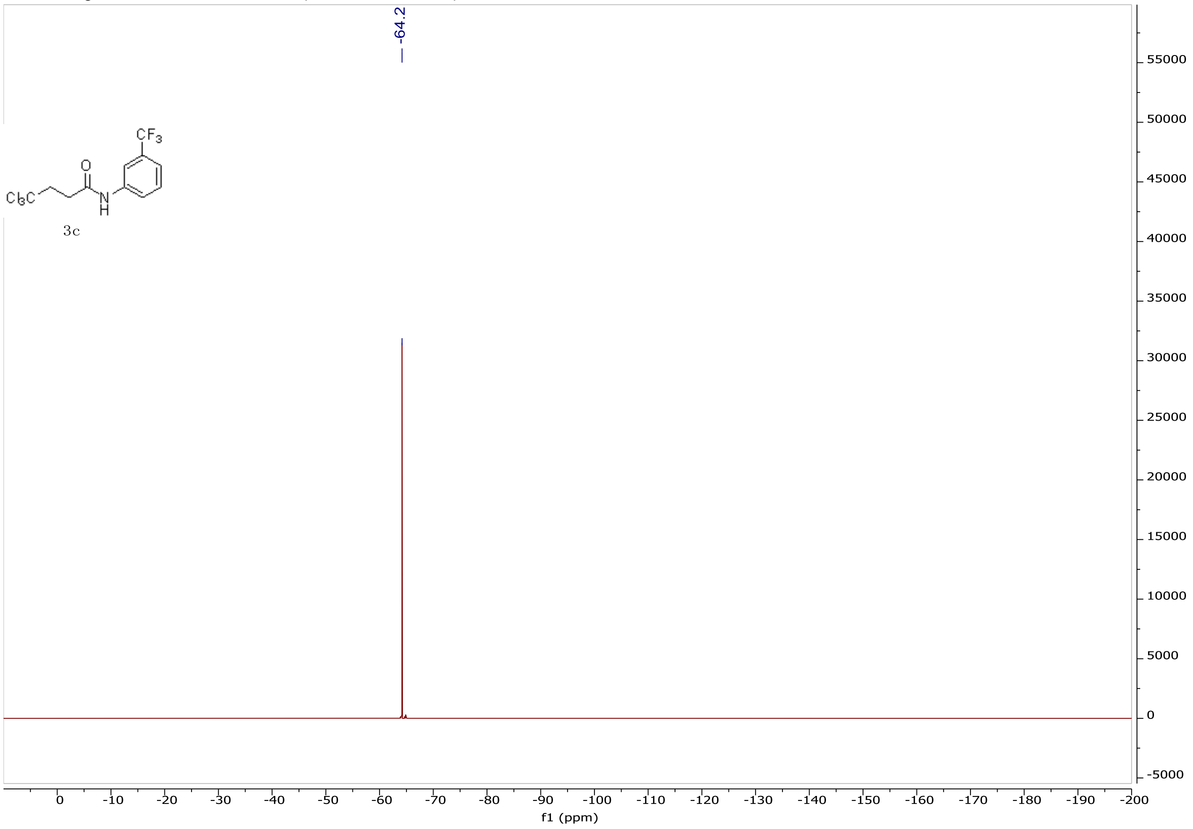

3d

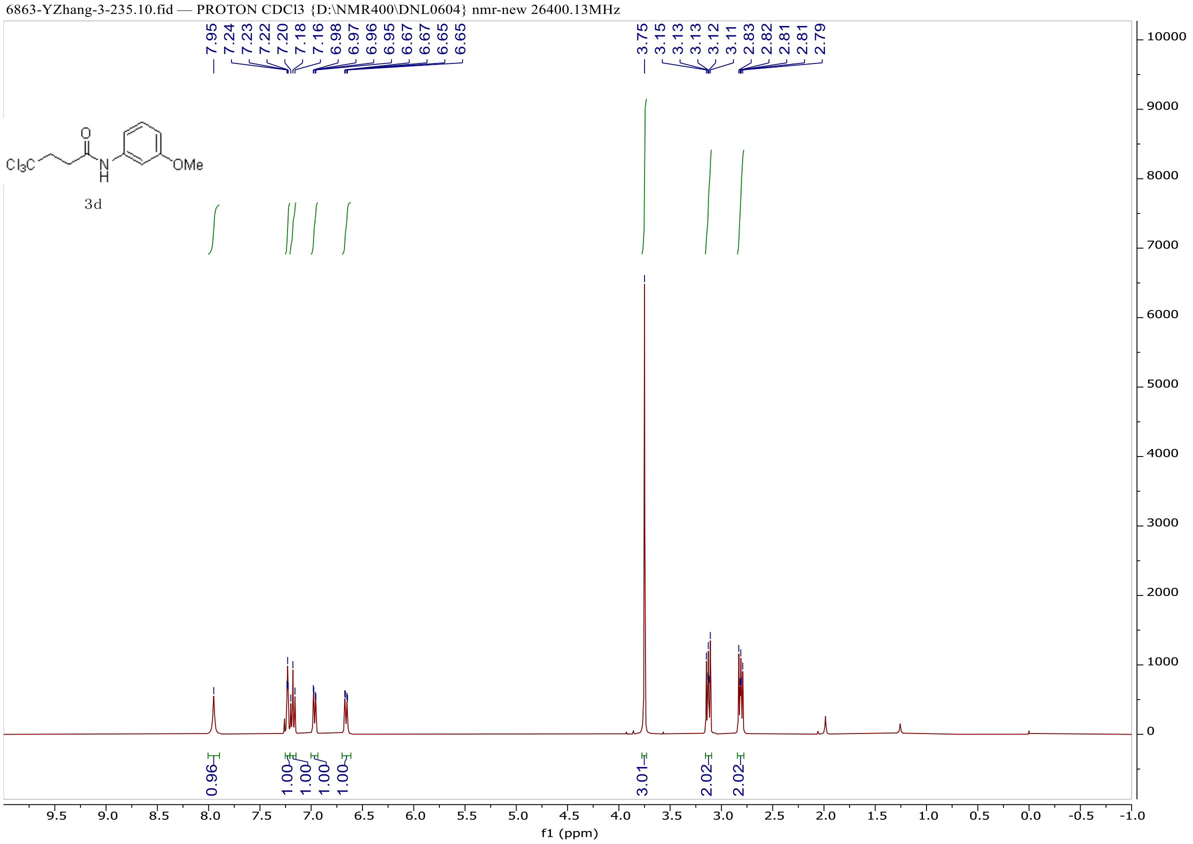
6863-YZhang-3-235.10.fid — PROTON CDCl3 {D:\NMR400\DNL0604} nmr-new 26400.13MHz
7.95
7.24
7.23
7.22
7.20
7.18
7.16
6.98
6.97
6.96
6.95
6.67
6.67
6.65
6.65
3.75
3.15
3.13
3.13
3.12
3.11
2.83
2.82
2.81
2.81
2.79
Cl3C
O
N
H
OMe
3d
0.96
1.00
1.00
1.00
1.00
3.01
2.02
2.02
f1 (ppm)

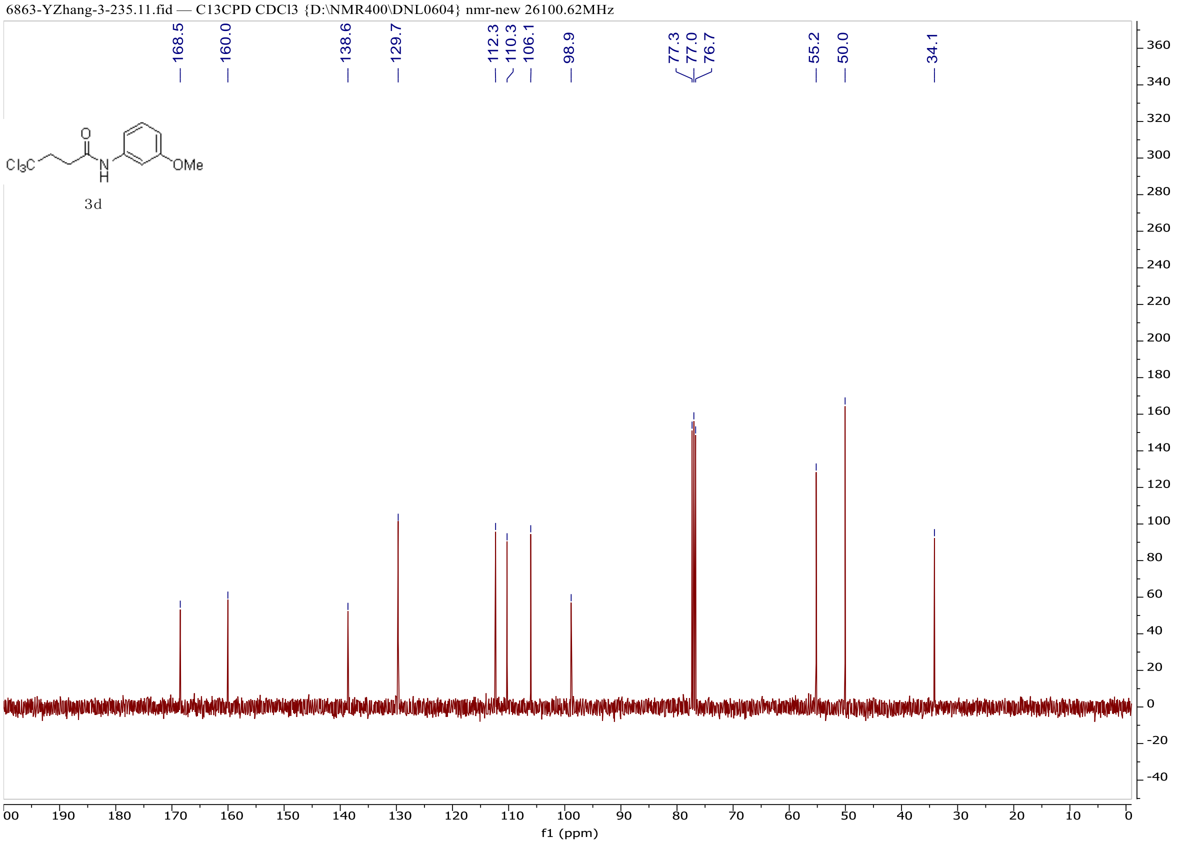
6863-YZhang-3-235.11.fid — C13CPD CDCl3 {D:\NMR400\DNL0604} nmr-new 26100.62MHz
168.5
160.0
138.6
129.7
112.3
110.3
106.1
98.9
77.3
77.0
76.7
55.2
50.0
34.1
Cl3C
O
N
H
OMe
3d
f1 (ppm)

3e

6606-yzhang-3-187.10.fid — PROTON DMSO {D:\NMR400\DNL0604} nmr-new 53400.13MHz

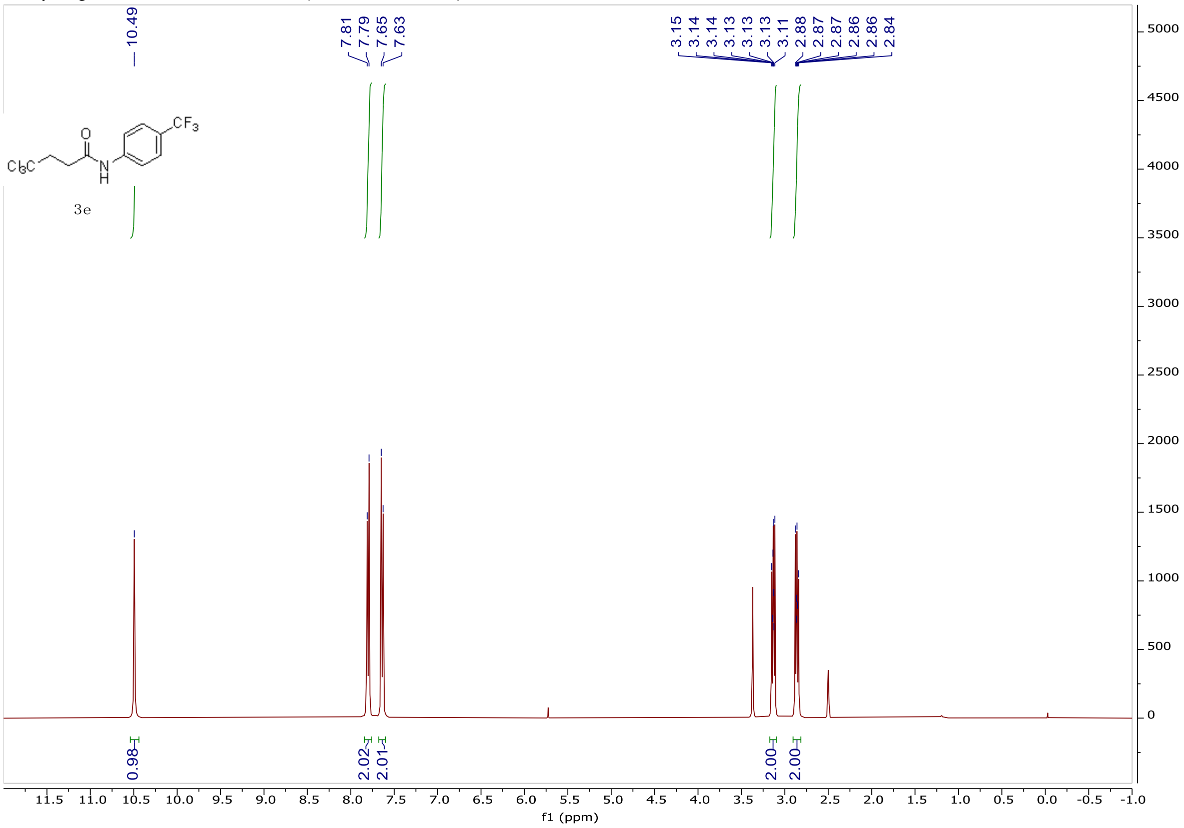

6606-yzhang-3-187.11.fid — C13CPD DMSO {D:\NMR400\DNL0604} nmr-new 53100.62MHz

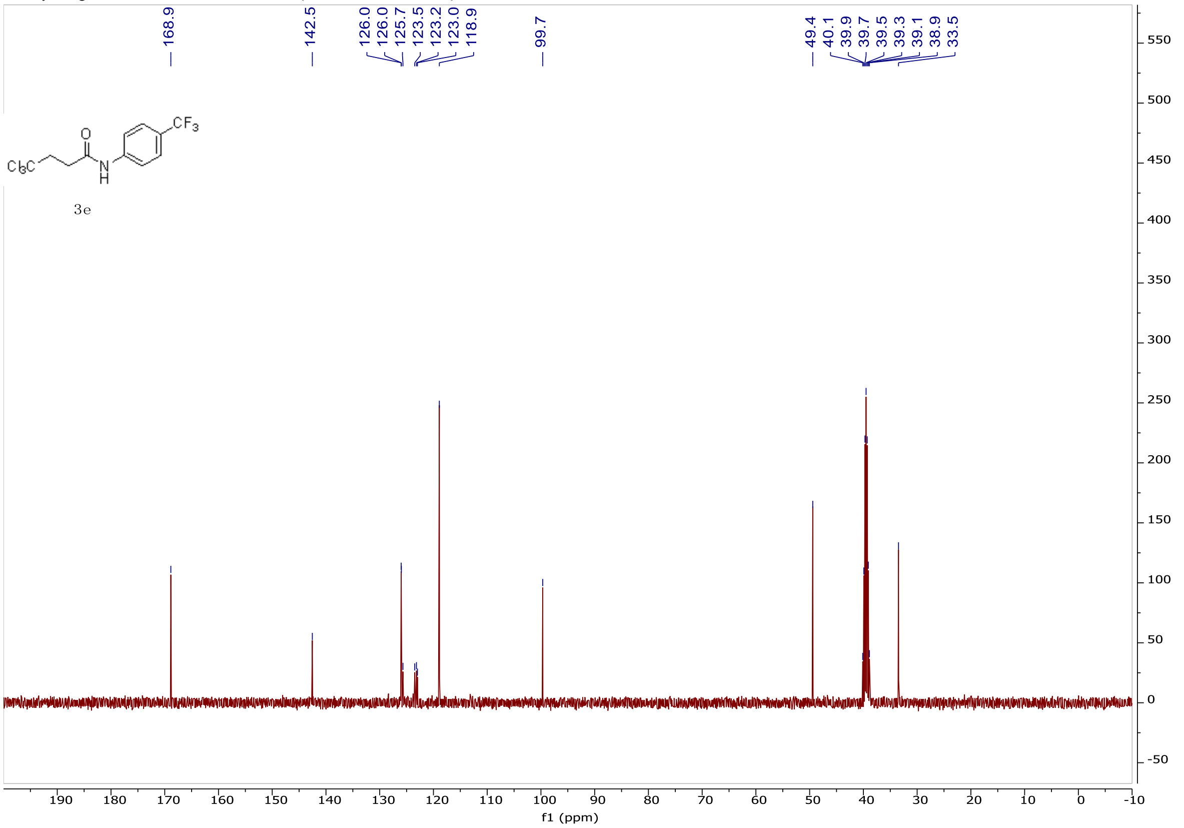

6606-yzhang-3-187.12.fid — F19 DMSO {D:\NMR400\DNL0604} nmr-new 53376.46MHz

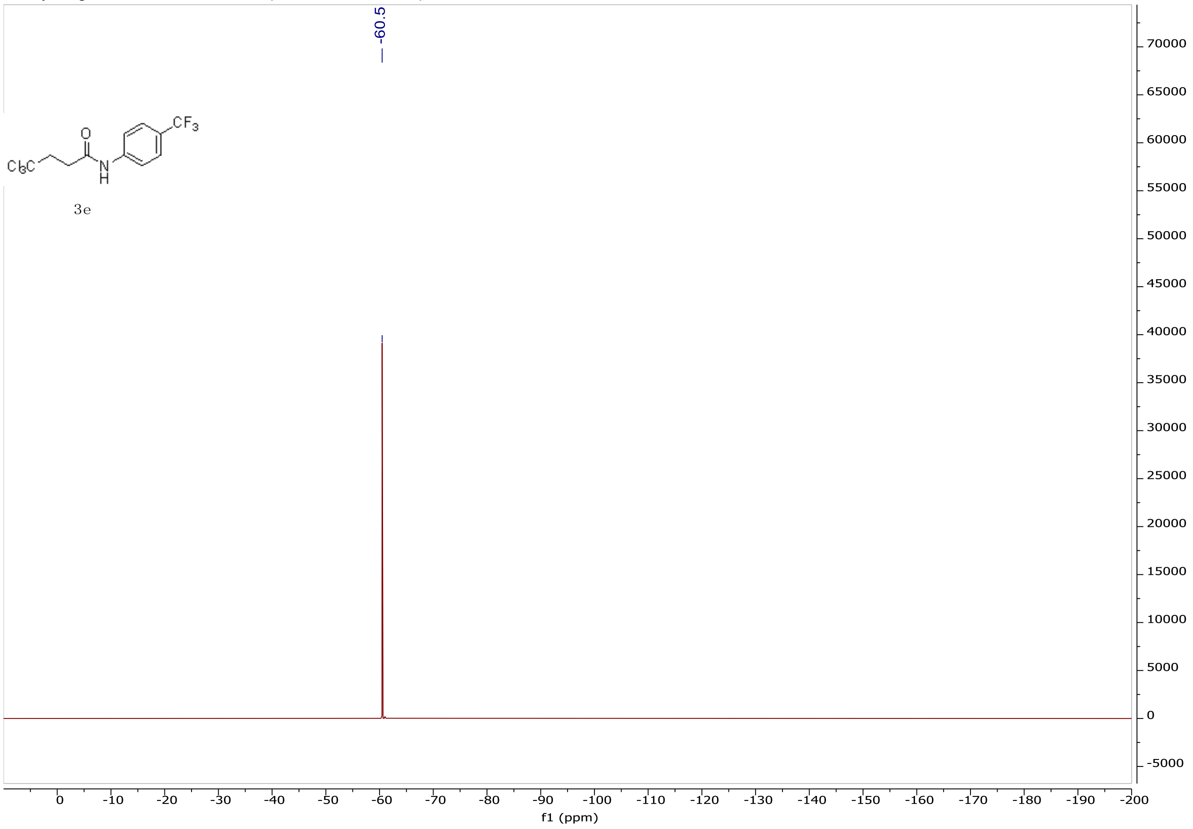

3f

6607-yzhang-3-188.10.fid — PROTON CDCl3 {D:\NMR400\DNL0604} nmr-new 24400.13MHz

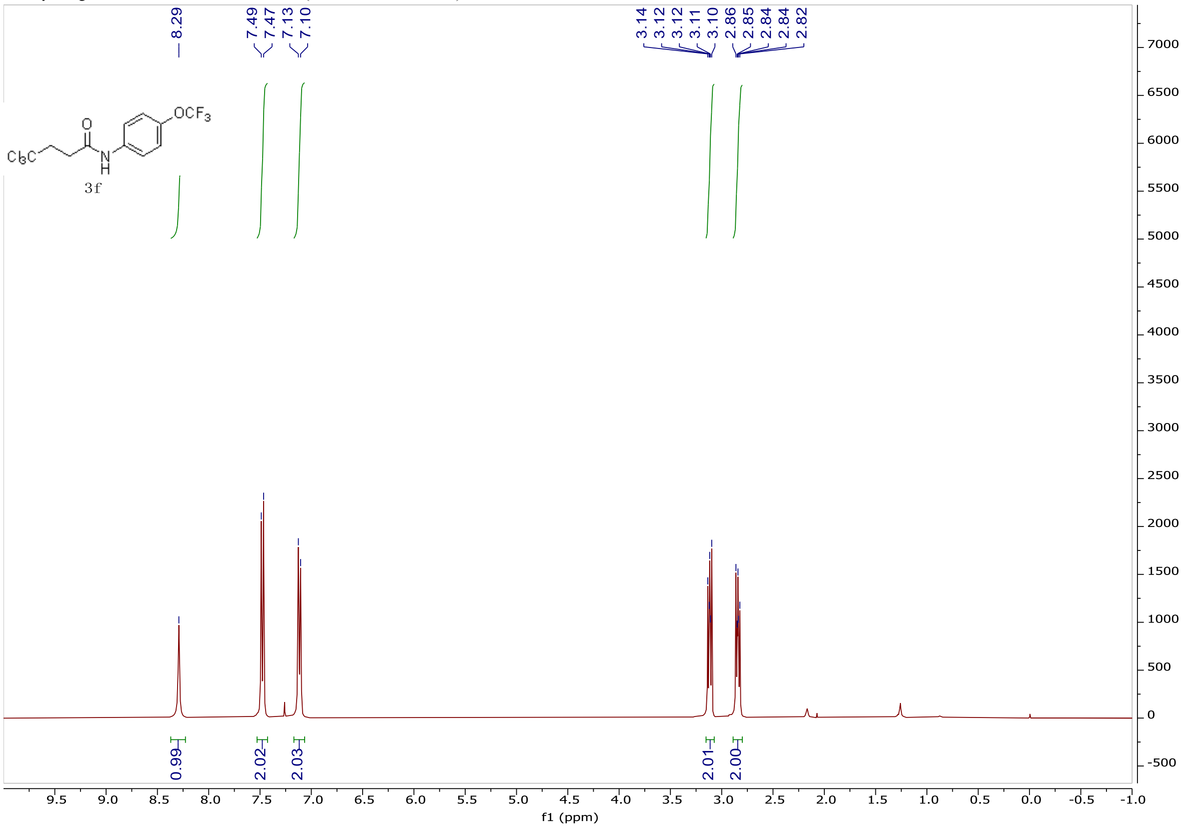

6607-yzhang-3-188.11.fid — C13CPD CDCl3 {D:\NMR400\DNL0604} nmr-new 24100.62MHz

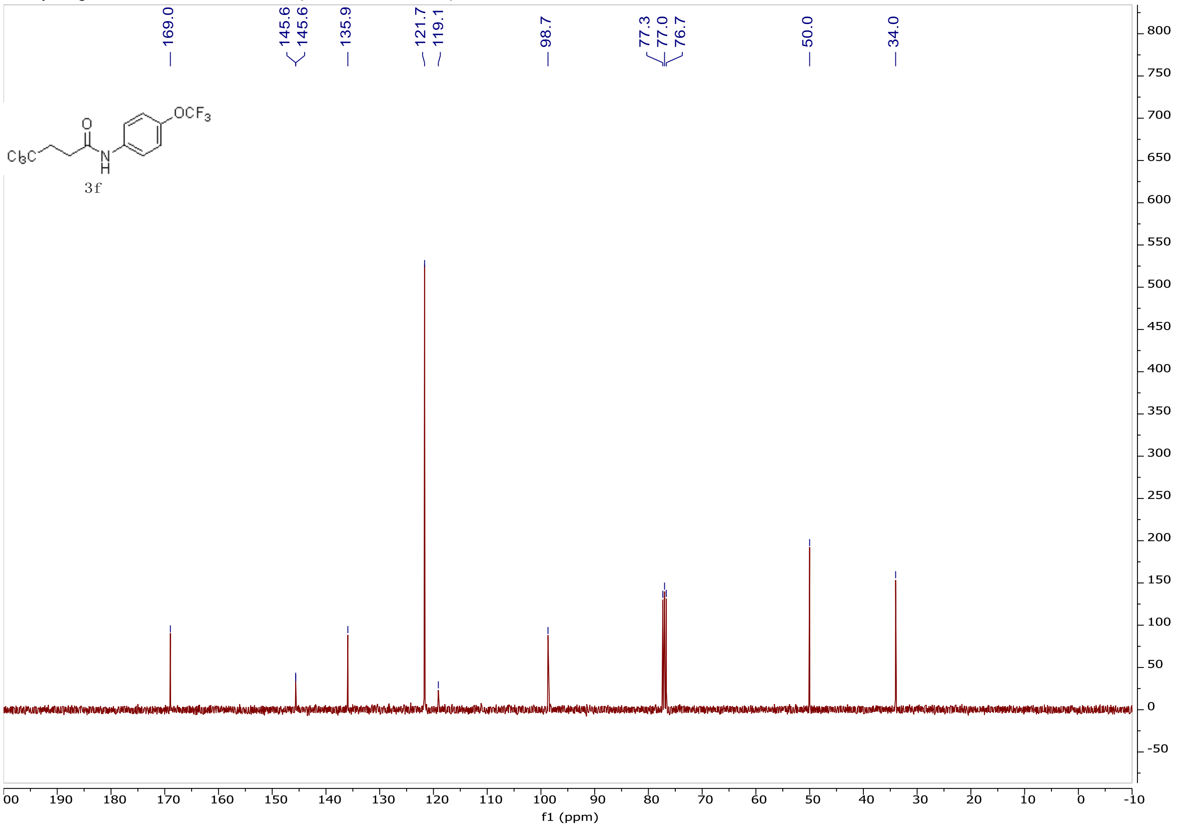

6607-yzhang-3-188.12.fid — F19 CDCl3 {D:\NMR400\DNL0604} nmr-new 24376.46MHz

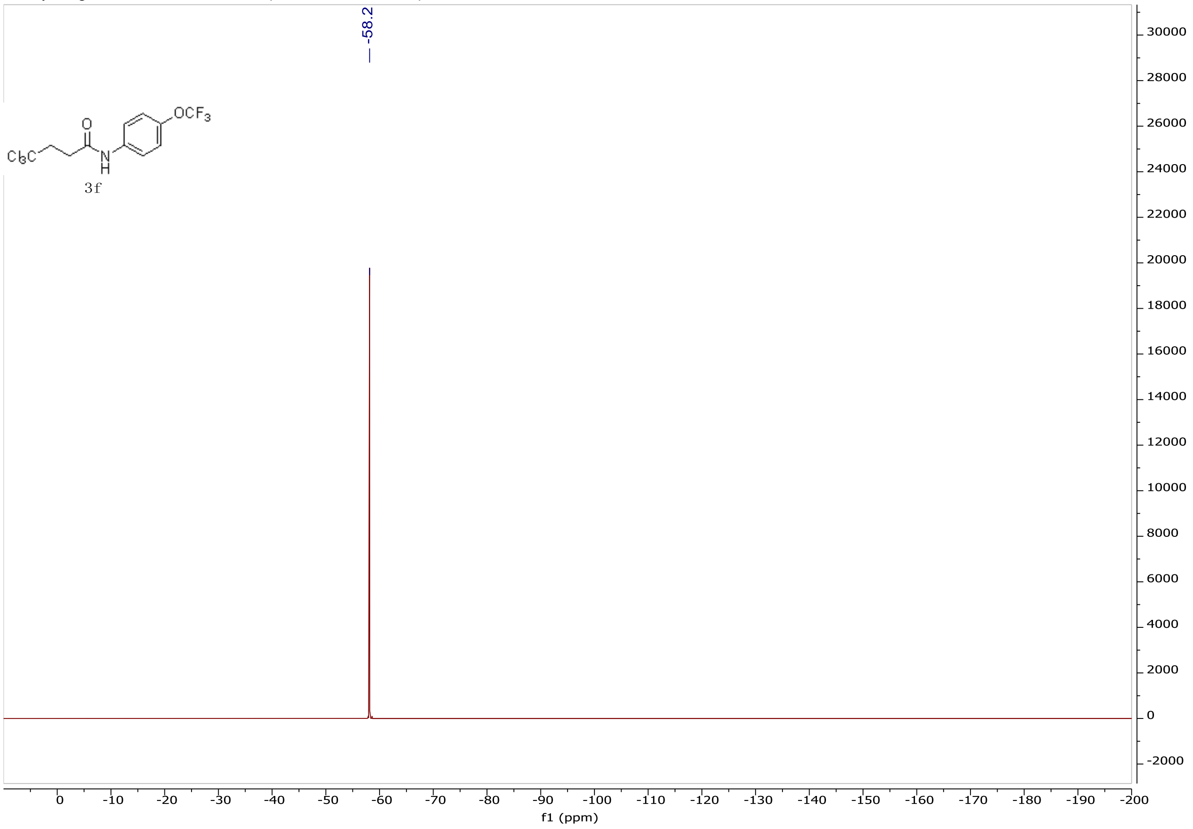

3g

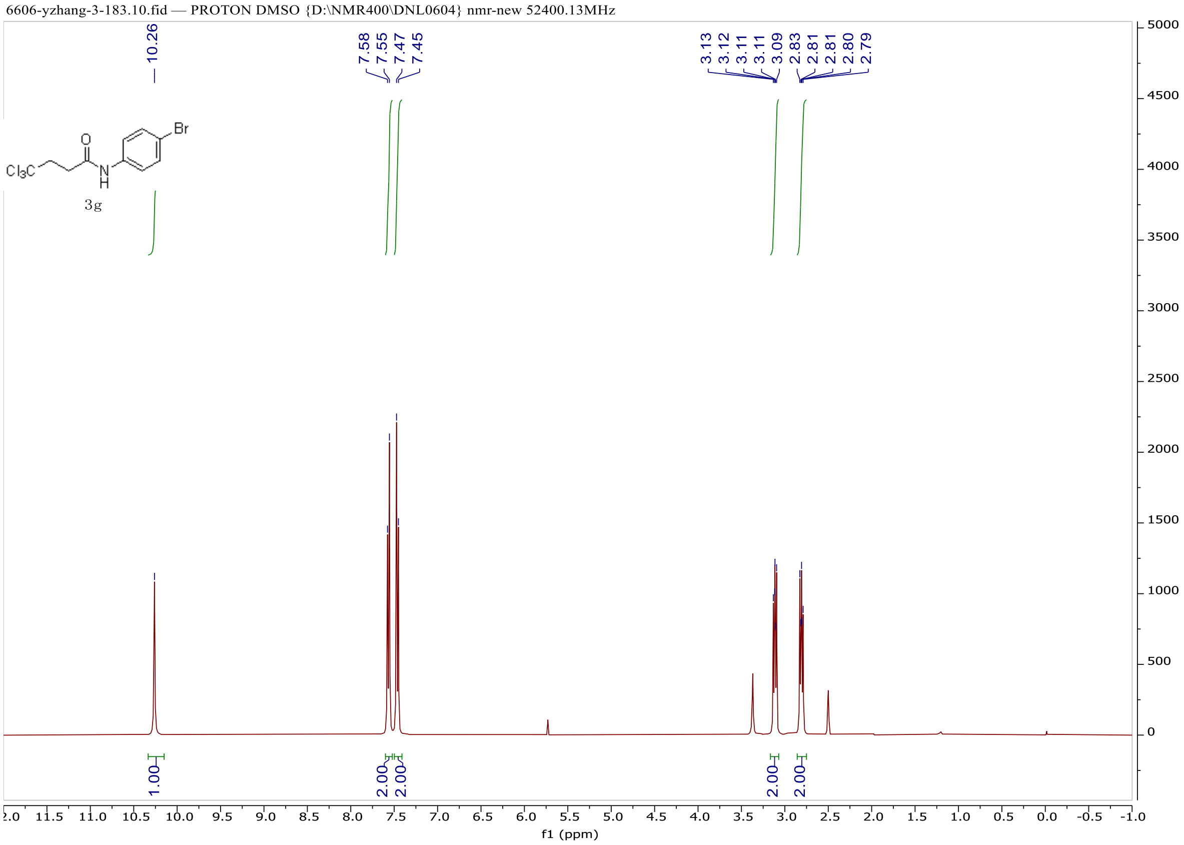
6606-yzhang-3-183.10.fid — PROTON DMSO {D:\NMR400\DNL0604} nmr-new 52400.13MHz
10.26
7.58
7.55
7.47
7.45
3.13
3.12
3.11
3.11
3.09
2.83
2.81
2.81
2.80
2.79
Br
O
Cl3C
N
H
3g
1.00
2.00
2.00
2.00
2.00
f1 (ppm)

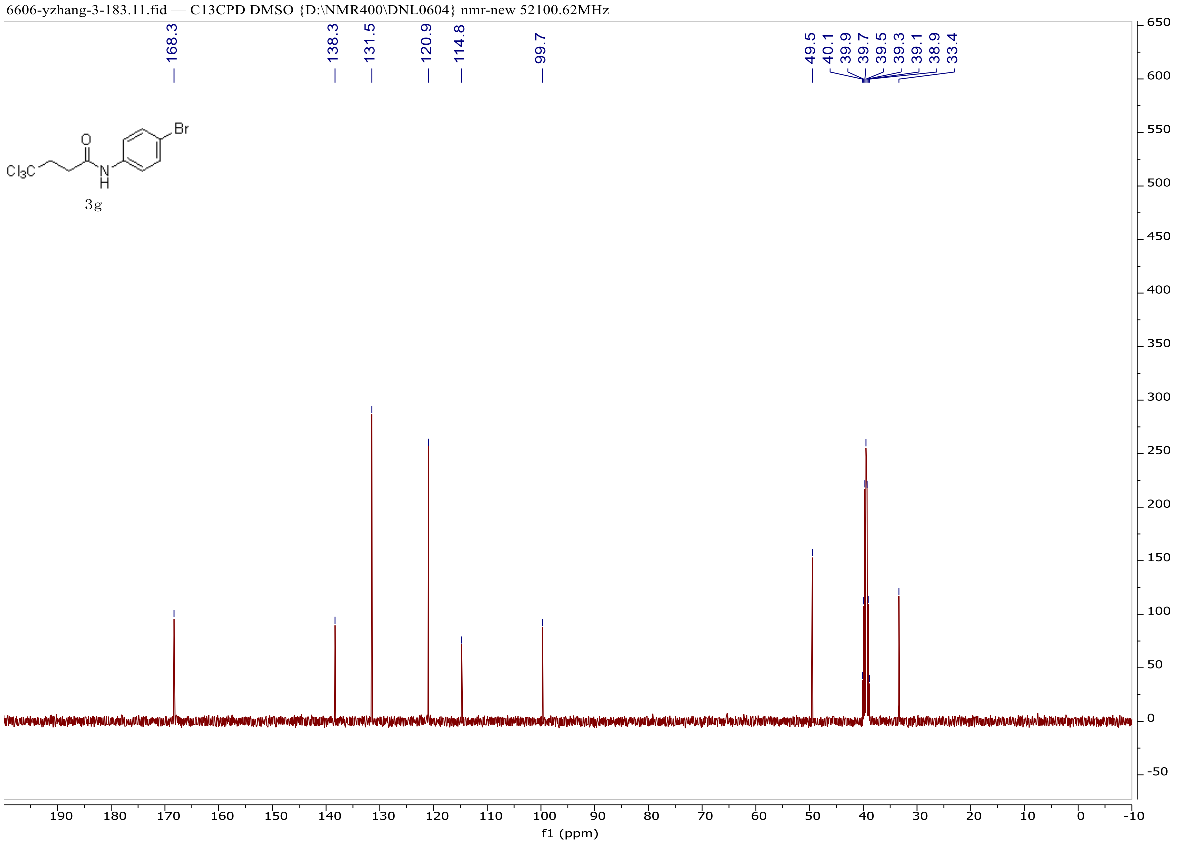
6606-yzhang-3-183.11.fid — C13CPD DMSO {D:\NMR400\DNL0604} nmr-new 52100.62MHz
168.3
138.3
131.5
120.9
114.8
99.7
49.5
40.1
39.9
39.7
39.5
39.3
39.1
38.9
33.4
Br
O
Cl3C
N
H
3g
f1 (ppm)

3h

6606-yzhang-3-189.10.fid — PROTON DMSO {D:\NMR400\DNL0604} nmr-new 54400.13MHz

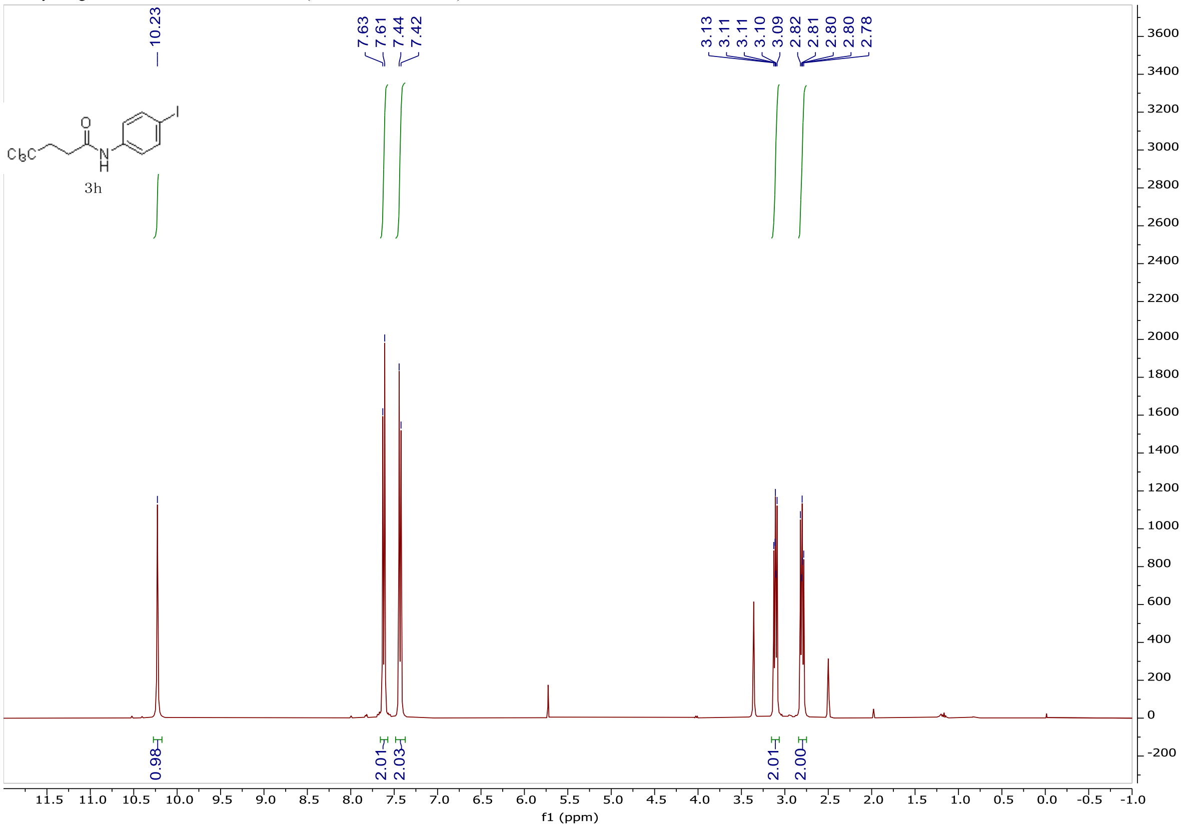

6606-yzhang-3-189.11.fid — C13CPD DMSO {D:\NMR400\DNL0604} nmr-new 54100.62MHz

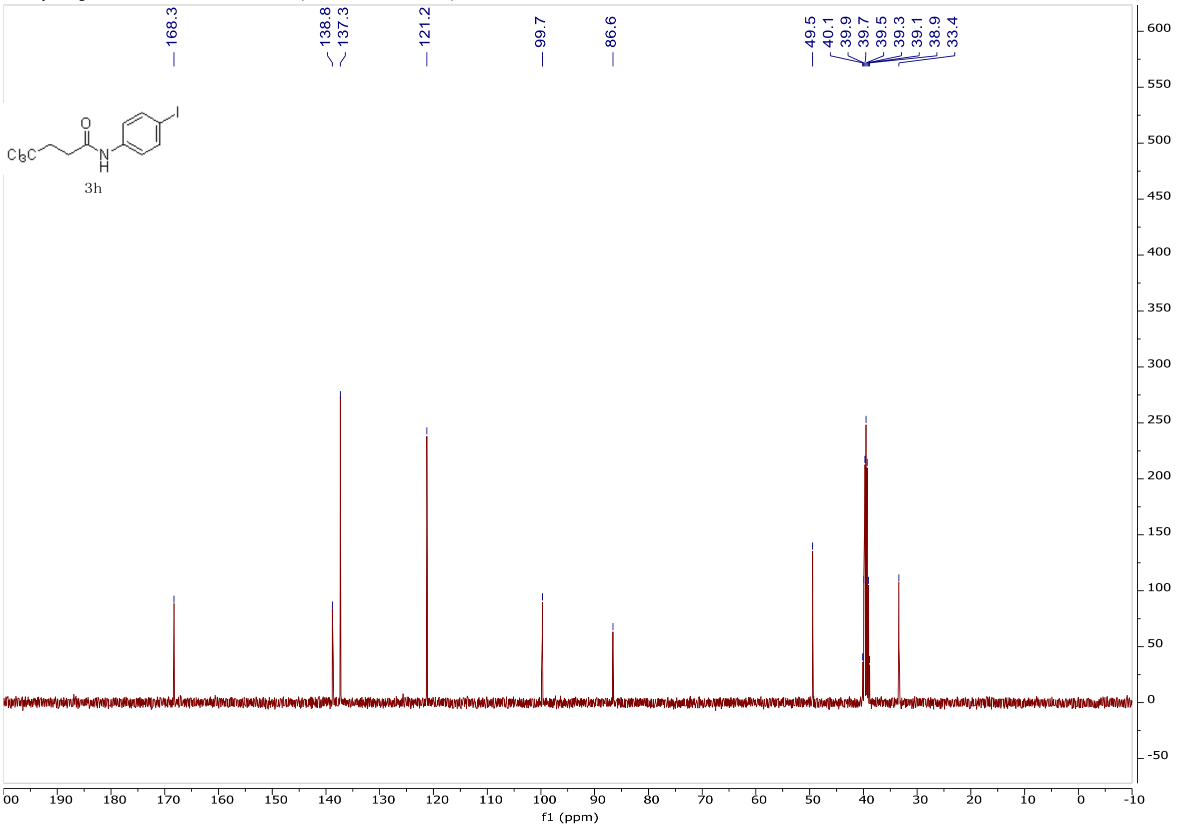

3i

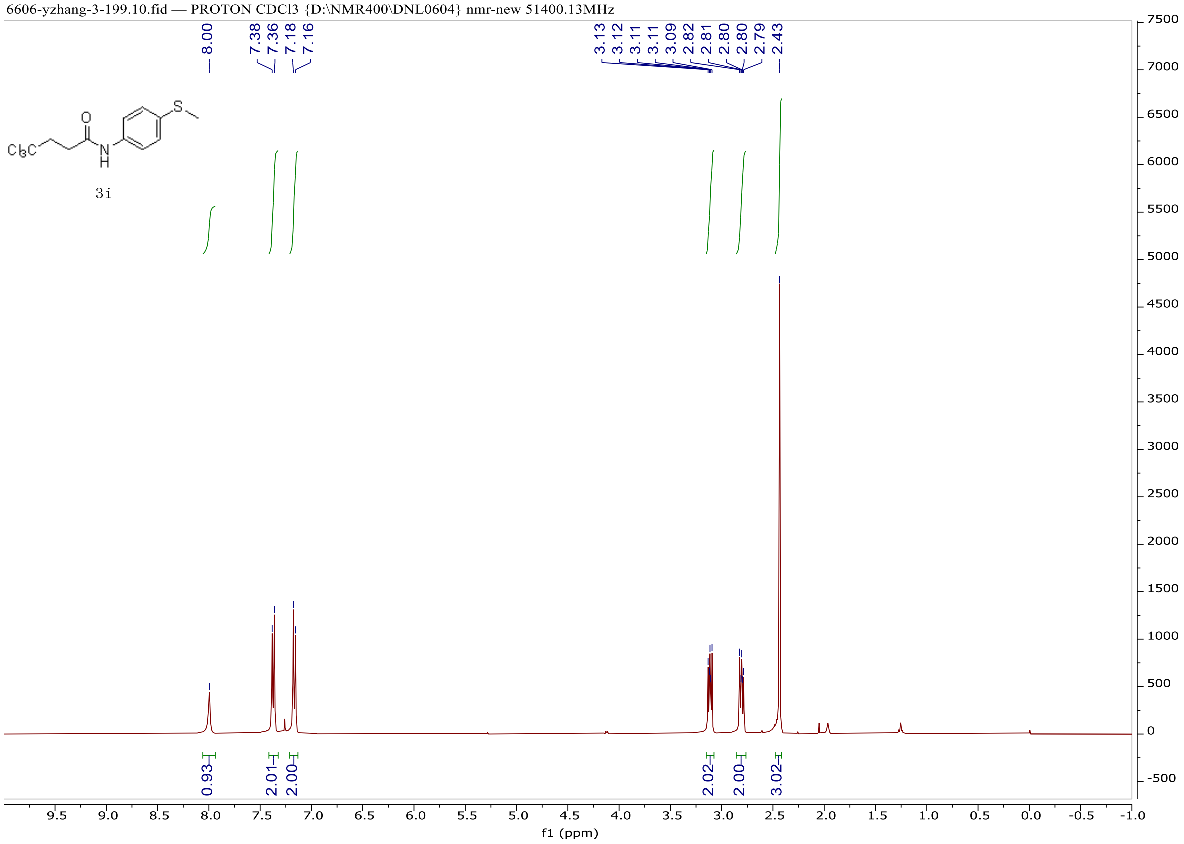
6606-yzhang-3-199.10.fid — PROTON CDCl3 {D:\NMR400\DNL0604} nmr-new 51400.13MHz
8.00
7.38
7.36
7.18
7.16
3.13
3.12
3.11
3.11
3.09
2.82
2.81
2.80
2.80
2.79
2.43
0.93
2.01
2.00
2.02
2.00
3.02
3i
f1 (ppm)

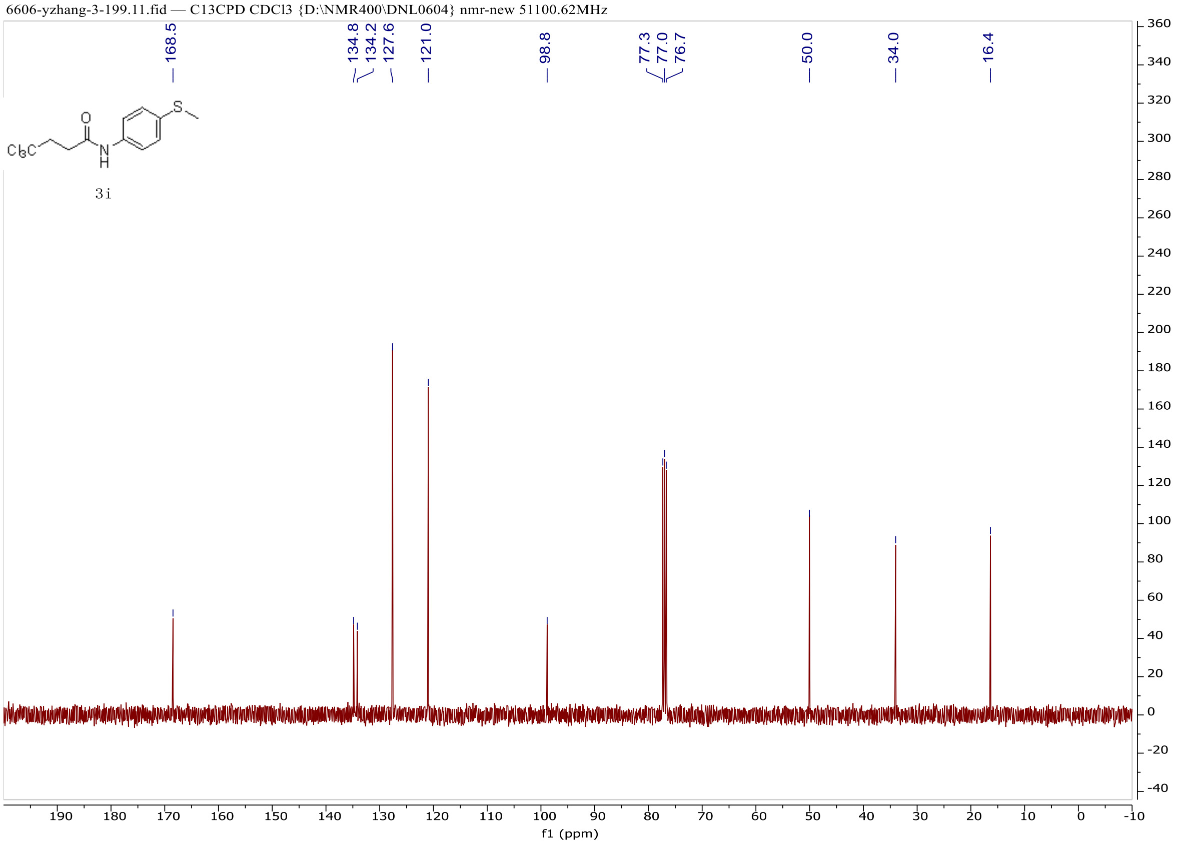
6606-yzhang-3-199.11.fid — C13CPD CDCl3 {D:\NMR400\DNL0604} nmr-new 51100.62MHz
168.5
134.8
134.2
127.6
121.0
98.8
77.3
77.0
76.7
50.0
34.0
16.4
3i
f1 (ppm)

3j

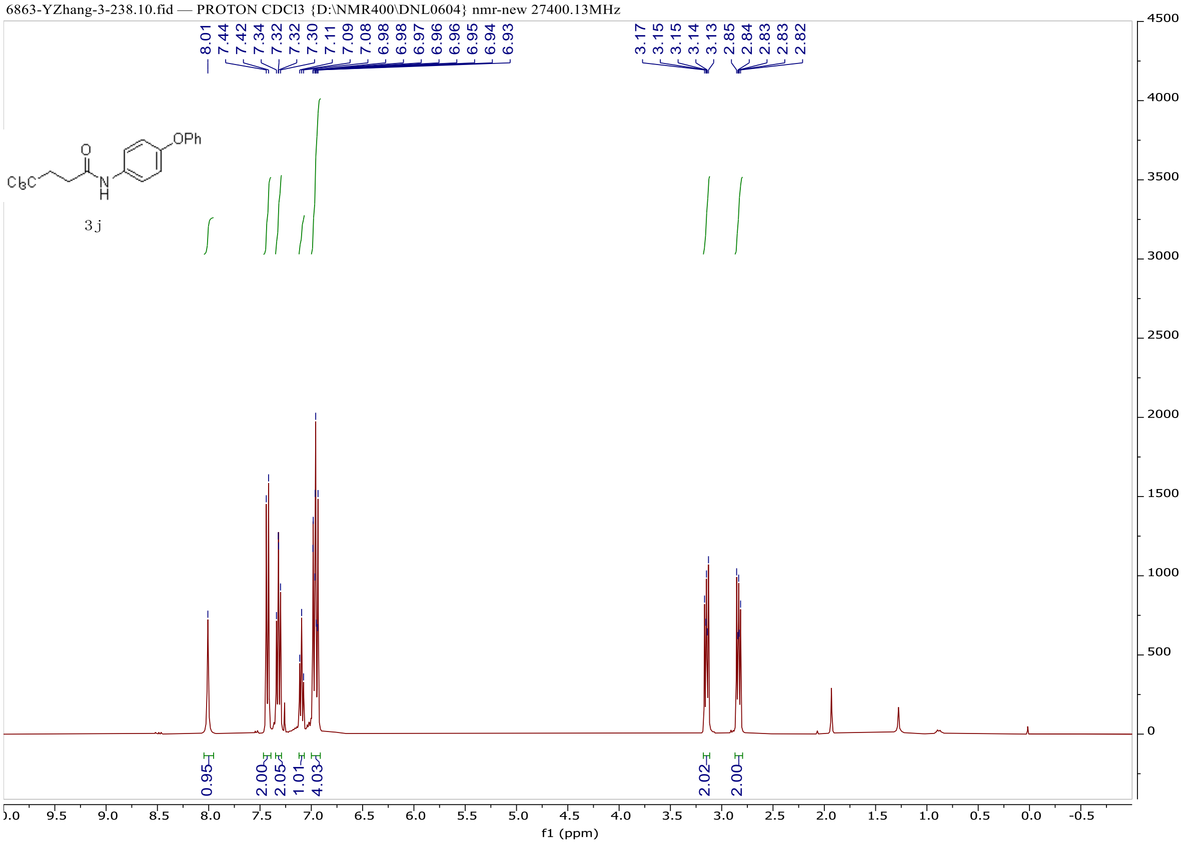
6863-YZhang-3-238.10.fid — PROTON CDCl3 {D:\NMR400\DNL0604} nmr-new 27400.13MHz
8.01
7.44
7.42
7.34
7.32
7.32
7.30
7.11
7.09
7.08
6.98
6.98
6.97
6.96
6.96
6.95
6.94
6.93
3.17
3.15
3.15
3.14
3.13
2.85
2.84
2.83
2.83
2.82
OPh
O
Cl3C
N
H
3j
0.95
2.00
2.05
1.01
4.03
2.02
2.00
f1 (ppm)

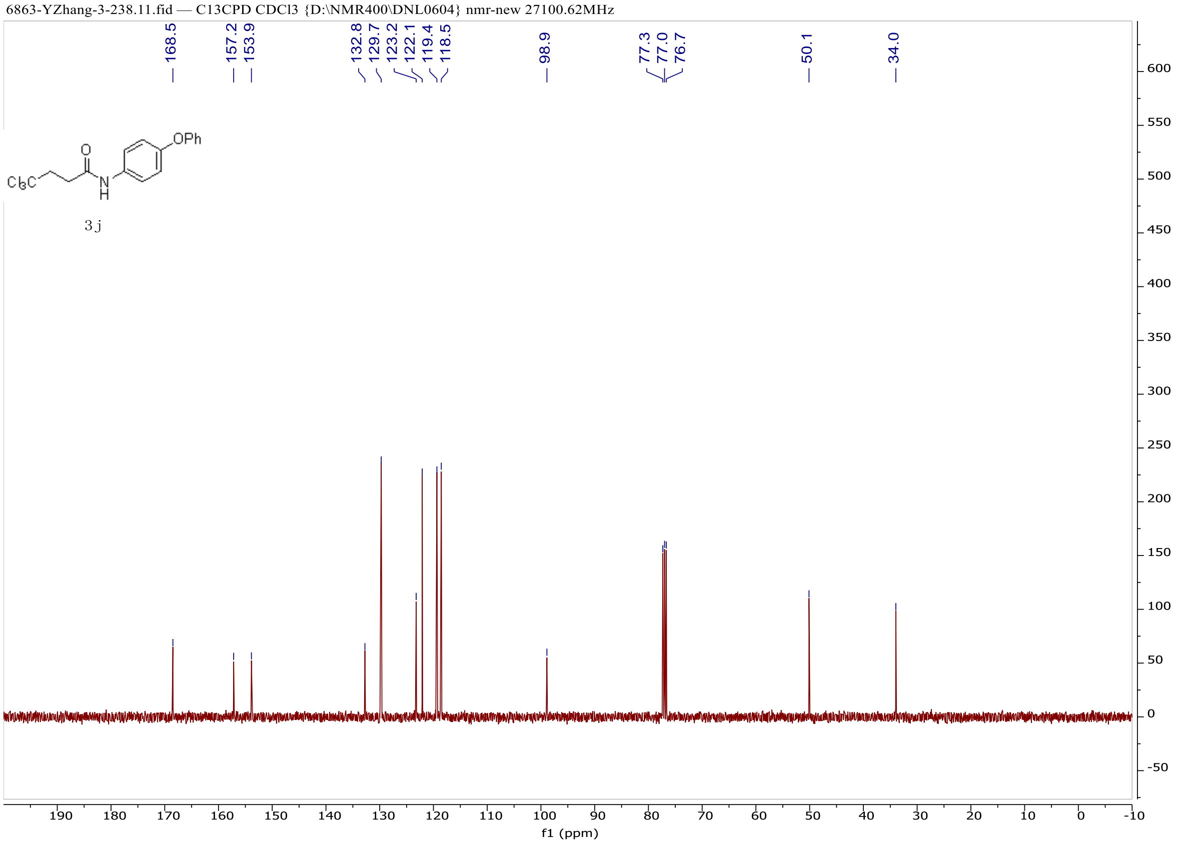
6863-YZhang-3-238.11.fid — C13CPD CDCl3 {D:\NMR400\DNL0604} nmr-new 27100.62MHz
168.5
157.2
153.9
132.8
129.7
123.2
122.1
119.4
118.5
98.9
77.3
77.0
76.7
50.1
34.0
OPh
O
Cl3C
N
H
3j
f1 (ppm)

3k

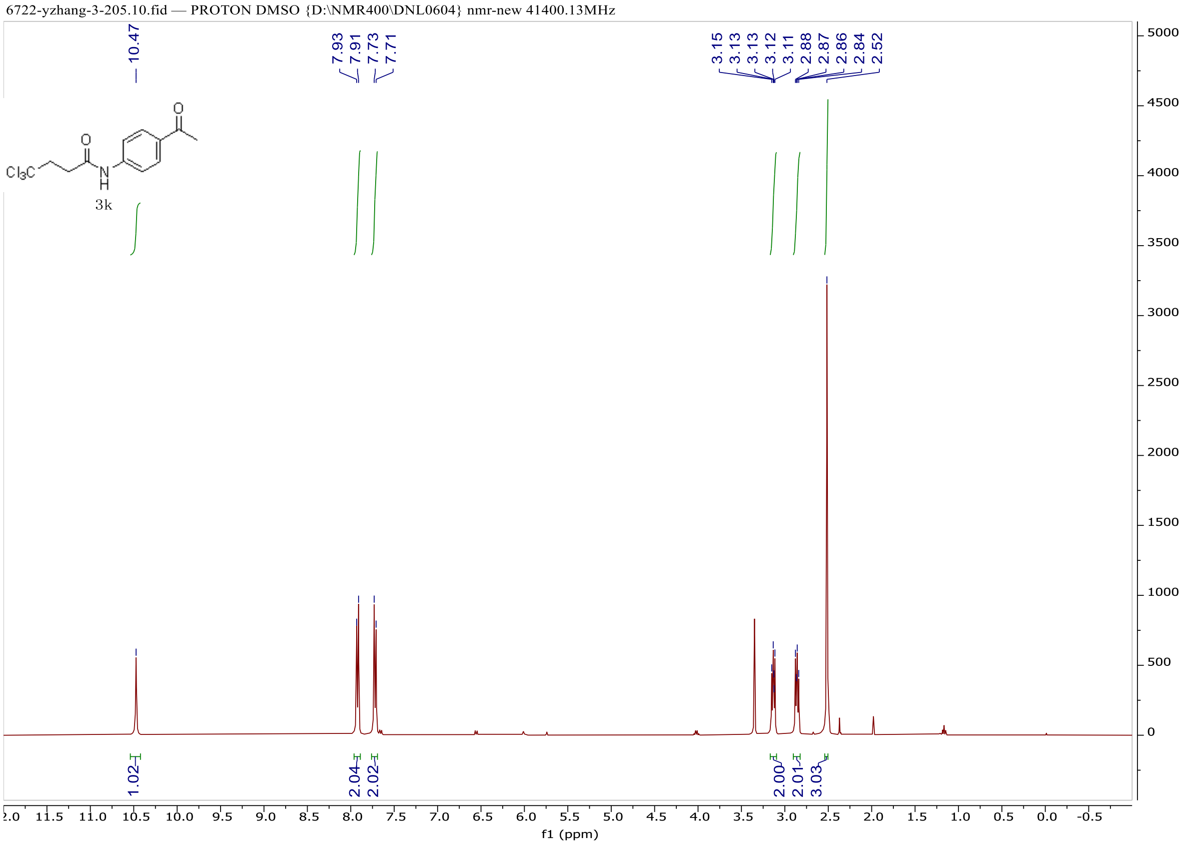
6722-yzhang-3-205.10.fid — PROTON DMSO {D:\NMR400\DNL0604} nmr-new 41400.13MHz
10.47
7.93
7.91
7.73
7.71
3.15
3.13
3.13
3.12
3.11
2.88
2.87
2.86
2.84
2.52
1.02
2.04
2.02
2.00
2.01
3.03
f1 (ppm)
3k

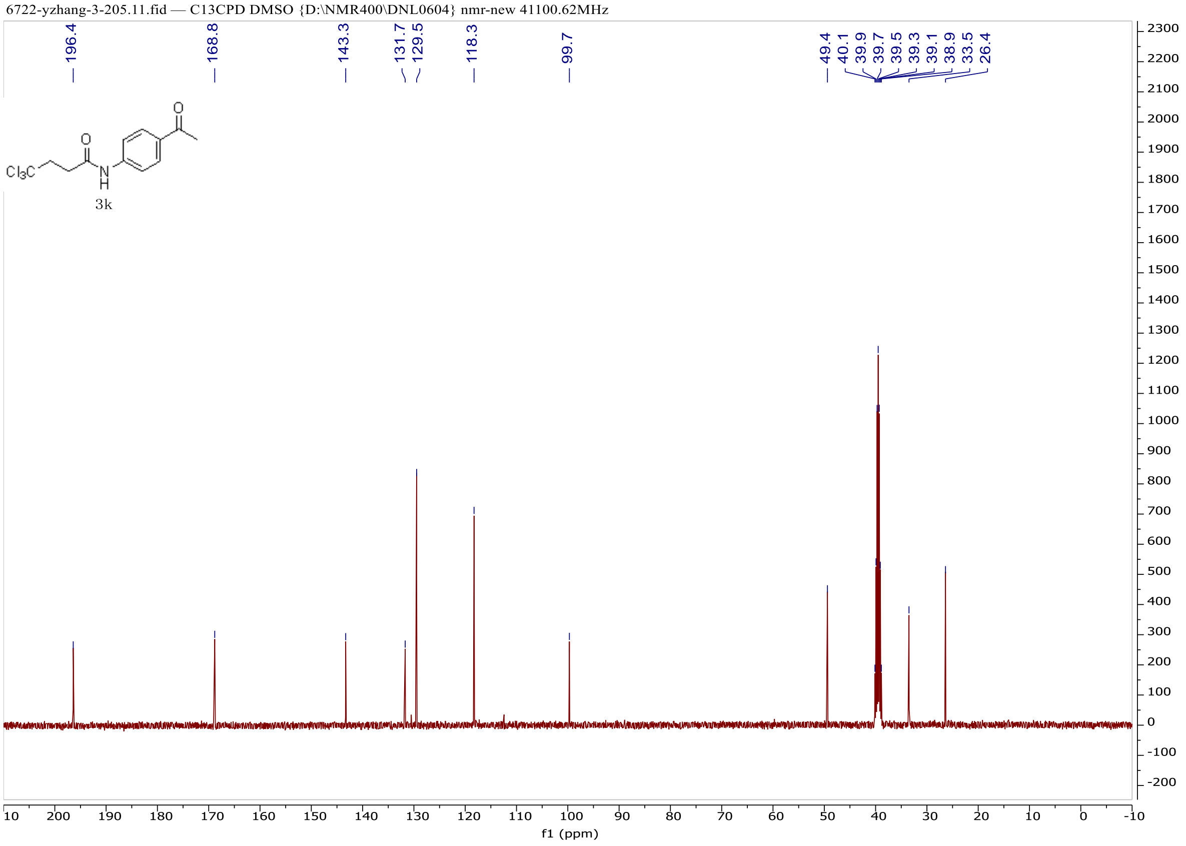
6722-yzhang-3-205.11.fid — C13CPD DMSO {D:\NMR400\DNL0604} nmr-new 41100.62MHz
196.4
168.8
143.3
131.7
129.5
118.3
99.7
49.4
40.1
39.9
39.7
39.5
39.3
39.1
38.9
33.5
26.4
f1 (ppm)
3k

3l

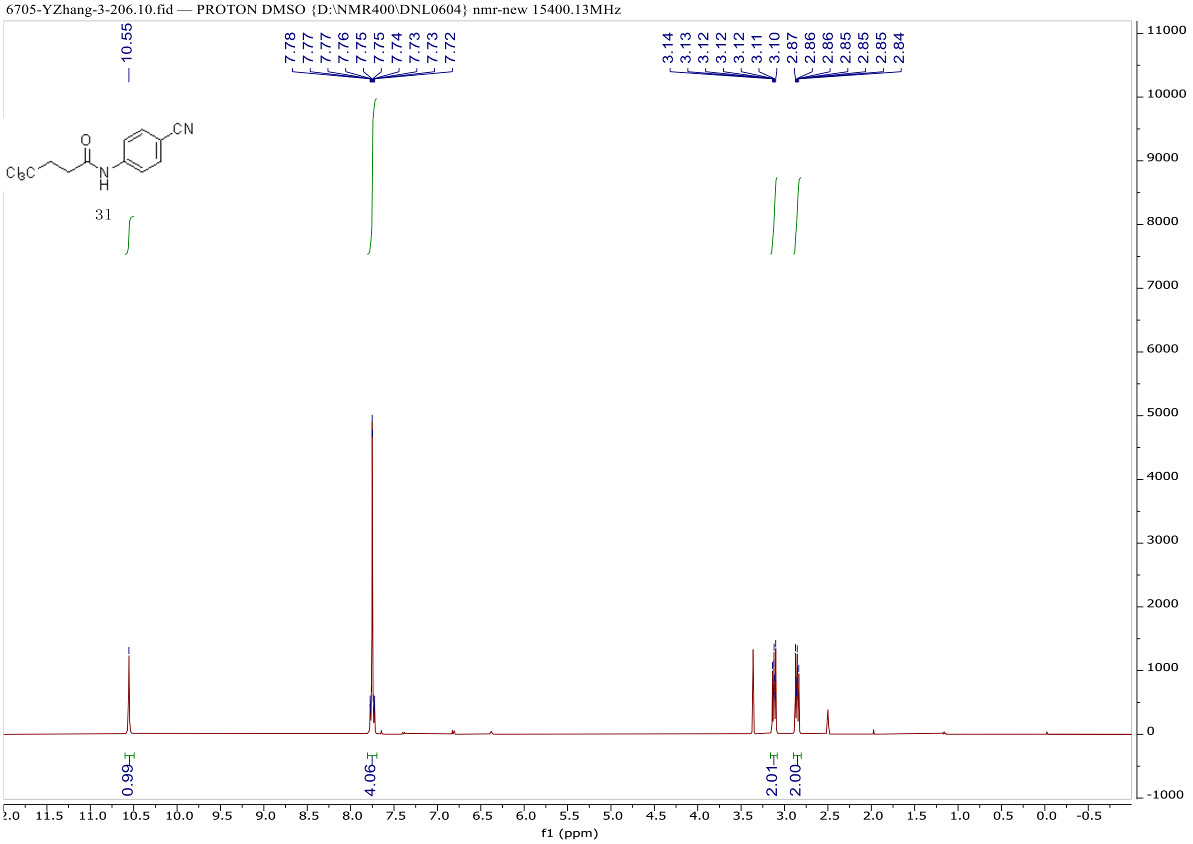
6705-YZhang-3-206.10.fid — PROTON DMSO {D:\NMR400\DNL0604} nmr-new 15400.13MHz
10.55
7.78 7.77 7.77 7.76 7.75 7.75 7.74 7.73 7.73 7.72
3.14 3.13 3.12 3.12 3.12 3.11 3.10 2.87 2.86 2.86 2.85 2.85 2.85 2.84
CN
O
Cl3C
N
H
3l
0.99
4.06
2.01
2.00
f1 (ppm)

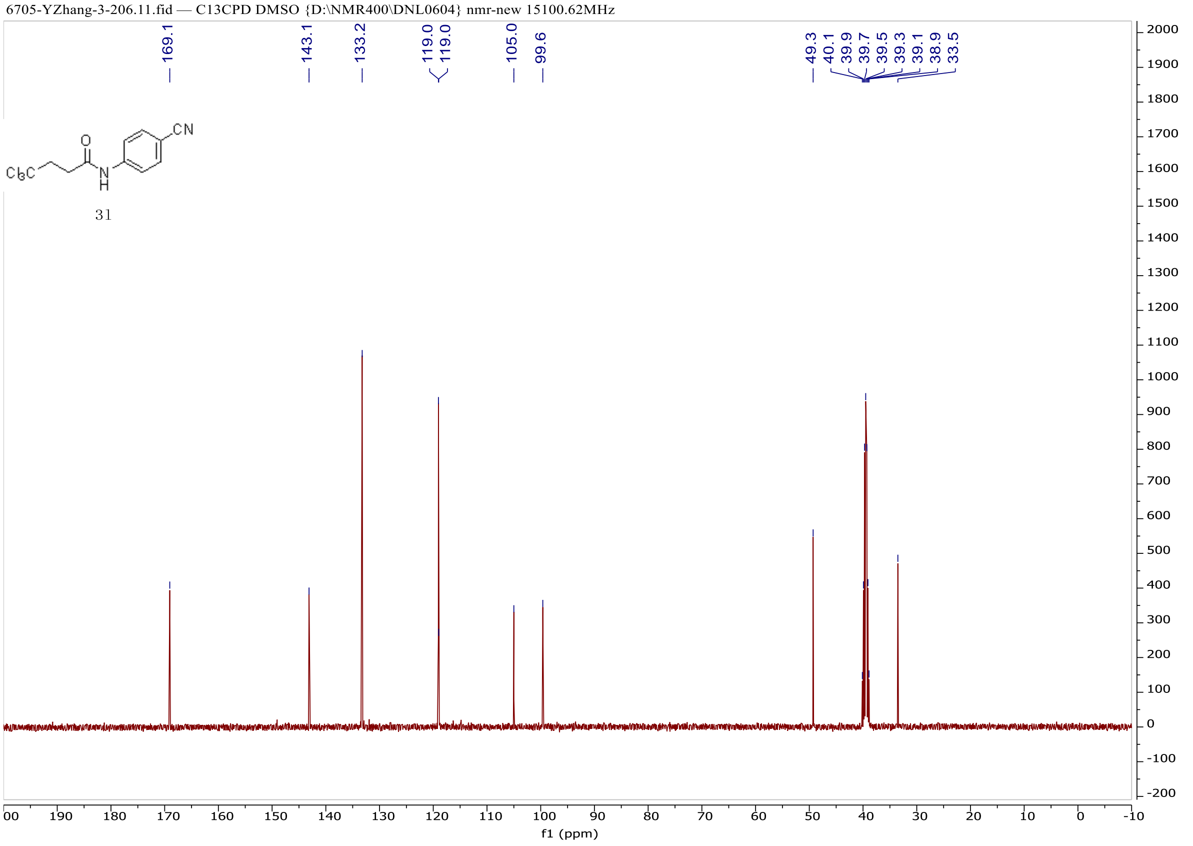
6705-YZhang-3-206.11.fid — C13CPD DMSO {D:\NMR400\DNL0604} nmr-new 15100.62MHz
169.1
143.1
133.2
119.0 119.0
105.0
99.6
49.3 40.1 39.9 39.7 39.5 39.3 39.1 38.9 33.5
CN
O
Cl3C
N
H
3l
f1 (ppm)

3m

6750-YZHANG-3-185-.10.fid — PROTON DMSO {D:\NMR400\DNL0604} nmr-new 17400.13MHz

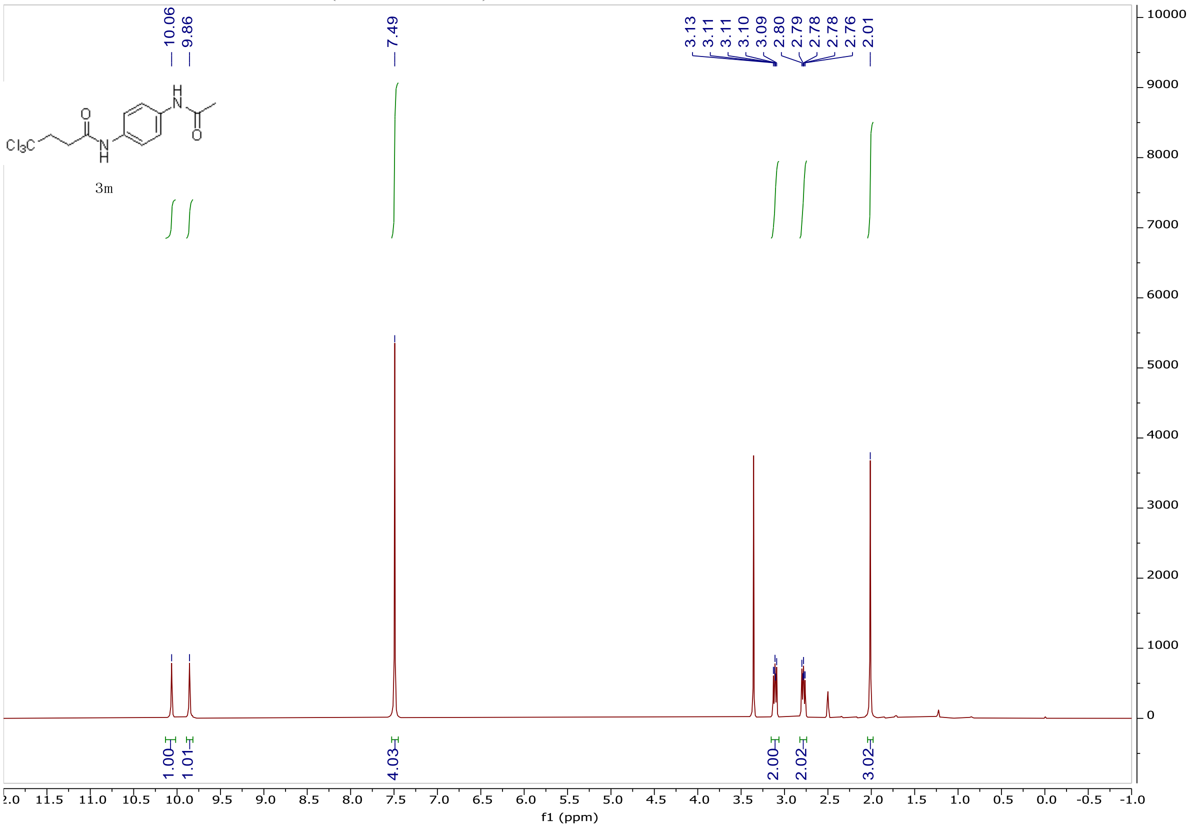

6750-YZHANG-3-185-.11.fid — C13CPD DMSO {D:\NMR400\DNL0604} nmr-new 17100.62MHz

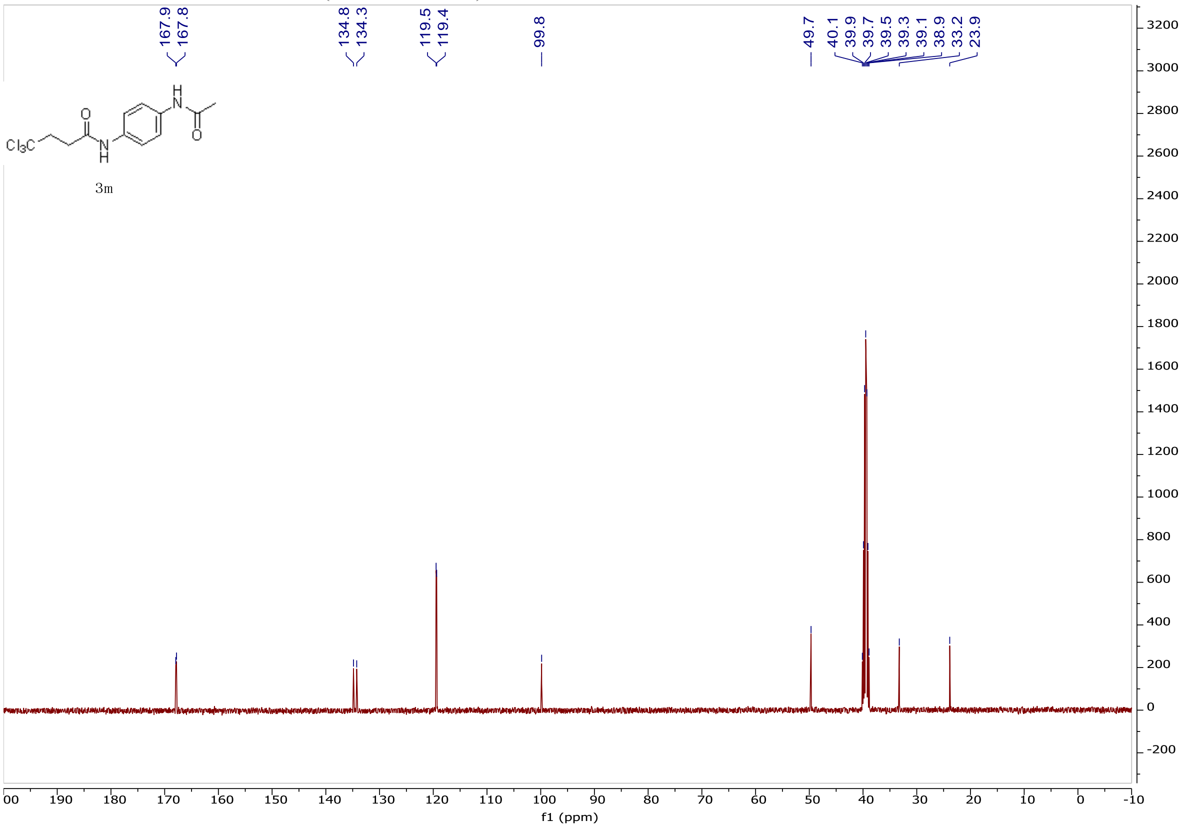

3n

7002-YZhang-3-258.10.fid — PROTON CDCl3 {D:\NMR400\DNL0604} nmr-new 46400.13MHz

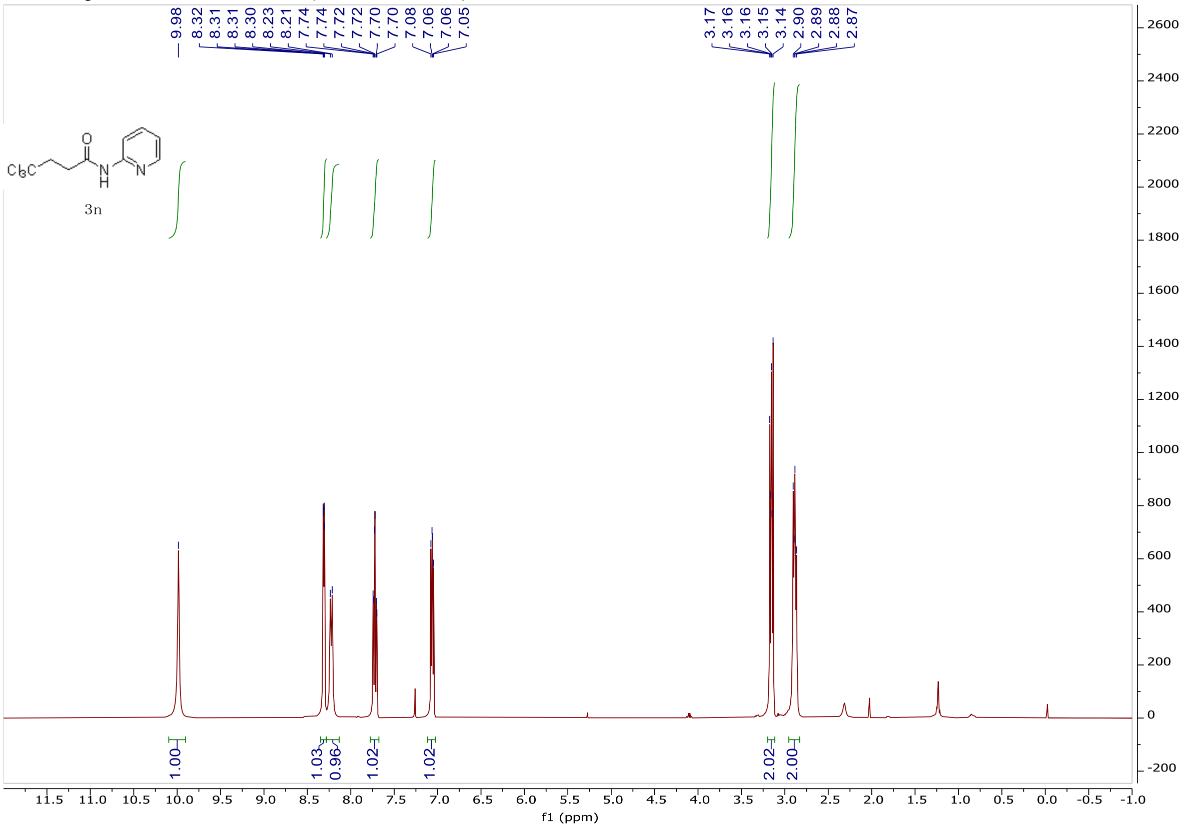

7002-YZhang-3-258.11.fid — C13CPD CDCl3 {D:\NMR400\DNL0604} nmr-new 46100.62MHz

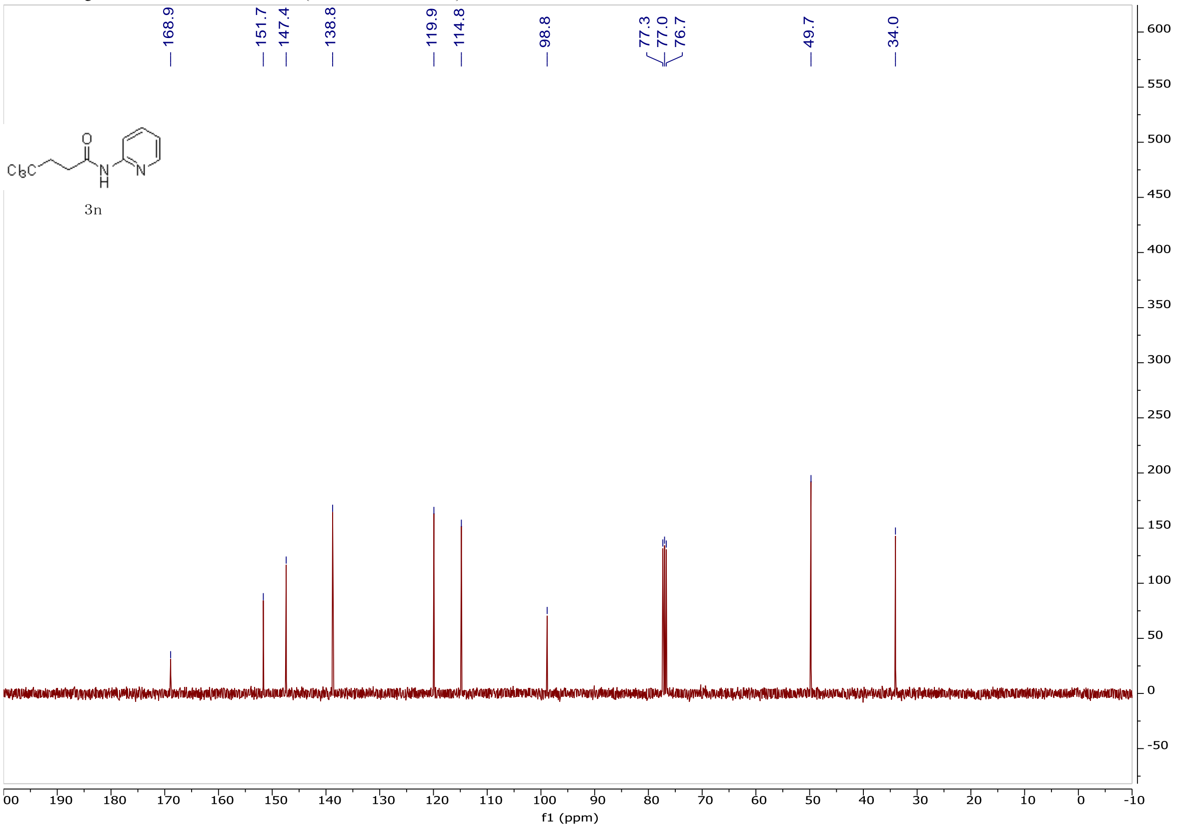

3o

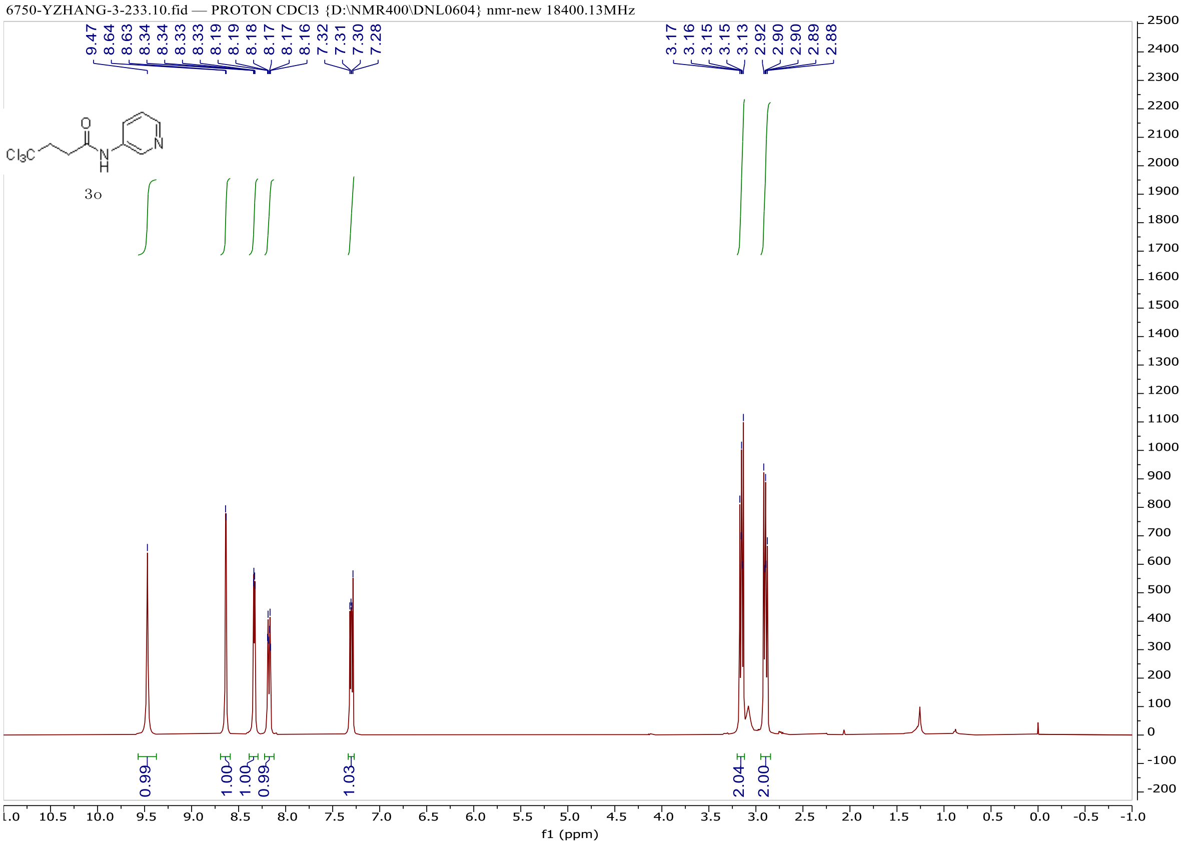
6750-YZHANG-3-233.10.fid — PROTON CDCl3 {D:\NMR400\DNL0604} nmr-new 18400.13MHz
9.47 8.64 8.63 8.34 8.34 8.33 8.33 8.19 8.18 8.17 8.17 8.16 7.32 7.31 7.30 7.28
3.17 3.16 3.15 3.15 3.13 2.92 2.90 2.90 2.89 2.88
3o
0.99 1.00 1.00 0.99 1.03 2.04 2.00
f1 (ppm)

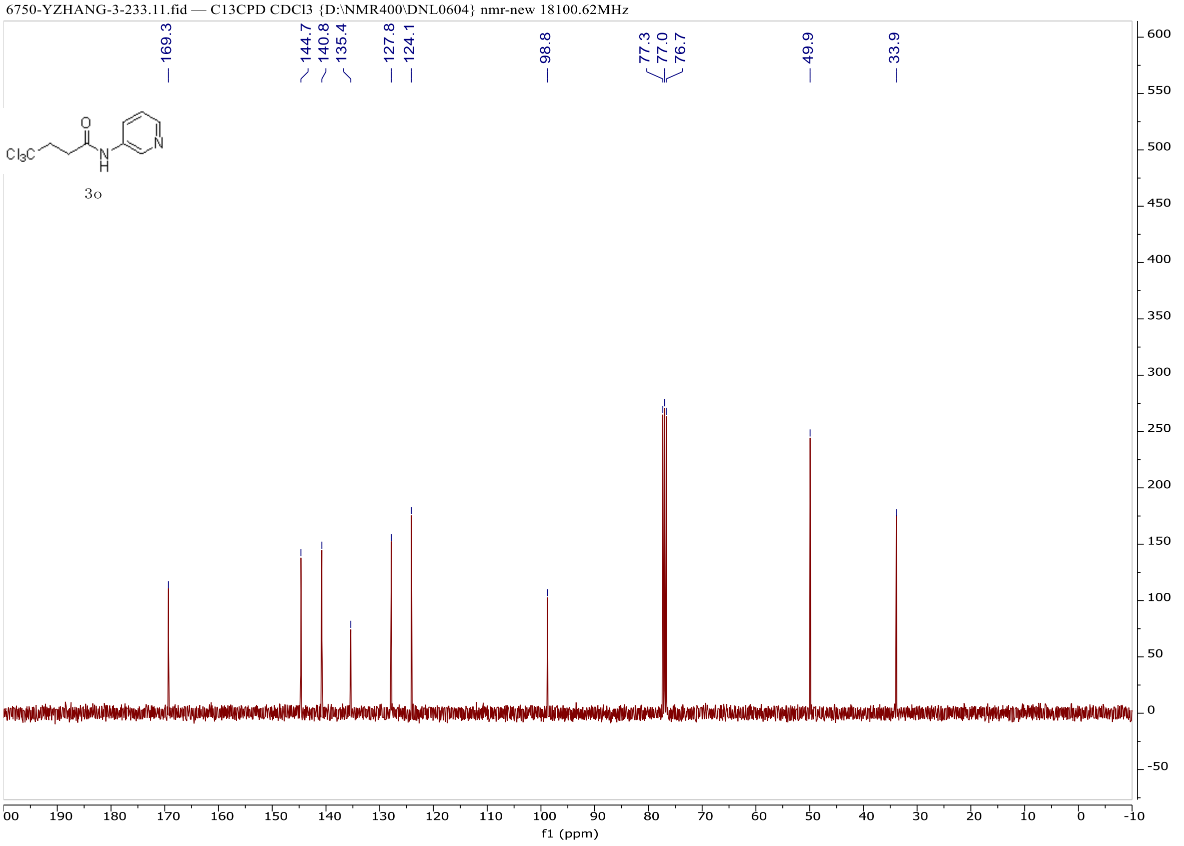
6750-YZHANG-3-233.11.fid — C13CPD CDCl3 {D:\NMR400\DNL0604} nmr-new 18100.62MHz
169.3 144.7 140.8 135.4 127.8 124.1 98.8 77.3 77.0 76.7 49.9 33.9
3o
f1 (ppm)

3p

6519-YZHANG-3-182.10.fid — PROTON CDCl3 {D:\NMR400\DNL0604} nmr-new 36400.13MHz

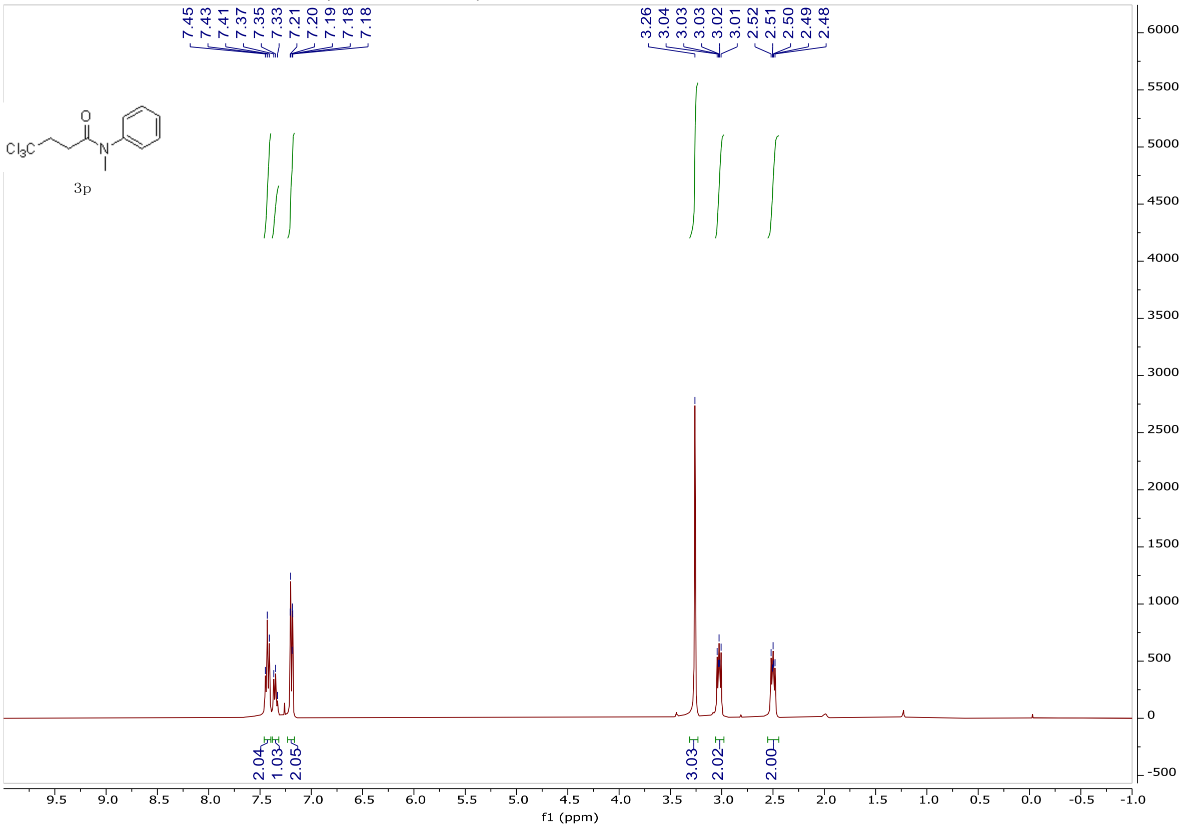

6519-YZHANG-3-182.11.fid — C13CPD CDCl3 {D:\NMR400\DNL0604} nmr-new 36100.62MHz

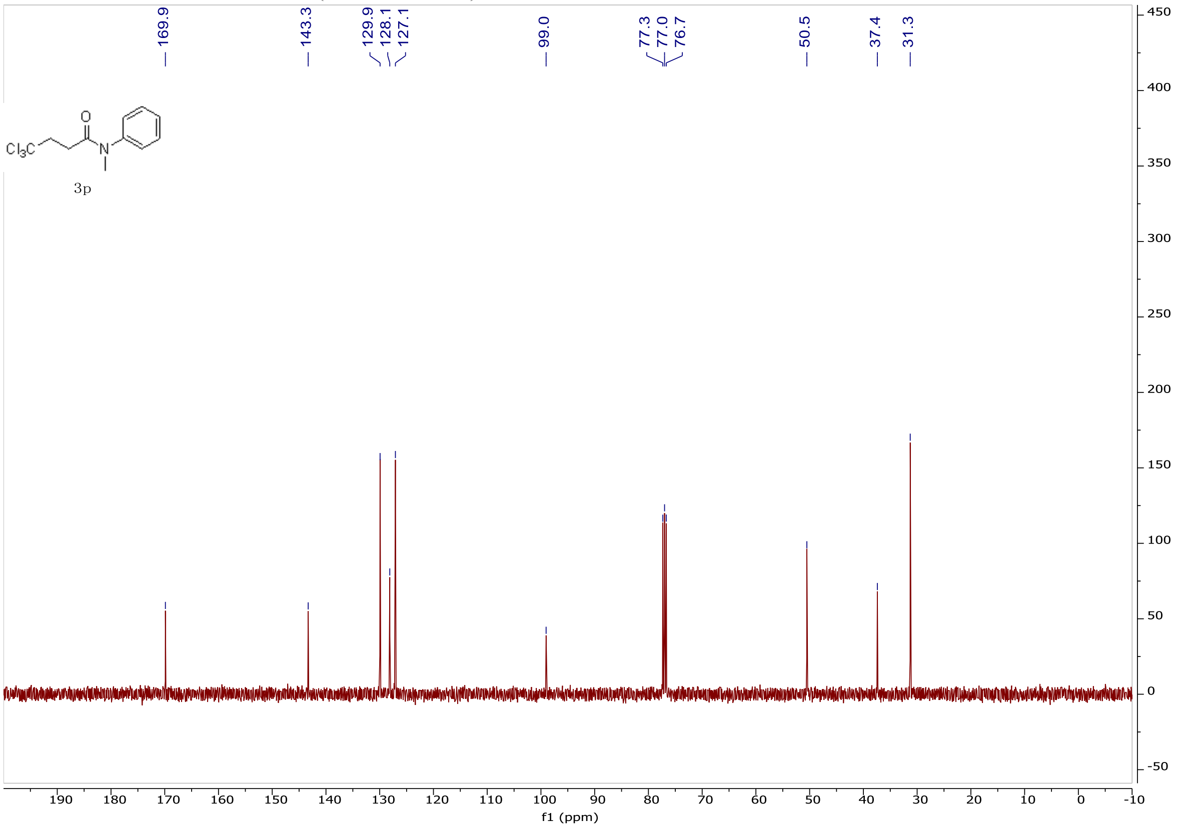

3q

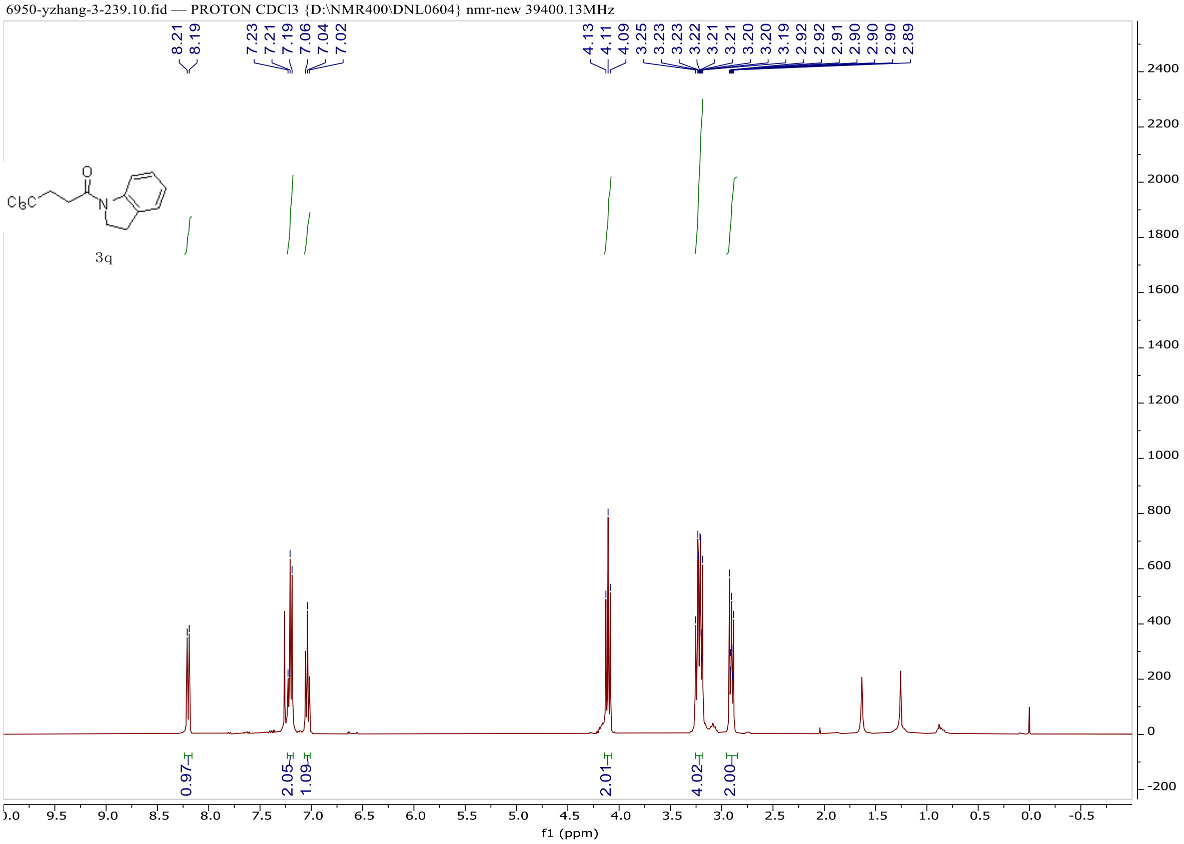
6950-yzhang-3-239.10.fid — PROTON CDCl3 {D:\NMR400\DNL0604} nmr-new 39400.13MHz
8.21
8.19
7.23
7.21
7.19
7.06
7.04
7.02
4.13
4.11
4.09
3.25
3.23
3.23
3.22
3.21
3.21
3.20
3.20
3.19
2.92
2.92
2.91
2.90
2.90
2.90
2.89
Cl3C
O
N
3q
0.97
2.05
1.09
2.01
4.02
2.00
f1 (ppm)

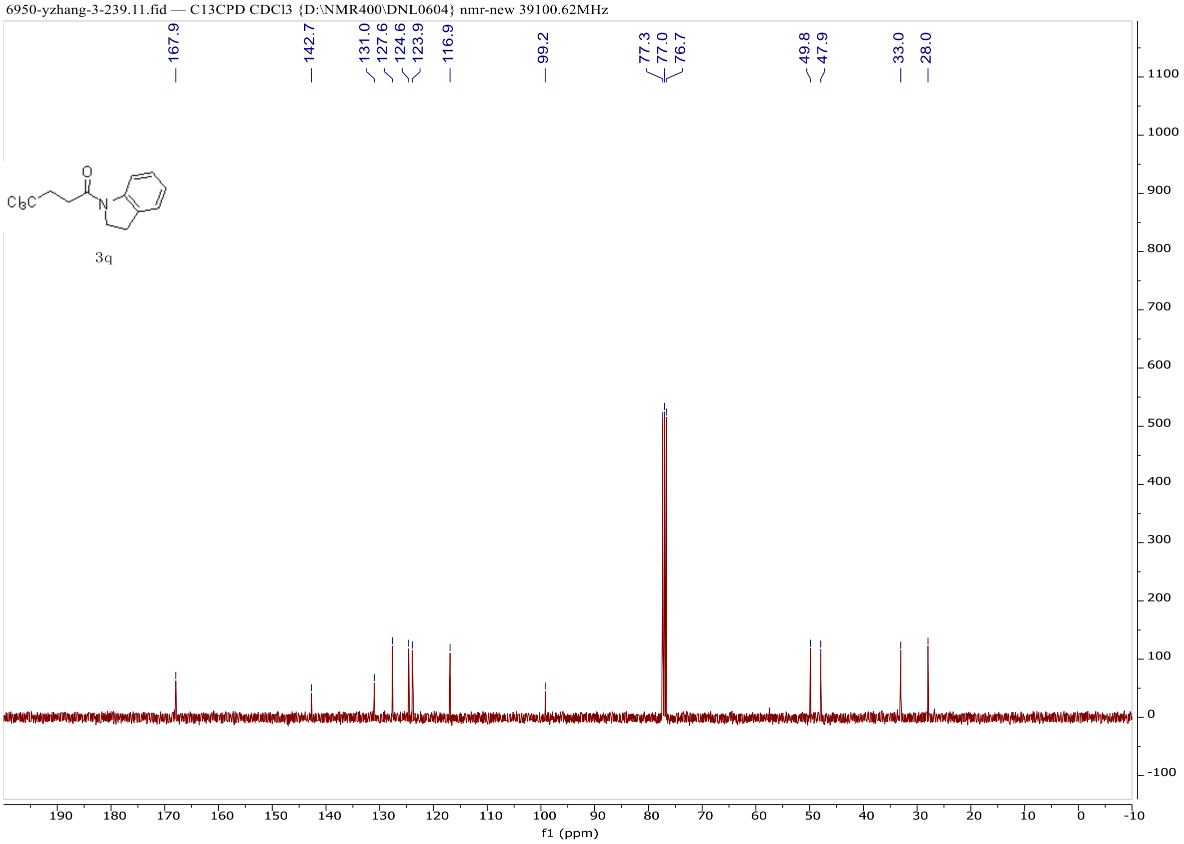
6950-yzhang-3-239.11.fid — C13CPD CDCl3 {D:\NMR400\DNL0604} nmr-new 39100.62MHz
167.9
142.7
131.0
127.6
124.6
123.9
116.9
99.2
77.3
77.0
76.7
49.8
47.9
33.0
28.0
Cl3C
O
N
3q
f1 (ppm)

3r

6256-YZhang-0-3-134.10.fid — PROTON CDCl3 {D:\NMR400\DNL0604} nmr-new 24400.13MHz

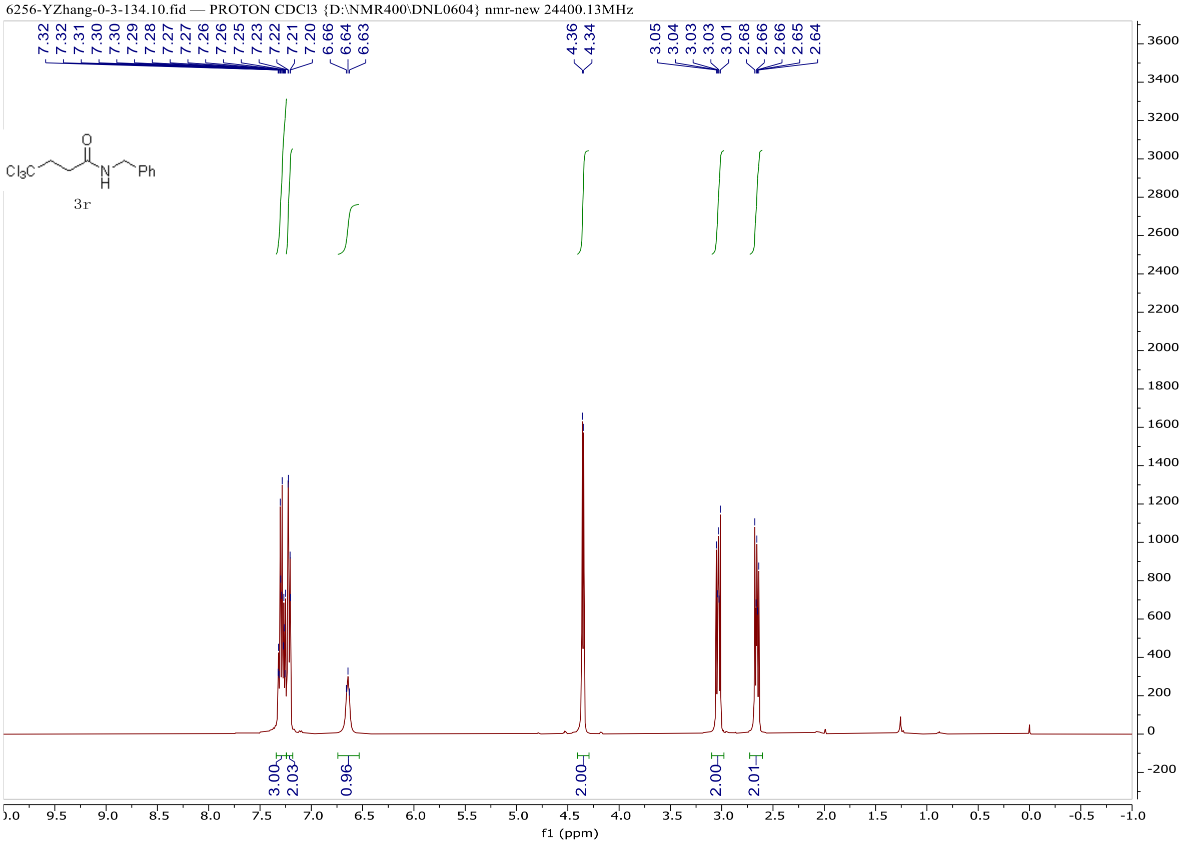

6256-YZhang-0-3-134.11.fid — C13CPD CDCl3 {D:\NMR400\DNL0604} nmr-new 24100.62MHz

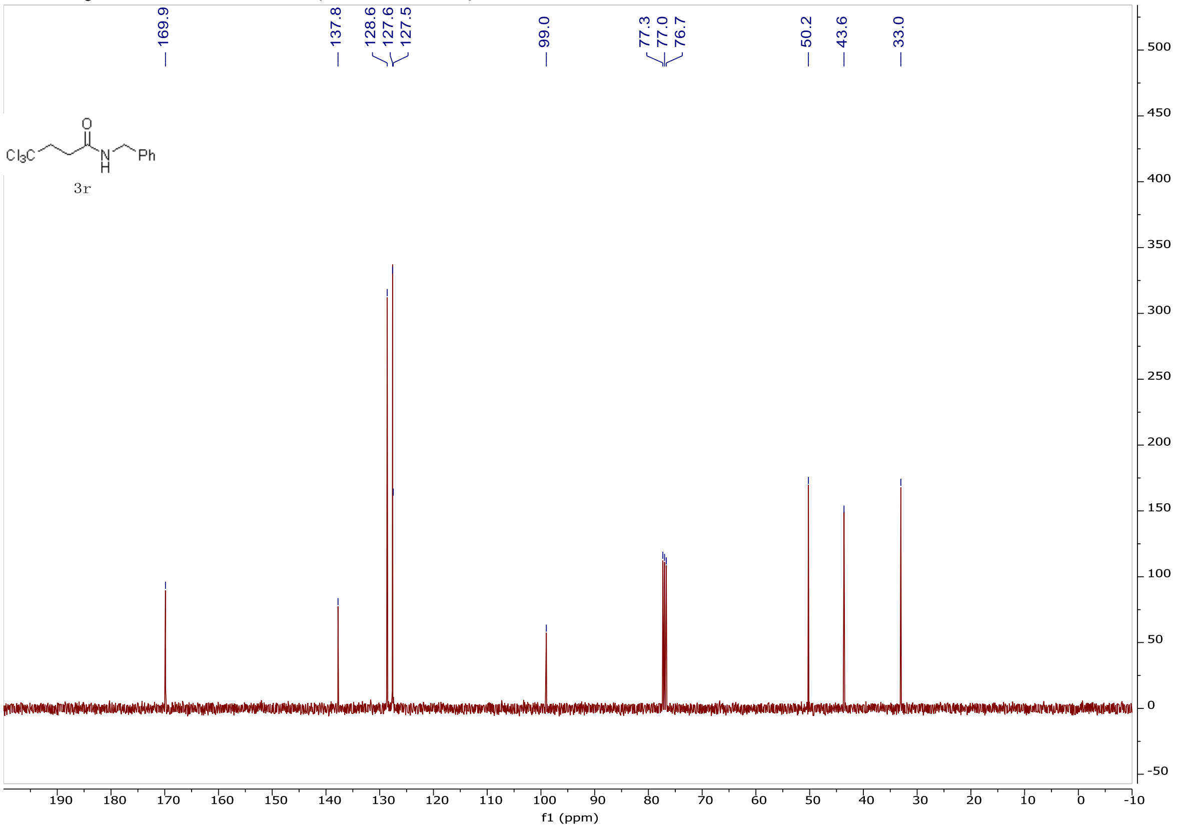

3s

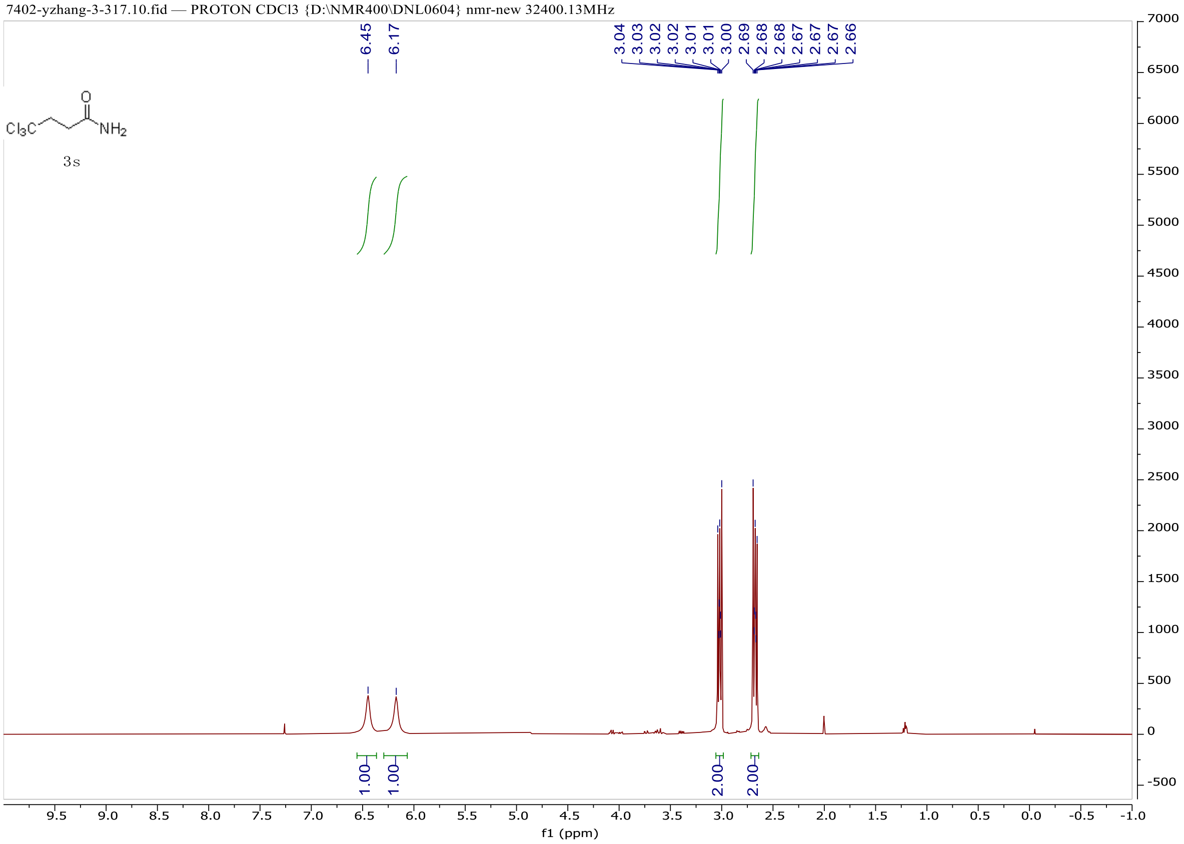
7402-yzhang-3-317.10.fid — PROTON CDCl3 {D:\NMR400\DNL0604} nmr-new 32400.13MHz
6.45
6.17
3.04
3.03
3.02
3.02
3.01
3.01
3.00
2.69
2.68
2.68
2.67
2.67
2.67
2.66
1.00
1.00
2.00
2.00
3s
f1 (ppm)

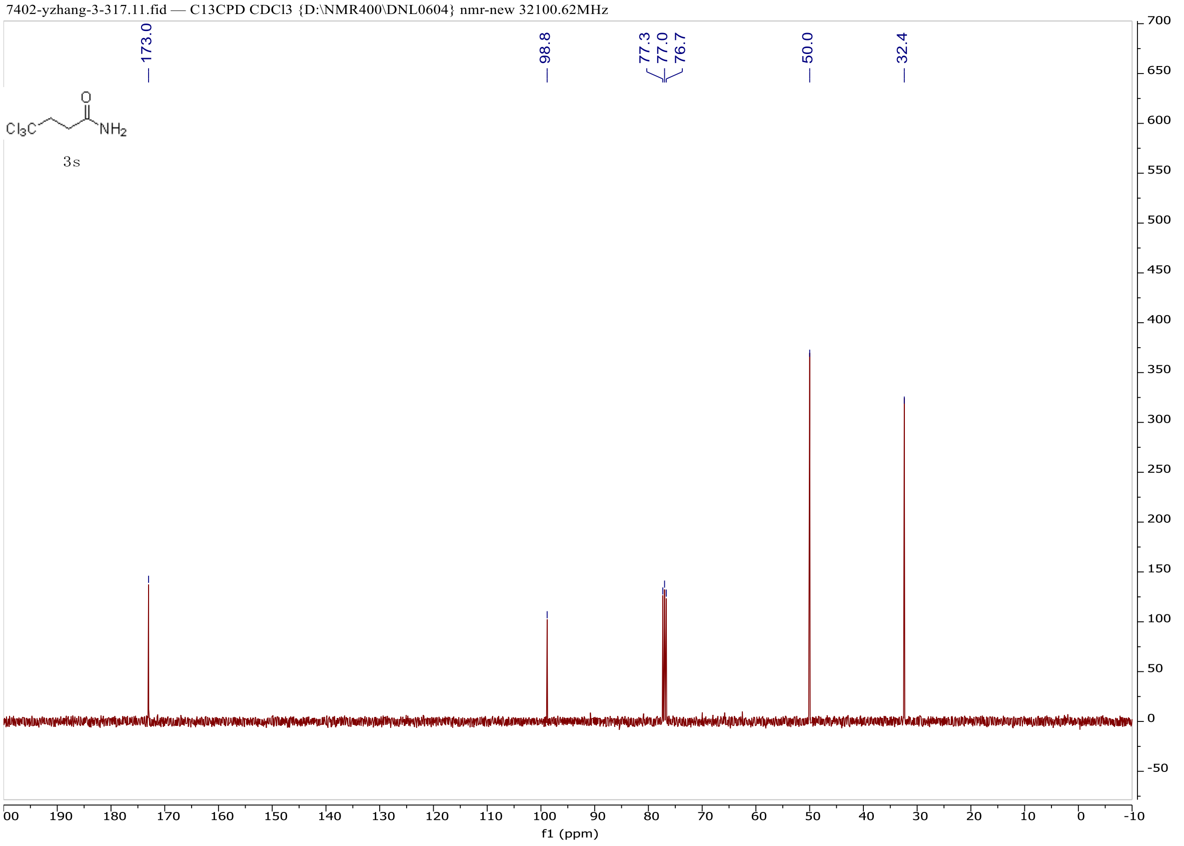
7402-yzhang-3-317.11.fid — C13CPD CDCl3 {D:\NMR400\DNL0604} nmr-new 32100.62MHz
173.0
98.8
77.3
77.0
76.7
50.0
32.4
3s
f1 (ppm)

4a

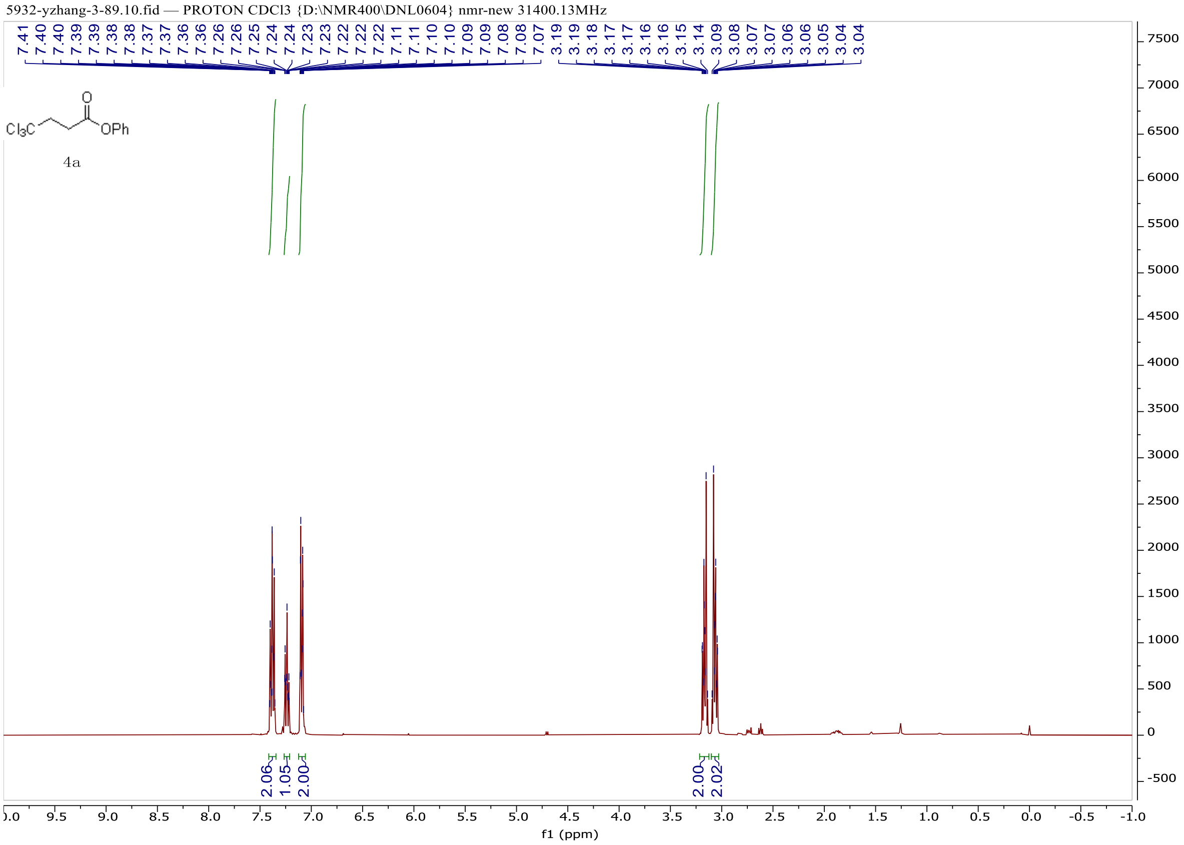
5932-yzhang-3-89.10.fid — PROTON CDCl3 {D:\NMR400\DNL0604} nmr-new 31400.13MHz
4a
f1 (ppm)

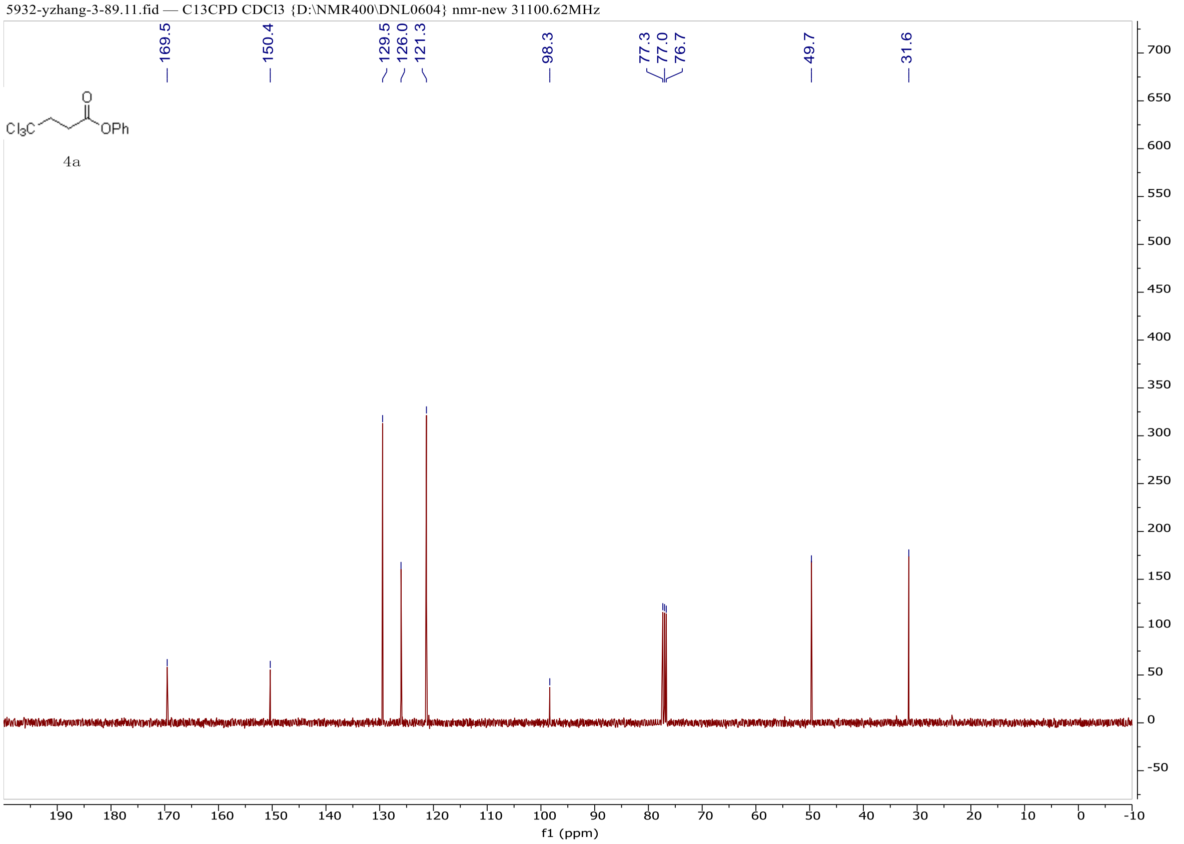
5932-yzhang-3-89.11.fid — C13CPD CDCl3 {D:\NMR400\DNL0604} nmr-new 31100.62MHz
169.5
150.4
129.5
126.0
121.3
98.3
77.3
77.0
76.7
49.7
31.6
4a
f1 (ppm)

4b

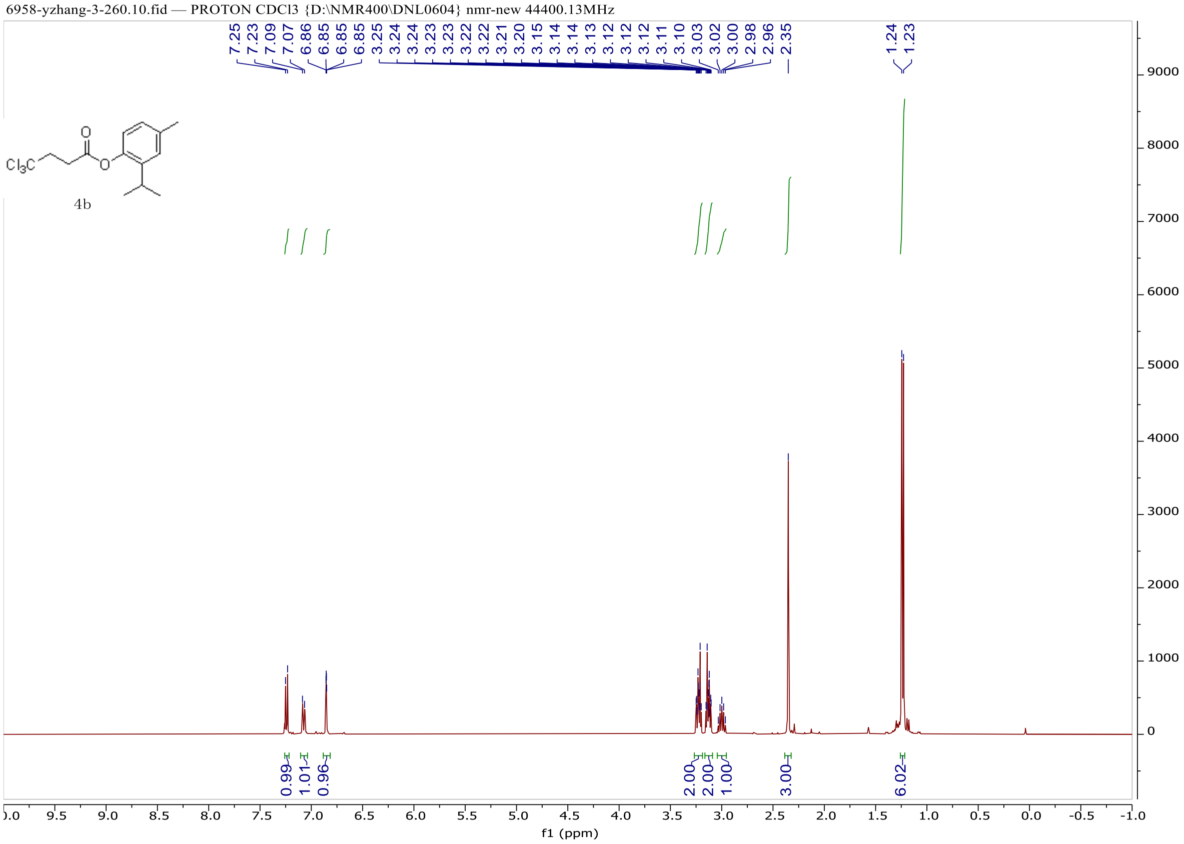
6958-yzhang-3-260.10.fid — PROTON CDCl3 {D:\NMR400\DNL0604} nmr-new 44400.13MHz
7.25 7.23 7.09 7.07 6.86 6.85 6.85 6.85 3.25 3.24 3.24 3.23 3.23 3.22 3.22 3.21 3.20 3.15 3.14 3.14 3.13 3.12 3.12 3.12 3.11 3.10 3.03 3.02 3.00 2.98 2.96 2.35 1.24 1.23
Cl3C
O
O
4b
0.99 1.01 0.96 2.00 2.00 1.00 3.00 6.02
f1 (ppm)

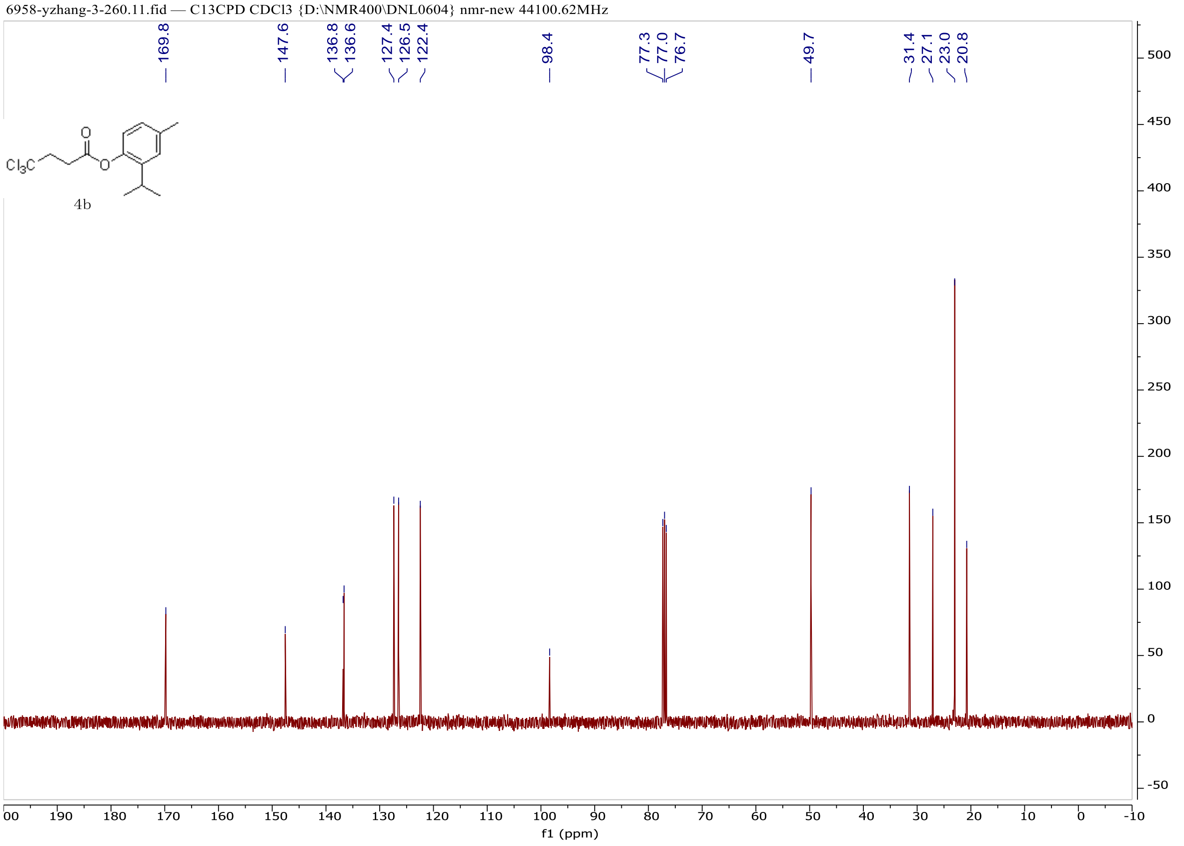
6958-yzhang-3-260.11.fid — C13CPD CDCl3 {D:\NMR400\DNL0604} nmr-new 44100.62MHz
169.8 147.6 136.8 136.6 127.4 126.5 122.4 98.4 77.3 77.0 76.7 49.7 31.4 27.1 23.0 20.8
Cl3C
O
O
4b
f1 (ppm)

4c

6705-YZhang-3-179b.10.fid — PROTON CDCl3 {D:\NMR400\DNL0604} nmr-new 14400.13MHz

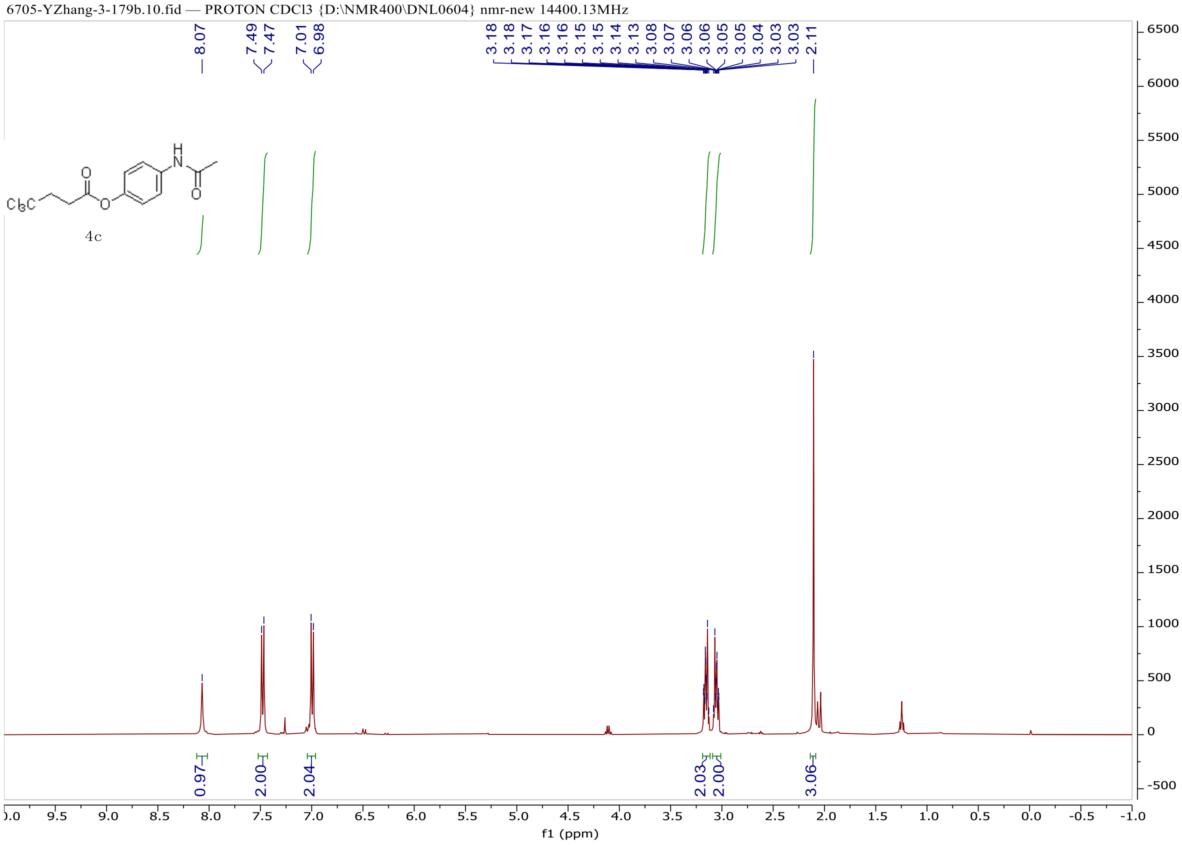

6705-YZhang-3-179b.11.fid — C13CPD CDCl3 {D:\NMR400\DNL0604} nmr-new 14100.62MHz

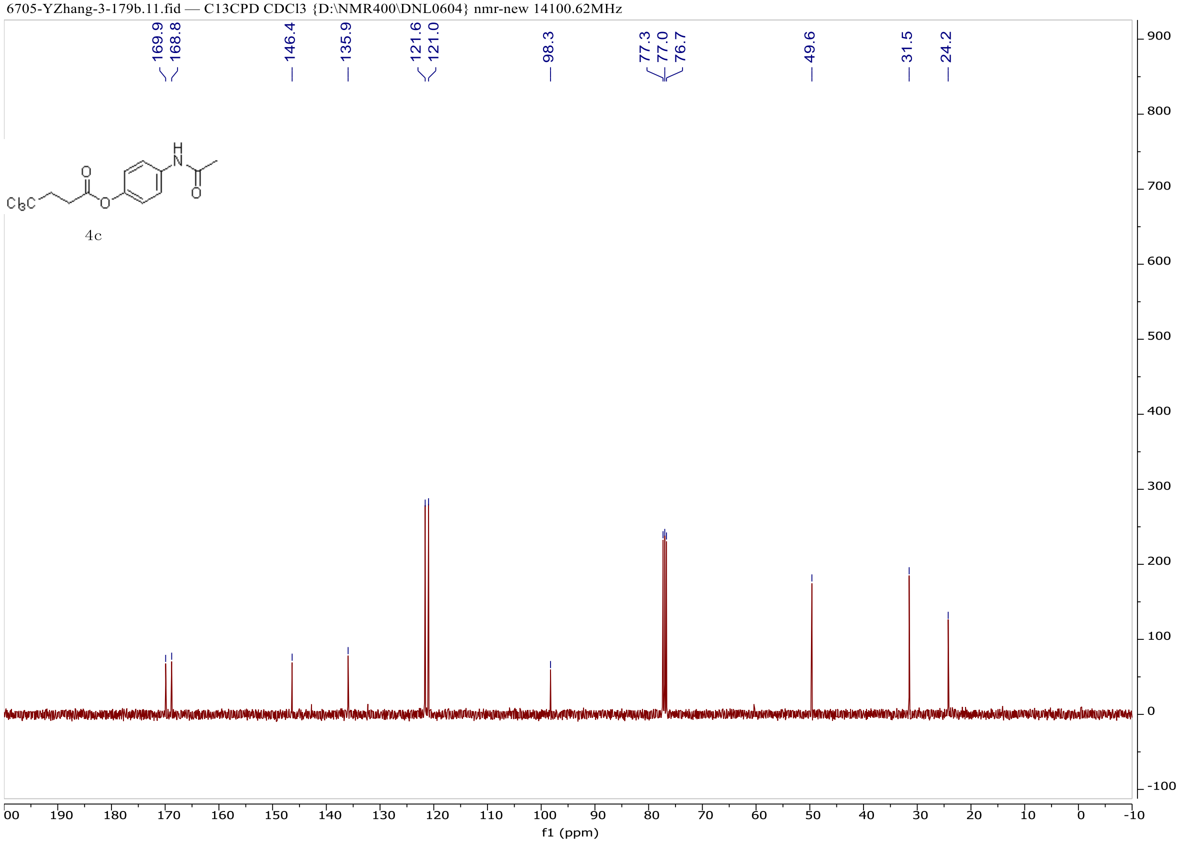

4d

6892-yZhang-3-254.10.fid — PROTON DMSO {D:\NMR400\DNL0604} nmr-new 41400.13MHz

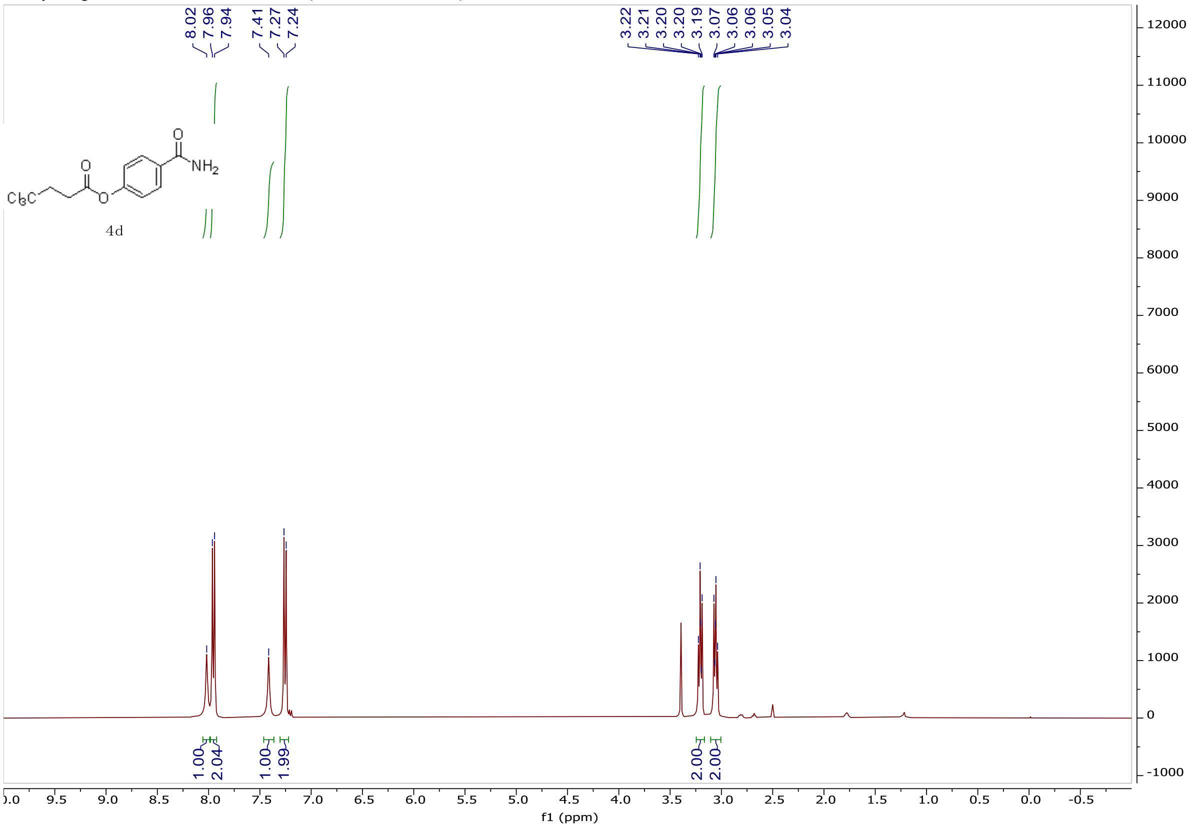

6892-yZhang-3-254.11.fid — C13CPD DMSO {D:\NMR400\DNL0604} nmr-new 41100.62MHz

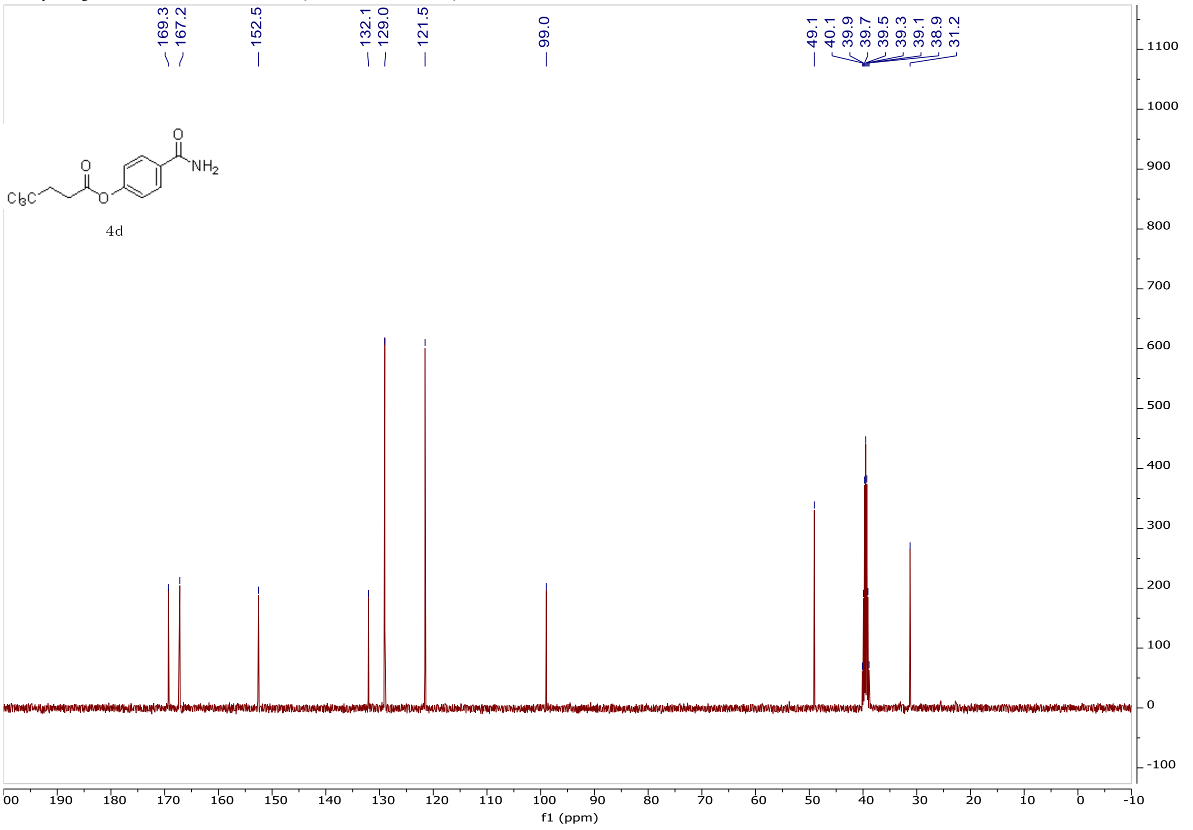

4e

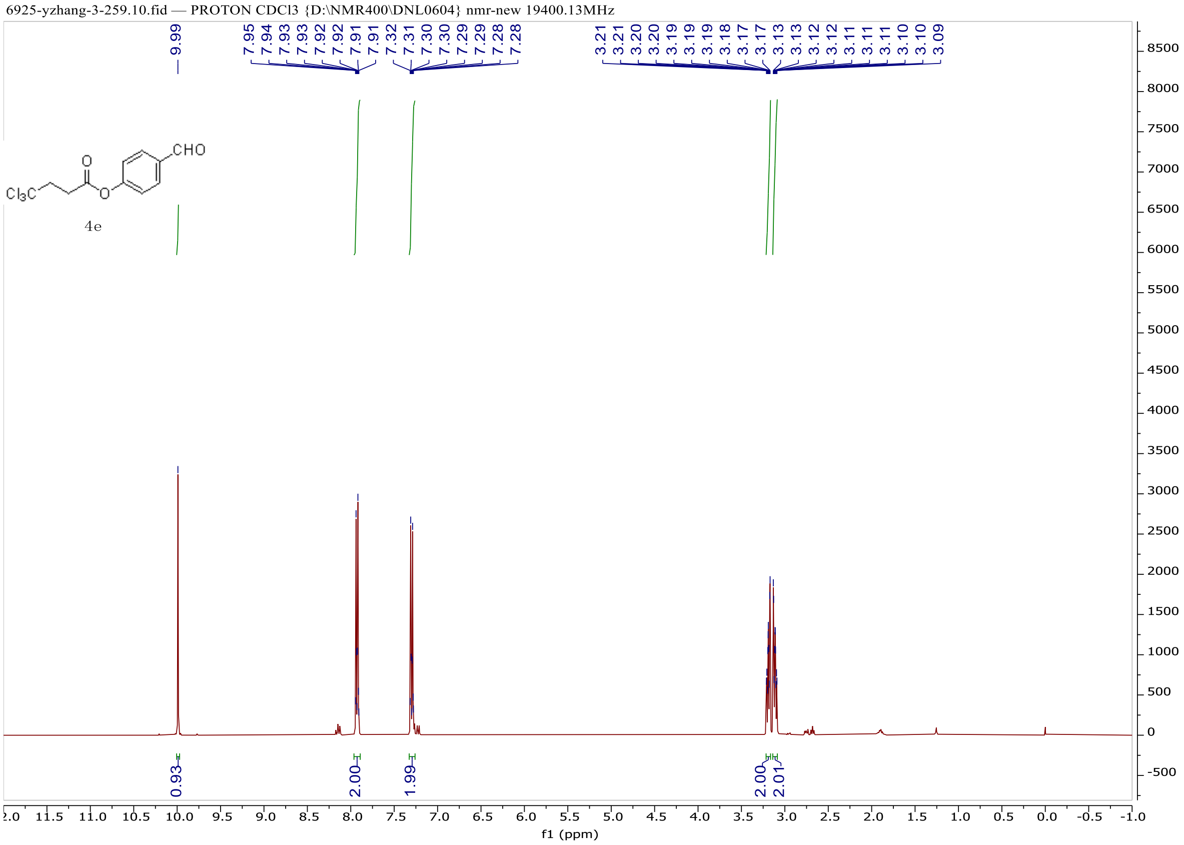
6925-yzhang-3-259.10.fid — PROTON CDCl3 {D:\NMR400\DNL0604} nmr-new 19400.13MHz
9.99
7.95 7.94 7.93 7.93 7.92 7.92 7.91 7.91 7.32 7.31 7.30 7.30 7.29 7.29 7.28 7.28
3.21 3.21 3.20 3.20 3.19 3.19 3.19 3.18 3.17 3.17 3.13 3.13 3.12 3.12 3.11 3.11 3.11 3.10 3.10 3.09
CHO
O
Cl3C
O
4e
0.93
2.00
1.99
2.00
2.01
f1 (ppm)

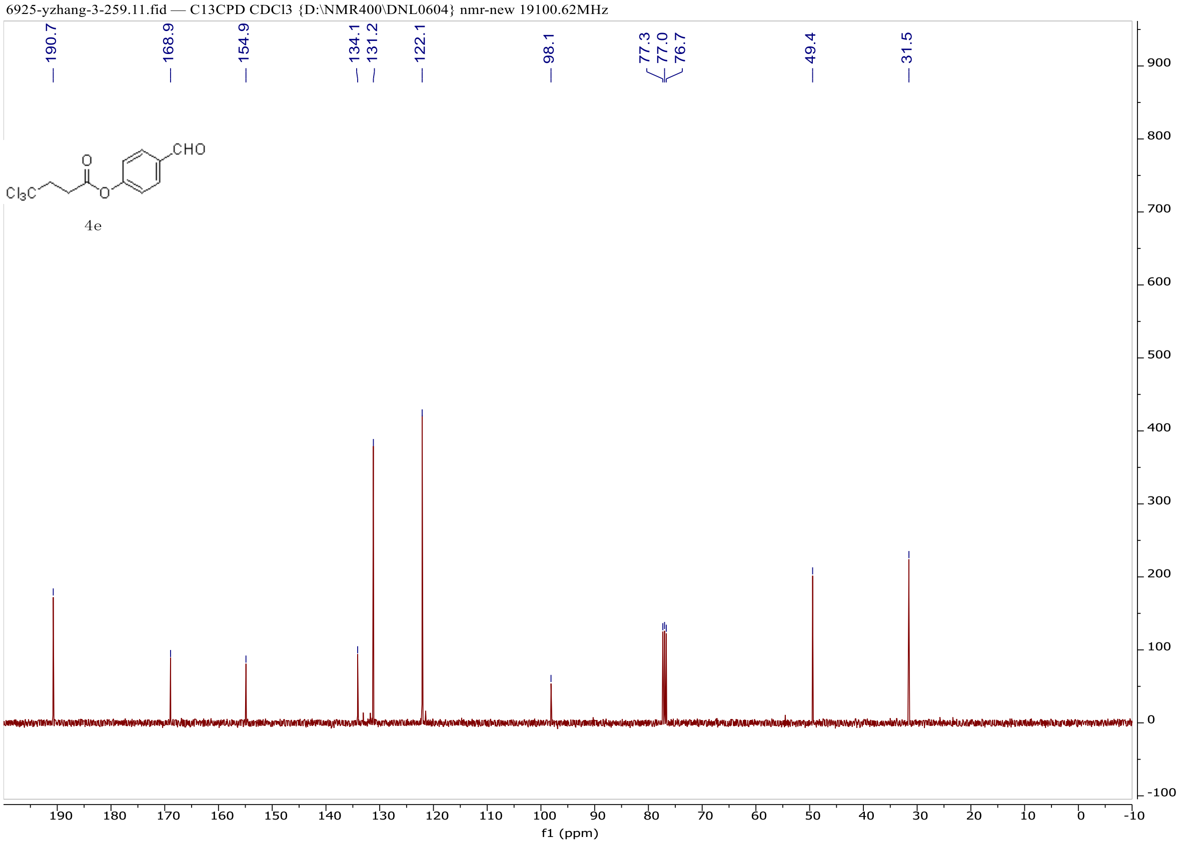
6925-yzhang-3-259.11.fid — C13CPD CDCl3 {D:\NMR400\DNL0604} nmr-new 19100.62MHz
190.7
168.9
154.9
134.1
131.2
122.1
98.1
77.3
77.0
76.7
49.4
31.5
CHO
O
Cl3C
O
4e
f1 (ppm)

4f

7124-YZHANG-3-299.10.fid — PROTON CDCl3 {D:\NMR400\DNL0604} nmr-new 34400.13MHz

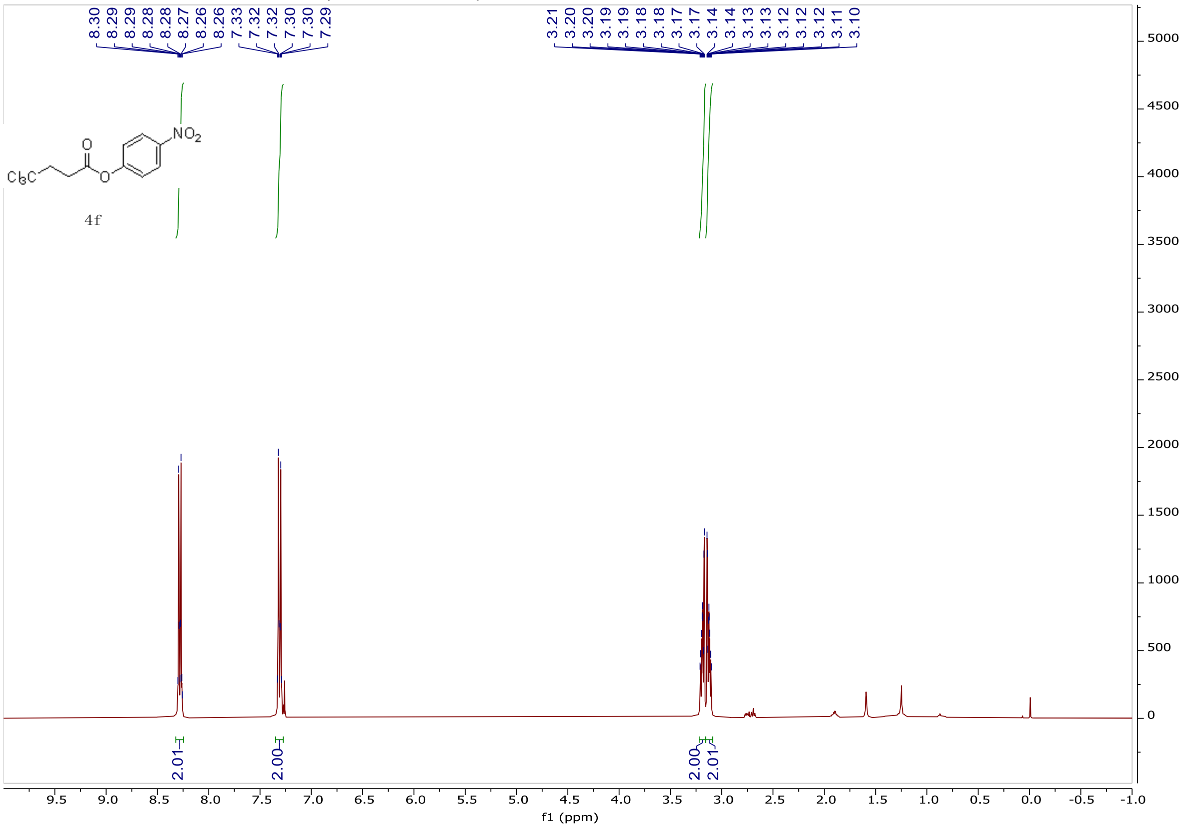

7124-YZHANG-3-299.11.fid — C13CPD CDCl3 {D:\NMR400\DNL0604} nmr-new 34100.62MHz

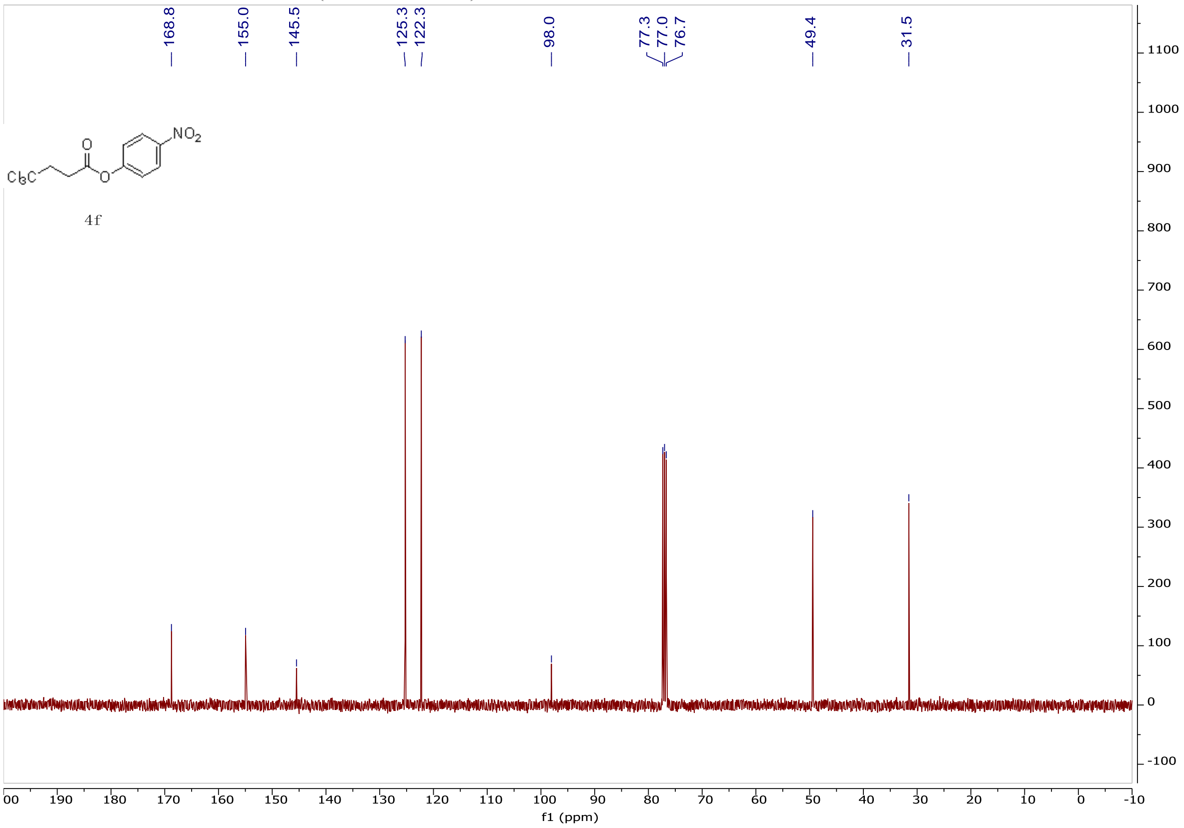

4g

6958-yzhang-3-263.10.fid — PROTON CDCl3 {D:\NMR400\DNL0604} nmr-new 46400.13MHz

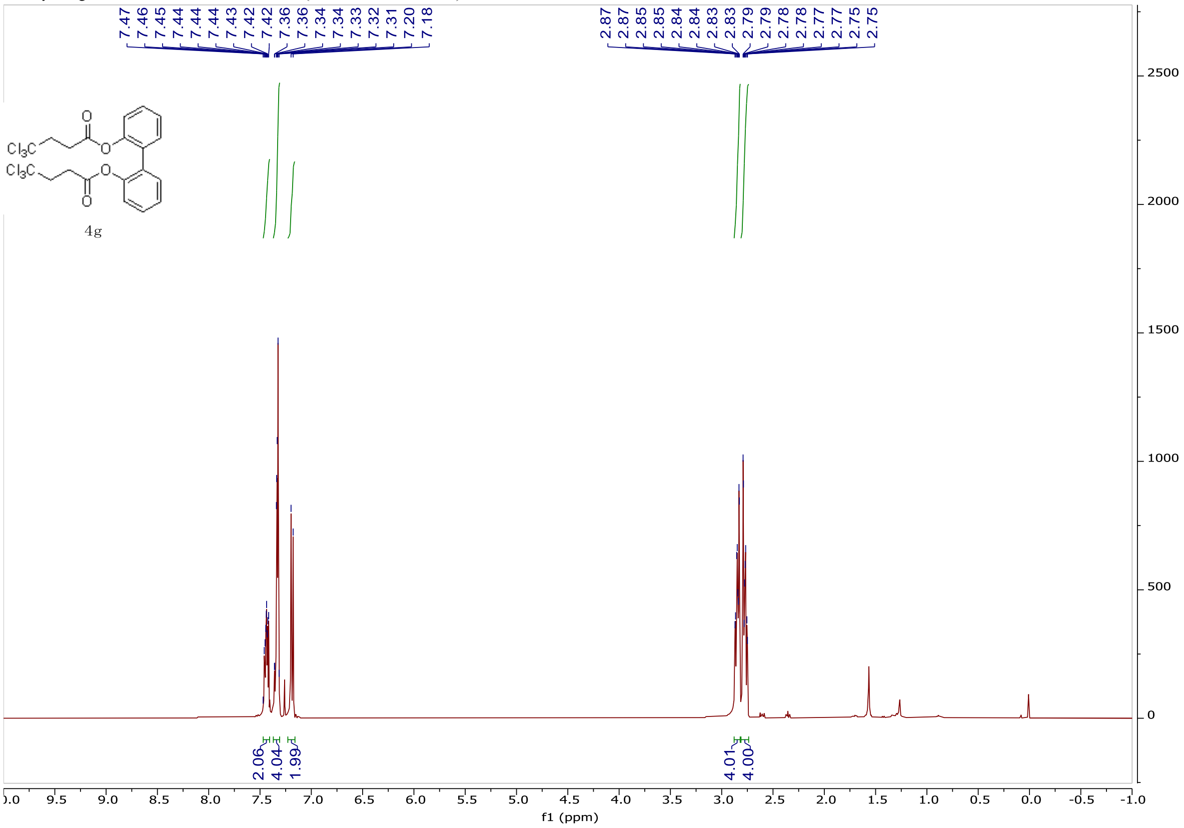

6958-yzhang-3-263.11.fid — C13CPD CDCl3 {D:\NMR400\DNL0604} nmr-new 46100.62MHz

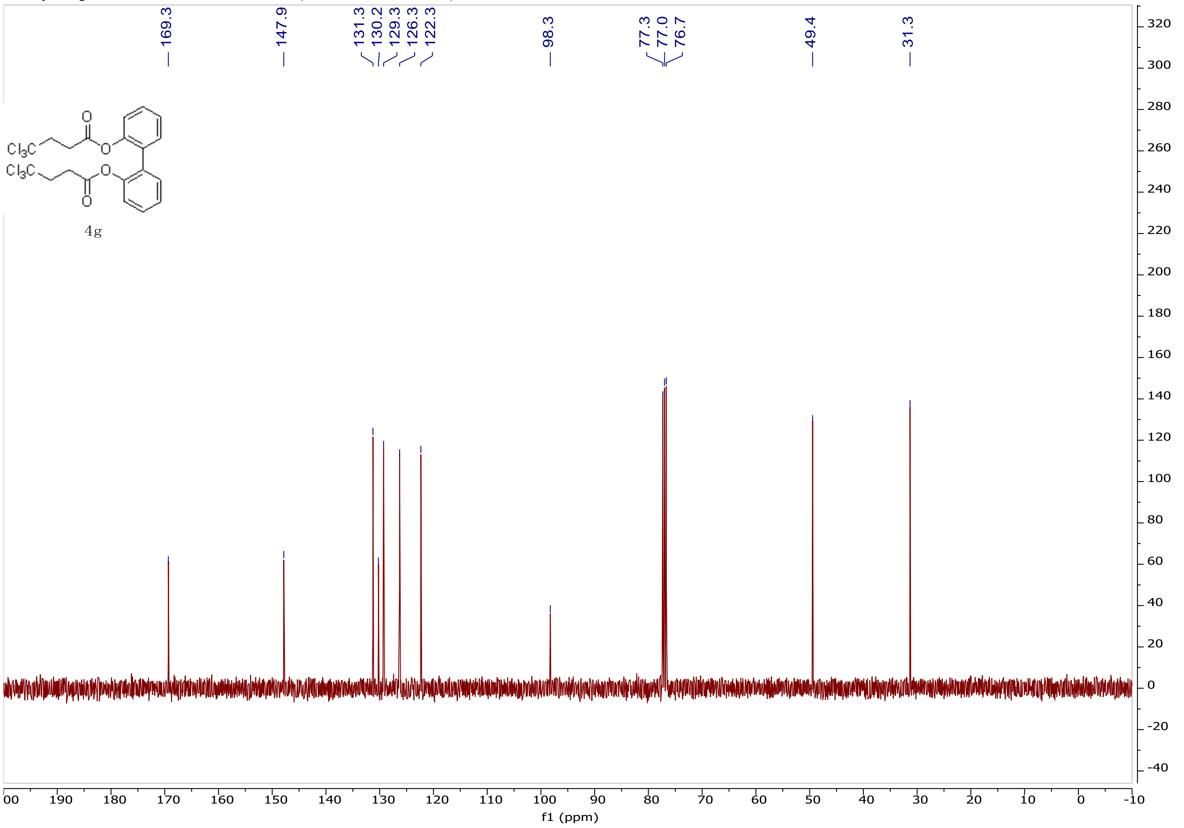

4h

6285-YZhang-3-130.10.fid — PROTON CDCl3 {D:\NMR400\DNL0604} nmr-new 6400.13MHz

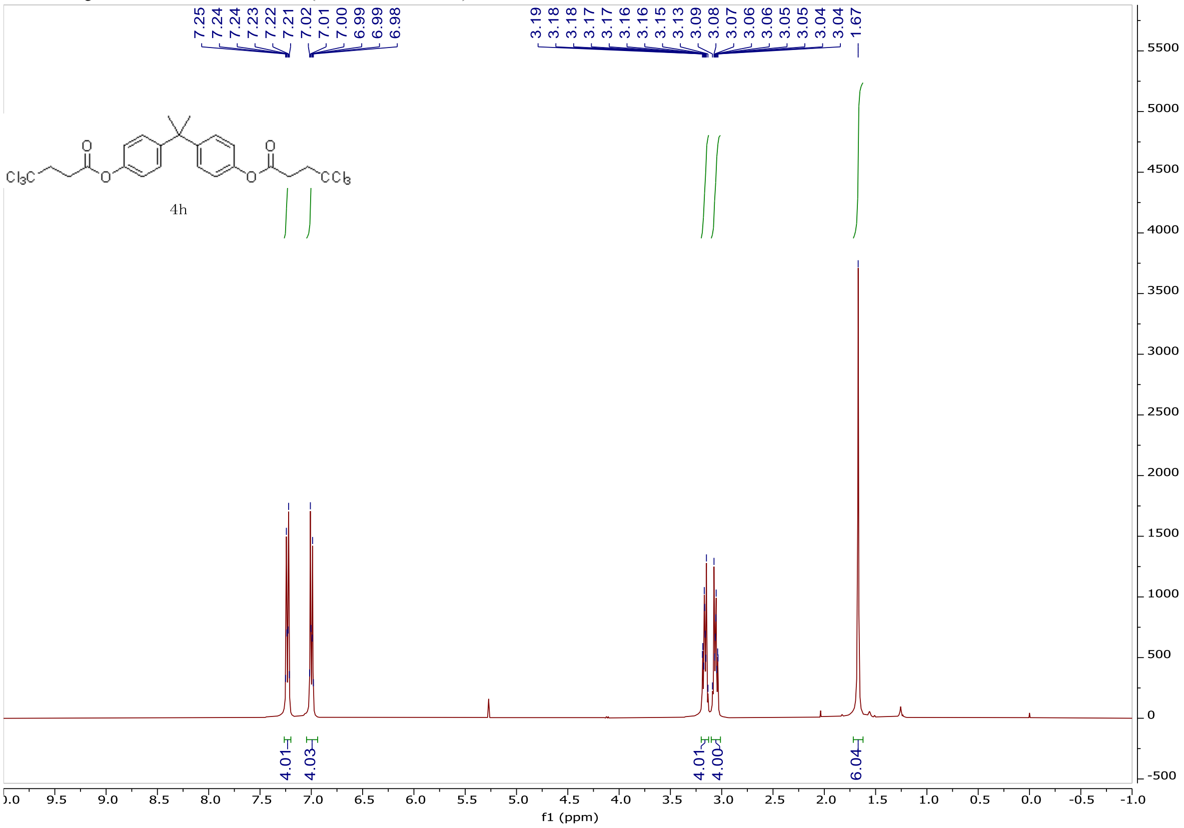

6285-YZhang-3-130.11.fid — C13CPD CDCl3 {D:\NMR400\DNL0604} nmr-new 6100.62MHz

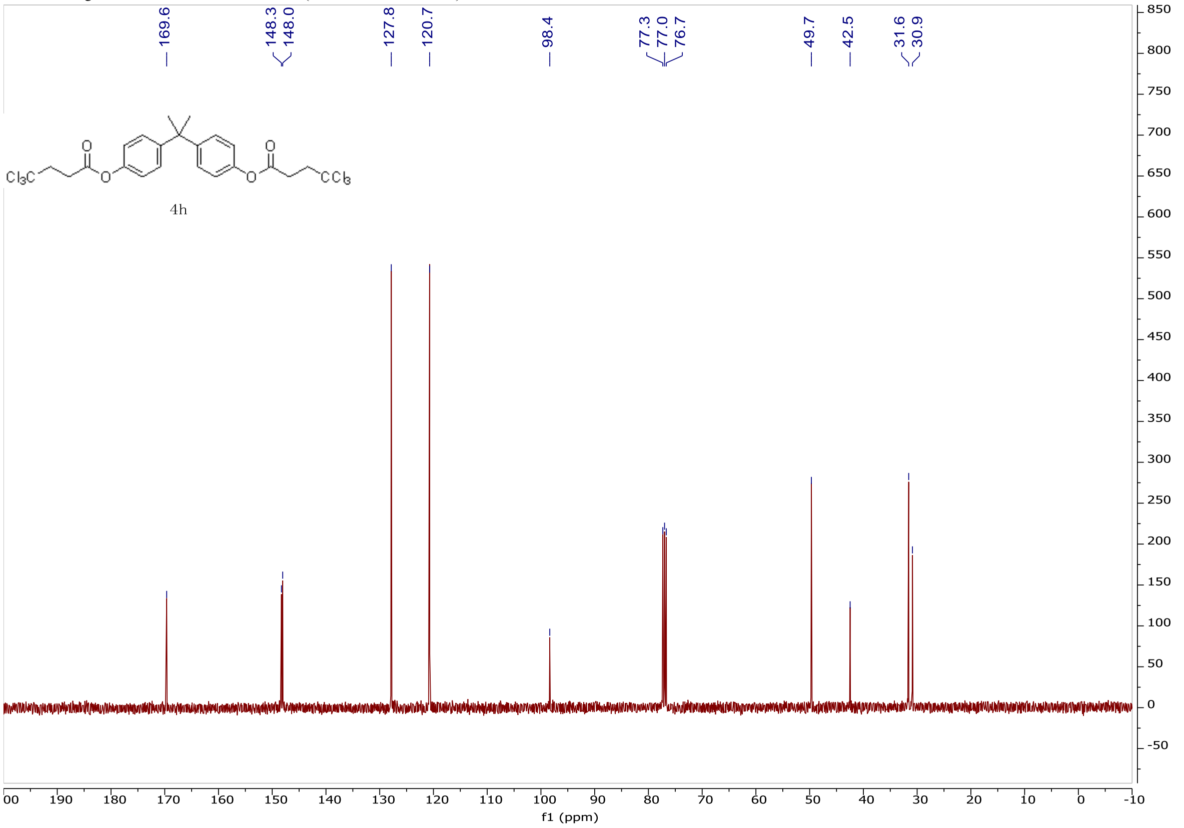

4i

7124-YZHANG-3-303A.10.fid — PROTON CDCl3 {D:\NMR400\DNL0604} nmr-new 31400.13MHz

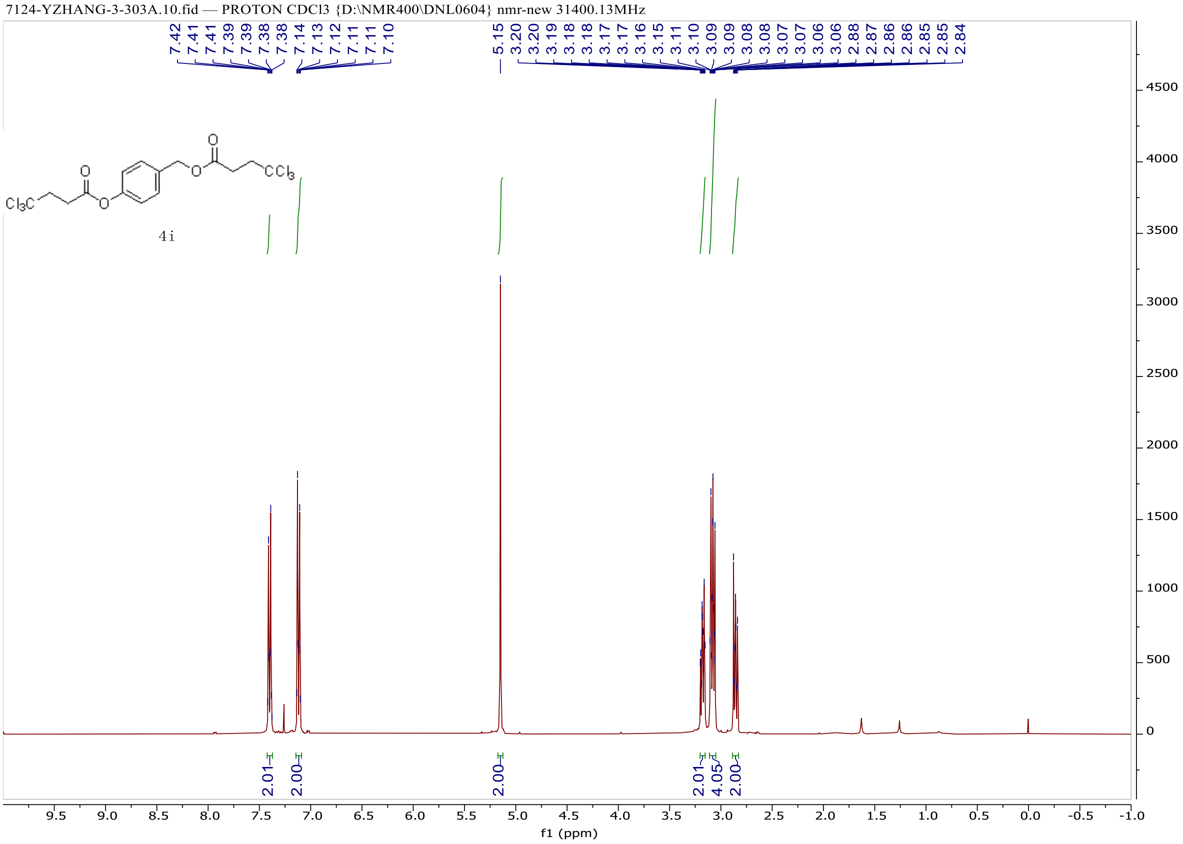

7124-YZHANG-3-303A.11.fid — C13CPD CDCl3 {D:\NMR400\DNL0604} nmr-new 31100.62MHz

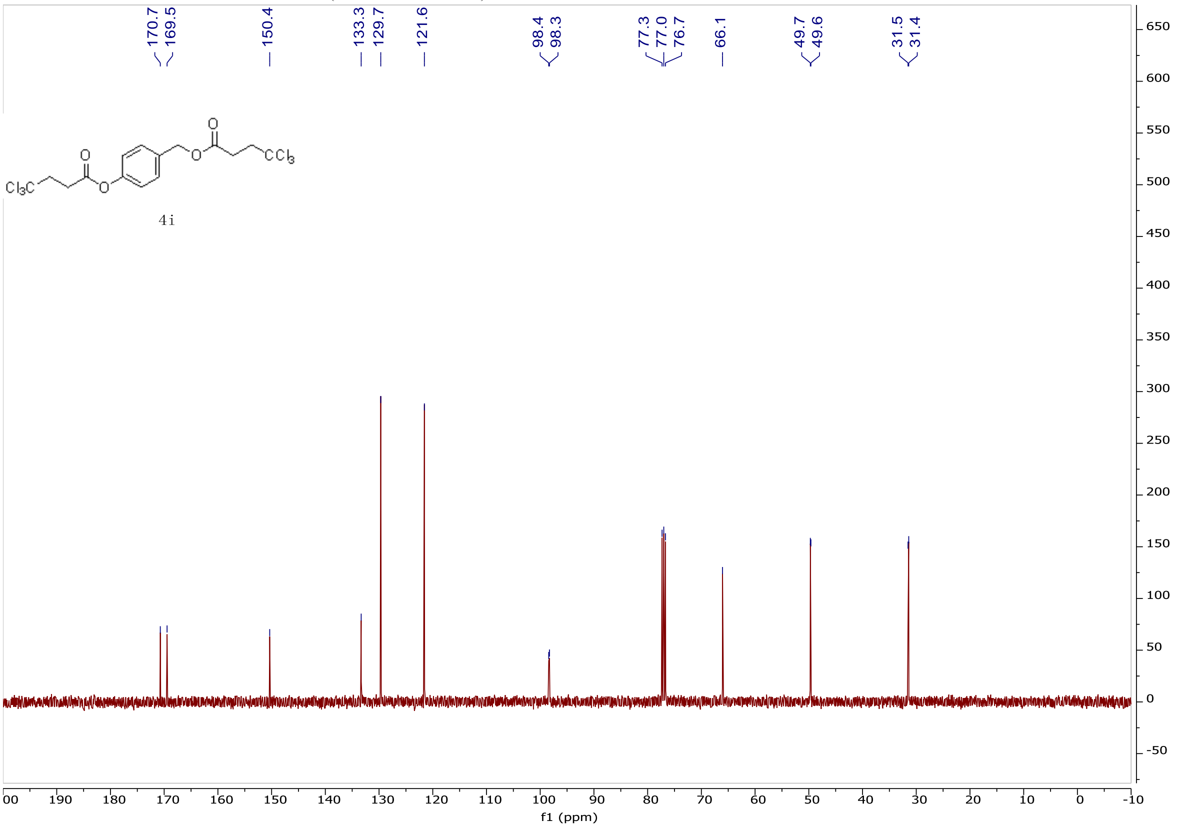

4j

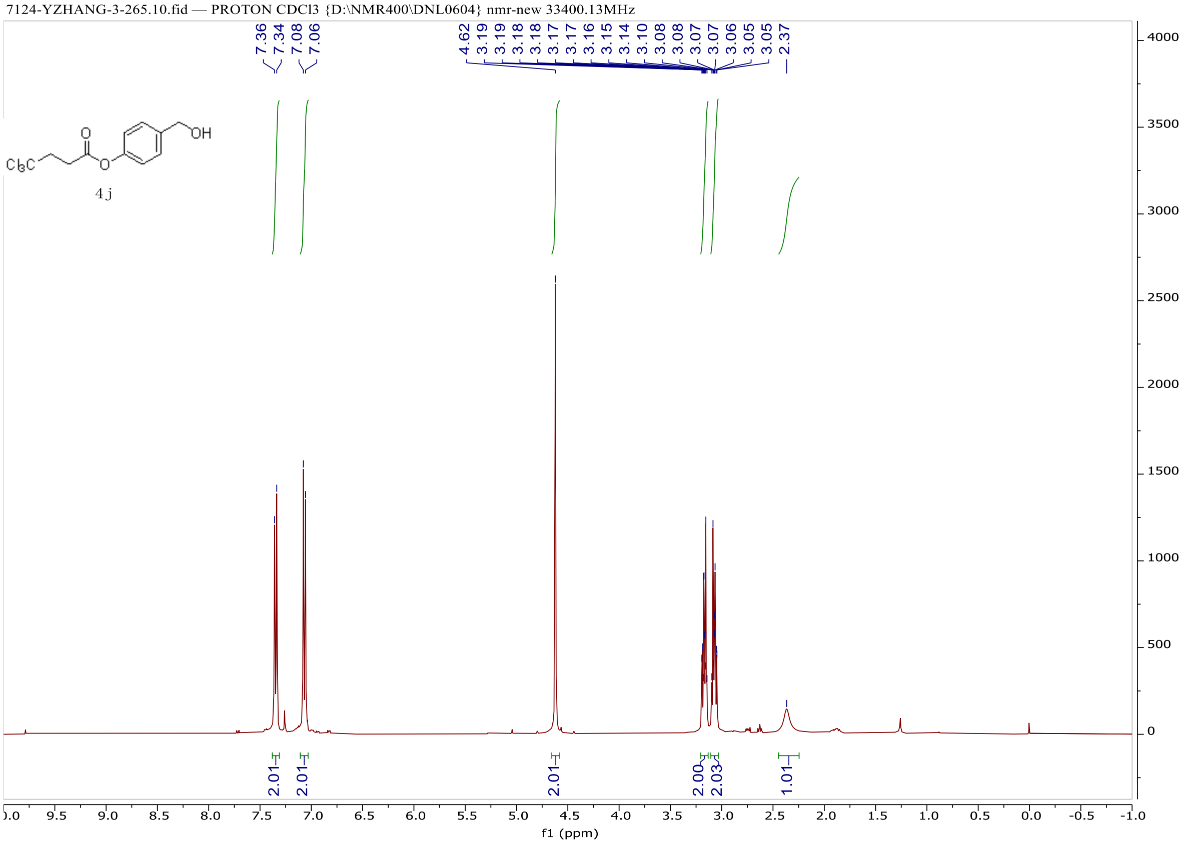
7124-YZHANG-3-265.10.fid — PROTON CDCl3 {D:\NMR400\DNL0604} nmr-new 33400.13MHz
7.36
7.34
7.08
7.06
4.62
3.19
3.19
3.18
3.17
3.16
3.15
3.14
3.10
3.08
3.08
3.07
3.07
3.06
3.05
2.37
2.01
2.01
2.01
2.00
2.03
1.01
4j
f1 (ppm)

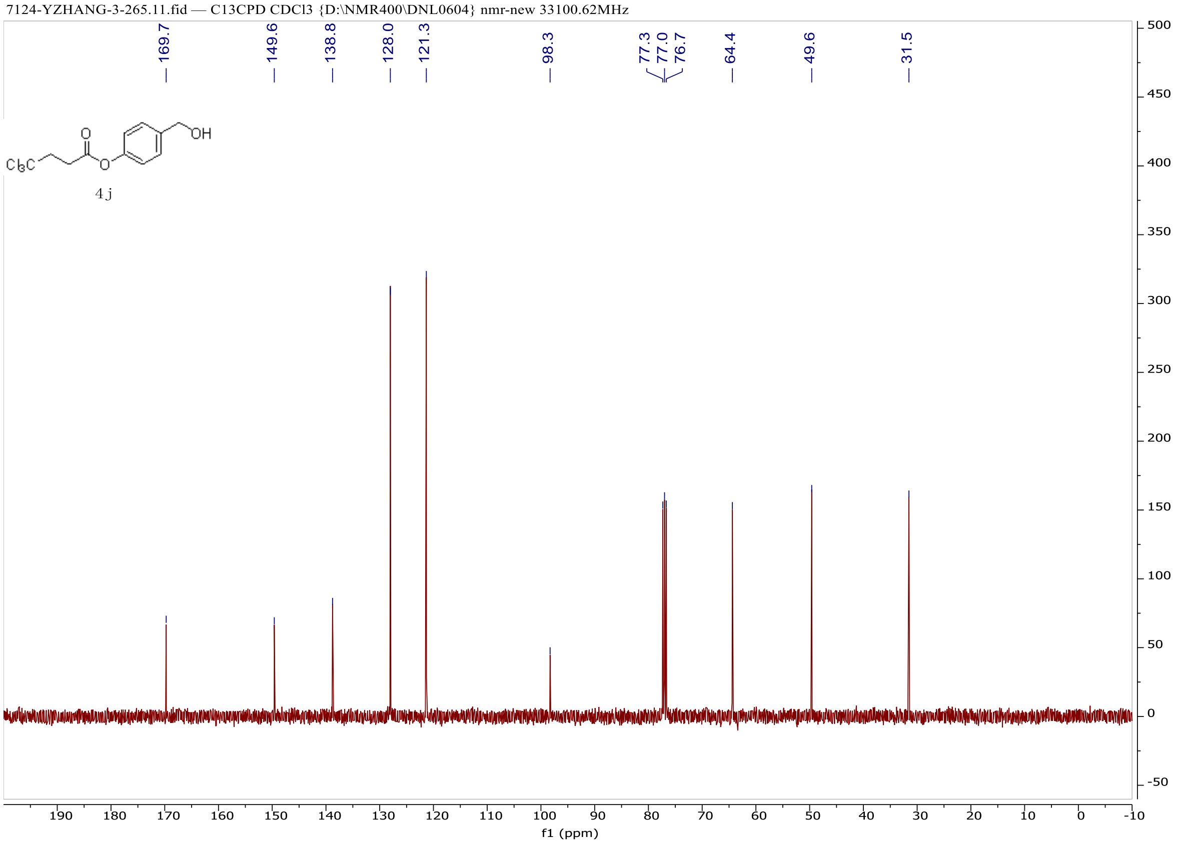
7124-YZHANG-3-265.11.fid — C13CPD CDCl3 {D:\NMR400\DNL0604} nmr-new 33100.62MHz
169.7
149.6
138.8
128.0
121.3
98.3
77.3
77.0
76.7
64.4
49.6
31.5
4j
f1 (ppm)

4k

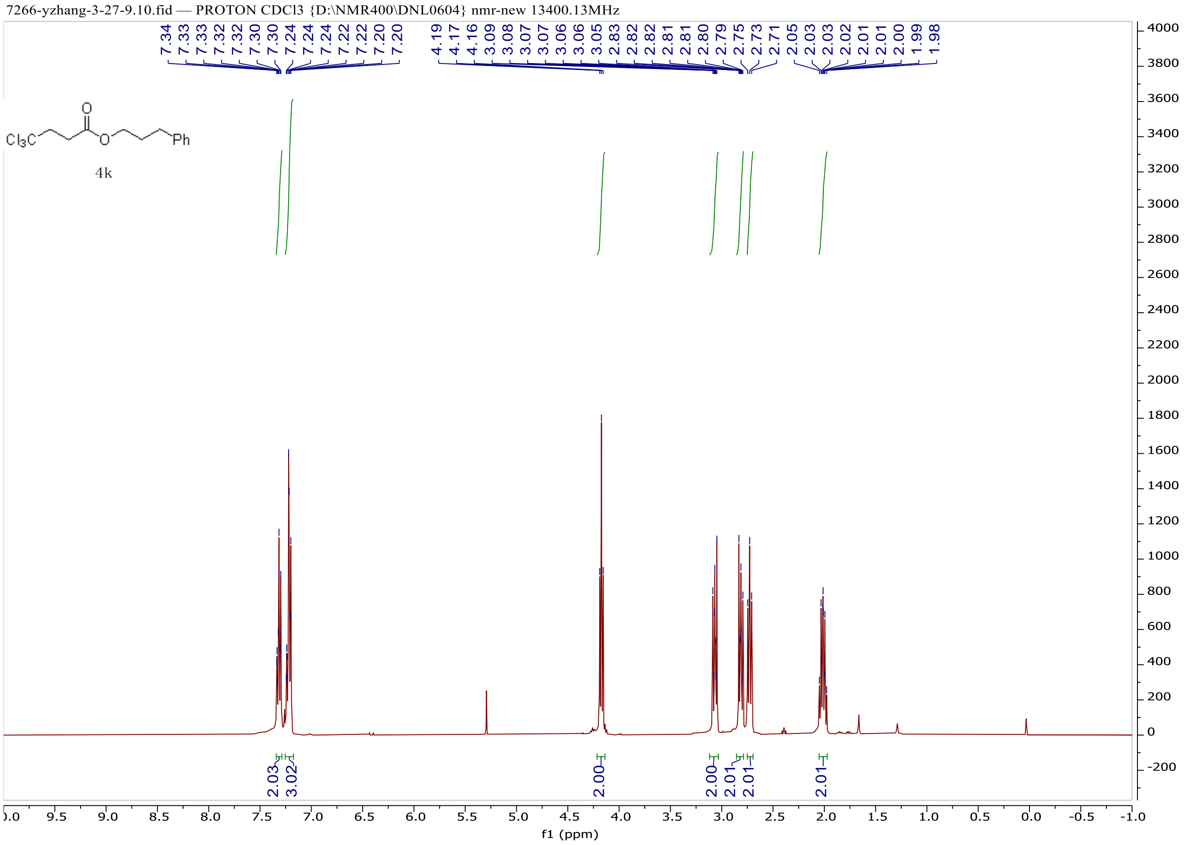
7266-yzhang-3-27-9.10.fid — PROTON CDCl3 {D:\NMR400\DNL0604} nmr-new 13400.13MHz
7.34 7.33 7.33 7.32 7.32 7.30 7.24 7.24 7.24 7.22 7.22 7.20 7.20 4.19 4.17 4.16 3.09 3.08 3.07 3.07 3.06 3.05 2.83 2.82 2.82 2.81 2.81 2.80 2.79 2.75 2.73 2.71 2.05 2.03 2.03 2.02 2.01 2.01 2.00 1.99 1.98
4k
2.03 3.02 2.00 2.00 2.01 2.01 2.01
f1 (ppm)

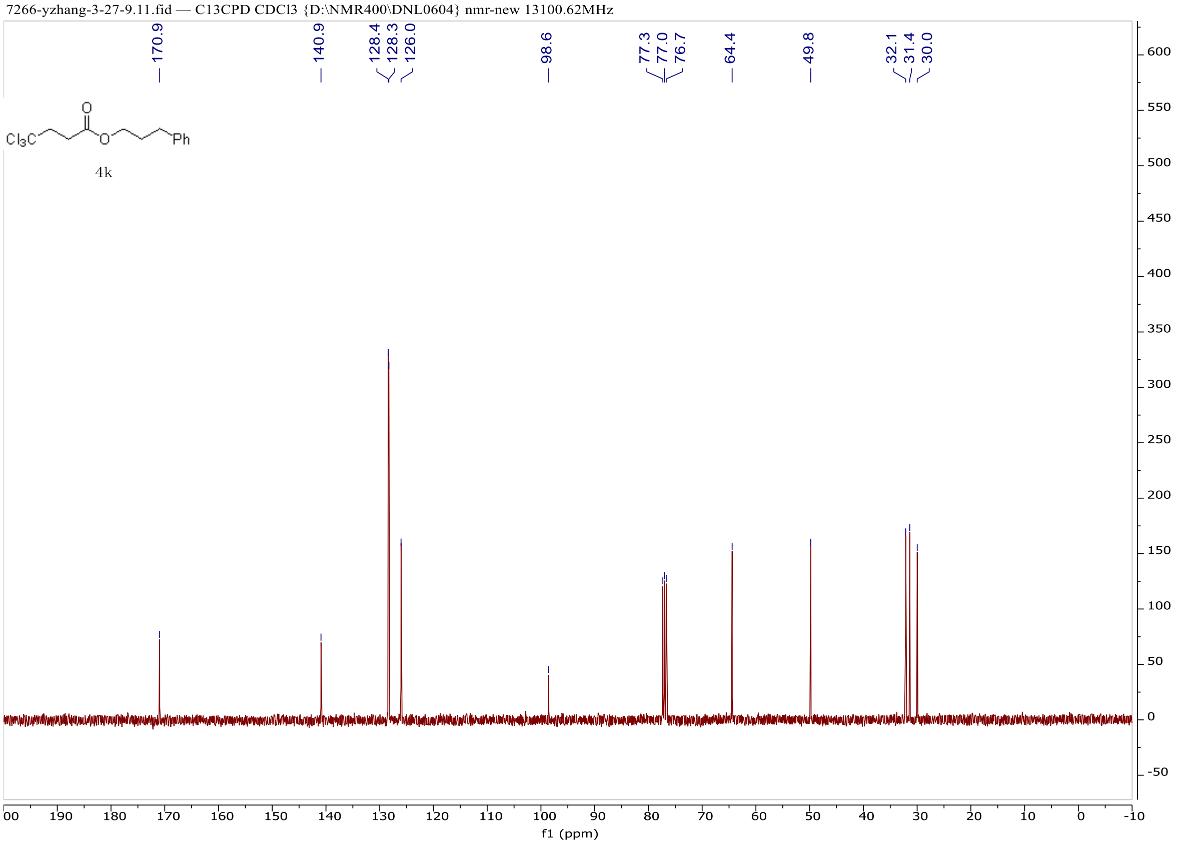
7266-yzhang-3-27-9.11.fid — C13CPD CDCl3 {D:\NMR400\DNL0604} nmr-new 13100.62MHz
170.9 140.9 128.4 128.3 126.0 98.6 77.3 77.0 76.7 64.4 49.8 32.1 31.4 30.0
4k
f1 (ppm)

4l

5951-YZhang-3-90.10.fid — PROTON CDCl3 {D:\NMR400\DNL0604} nmr-new 45400.13MHz

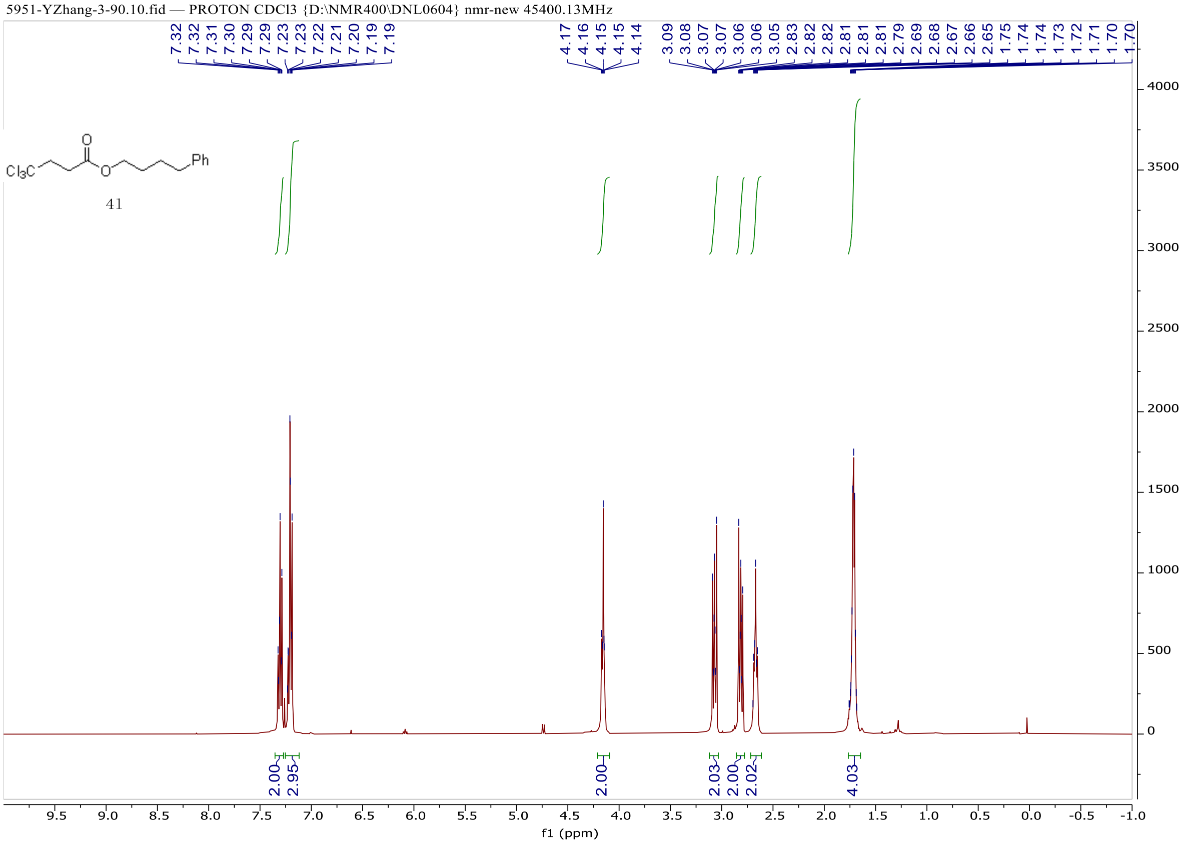

5951-YZhang-3-90.11.fid — C13CPD CDCl3 {D:\NMR400\DNL0604} nmr-new 45100.62MHz

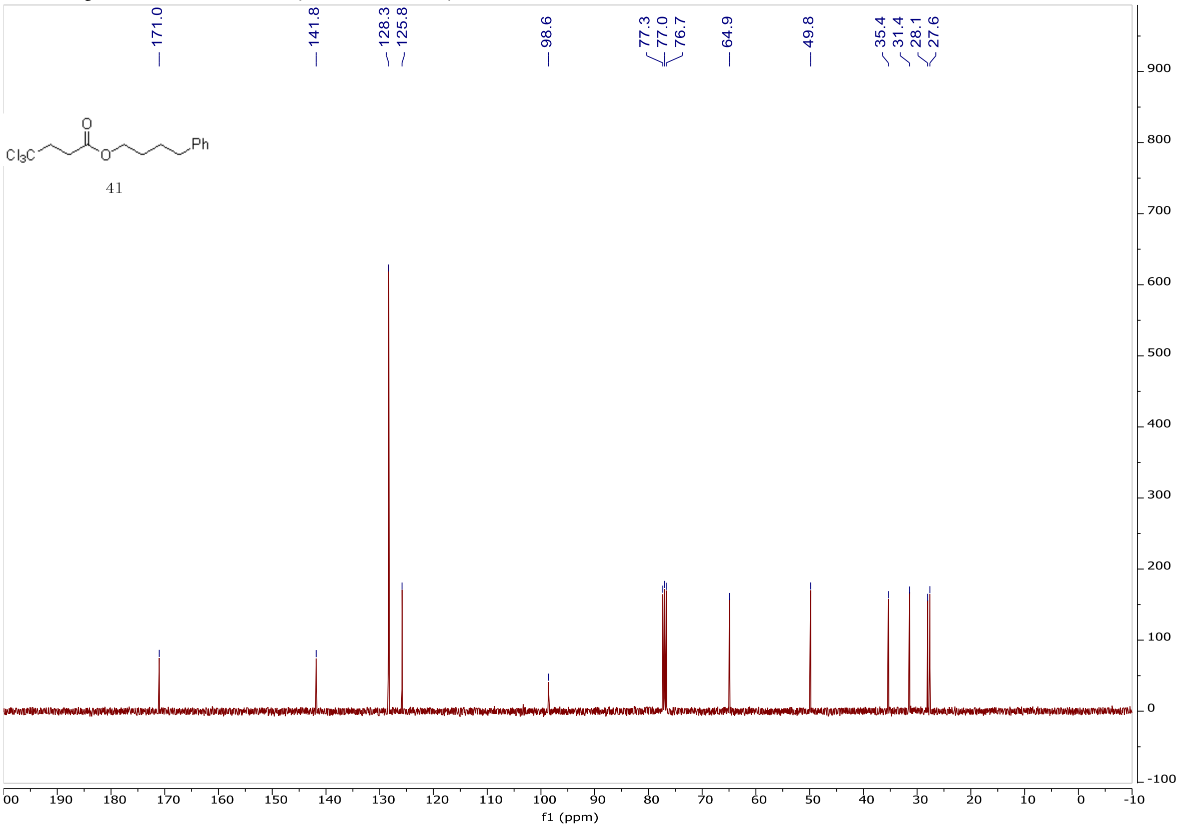

4m

6072-YZhang-3-126.10.fid — PROTON CDCl3 {D:\NMR400\DNL0604} nmr-new 18400.13MHz

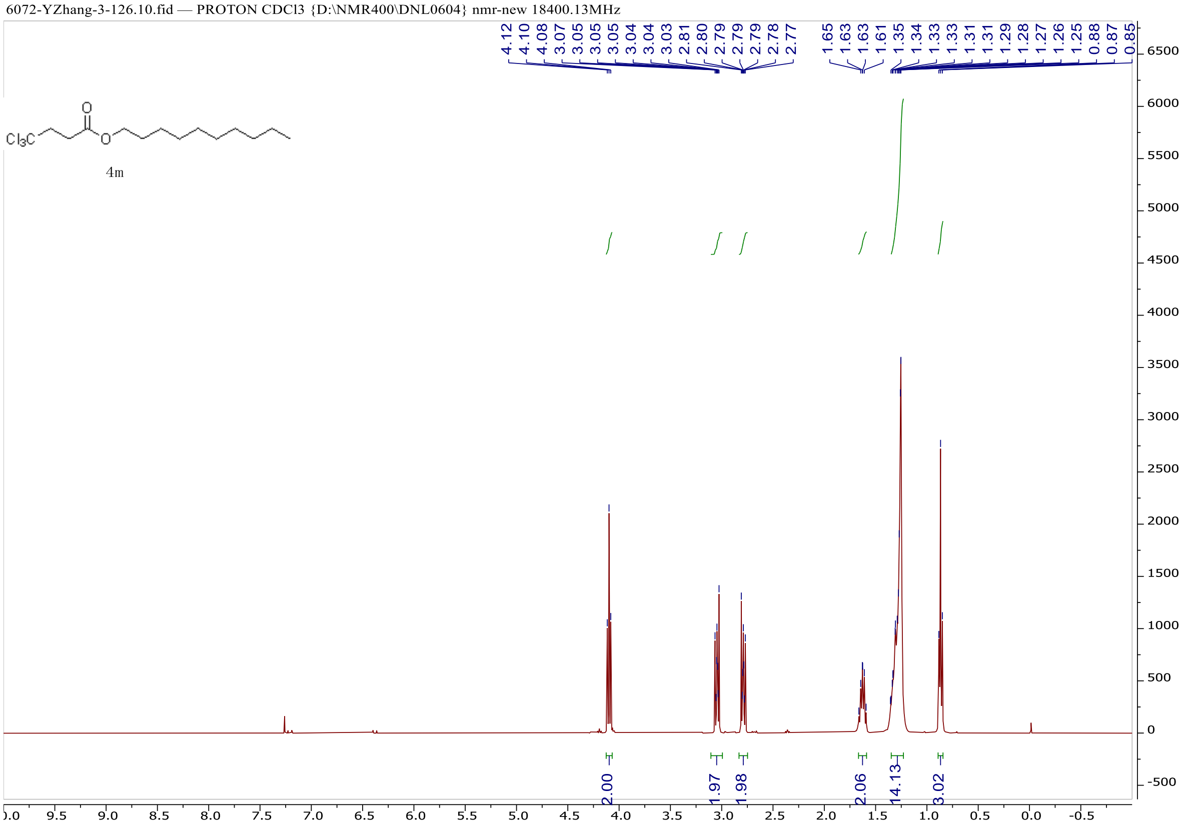

6072-YZhang-3-126.52.fid — C13CPD CDCl3 {D:\NMR400\DNL0604} nmr-new 18100.62MHz

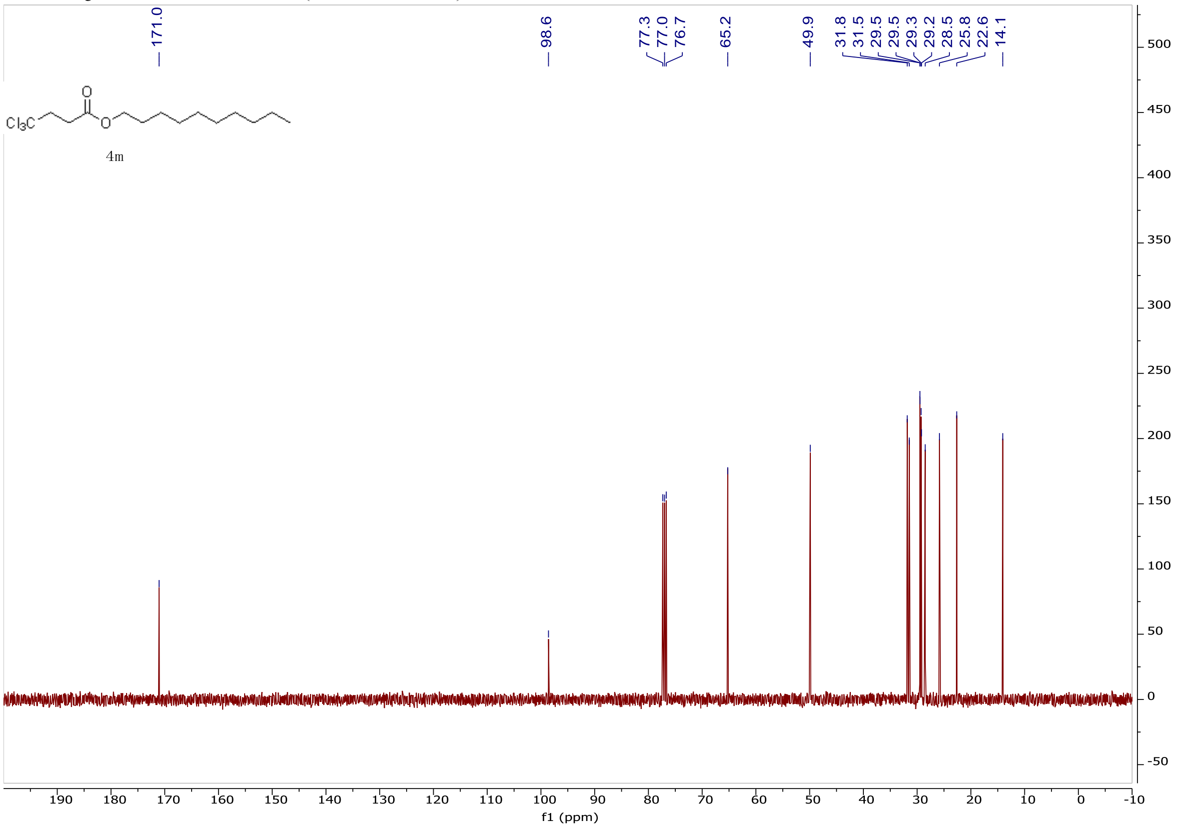

4n

7026-yzhang-3-275.10.fid — PROTON CDCl3 {D:\NMR400\DNL0604} nmr-new 4400.13MHz

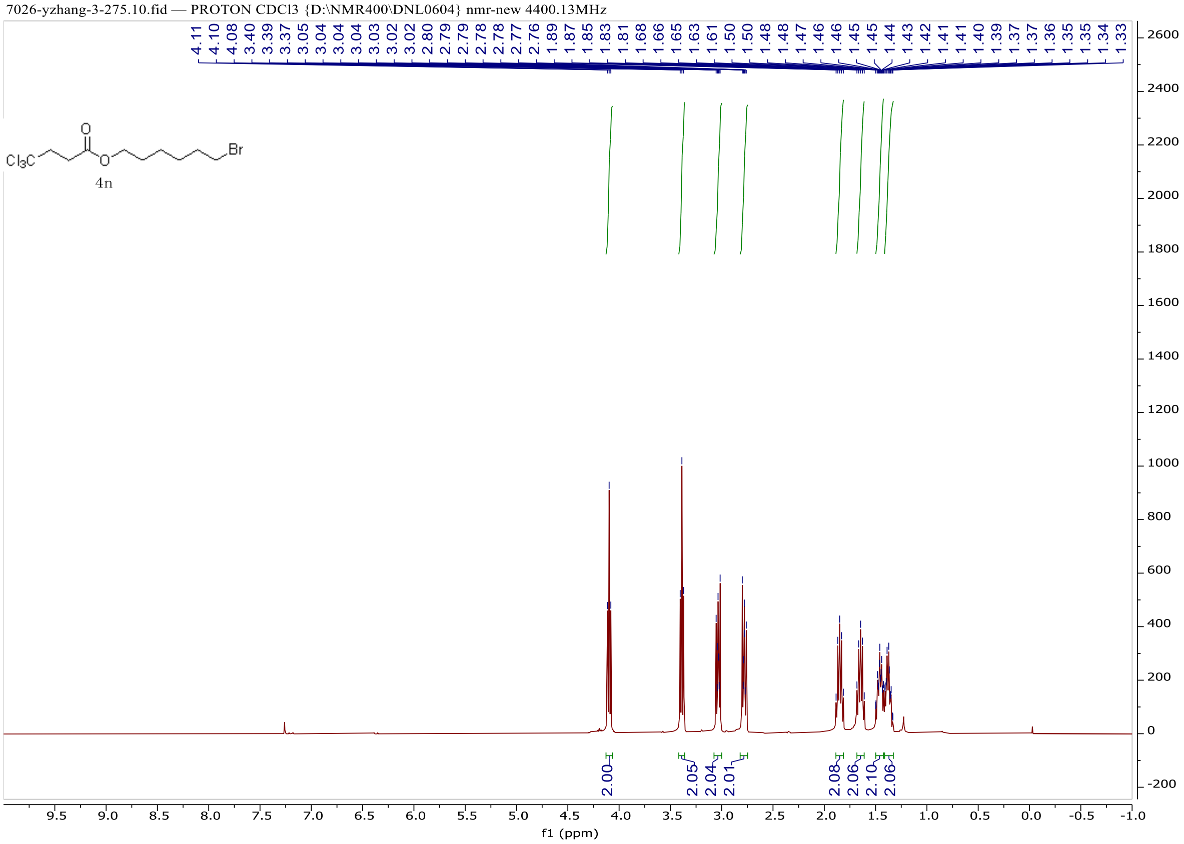

7026-yzhang-3-275.11.fid — C13CPD CDCl3 {D:\NMR400\DNL0604} nmr-new 4100.62MHz

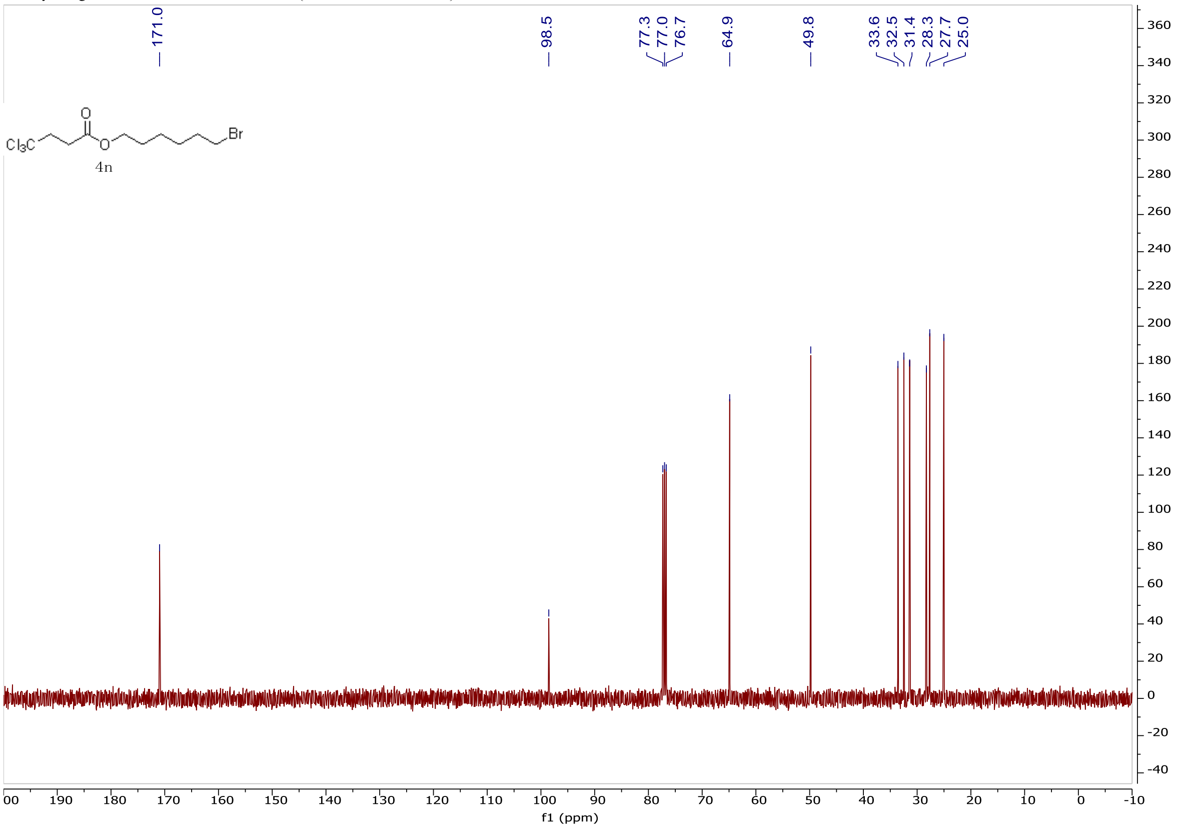

4o

6256-YZhang-0-3-133.10.fid — PROTON CDCl3 {D:\NMR400\DNL0604} nmr-new 23400.13MHz

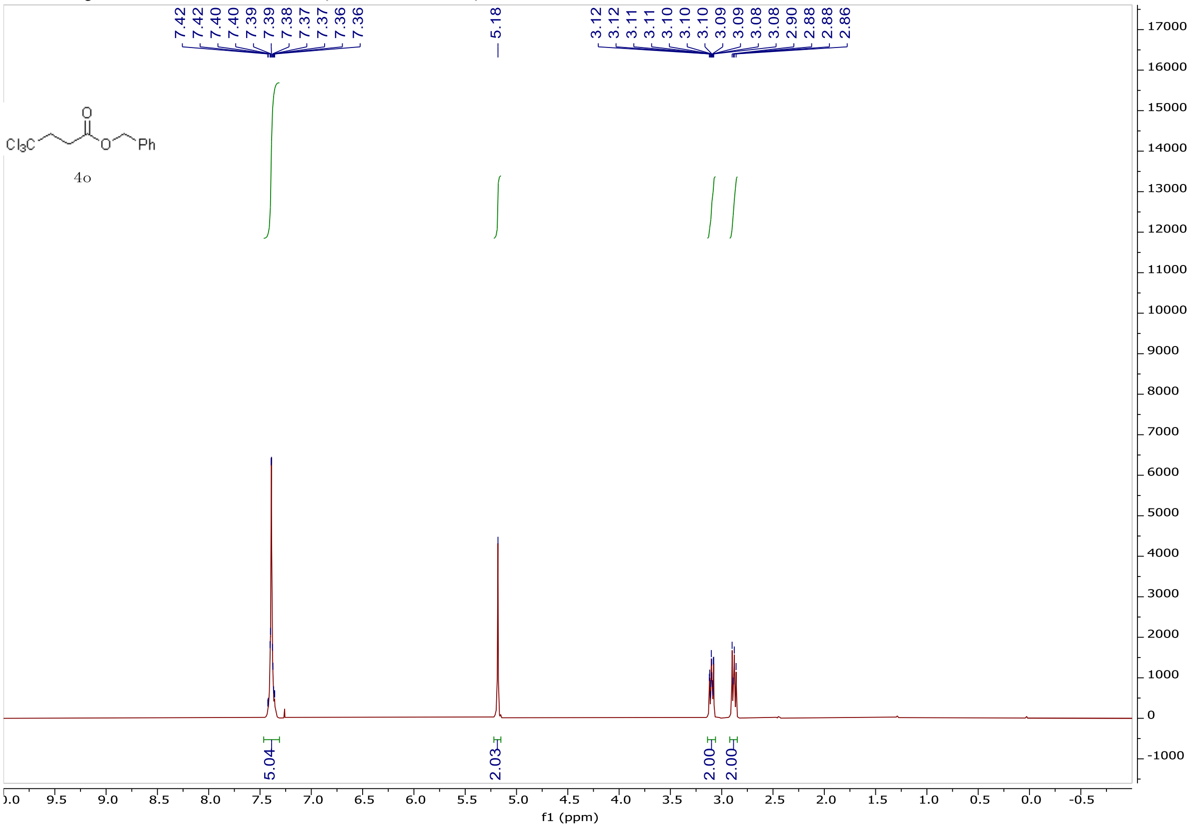

6256-YZhang-0-3-133.11.fid — C13CPD CDCl3 {D:\NMR400\DNL0604} nmr-new 23100.62MHz

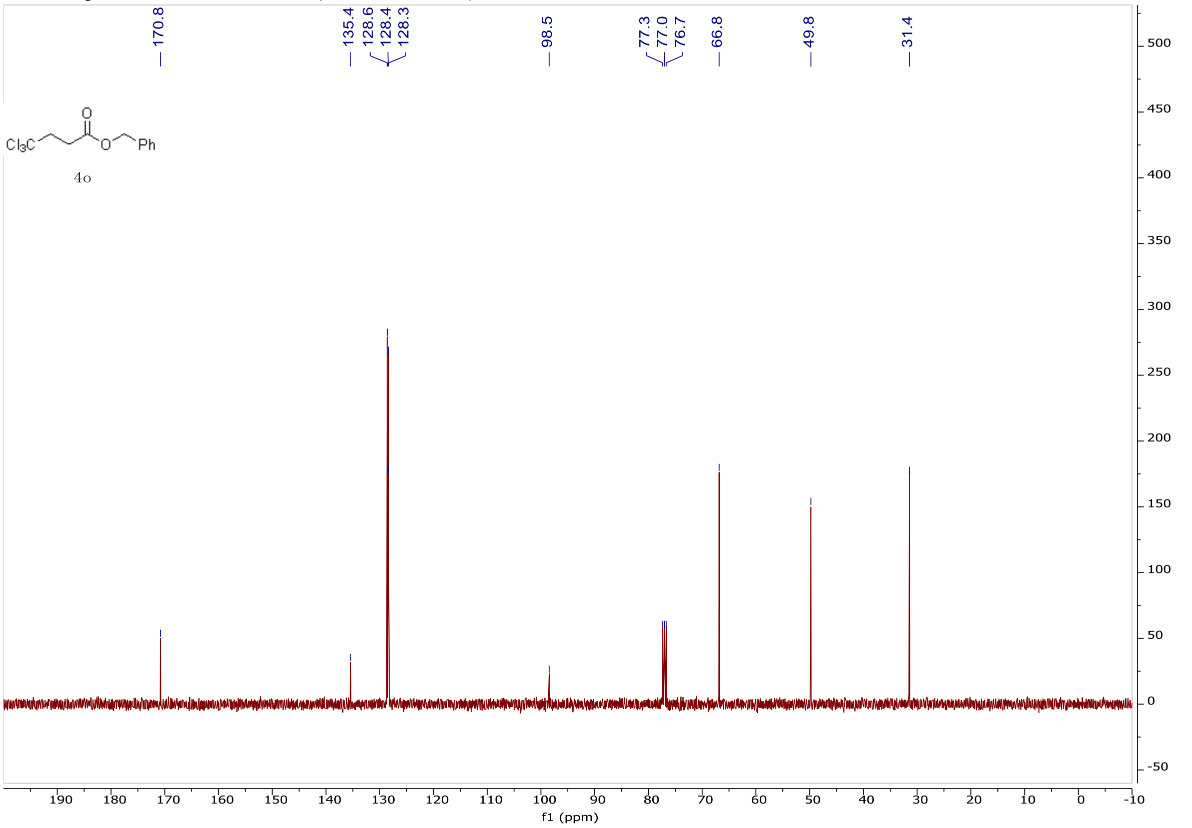

4p

7002-YZhang-3-267.10.fid — PROTON CDCl3 {D:\NMR400\DNL0604} nmr-new 49400.13MHz

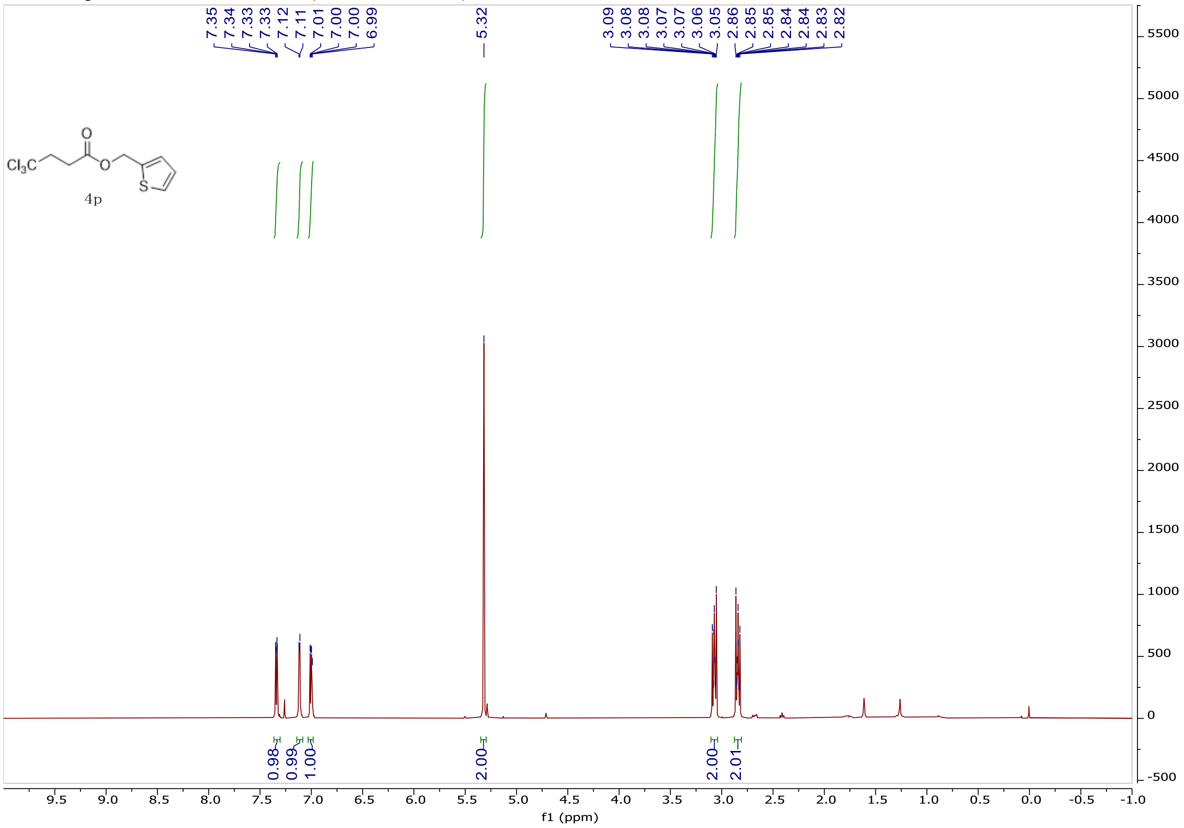

7002-YZhang-3-267.11.fid — C13CPD CDCl3 {D:\NMR400\DNL0604} nmr-new 49100.62MHz

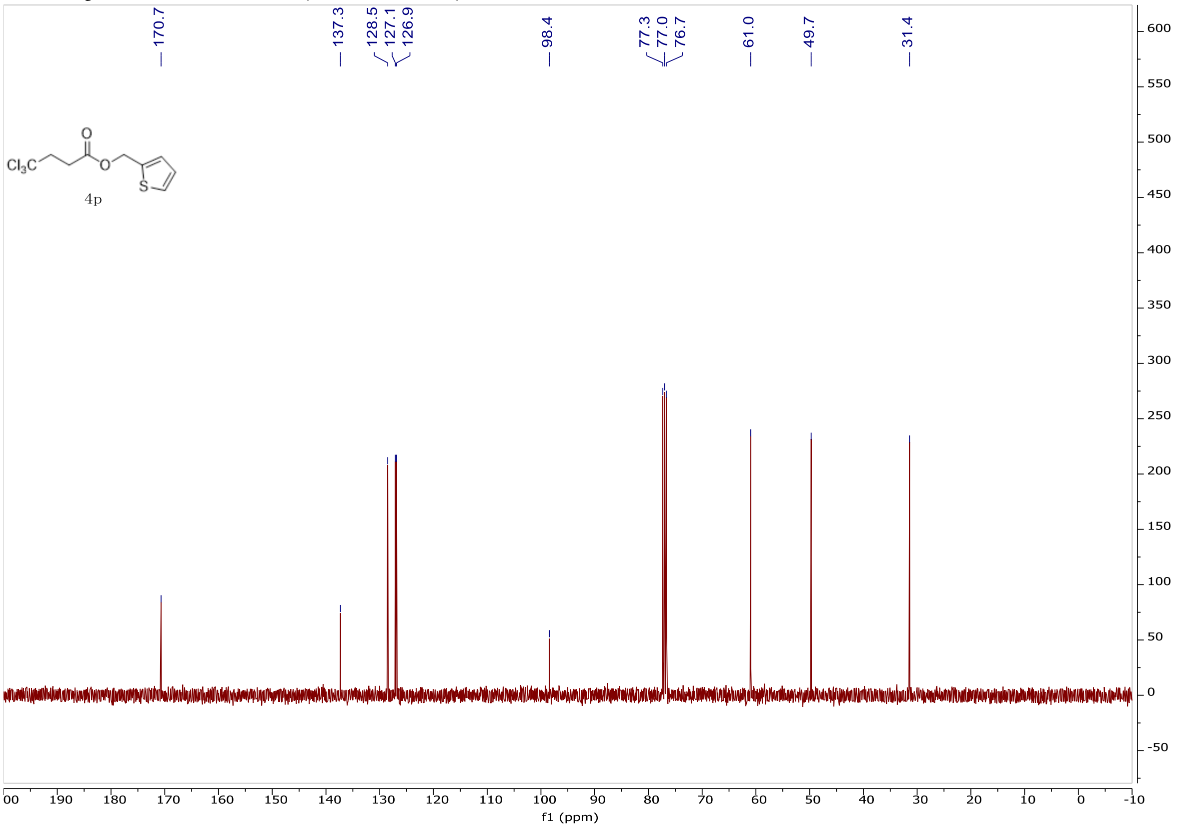

4q

7002-YZhang-3-269.10.fid — PROTON CDCl3 {D:\NMR400\DNL0604} nmr-new 50400.13MHz

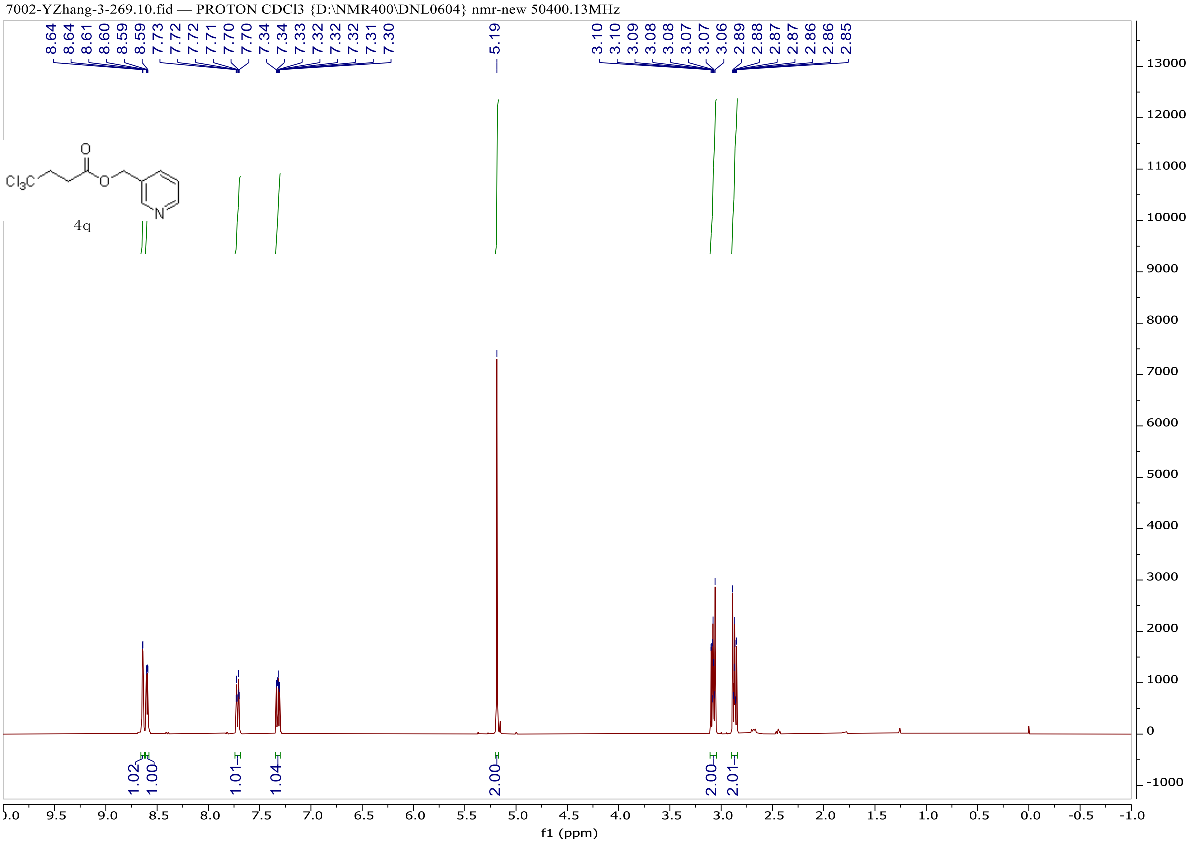

7002-YZhang-3-269.11.fid — C13CPD CDCl3 {D:\NMR400\DNL0604} nmr-new 50100.62MHz

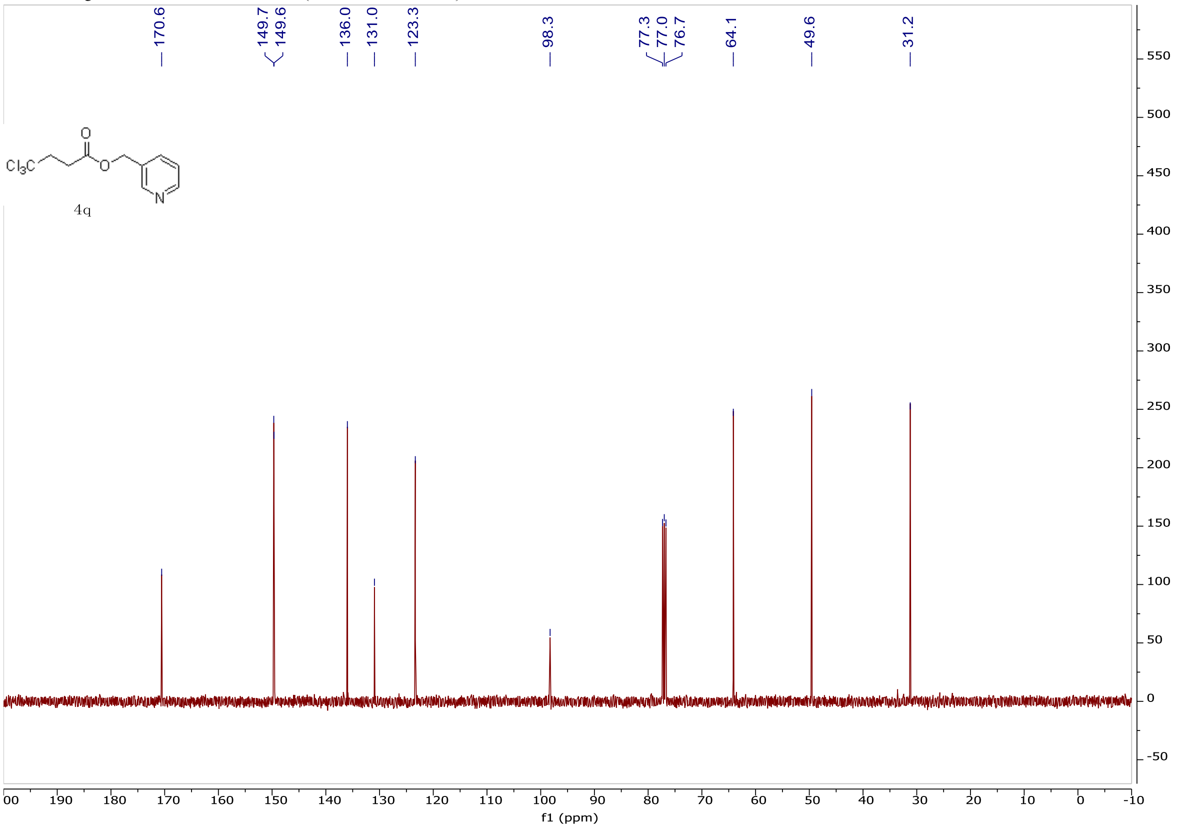

4r

7093-yzhang-3-302.10.fid — PROTON CDCl3 {D:\NMR400\DNL0604} nmr-new 11400.13MHz

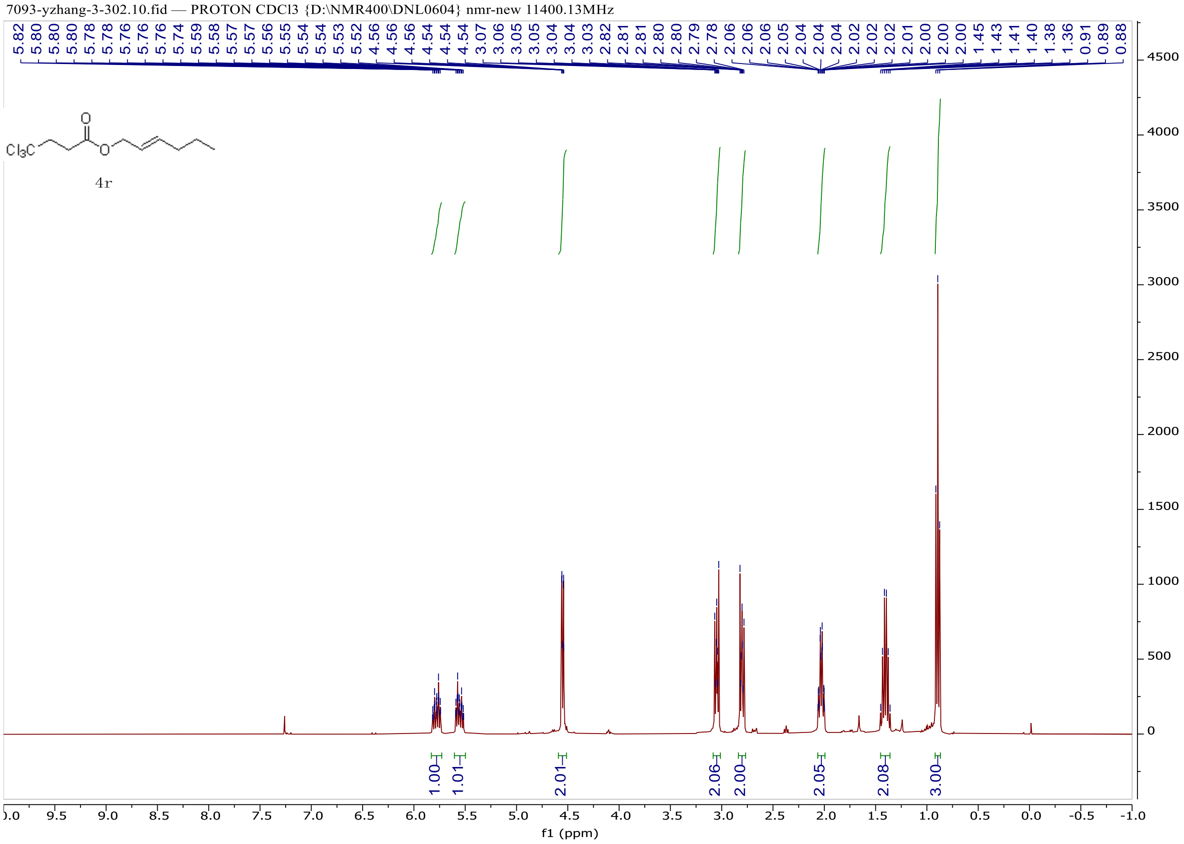

7093-yzhang-3-302.11.fid — C13CPD CDCl3 {D:\NMR400\DNL0604} nmr-new 11100.62MHz

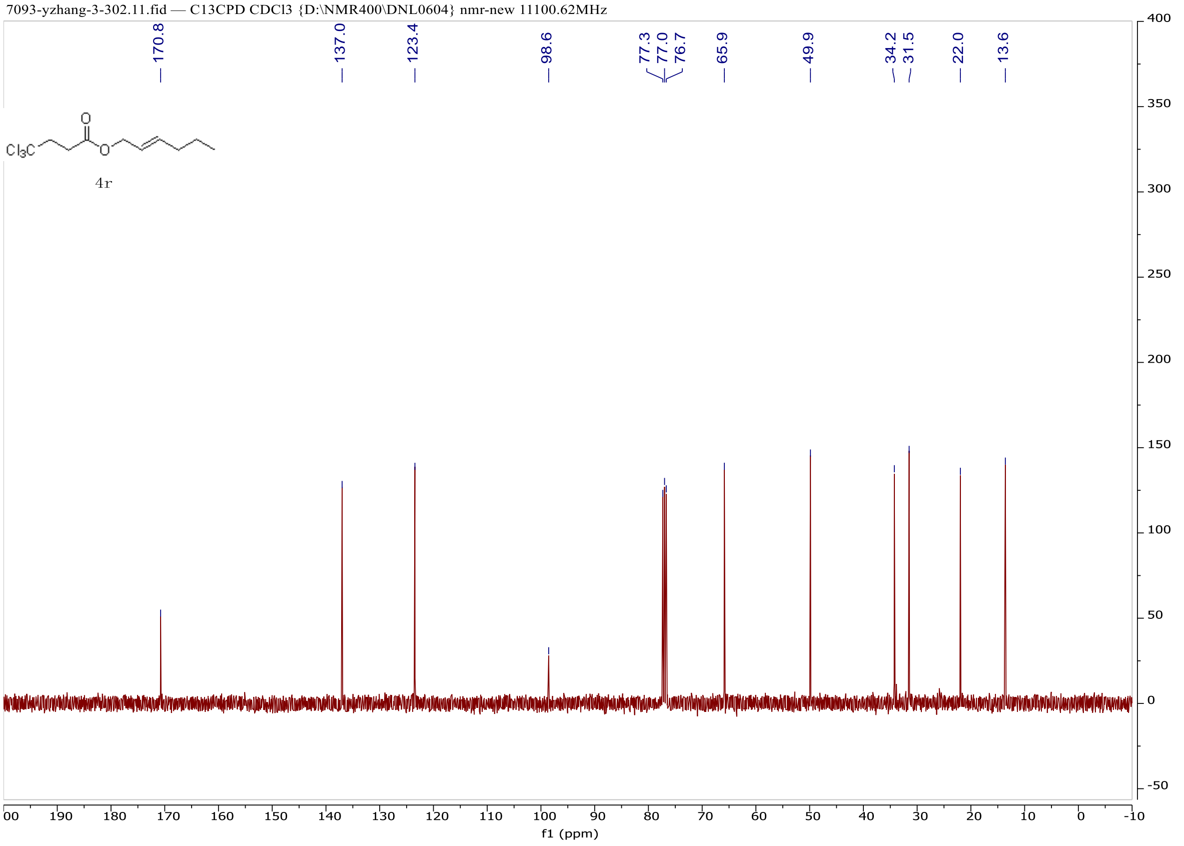

4s

7002-YZhang-3-265.10.fid — PROTON CDCl3 {D:\NMR400\DNL0604} nmr-new 47400.13MHz

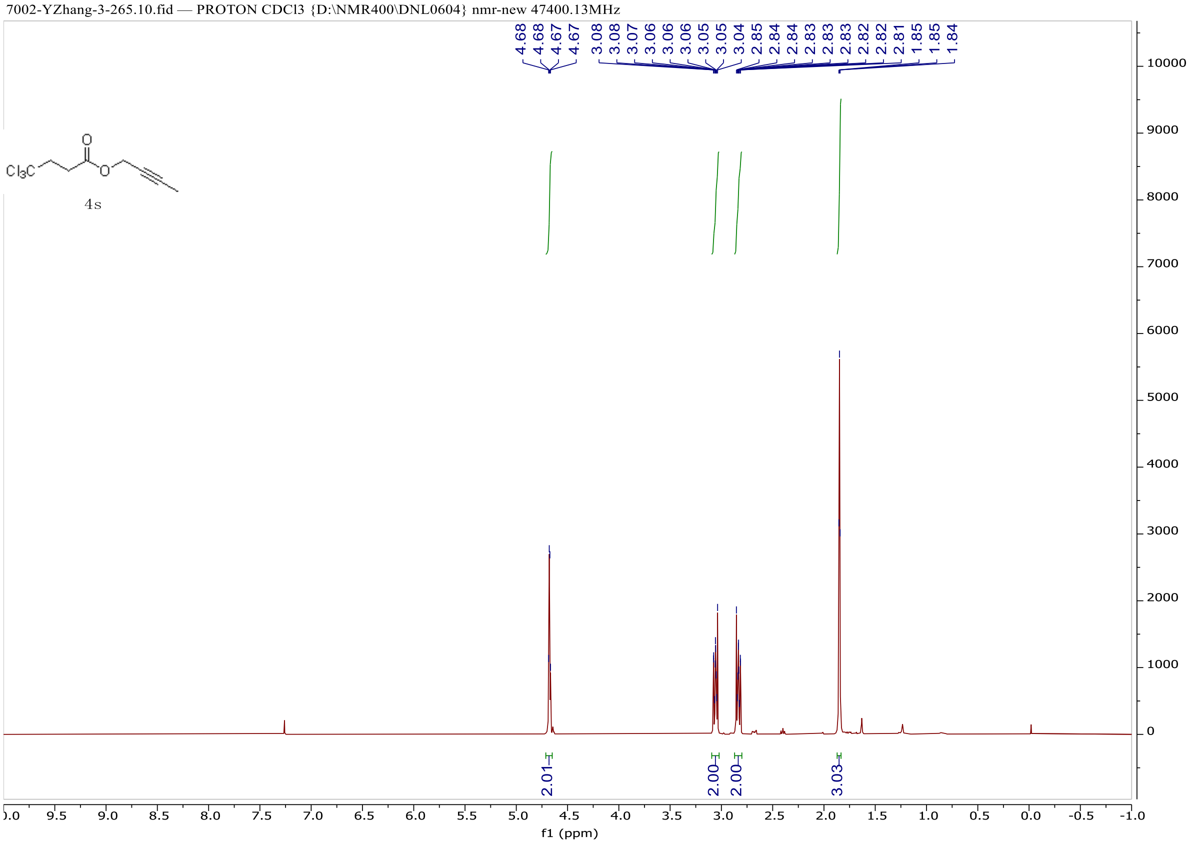

7002-YZhang-3-265.11.fid — C13CPD CDCl3 {D:\NMR400\DNL0604} nmr-new 47100.62MHz

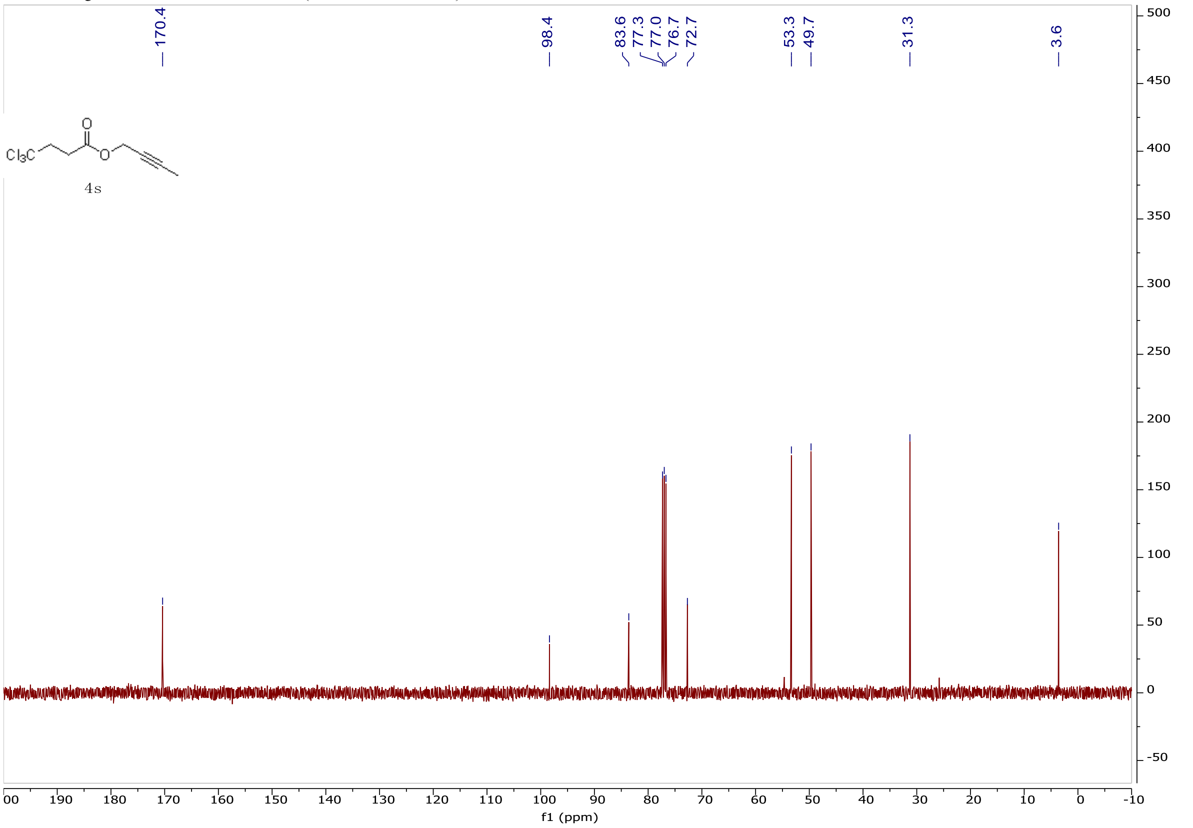

4t

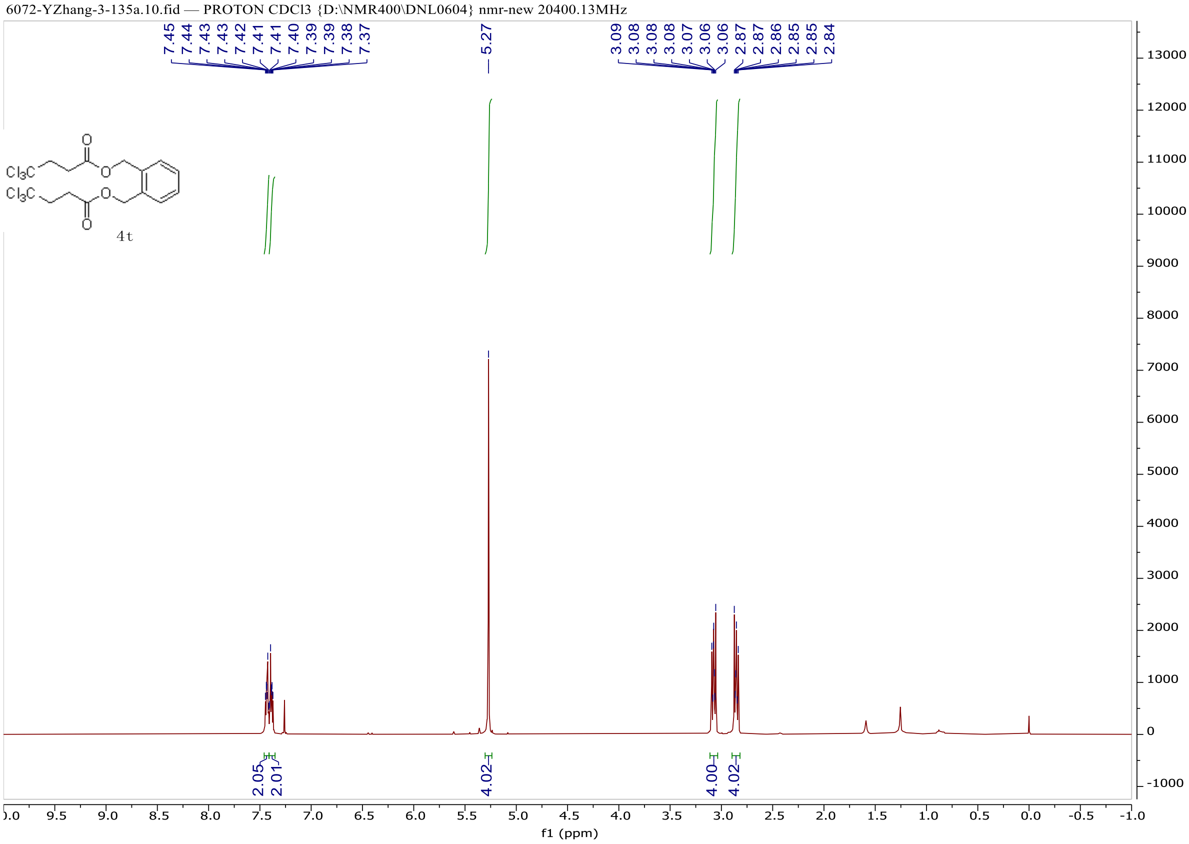
6072-YZhang-3-135a.10.fid — PROTON CDCl3 {D:\NMR400\DNL0604} nmr-new 20400.13MHz
7.45 7.44 7.43 7.42 7.41 7.41 7.40 7.39 7.39 7.38 7.37
5.27
3.09 3.08 3.08 3.08 3.07 3.06 3.06 2.87 2.87 2.86 2.85 2.85 2.84
2.05 2.01 4.02 4.00 4.02
f1 (ppm)
4t

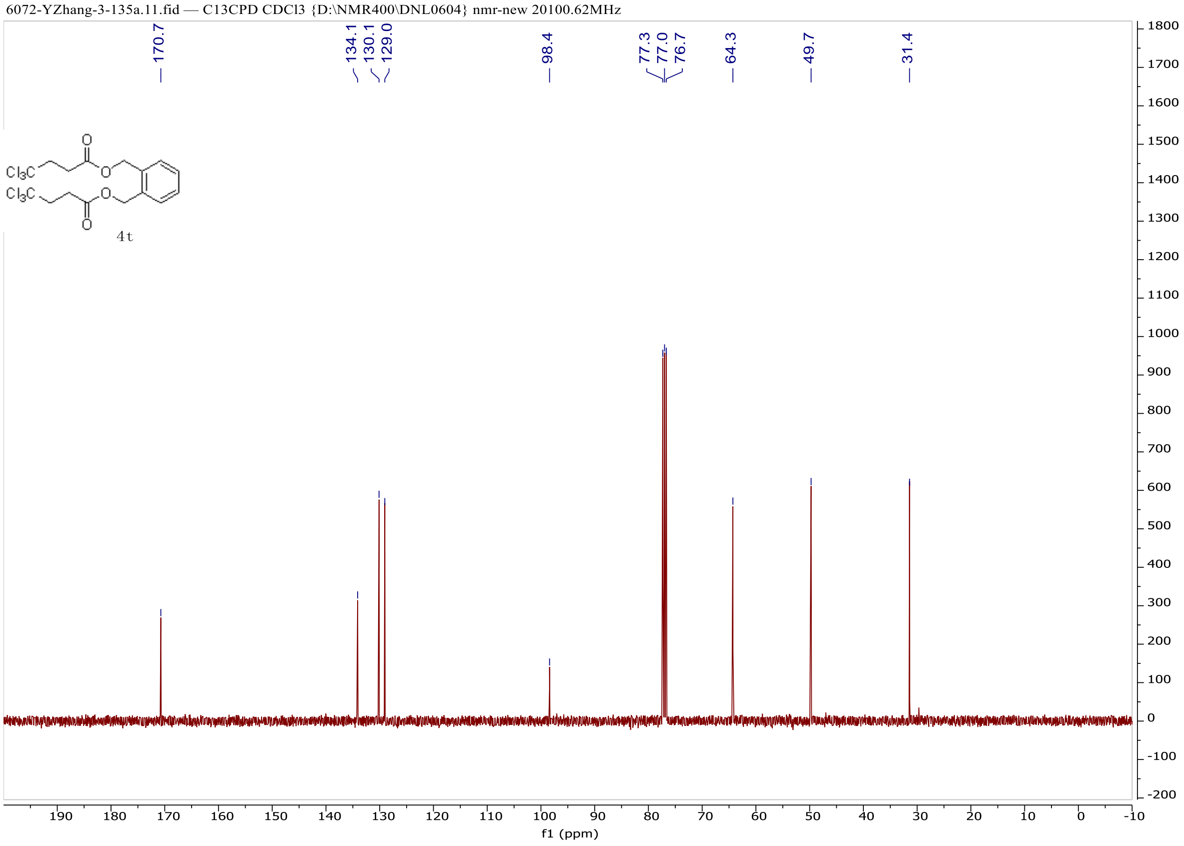
6072-YZhang-3-135a.11.fid — C13CPD CDCl3 {D:\NMR400\DNL0604} nmr-new 20100.62MHz
170.7
134.1 130.1 129.0
98.4
77.3 77.0 76.7
64.3
49.7
31.4
f1 (ppm)
4t

4u

6072-YZhang-3-135b.10.fid — PROTON CDCl3 {D:\NMR400\DNL0604} nmr-new 21400.13MHz

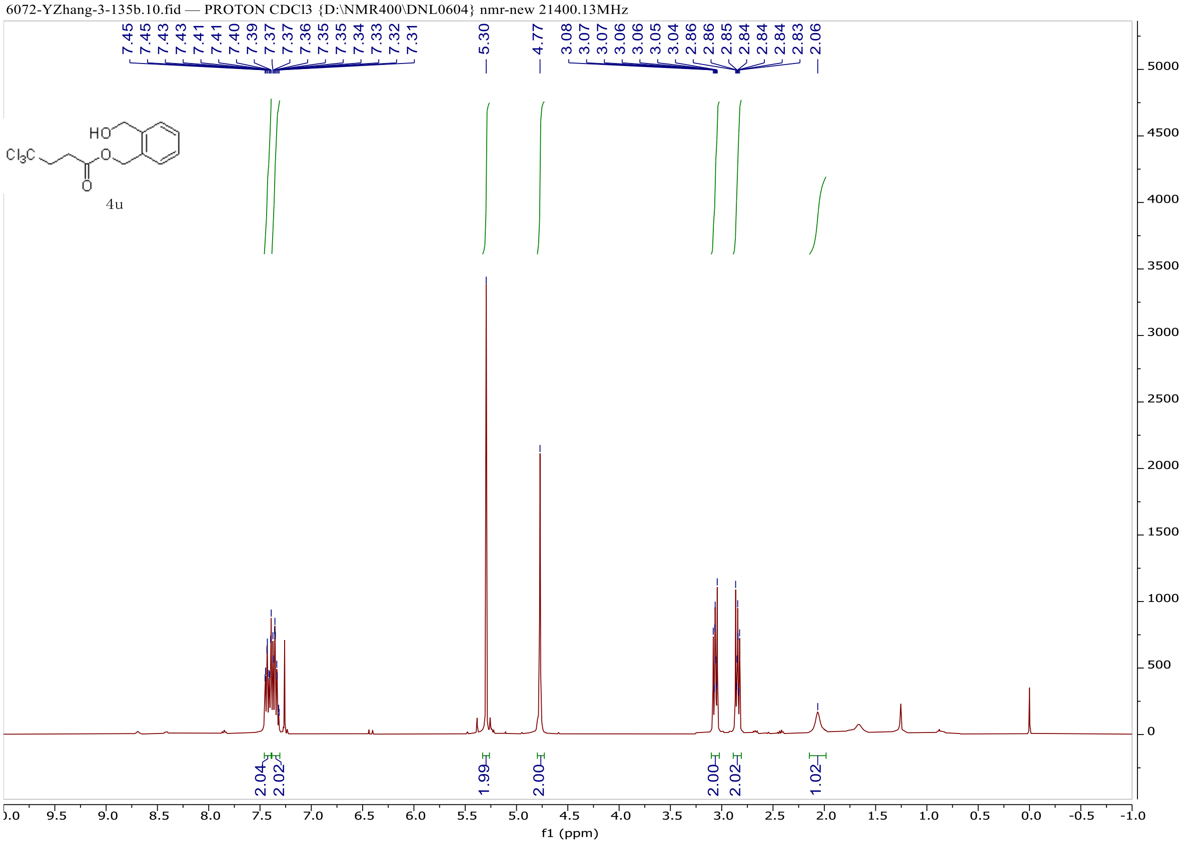

6072-YZhang-3-135b.11.fid — C13CPD CDCl3 {D:\NMR400\DNL0604} nmr-new 21100.62MHz

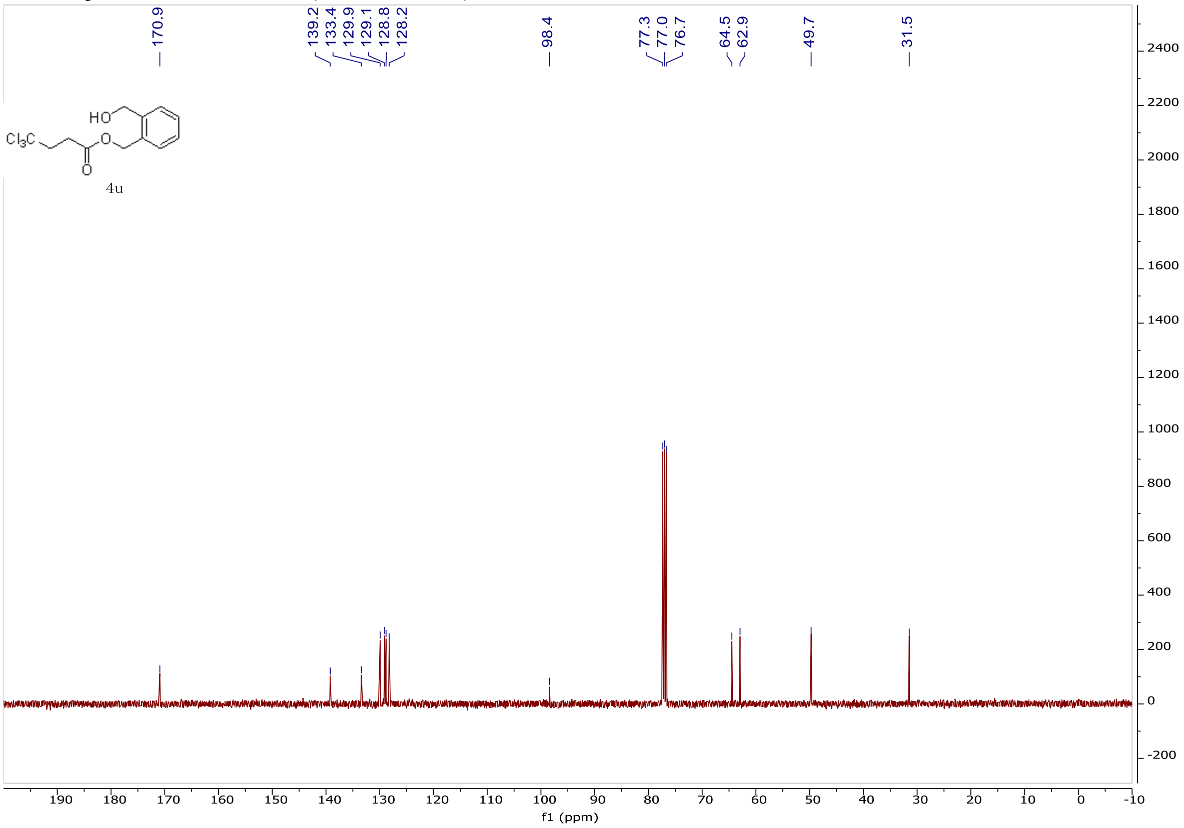

4v

6958-yzhang-3-261.10.fid — PROTON CDCl3 {D:\NMR400\DNL0604} nmr-new 45400.13MHz

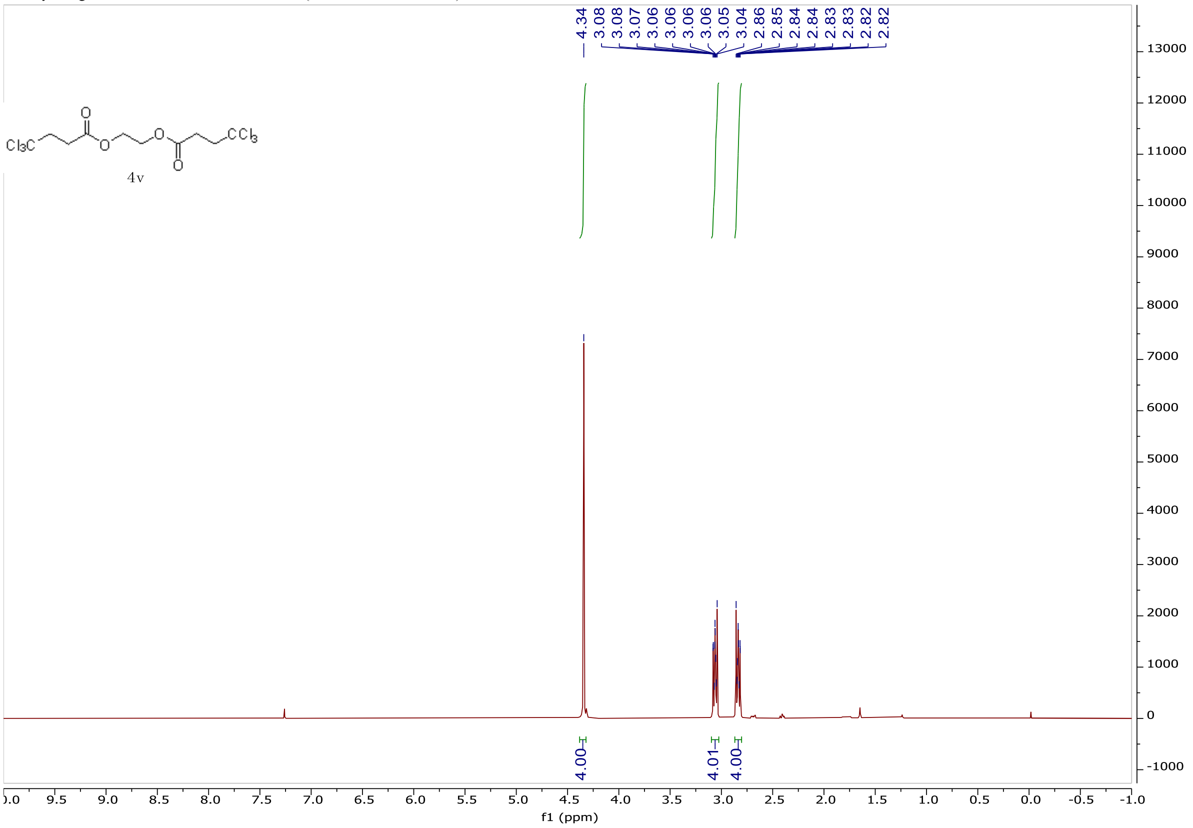

6958-yzhang-3-261.11.fid — C13CPD CDCl3 {D:\NMR400\DNL0604} nmr-new 45100.62MHz

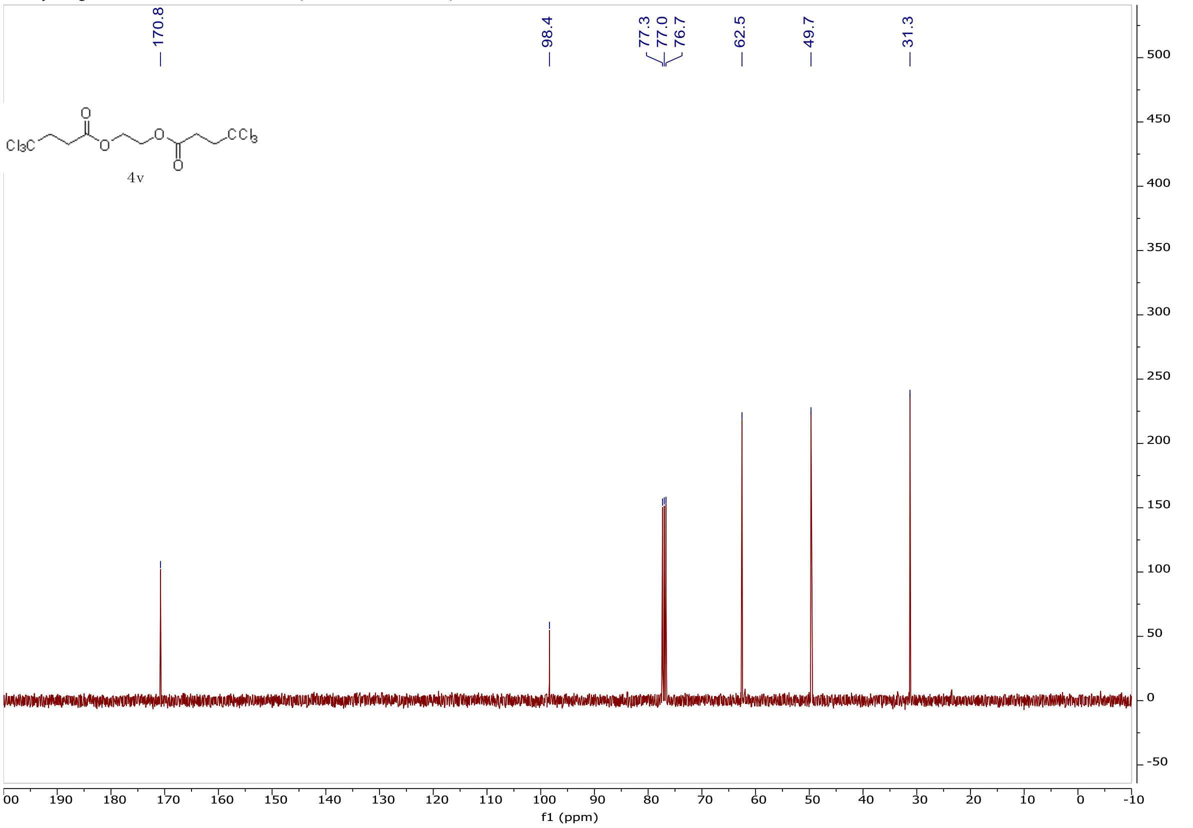

4w

7207-yzhang-3-297a.10.fid — PROTON CDCl3 {D:\NMR400\DNL0604} nmr-new 37400.13MHz

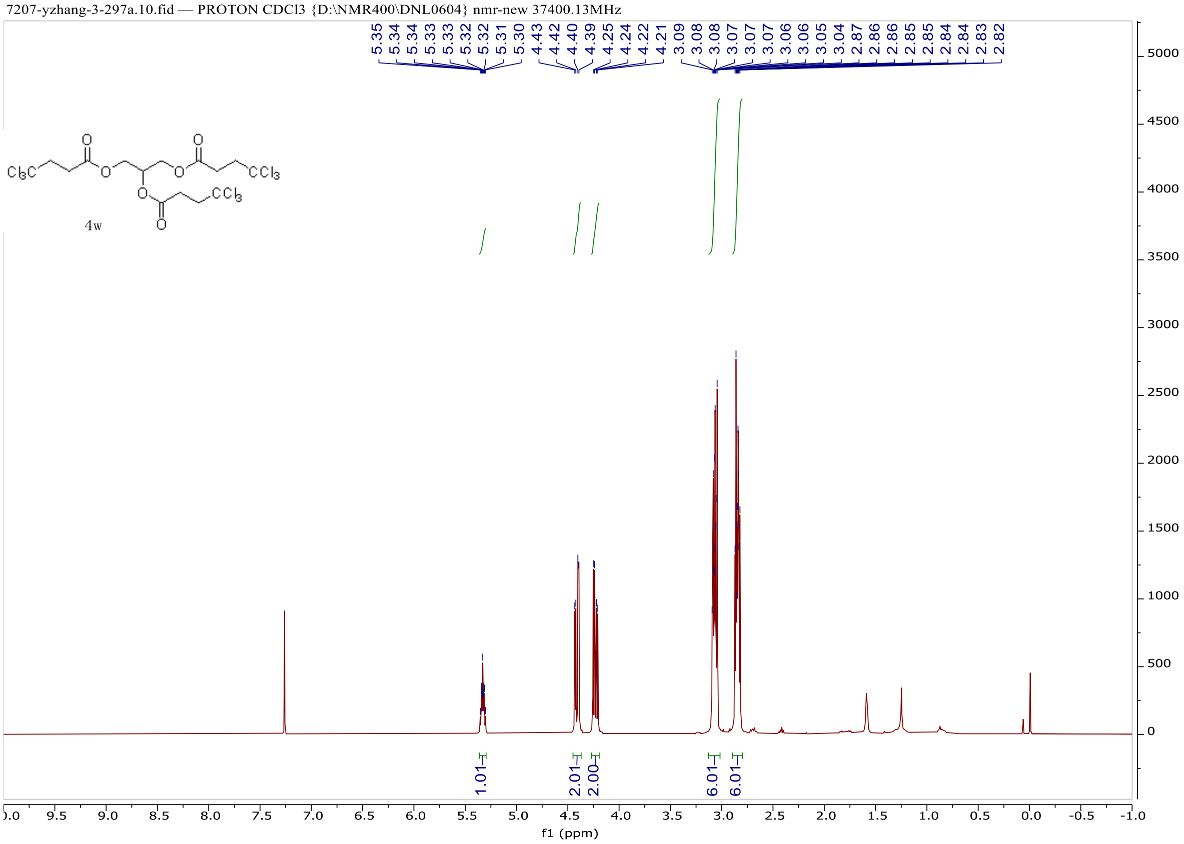

7207-yzhang-3-297a.11.fid — C13CPD CDCl3 {D:\NMR400\DNL0604} nmr-new 37100.62MHz

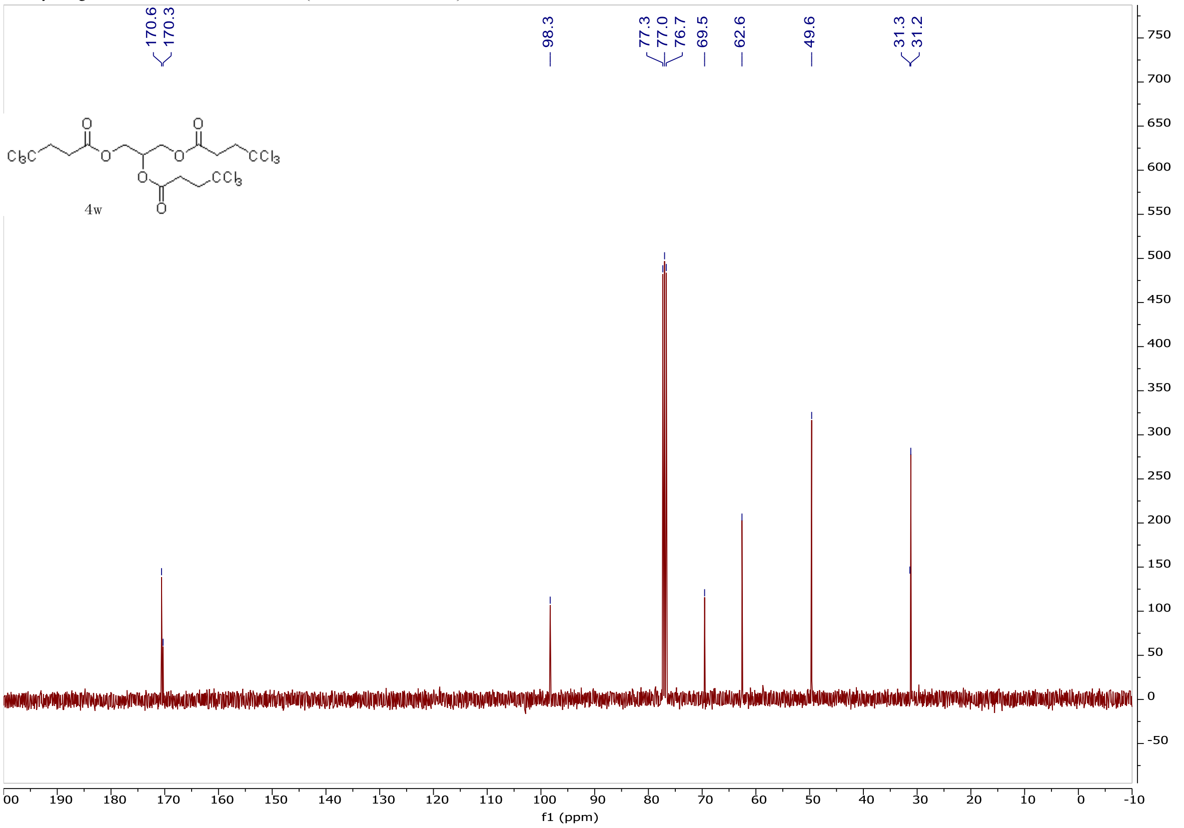

5a

6722-yzhang-3-177.11.fid — PROTON DMSO {D:\NMR400\DNL0604} nmr-new 40400.13MHz

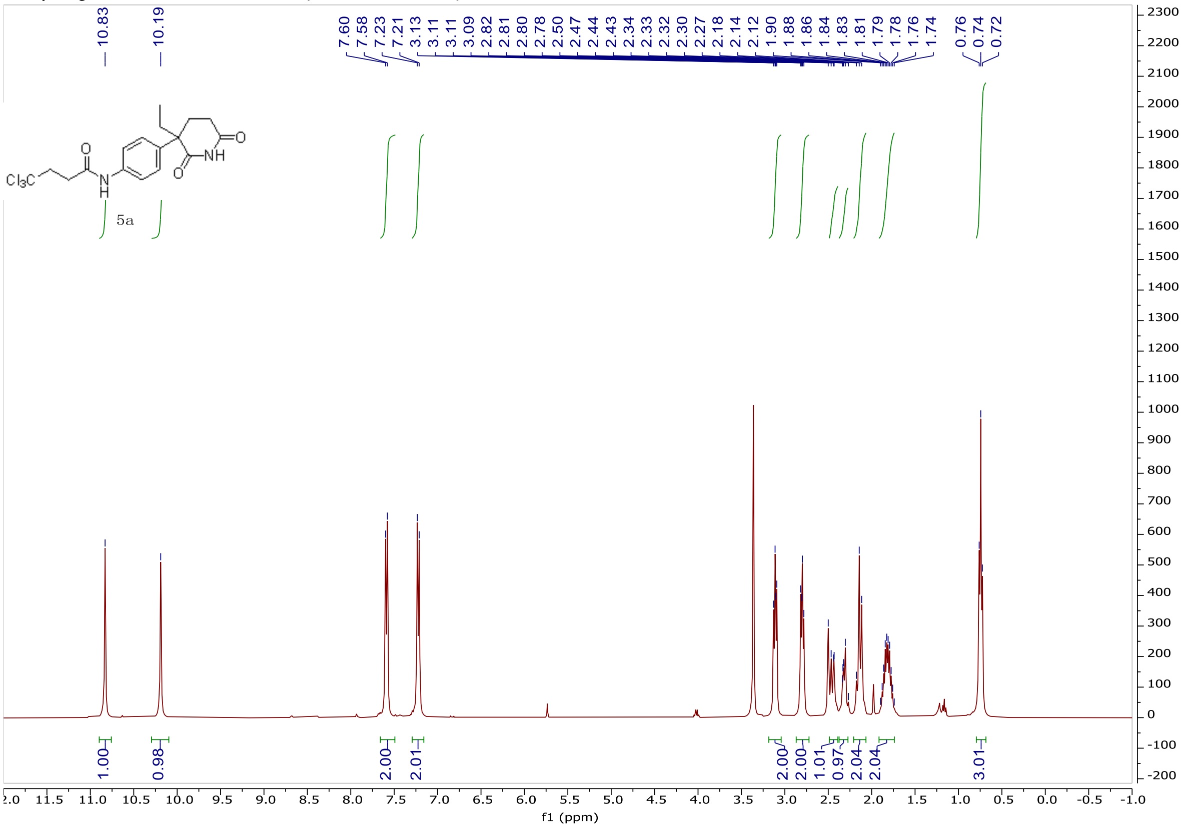

6722-yzhang-3-177.10.fid — C13CPD DMSO {D:\NMR400\DNL0604} nmr-new 40100.62MHz

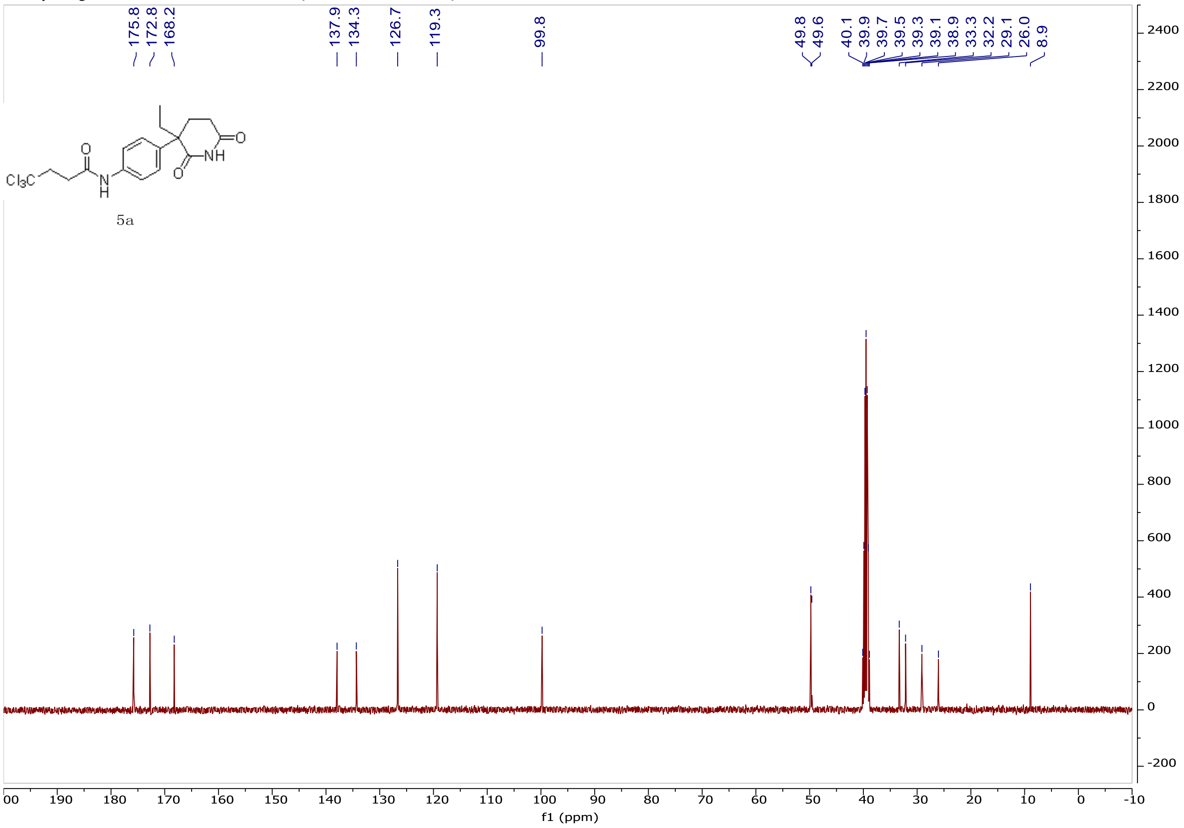

5b

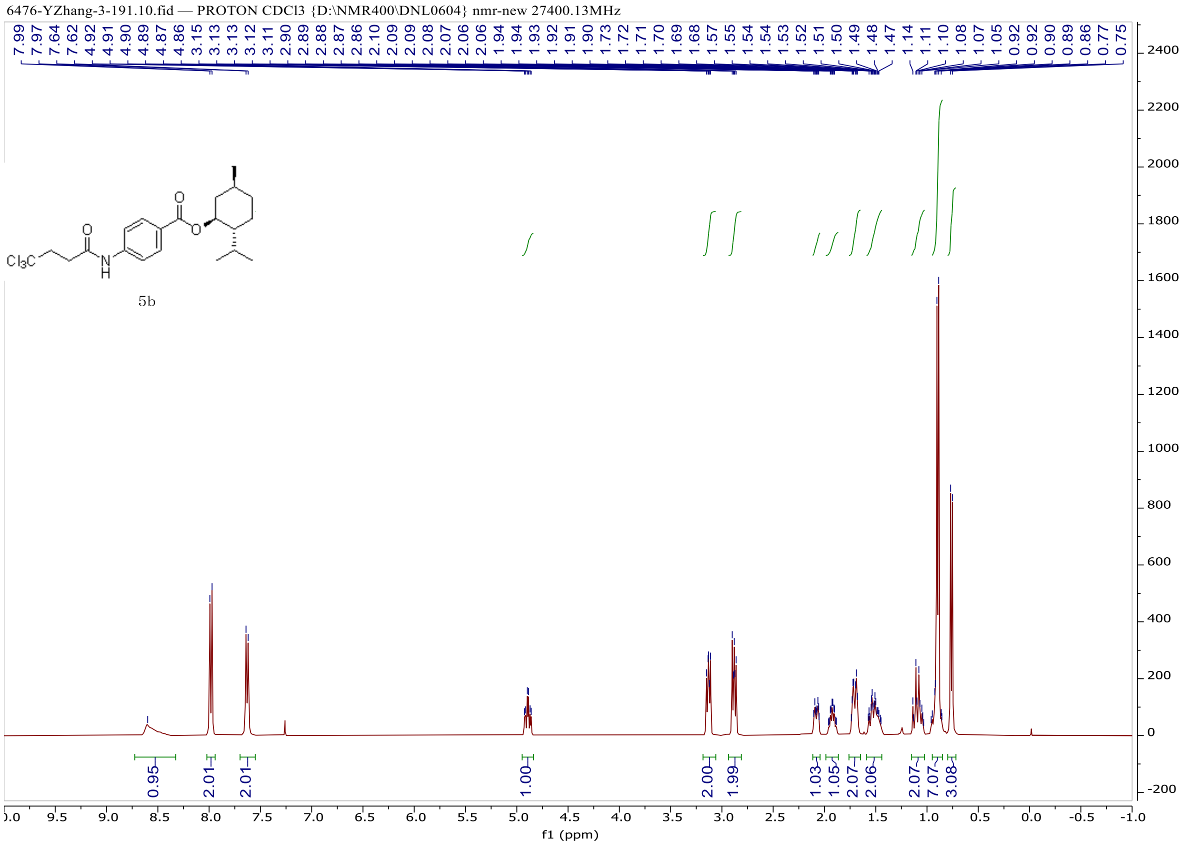
6476-YZhang-3-191.10.fid — PROTON CDCl3 {D:\NMR400\DNL0604} nmr-new 27400.13MHz
7.99 7.97 7.64 7.62 4.92 4.91 4.90 4.89 4.87 4.86 3.15 3.13 3.13 3.12 3.11 2.90 2.89 2.88 2.87 2.86 2.10 2.09 2.09 2.08 2.07 2.06 2.06 1.94 1.94 1.93 1.92 1.91 1.90 1.73 1.72 1.71 1.70 1.69 1.68 1.57 1.55 1.54 1.54 1.53 1.52 1.51 1.50 1.49 1.48 1.47 1.14 1.11 1.10 1.08 1.07 1.05 0.92 0.92 0.90 0.89 0.86 0.77 0.75
Cl3C
O
N
H
O
O
5b
0.95 2.01 2.01 1.00 2.00 1.99 1.03 1.05 2.07 2.06 2.07 7.07 3.08
f1 (ppm)

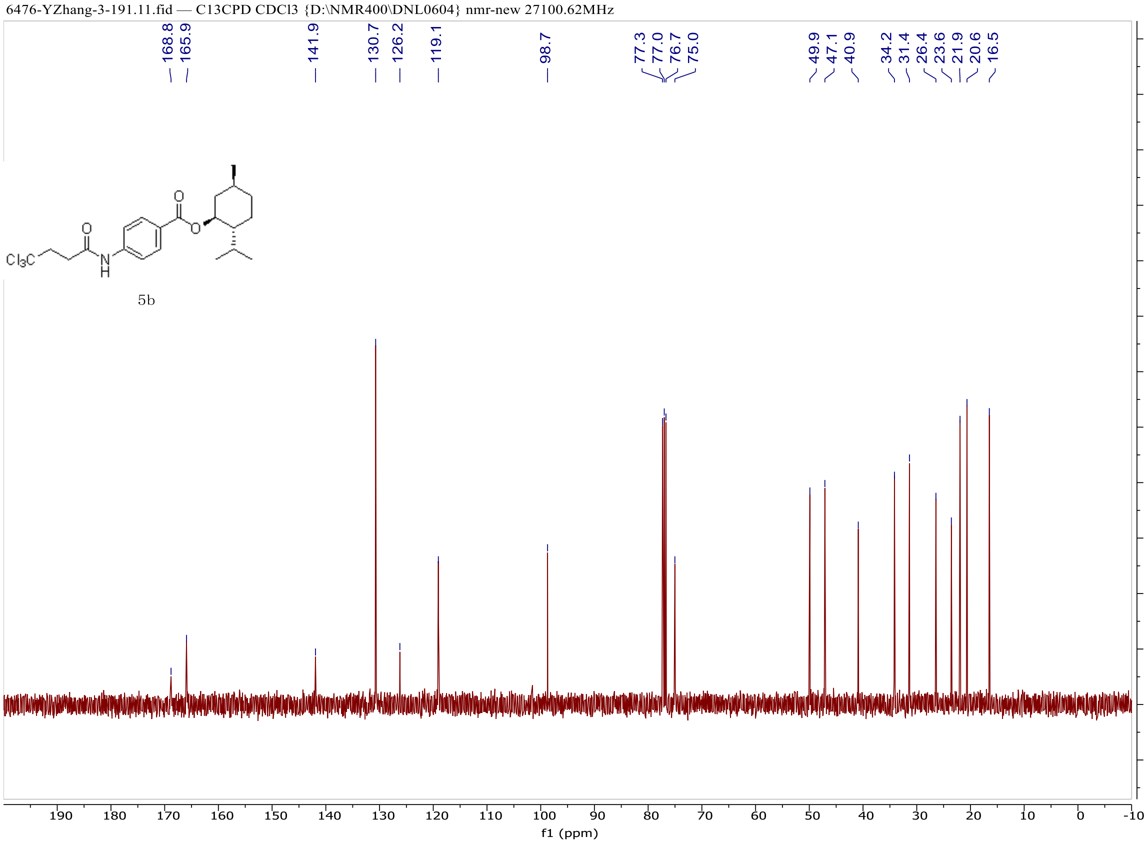
6476-YZhang-3-191.11.fid — C13CPD CDCl3 {D:\NMR400\DNL0604} nmr-new 27100.62MHz
168.8 165.9 141.9 130.7 126.2 119.1 98.7 77.3 77.0 76.7 75.0 49.9 47.1 40.9 34.2 31.4 26.4 23.6 21.9 20.6 16.5
Cl3C
O
N
H
O
O
5b
f1 (ppm)

5c

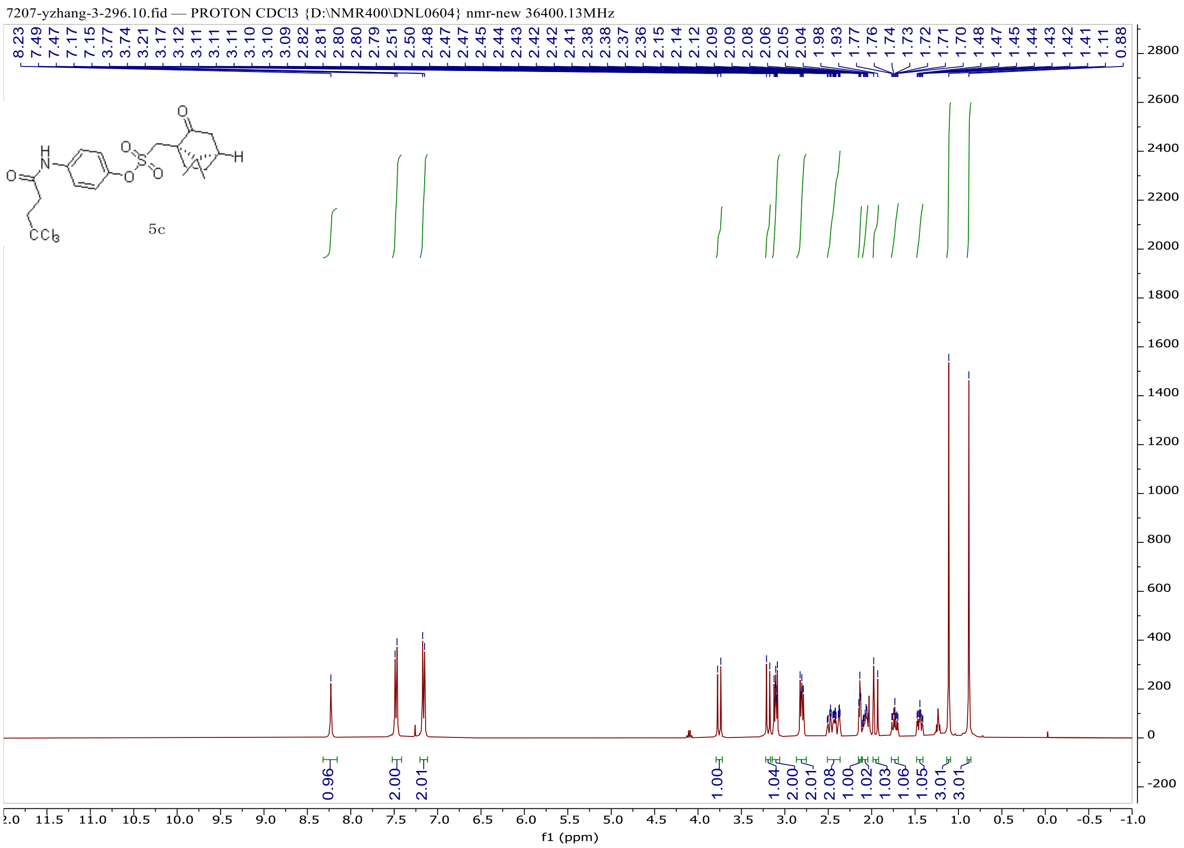
7207-yzhang-3-296.10.fid — PROTON CDCl3 {D:\NMR400\DNL0604} nmr-new 36400.13MHz
8.23 7.49 7.47 7.17 7.15 3.77 3.74 3.21 3.17 3.12 3.11 3.11 3.11 3.10 3.10 3.09 2.82 2.81 2.80 2.80 2.79 2.51 2.50 2.48 2.47 2.47 2.45 2.44 2.43 2.42 2.42 2.41 2.38 2.38 2.37 2.36 2.15 2.14 2.12 2.09 2.09 2.08 2.06 2.05 2.04 1.98 1.93 1.77 1.76 1.74 1.73 1.72 1.71 1.70 1.48 1.47 1.45 1.44 1.43 1.42 1.41 1.11 0.88
5c
0.96 2.00 2.01 1.00 1.04 2.00 2.01 2.08 1.00 1.02 1.03 1.06 1.05 3.01 3.01
f1 (ppm)

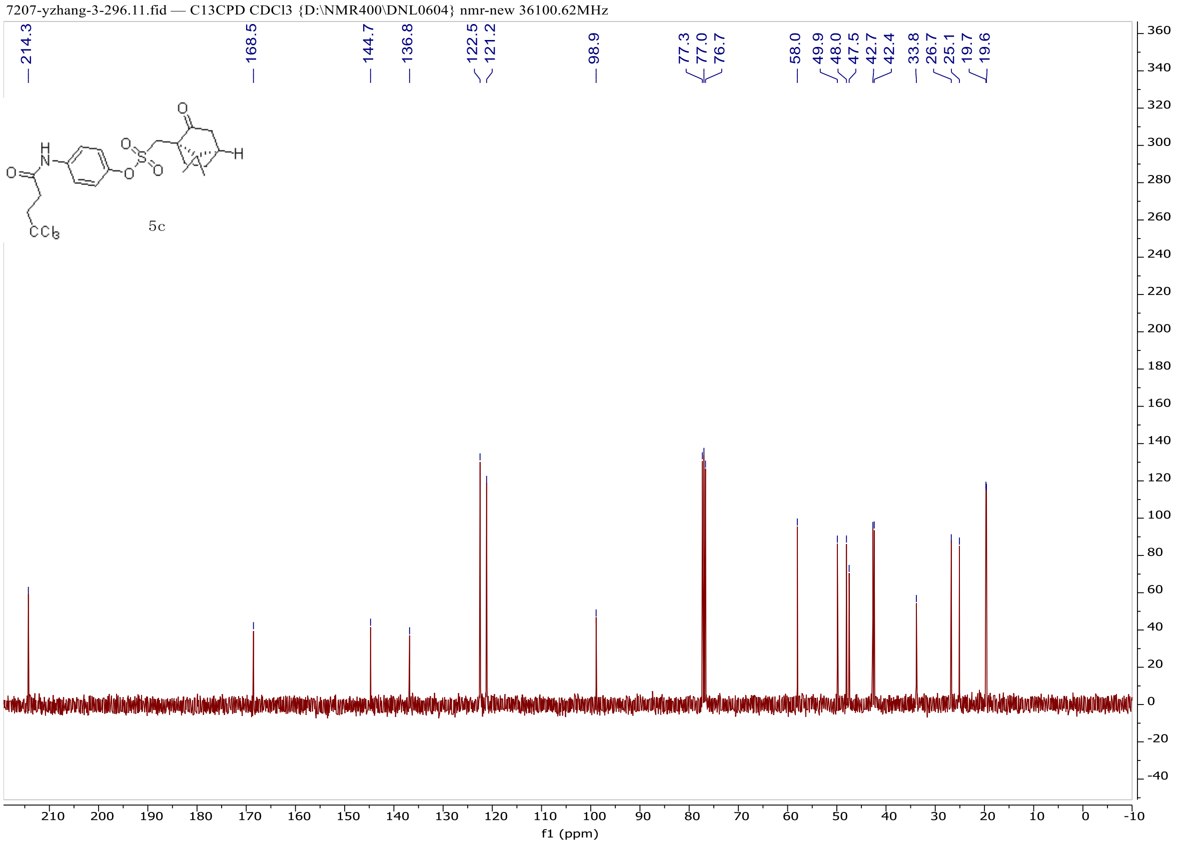
7207-yzhang-3-296.11.fid — C13CPD CDCl3 {D:\NMR400\DNL0604} nmr-new 36100.62MHz
214.3 168.5 144.7 136.8 122.5 121.2 98.9 77.3 77.0 76.7 58.0 49.9 48.0 47.5 42.7 42.4 33.8 26.7 25.1 19.7 19.6
5c
f1 (ppm)

5d

6477-YZhang-3-175.10.fid — PROTON DMSO {D:\NMR400\DNL0604} nmr-new 4400.13MHz

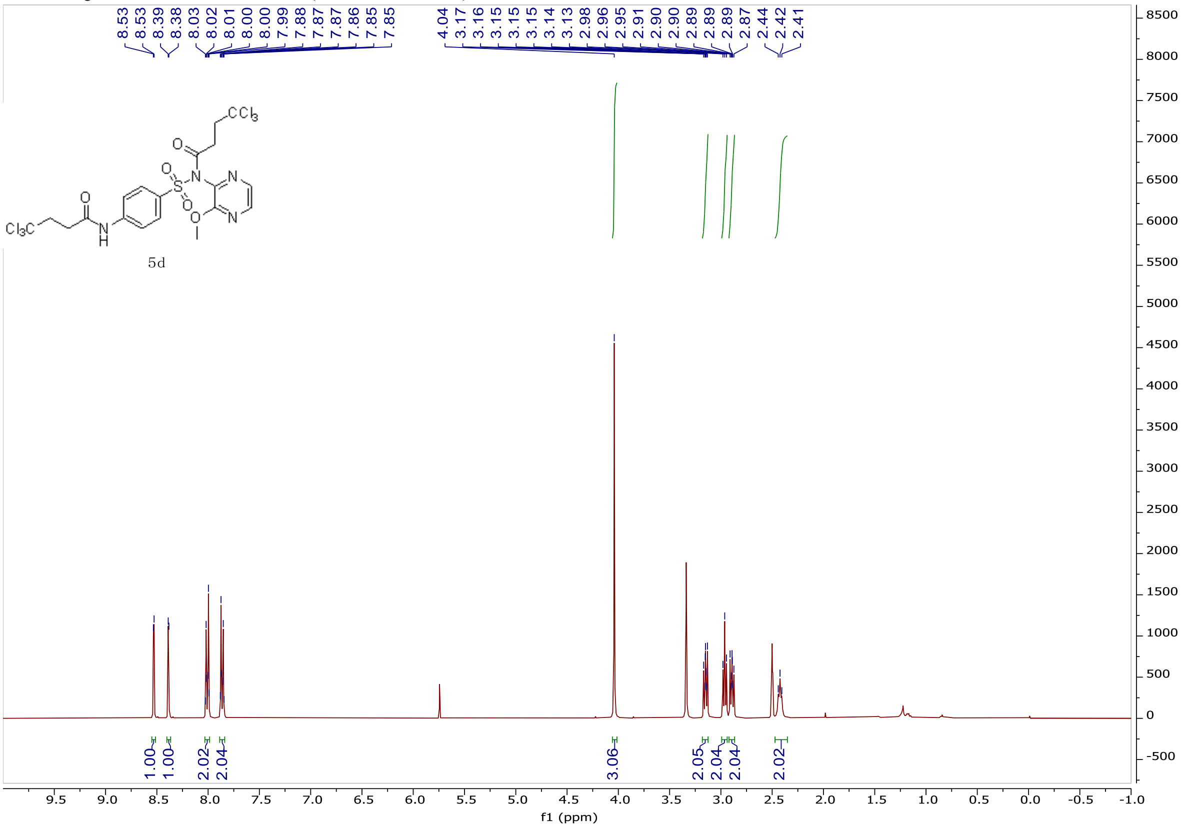

6477-YZhang-3-175.11.fid — C13CPD DMSO {D:\NMR400\DNL0604} nmr-new 4100.62MHz

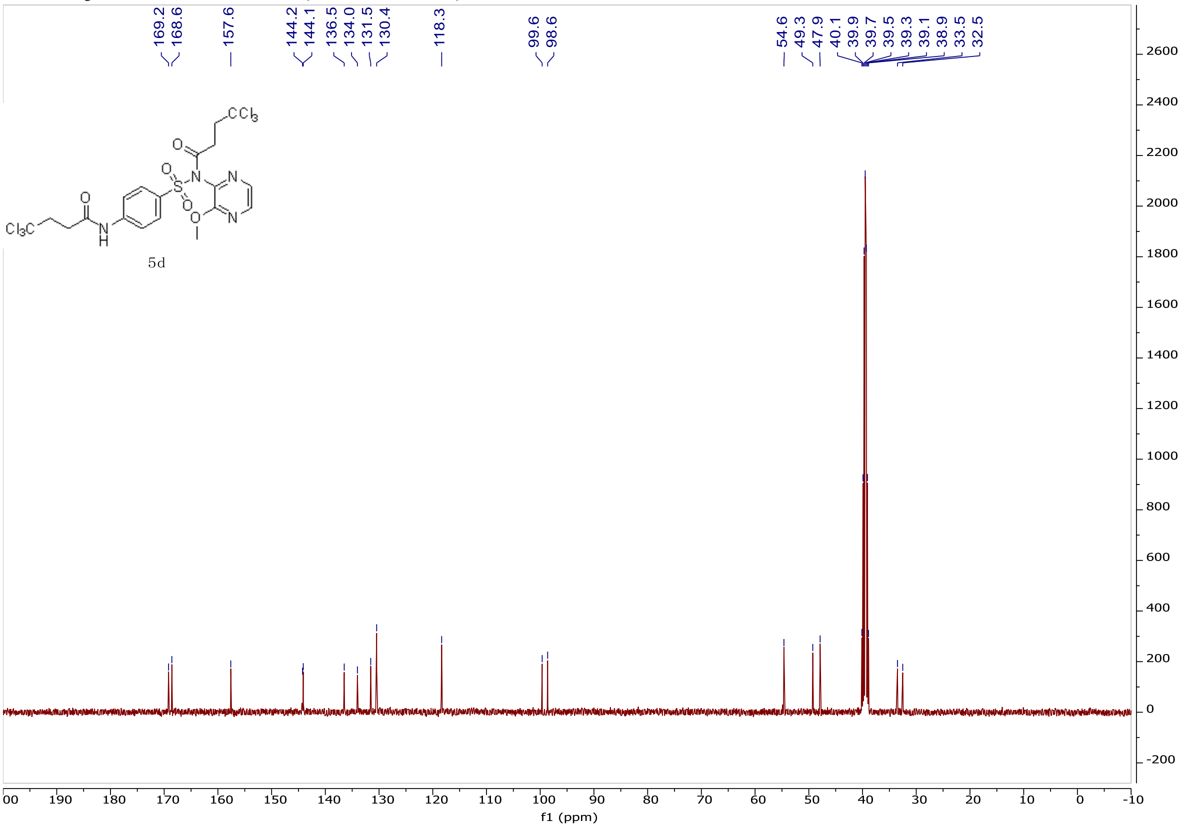

5e

6925-yzhang-3-256.10.fid — PROTON CDCl3 {D:\NMR400\DNL0604} nmr-new 17400.13MHz

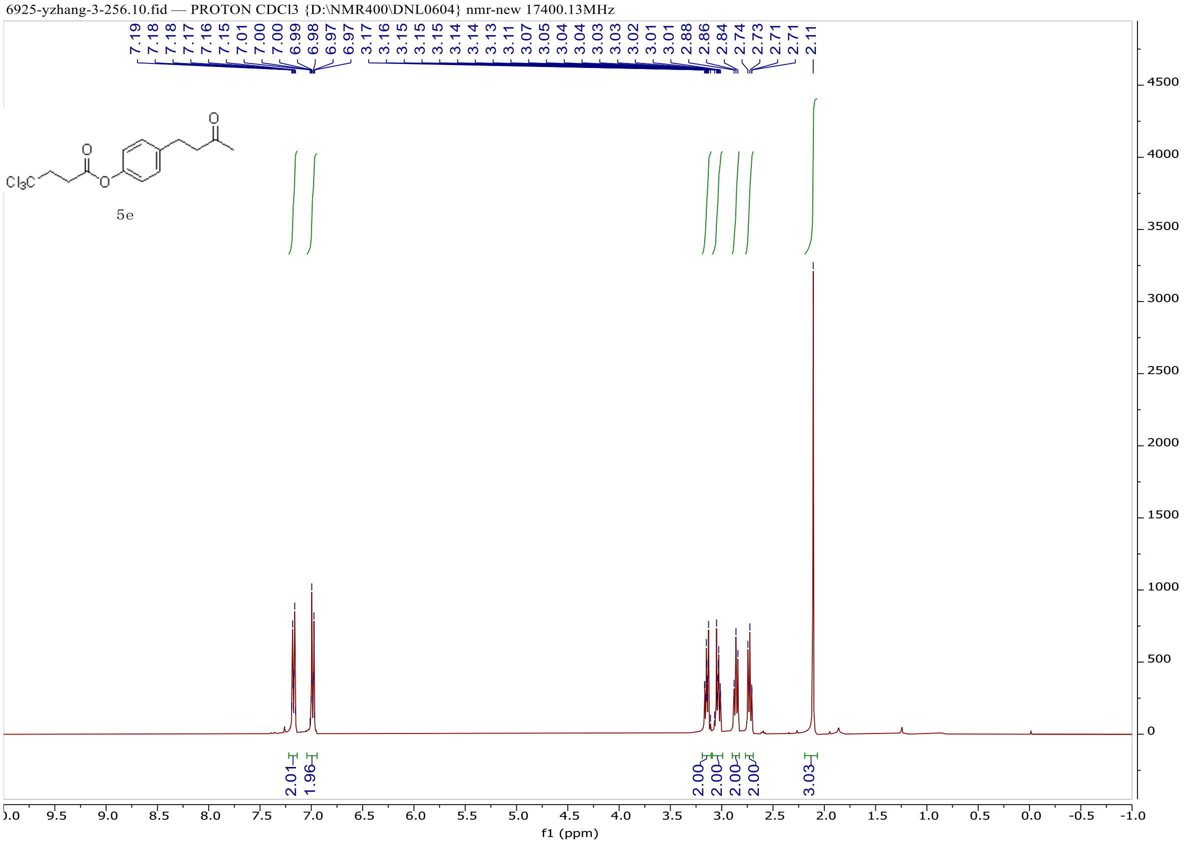

6925-yzhang-3-256.11.fid — C13CPD CDCl3 {D:\NMR400\DNL0604} nmr-new 17100.62MHz

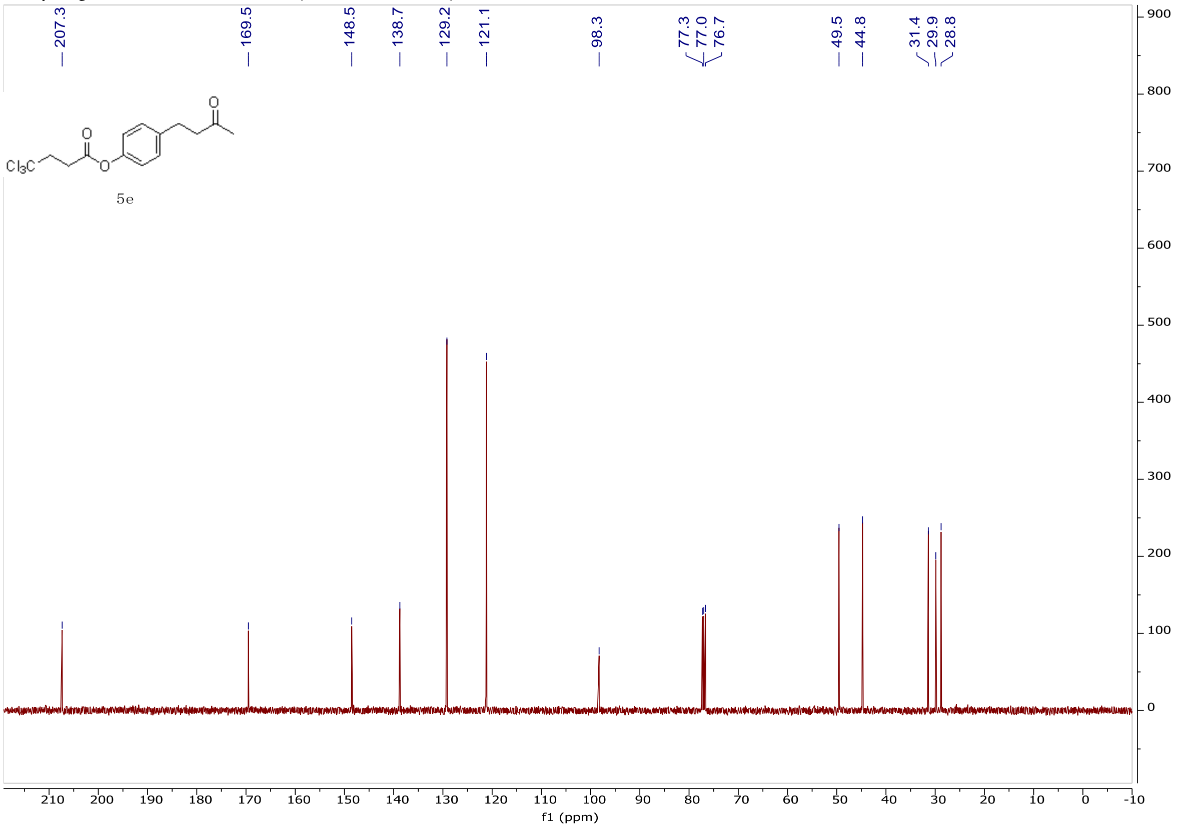

5f

6684-yzhang-3-204.10.fid — PROTON CDCl3 {D:\NMR400\DNL0604} nmr-new 5400.13MHz

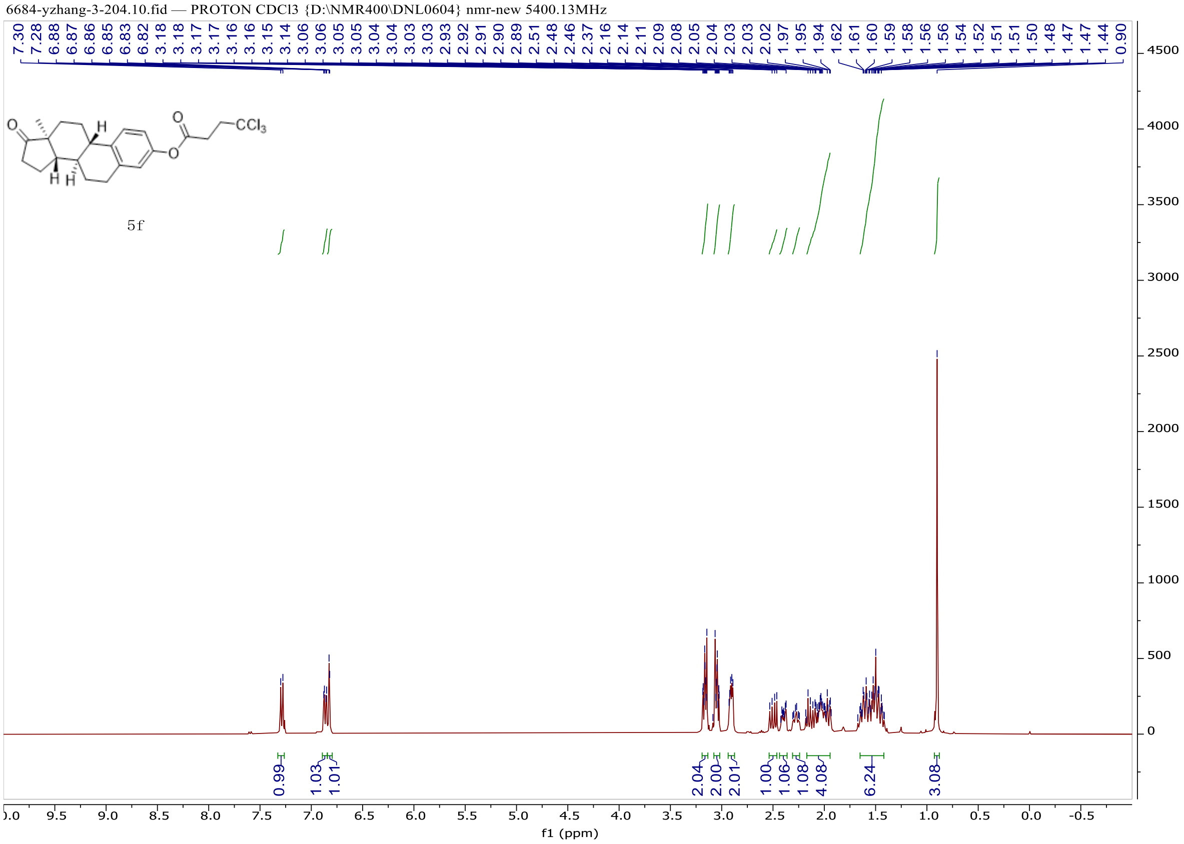

6684-yzhang-3-204.11.fid — C13CPD CDCl3 {D:\NMR400\DNL0604} nmr-new 5100.62MHz

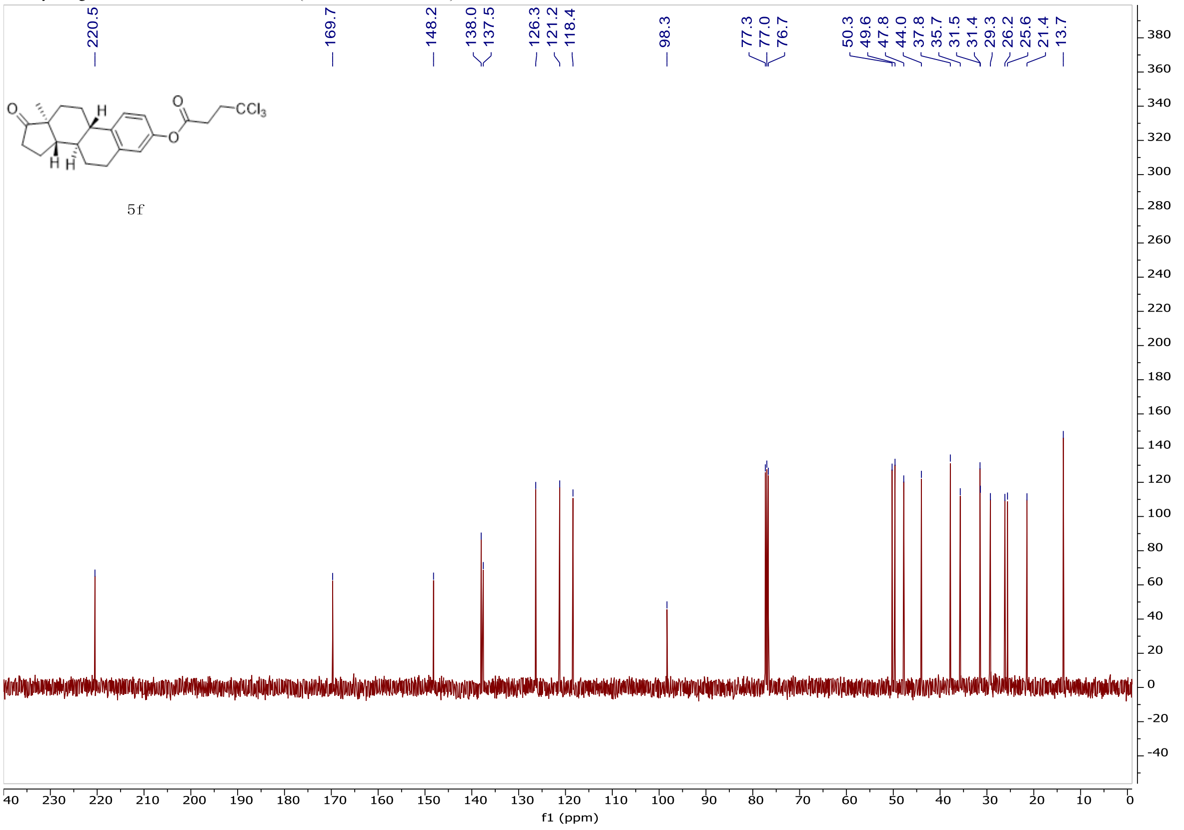

5g

6722-yzhang-3-201.10.fid — PROTON CDCl3 {D:\NMR400\DNL0604} nmr-new 39400.13MHz

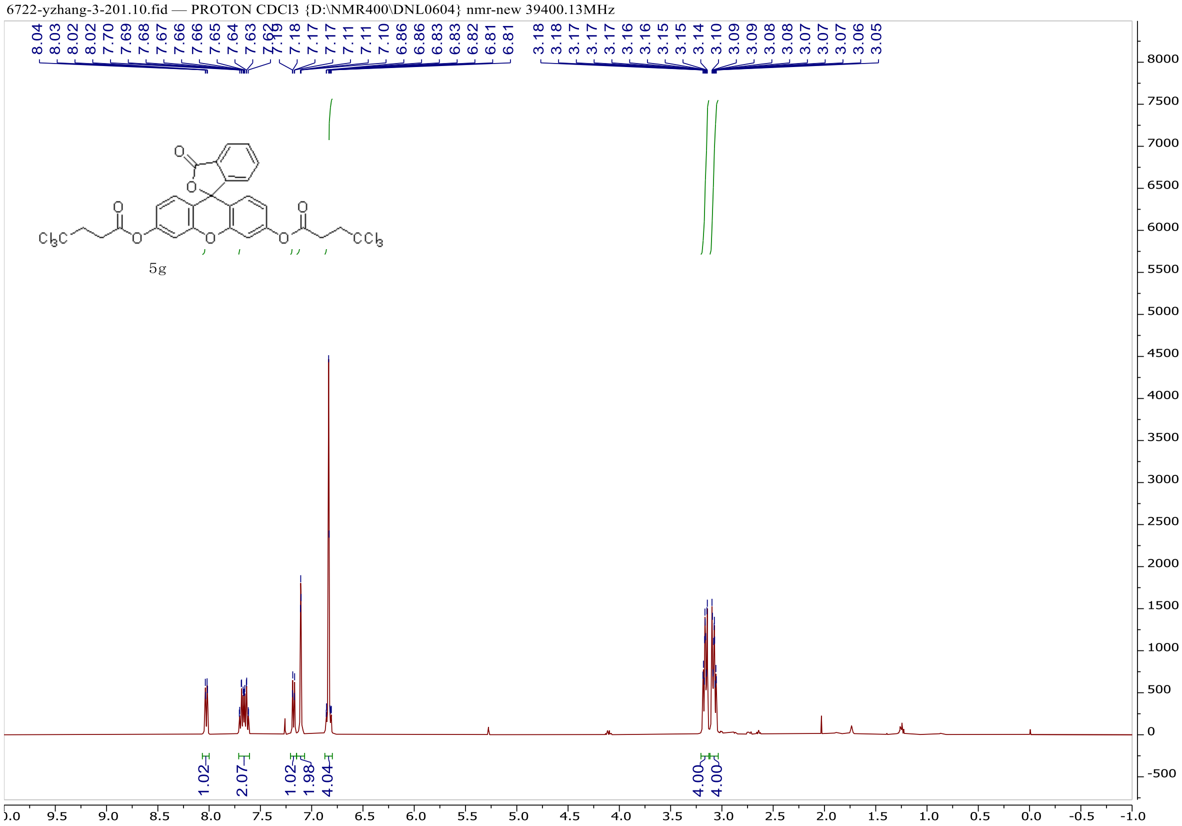

6722-yzhang-3-201.11.fid — C13CPD CDCl3 {D:\NMR400\DNL0604} nmr-new 39100.62MHz

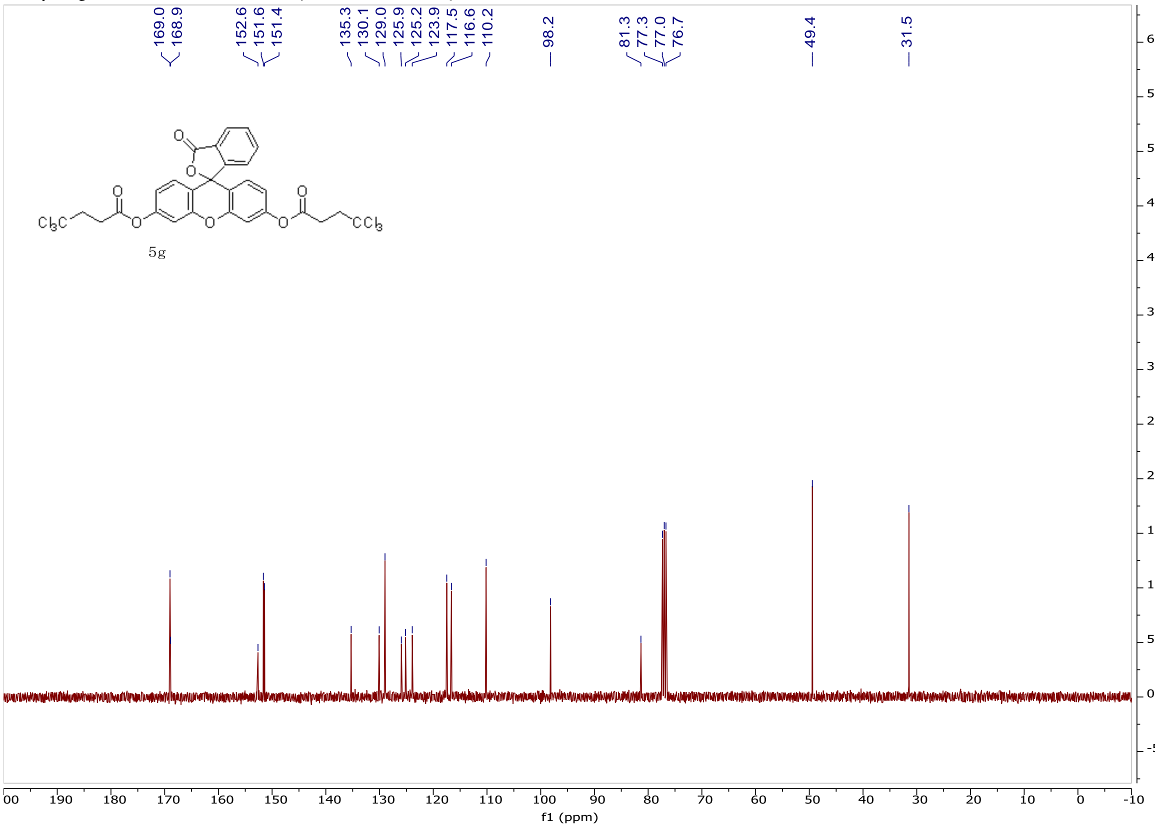

5h

6242-YZhang-3-150.10.fid — PROTON CDCl3 {D:\NMR400\DNL0604} nmr-new 40400.13MHz

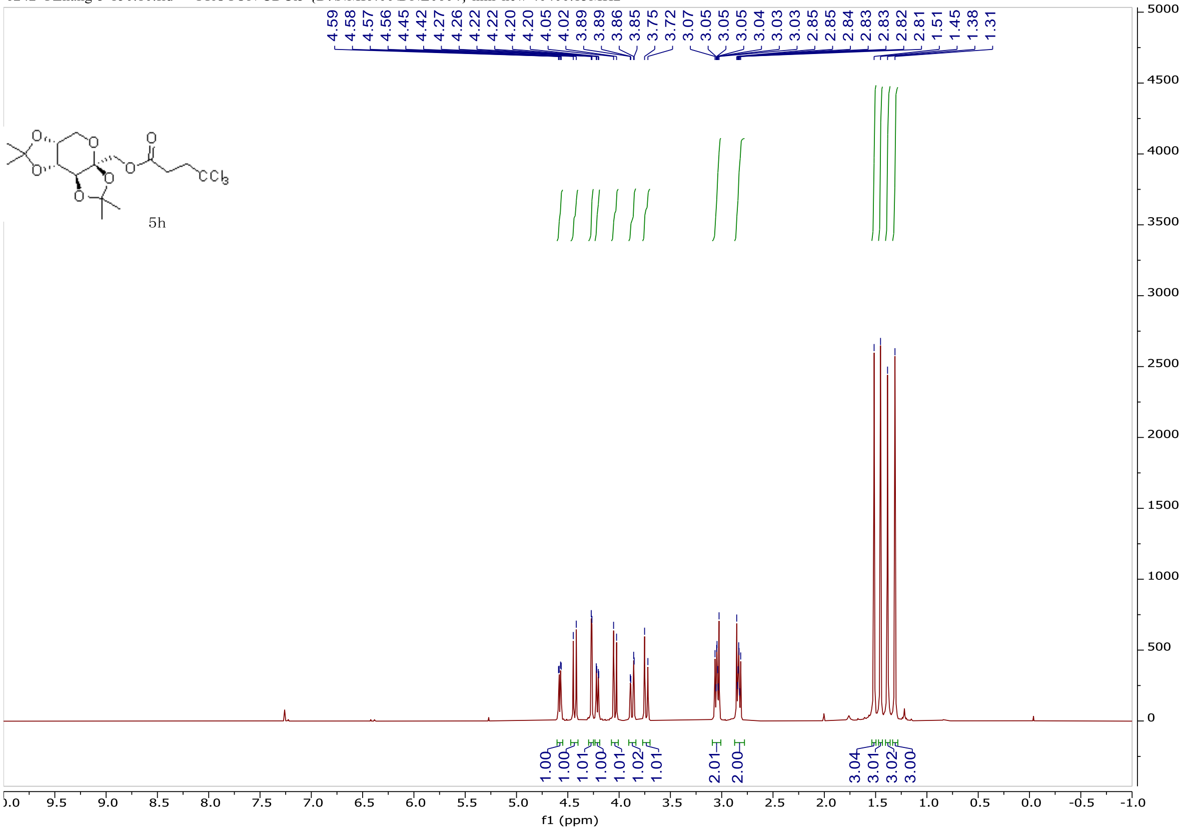

6242-YZhang-3-150.11.fid — C13CPD CDCl3 {D:\NMR400\DNL0604} nmr-new 40100.62MHz

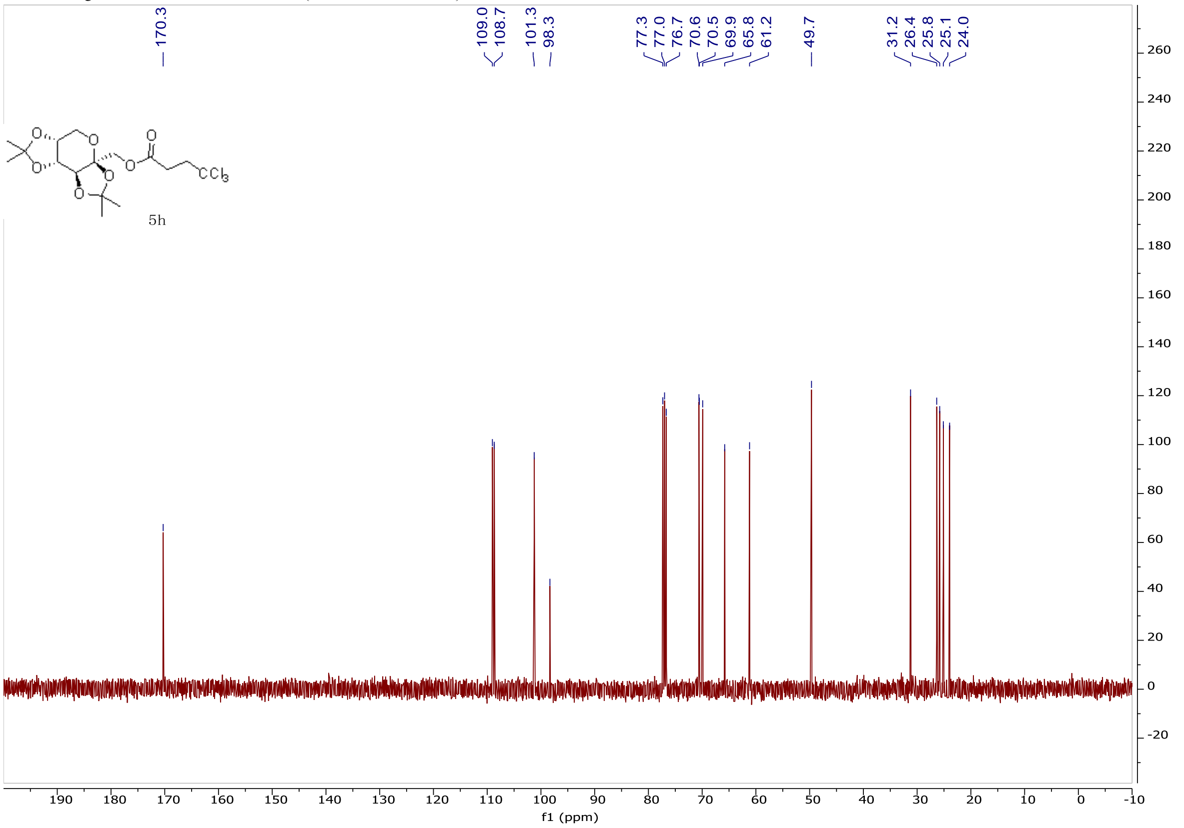

5i

6476-YZhang-3-192.10.fid — PROTON CDCl3 {D:\NMR400\DNL0604} nmr-new 28400.13MHz

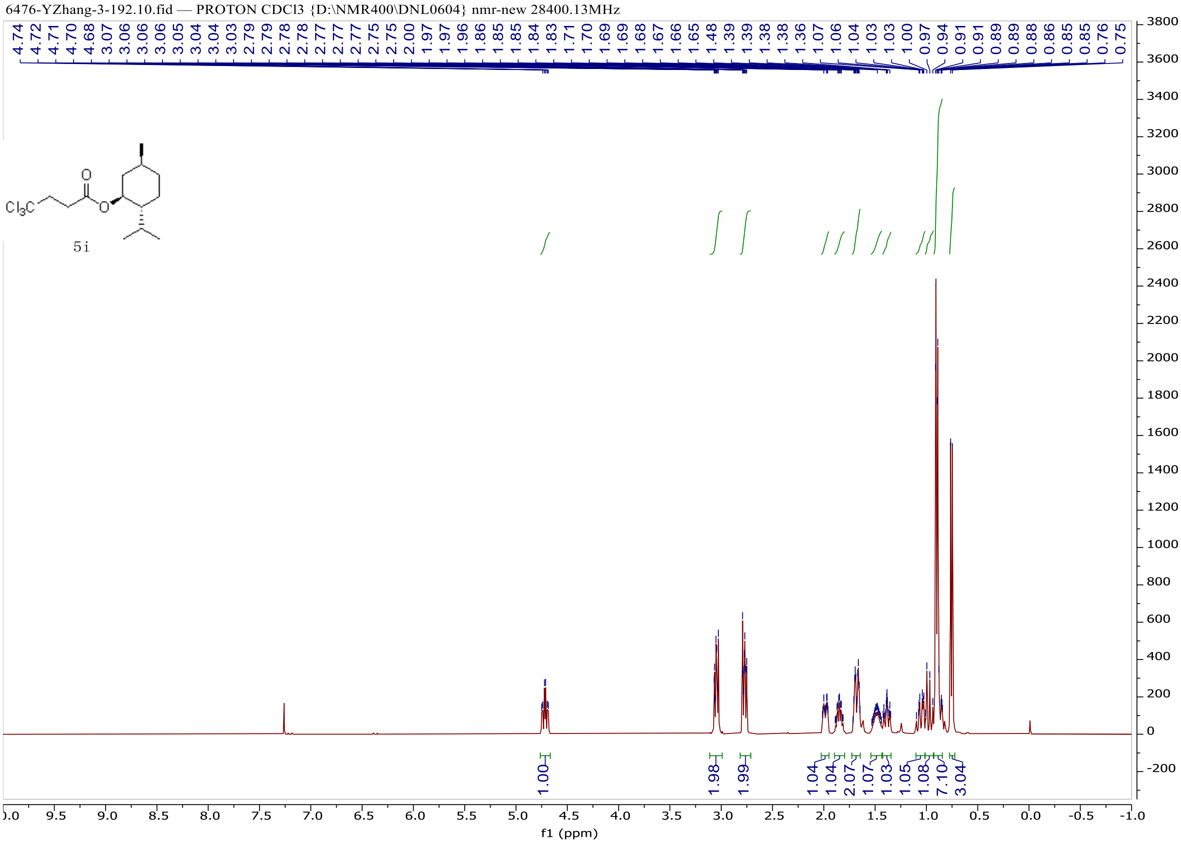

6476-YZhang-3-192.11.fid — C13CPD CDCl3 {D:\NMR400\DNL0604} nmr-new 28100.62MHz

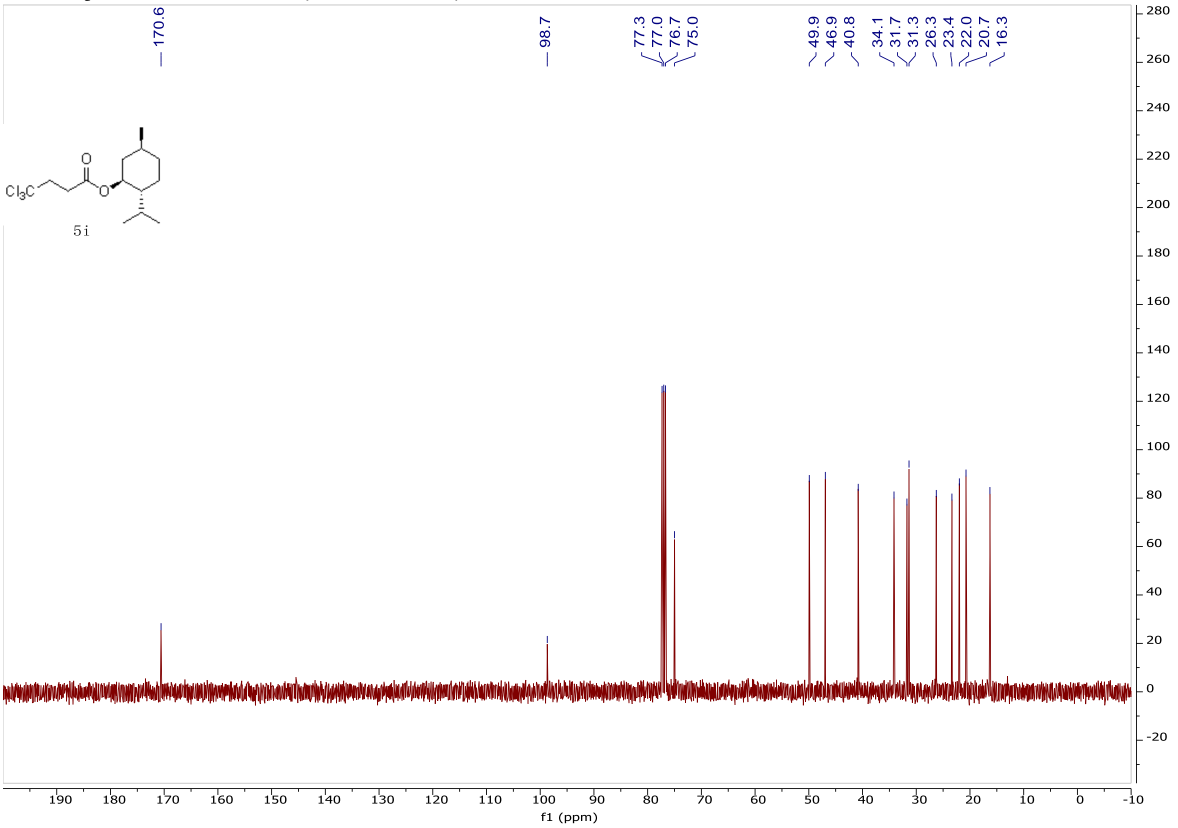

5j

6950-yzhang-3-241.10.fid — PROTON CDCl3 {D:\NMR400\DNL0604} nmr-new 40400.13MHz

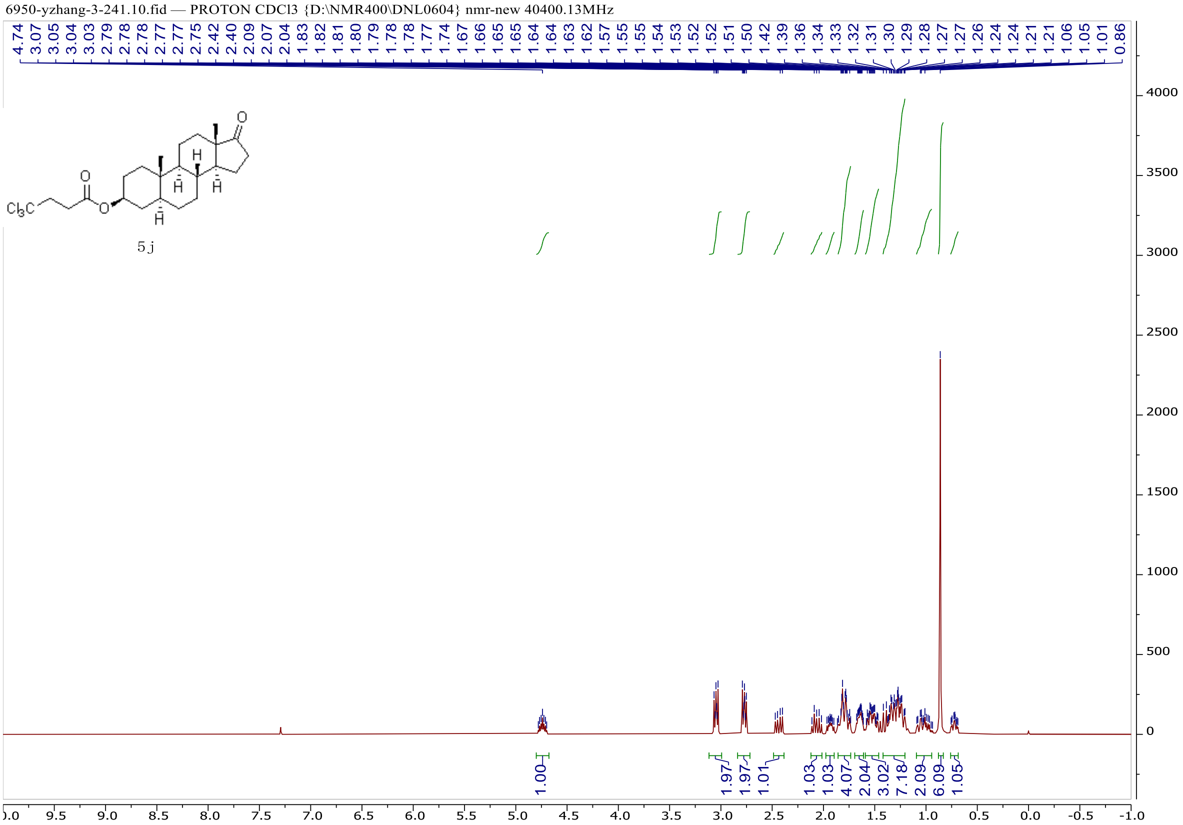

6950-yzhang-3-241.11.fid — C13CPD CDCl3 {D:\NMR400\DNL0604} nmr-new 40100.62MHz

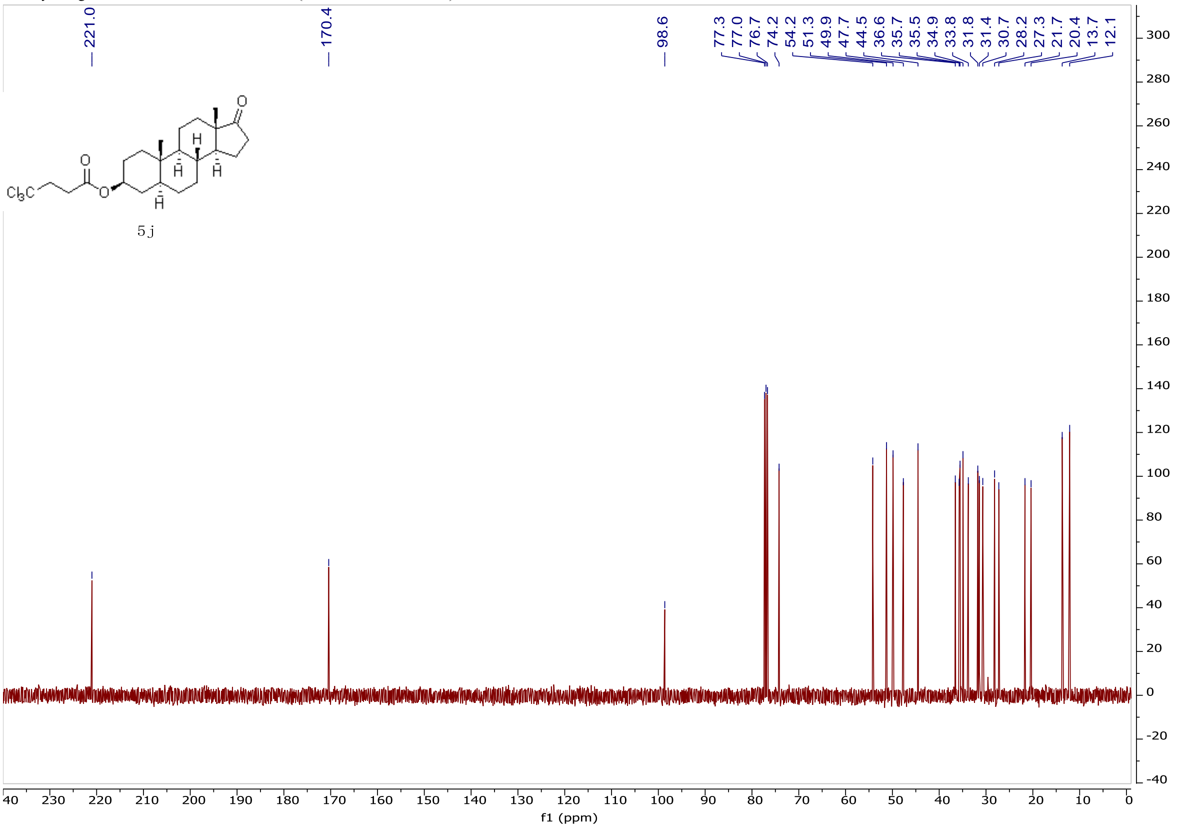

5k

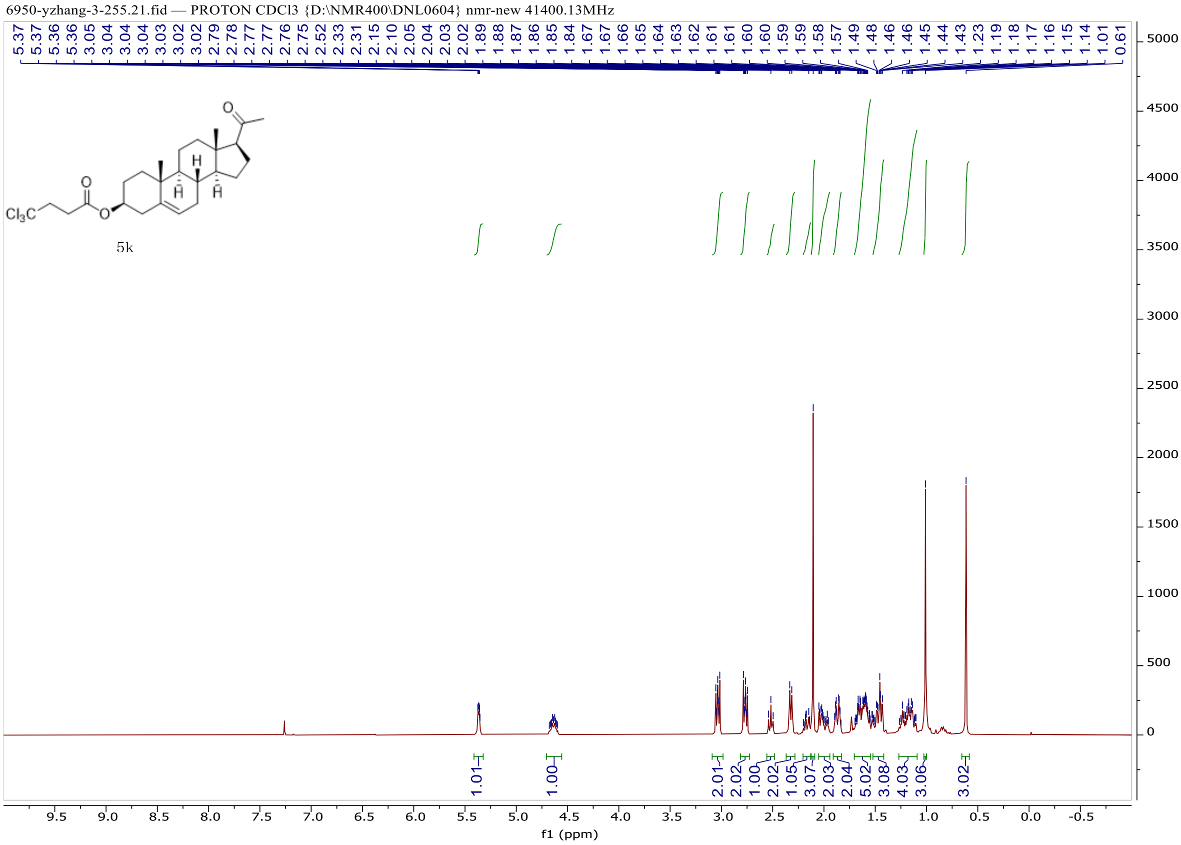
6950-yzhang-3-255.21.fid — PROTON CDCl3 {D:\NMR400\DNL0604} nmr-new 41400.13MHz
5.37 5.37 5.36 5.36 3.05 3.04 3.04 3.04 3.03 3.02 3.02 2.79 2.78 2.77 2.77 2.76 2.75 2.52 2.33 2.31 2.15 2.10 2.05 2.04 2.03 2.02 1.89 1.88 1.87 1.86 1.85 1.84 1.67 1.67 1.66 1.65 1.64 1.63 1.62 1.61 1.61 1.60 1.60 1.59 1.59 1.58 1.57 1.49 1.48 1.46 1.46 1.45 1.44 1.43 1.23 1.19 1.18 1.17 1.16 1.15 1.14 1.01 0.61
5k
1.01 1.00 2.01 2.02 1.00 2.02 1.05 3.07 2.03 2.04 5.02 3.08 4.03 3.06 3.02
f1 (ppm)

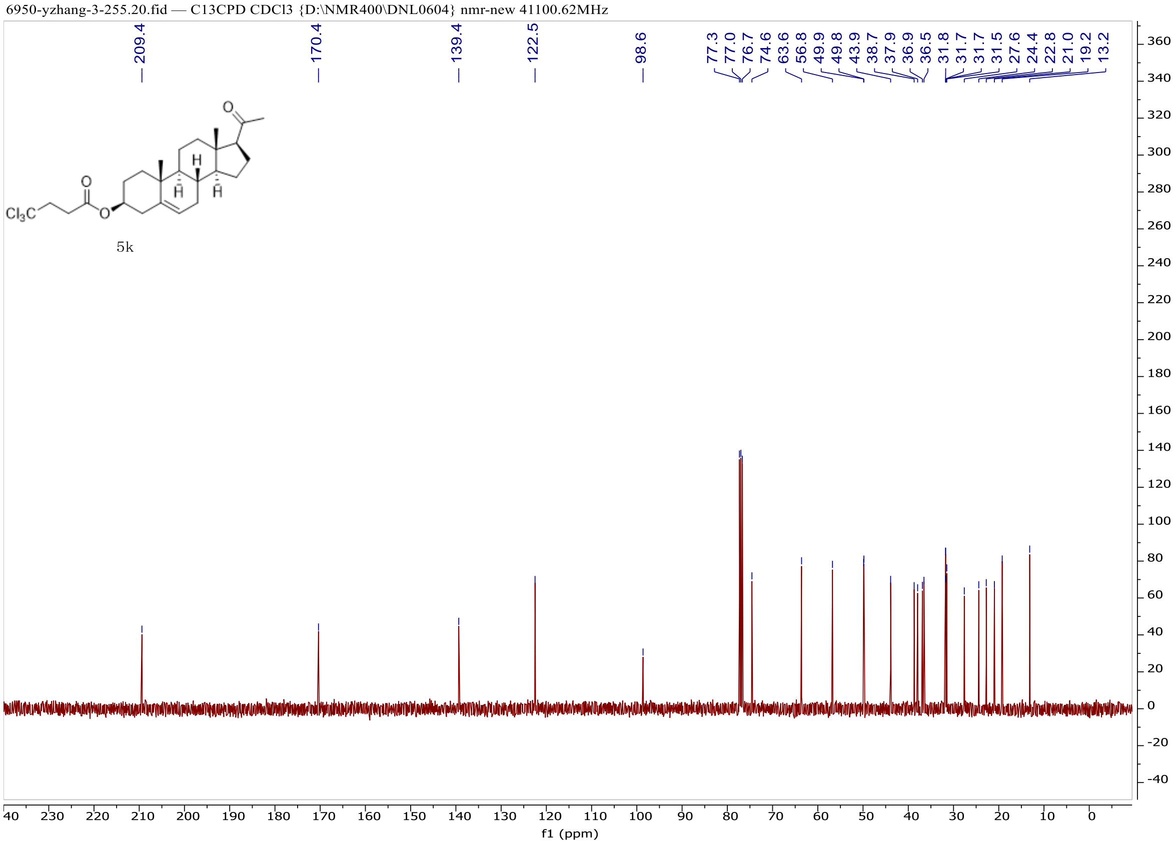
6950-yzhang-3-255.20.fid — C13CPD CDCl3 {D:\NMR400\DNL0604} nmr-new 41100.62MHz
209.4 170.4 139.4 122.5 98.6 77.3 77.0 76.7 74.6 63.6 56.8 49.9 49.8 43.9 38.7 37.9 36.9 36.5 31.8 31.7 31.5 27.6 24.4 22.8 21.0 19.2 13.2
5k
f1 (ppm)

5l

6285-YZhang-3-165.10.fid — PROTON CDCl3 {D:\NMR400\DNL0604} nmr-new 8400.13MHz

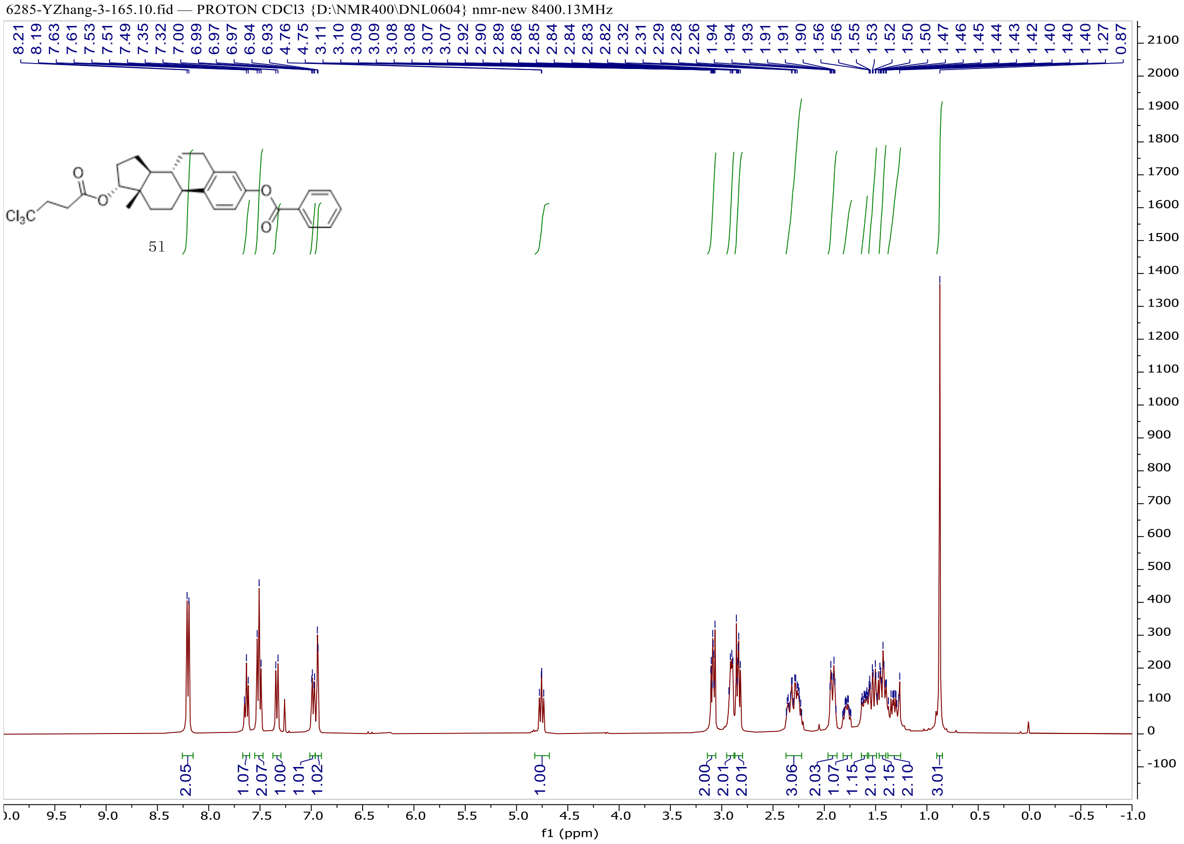

6285-YZhang-3-165.11.fid — C13CPD CDCl3 {D:\NMR400\DNL0604} nmr-new 8100.62MHz

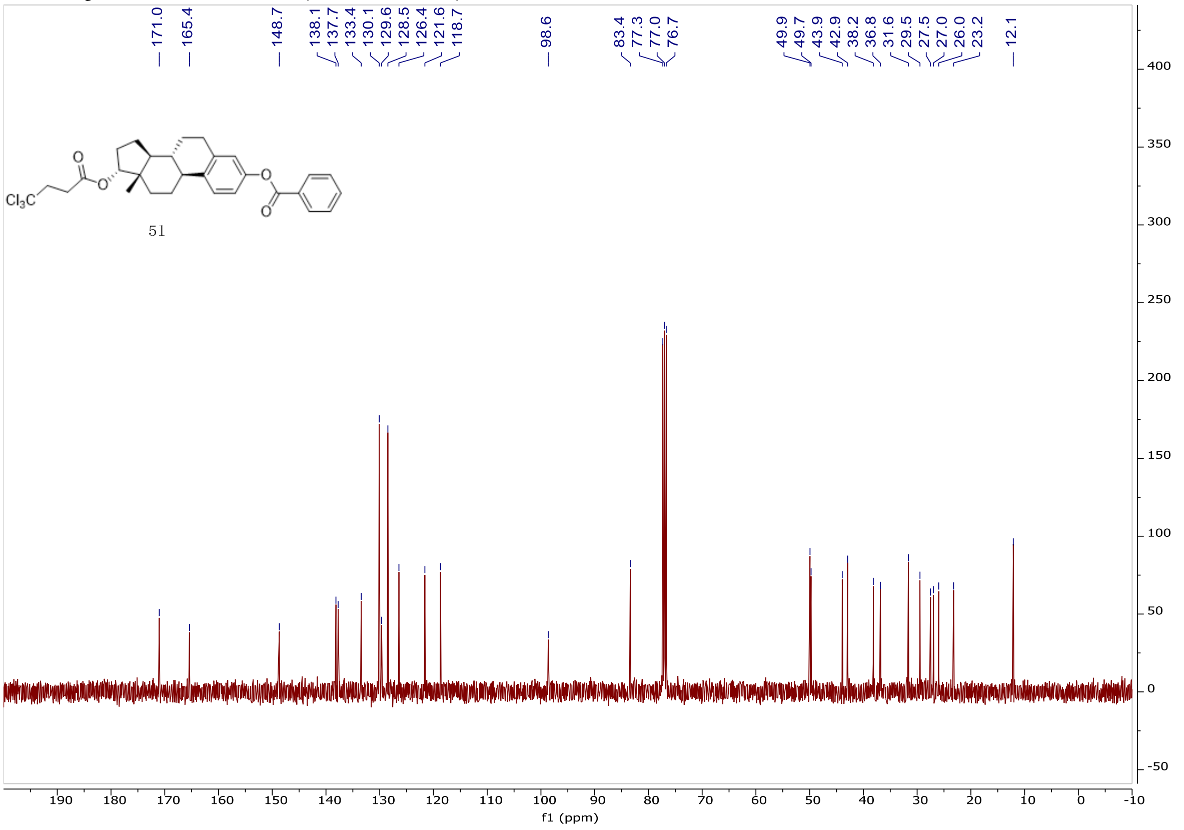

5m

7207-yzhang-3-298.10.fid — PROTON CDCl3 {D:\NMR400\DNL0604} nmr-new 39400.13MHz

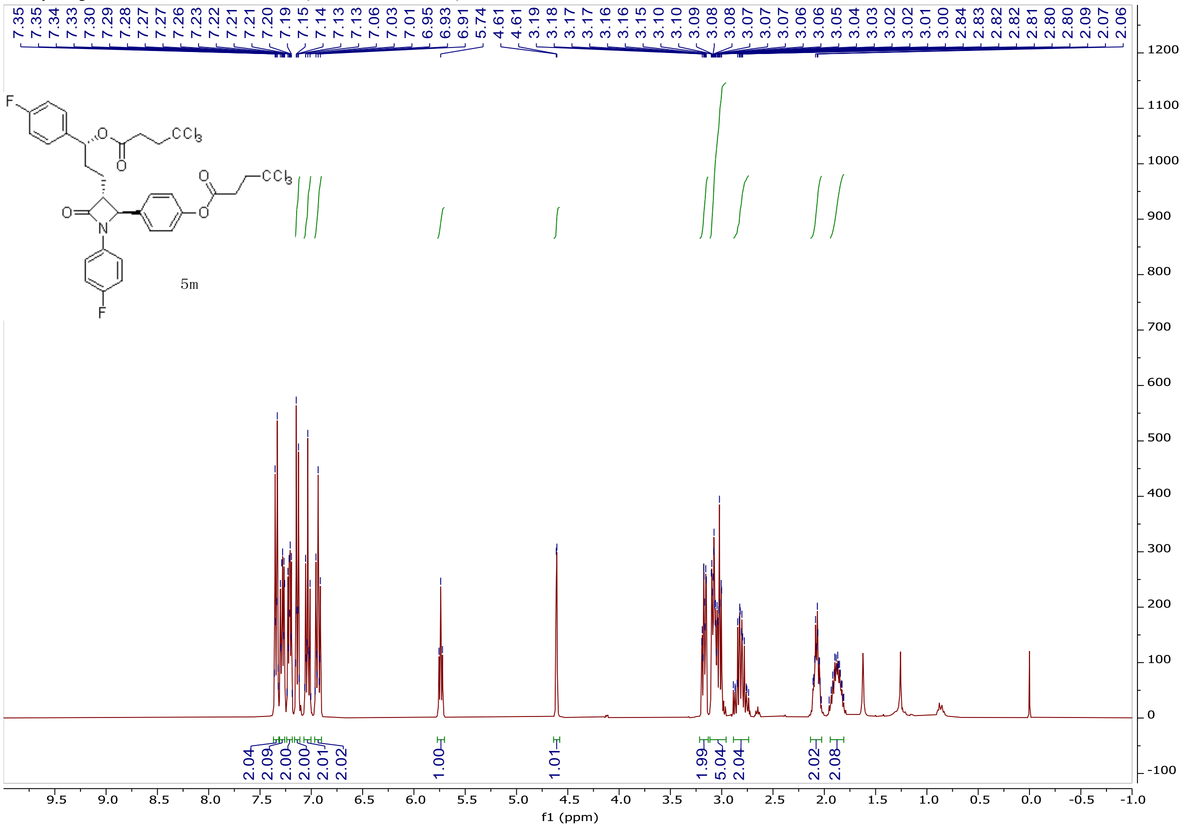

7207-yzhang-3-298.11.fid — C13CPD CDCl3 {D:\NMR400\DNL0604} nmr-new 39100.62MHz

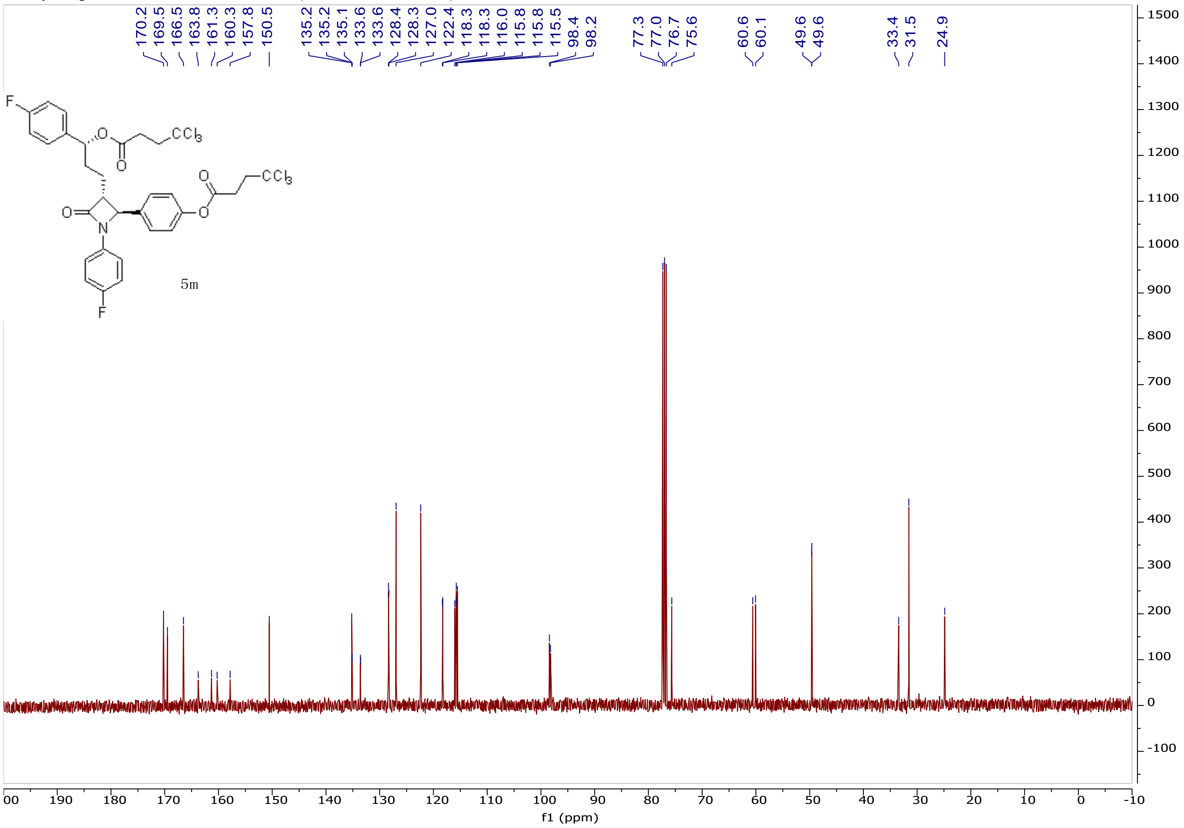

7207-yzhang-3-298.12.fid — F19CPD CDCl3 {D:\NMR400\DNL0604} nmr-new 39376.46MHz

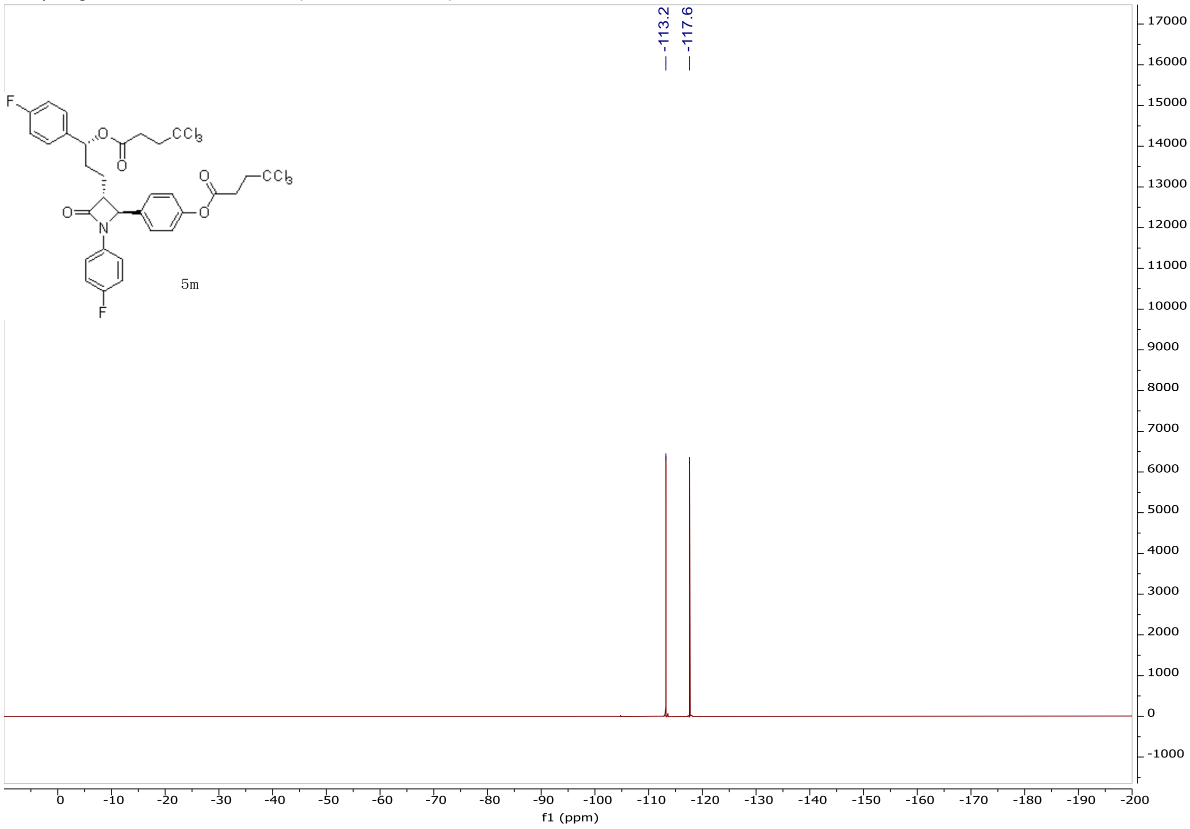

6a

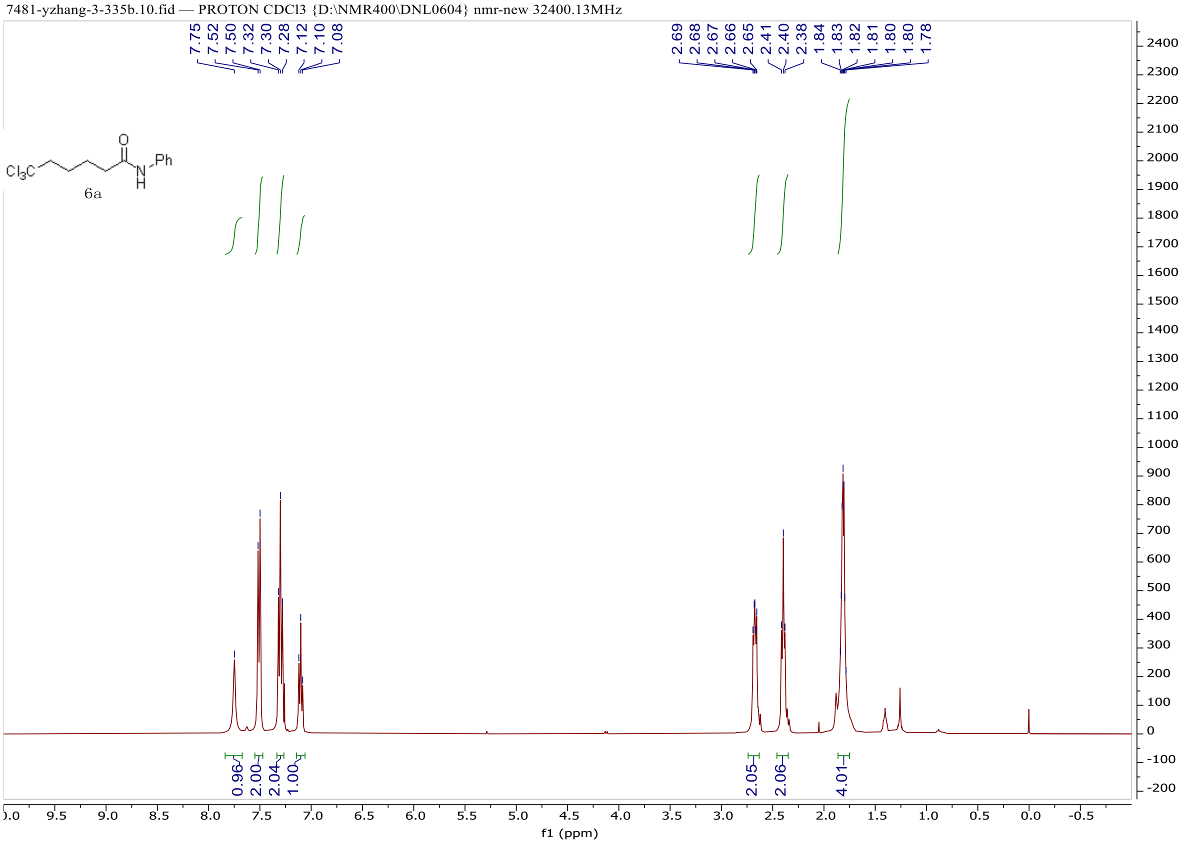
7481-yzhang-3-335b.10.fid — PROTON CDCl3 {D:\NMR400\DNL0604} nmr-new 32400.13MHz
7.75 7.52 7.50 7.32 7.30 7.28 7.12 7.10 7.08
2.69 2.68 2.67 2.66 2.65 2.41 2.40 2.38 1.84 1.83 1.82 1.81 1.80 1.80 1.78
Cl3C
Ph
N
H
O
6a
0.96 2.00 2.04 1.00 2.05 2.06 4.01
f1 (ppm)

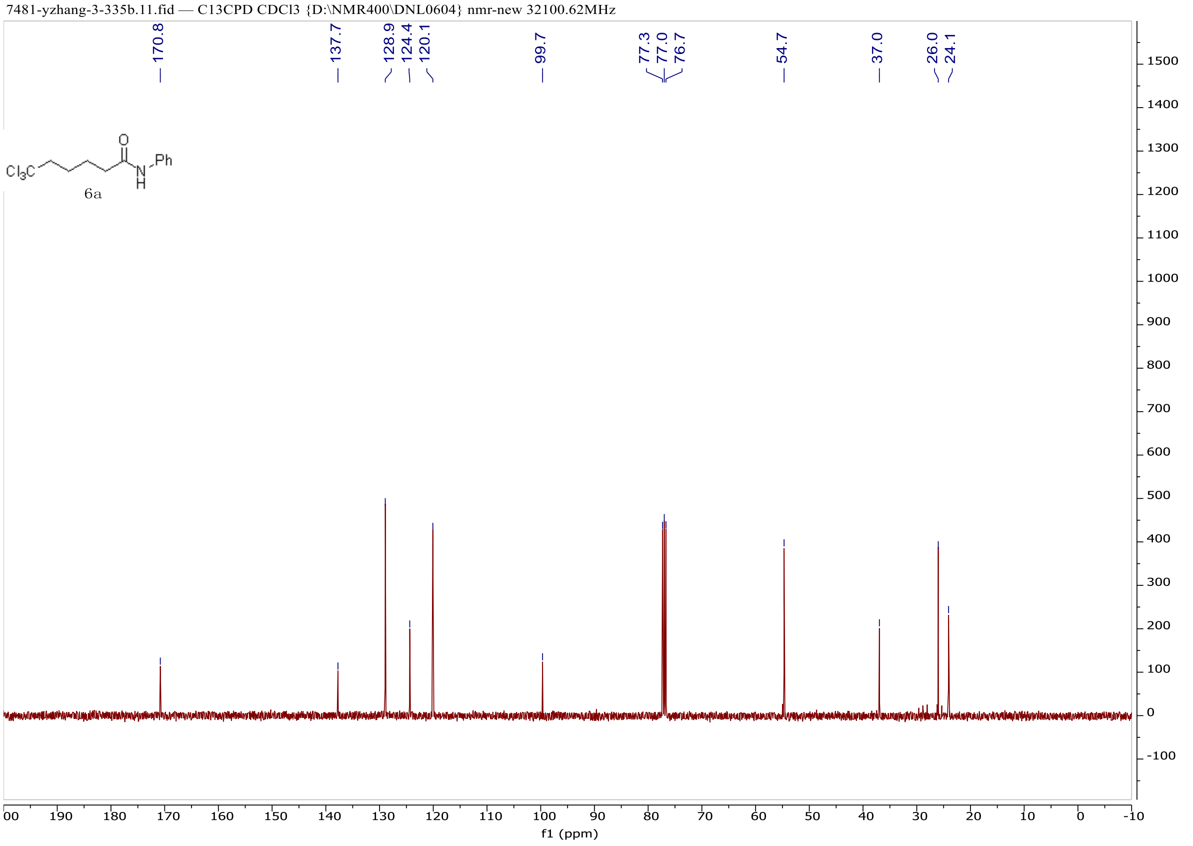
7481-yzhang-3-335b.11.fid — C13CPD CDCl3 {D:\NMR400\DNL0604} nmr-new 32100.62MHz
170.8
137.7
128.9 124.4 120.1
99.7
77.3 77.0 76.7
54.7
37.0
26.0 24.1
Cl3C
Ph
N
H
O
6a
f1 (ppm)

6b

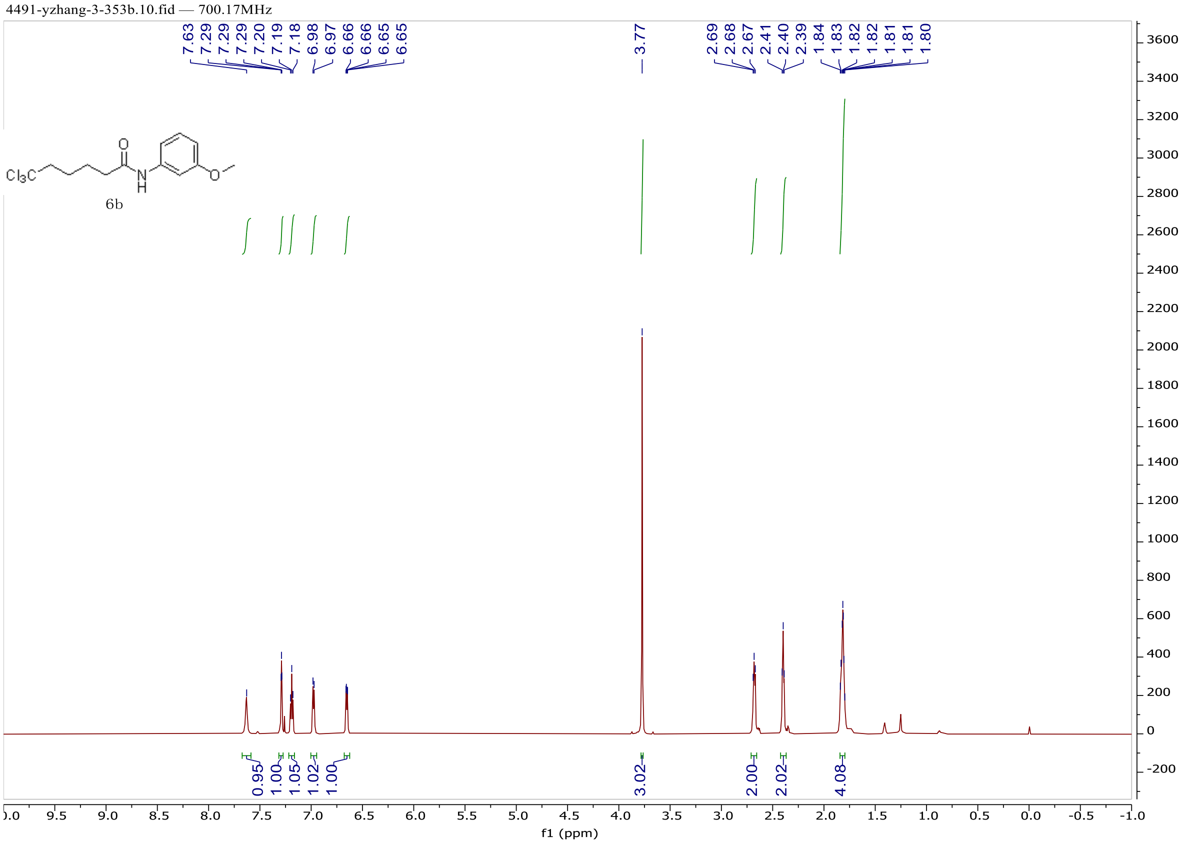
4491-yzhang-3-353b.10.fid — 700.17MHz
7.63 7.29 7.29 7.29 7.20 7.19 7.18 6.98 6.97 6.66 6.66 6.65 6.65
3.77
2.69 2.68 2.67 2.41 2.40 2.39 1.84 1.83 1.82 1.81 1.81 1.80
Cl3C
O
N
H
O
6b
0.95 1.00 1.05 1.02 1.00
3.02
2.00 2.02 4.08
f1 (ppm)

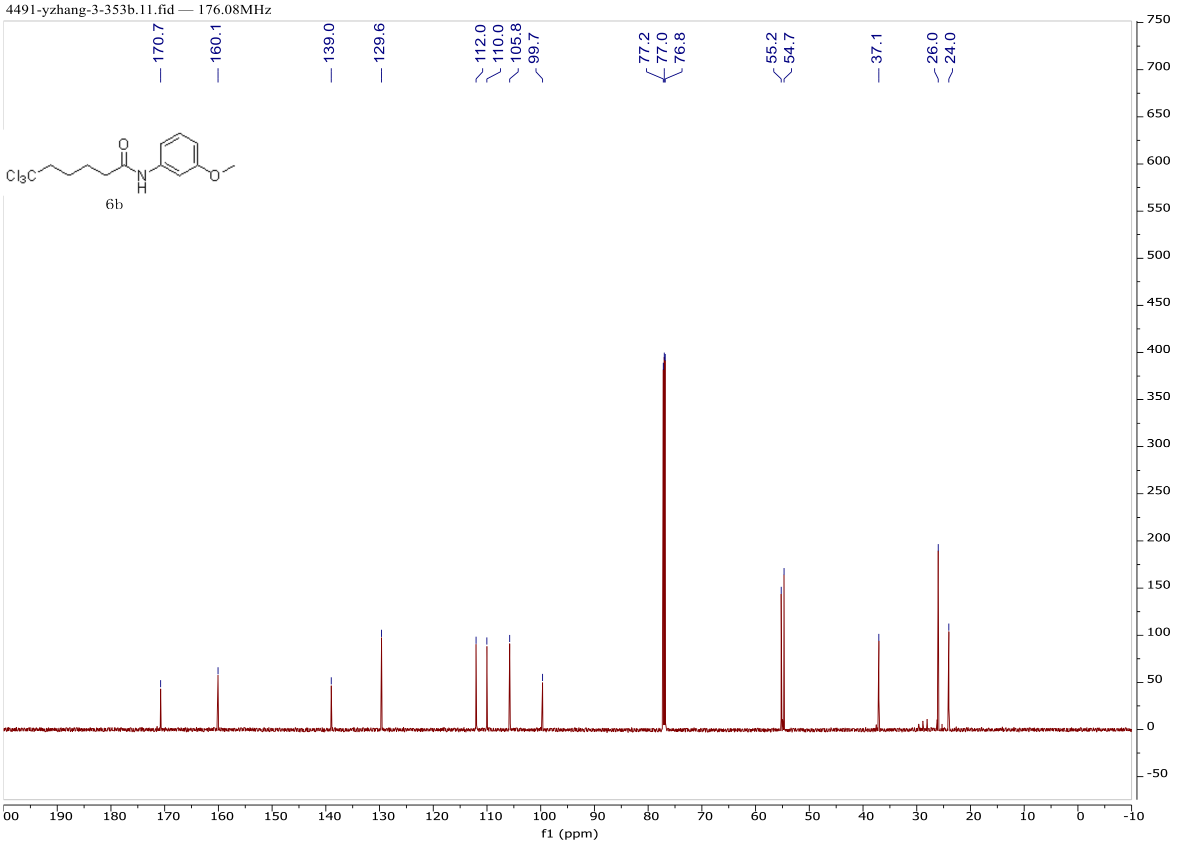
4491-yzhang-3-353b.11.fid — 176.08MHz
170.7
160.1
139.0
129.6
112.0 110.0 105.8 99.7
77.2 77.0 76.8
55.2 54.7
37.1
26.0 24.0
Cl3C
O
N
H
O
6b
f1 (ppm)

6c

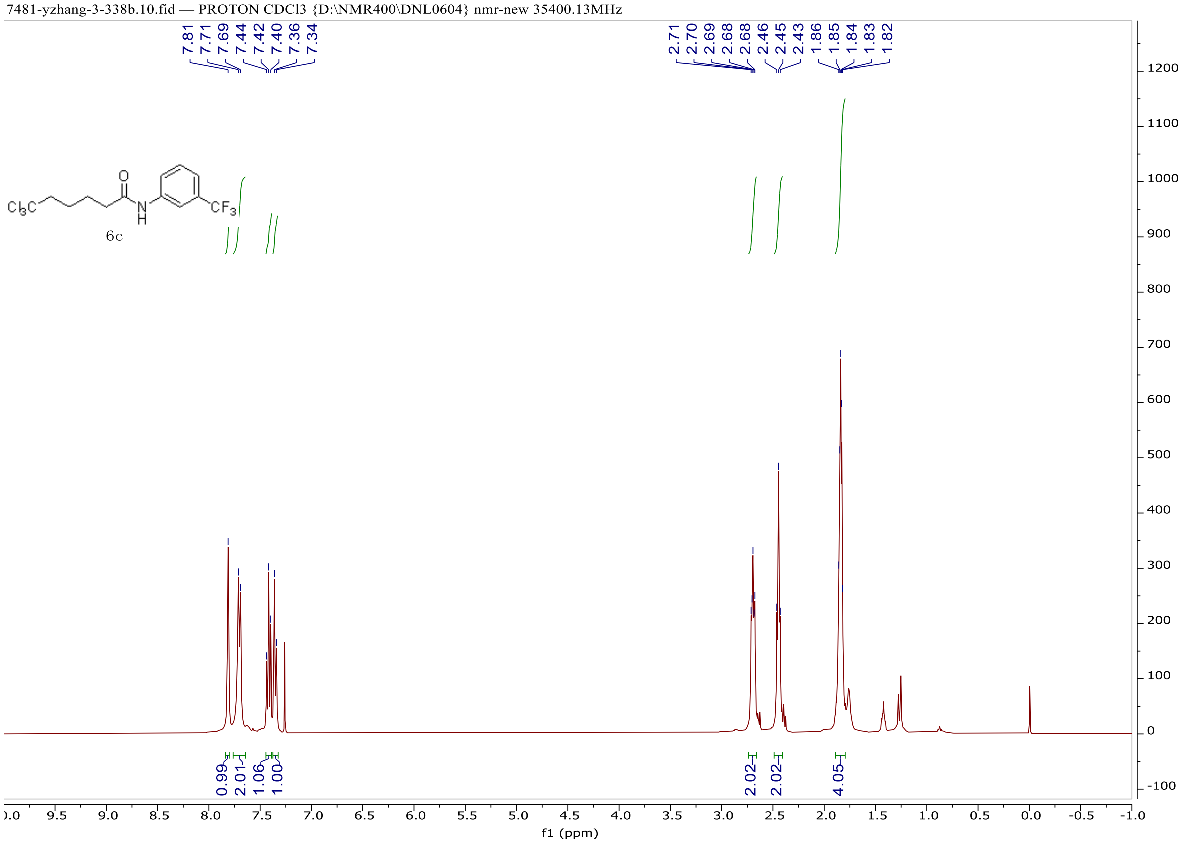

7481-yzhang-3-338b.10.fid — PROTON CDCl3 {D:\NMR400\DNL0604} nmr-new 35400.13MHz
7.81
7.71
7.69
7.44
7.42
7.40
7.36
7.34
2.71
2.70
2.69
2.68
2.68
2.46
2.45
2.43
1.86
1.85
1.84
1.83
1.82
6c
0.99
2.01
1.06
1.00
2.02
2.02
4.05
f1 (ppm)

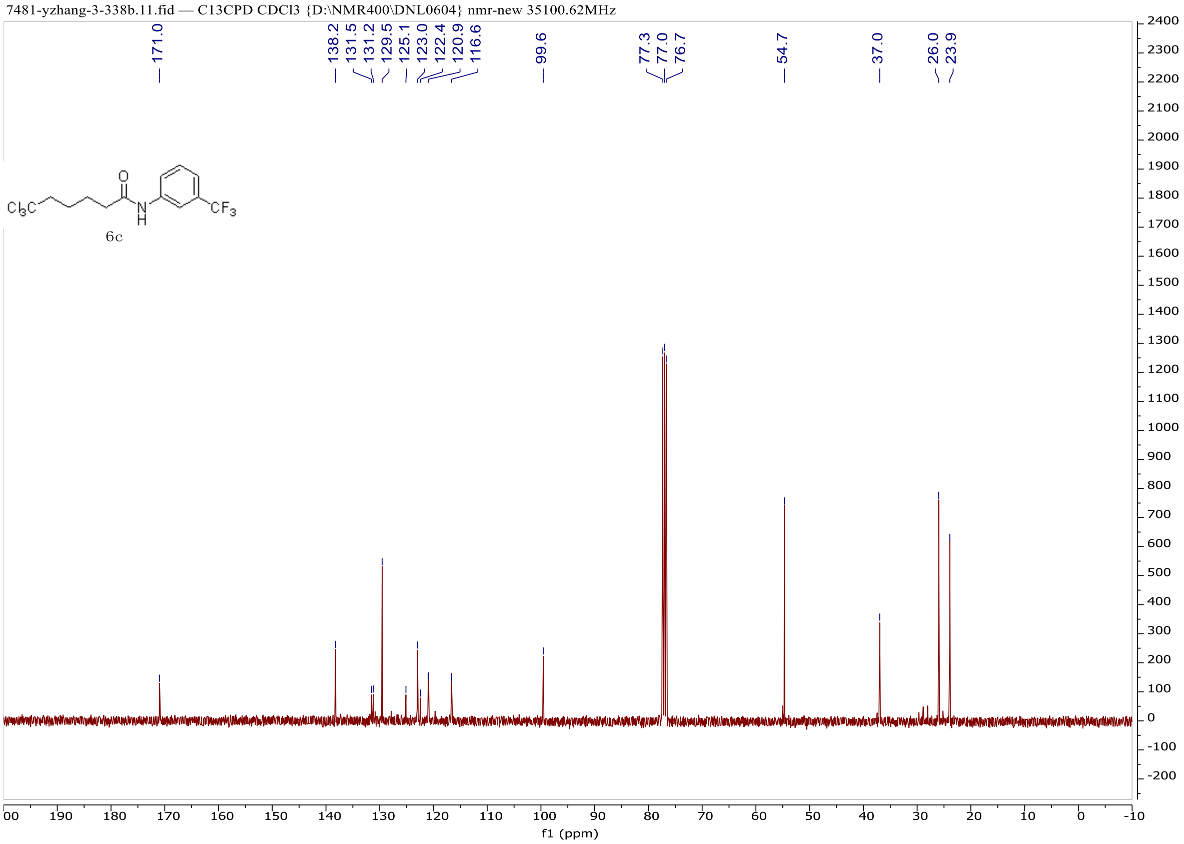

7481-yzhang-3-338b.11.fid — C13CPD CDCl3 {D:\NMR400\DNL0604} nmr-new 35100.62MHz
171.0
138.2
131.5
131.2
129.5
125.1
123.0
122.4
120.9
116.6
99.6
77.3
77.0
76.7
54.7
37.0
26.0
23.9
6c
f1 (ppm)

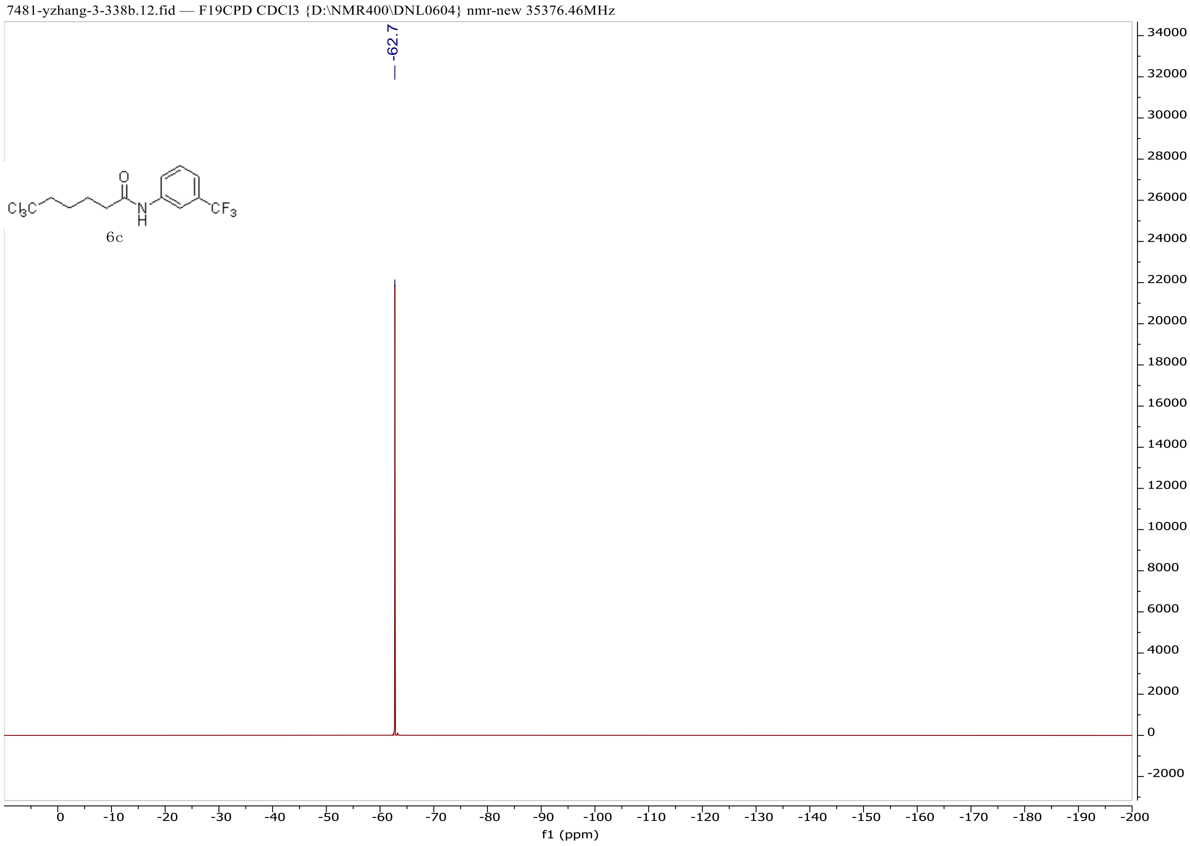
7481-yzhang-3-338b.12.fid — F19CPD CDCl3 {D:\NMR400\DNL0604} nmr-new 35376.46MHz
-62.7
Cl3C
O
N
H
CF3
6c
f1 (ppm)

6d

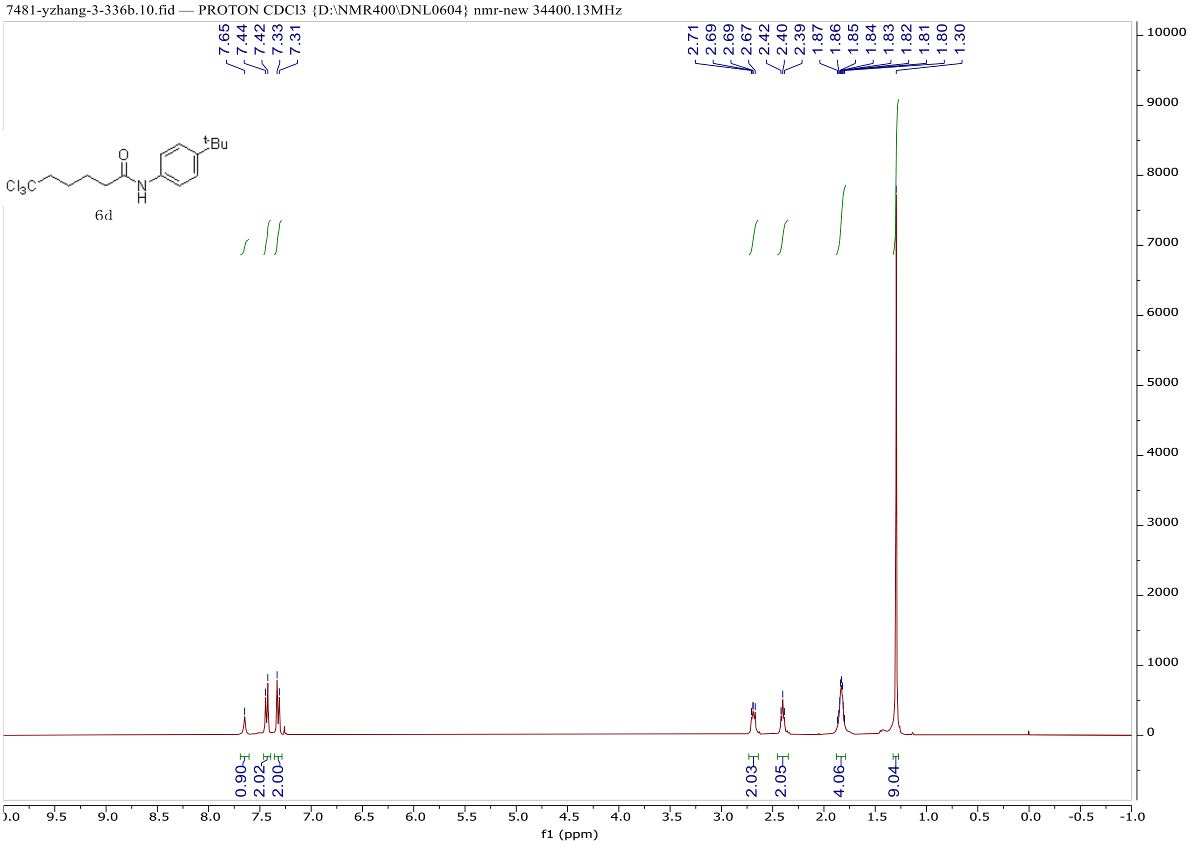
7481-yzhang-3-336b.10.fid — PROTON CDCl3 {D:\NMR400\DNL0604} nmr-new 34400.13MHz
7.65 7.44 7.42 7.33 7.31
2.71 2.69 2.69 2.67 2.42 2.40 2.39 1.87 1.86 1.85 1.84 1.83 1.82 1.81 1.80 1.30
Cl3C
O
N
H
t-Bu
6d
0.90 2.02 2.00
2.03 2.05
4.06
9.04
f1 (ppm)

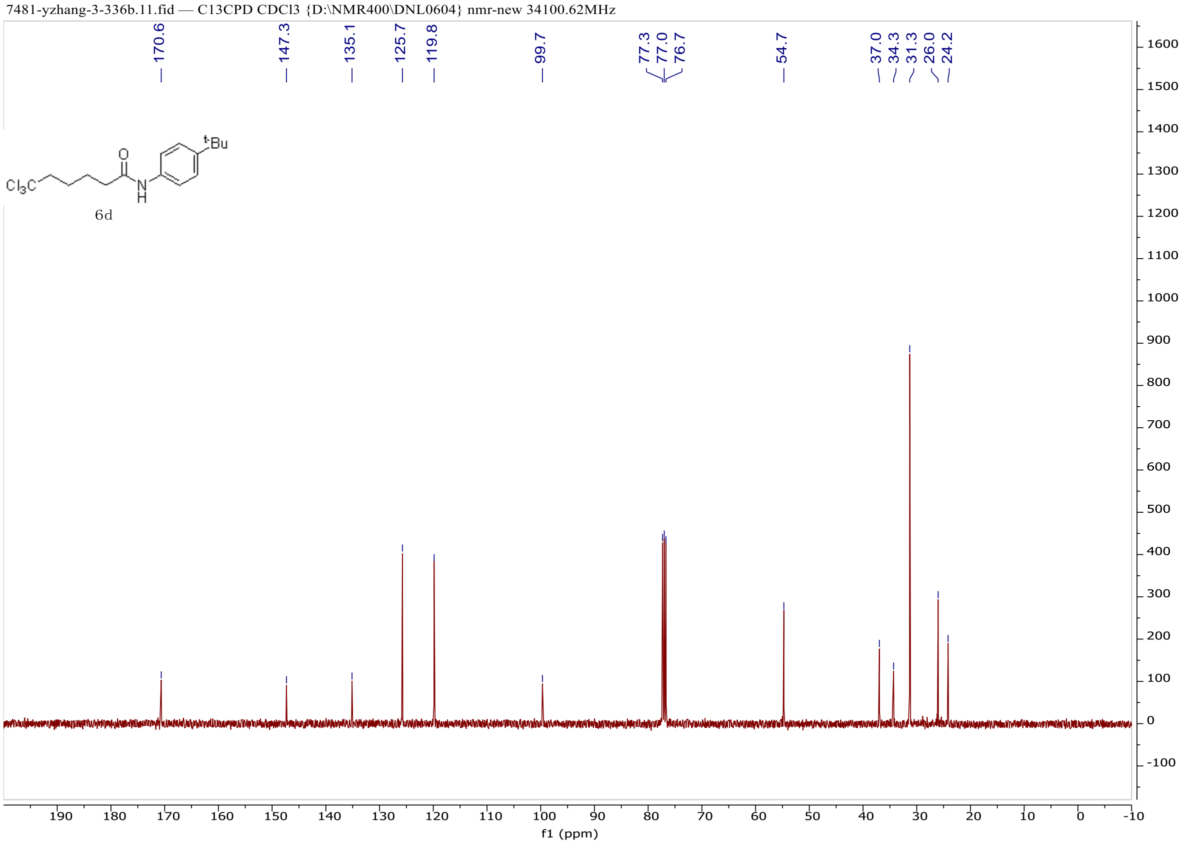
7481-yzhang-3-336b.11.fid — C13CPD CDCl3 {D:\NMR400\DNL0604} nmr-new 34100.62MHz
170.6
147.3
135.1
125.7
119.8
99.7
77.3 77.0 76.7
54.7
37.0 34.3 31.3 26.0 24.2
Cl3C
O
N
H
t-Bu
6d
f1 (ppm)

6e

4491-yzhang-3-352b.10.fid — 700.17MHz

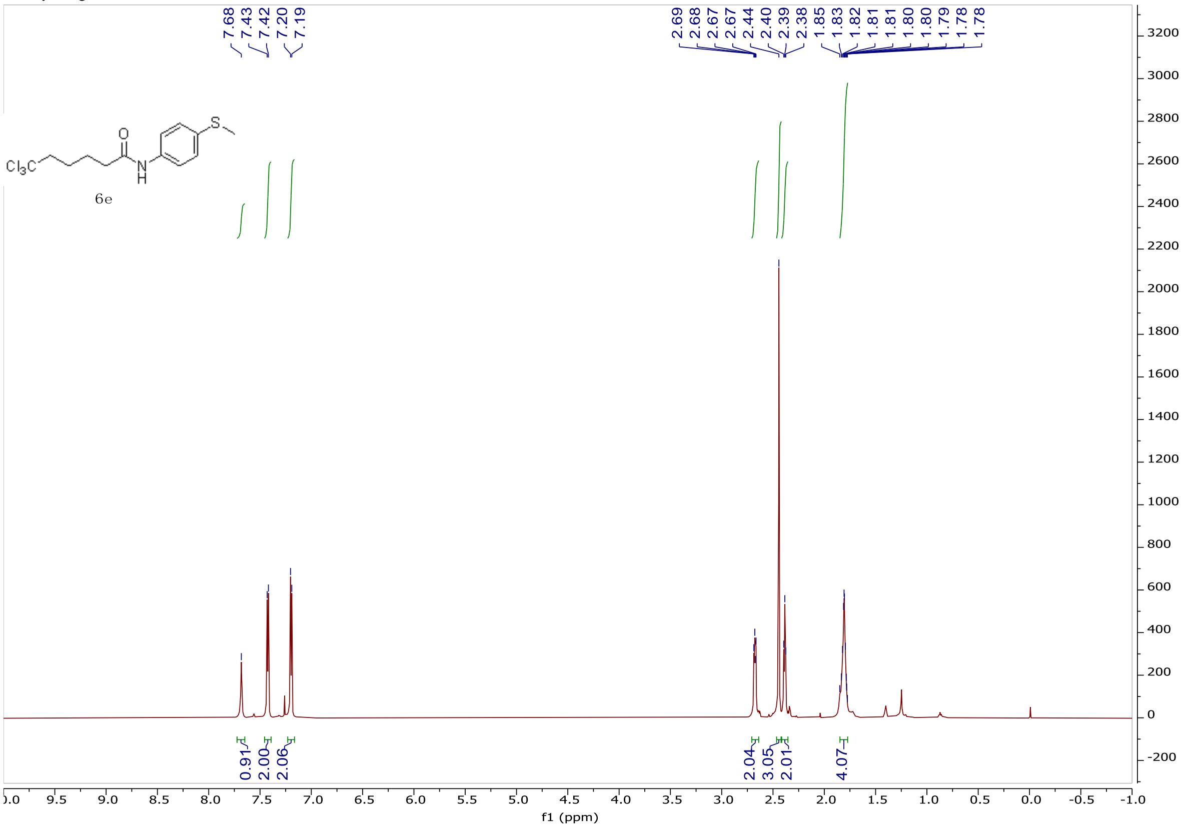

4491-yzhang-3-352b.11.fid — 176.08MHz

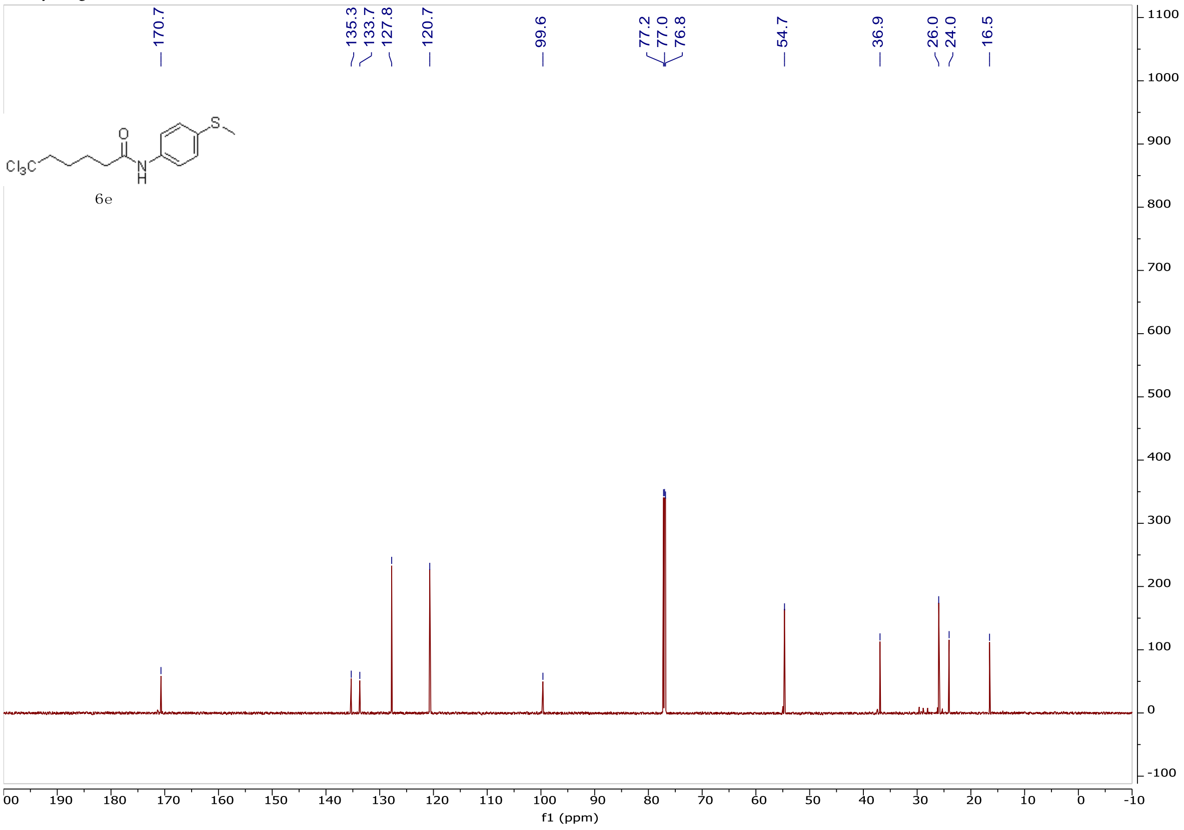

6f

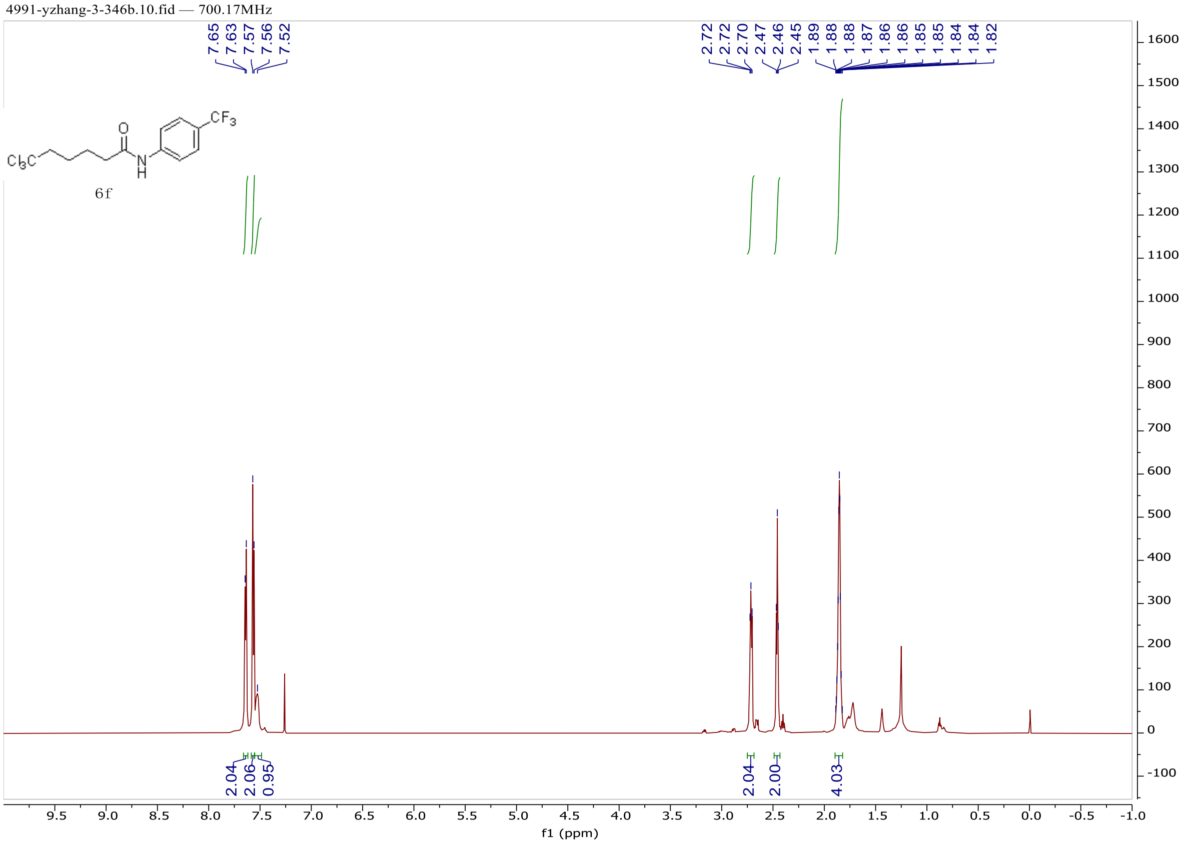
4991-yzhang-3-346b.10.fid — 700.17MHz
7.65
7.63
7.57
7.56
7.52
2.72
2.72
2.70
2.47
2.46
2.45
1.89
1.88
1.88
1.87
1.86
1.86
1.85
1.85
1.84
1.84
1.82
6f
2.04
2.06
0.95
2.04
2.00
4.03
f1 (ppm)

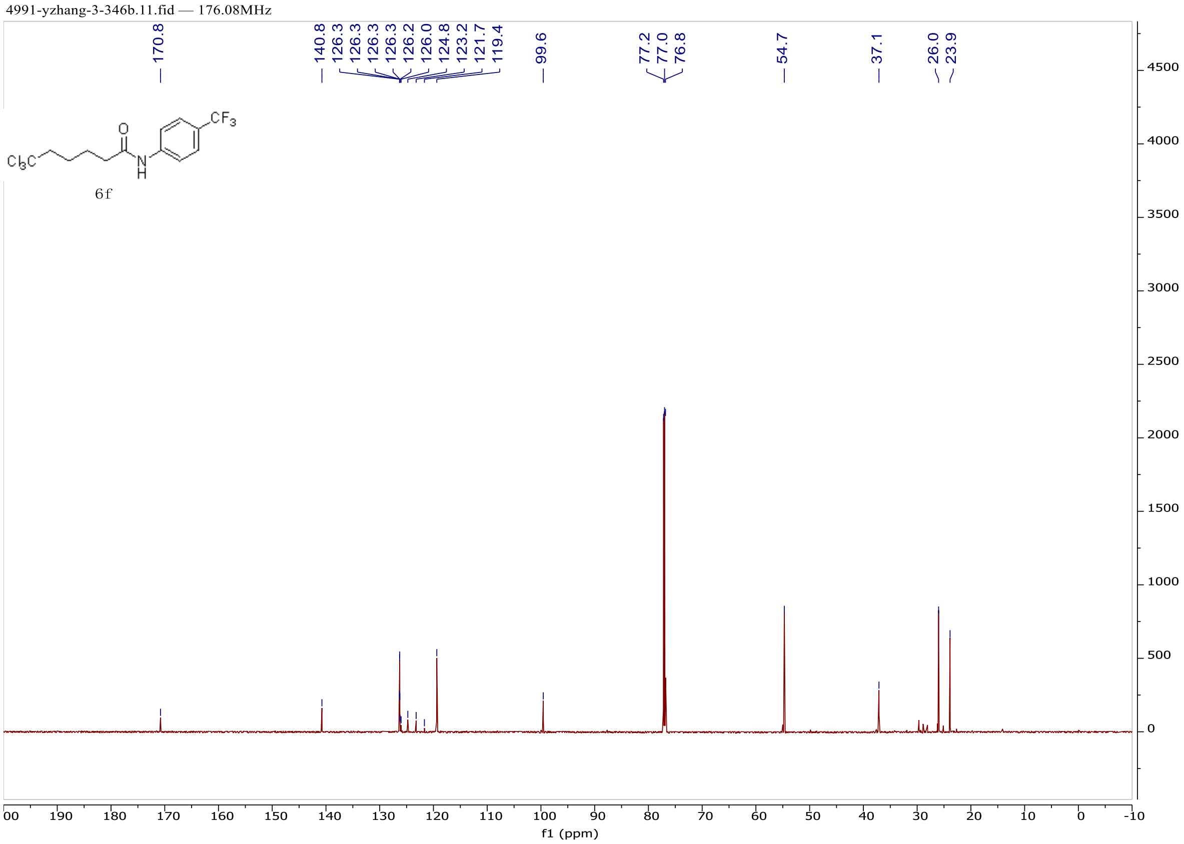
4991-yzhang-3-346b.11.fid — 176.08MHz
170.8
140.8
126.3
126.3
126.3
126.2
126.0
124.8
123.2
121.7
119.4
99.6
77.2
77.0
76.8
54.7
37.1
26.0
23.9
6f
f1 (ppm)

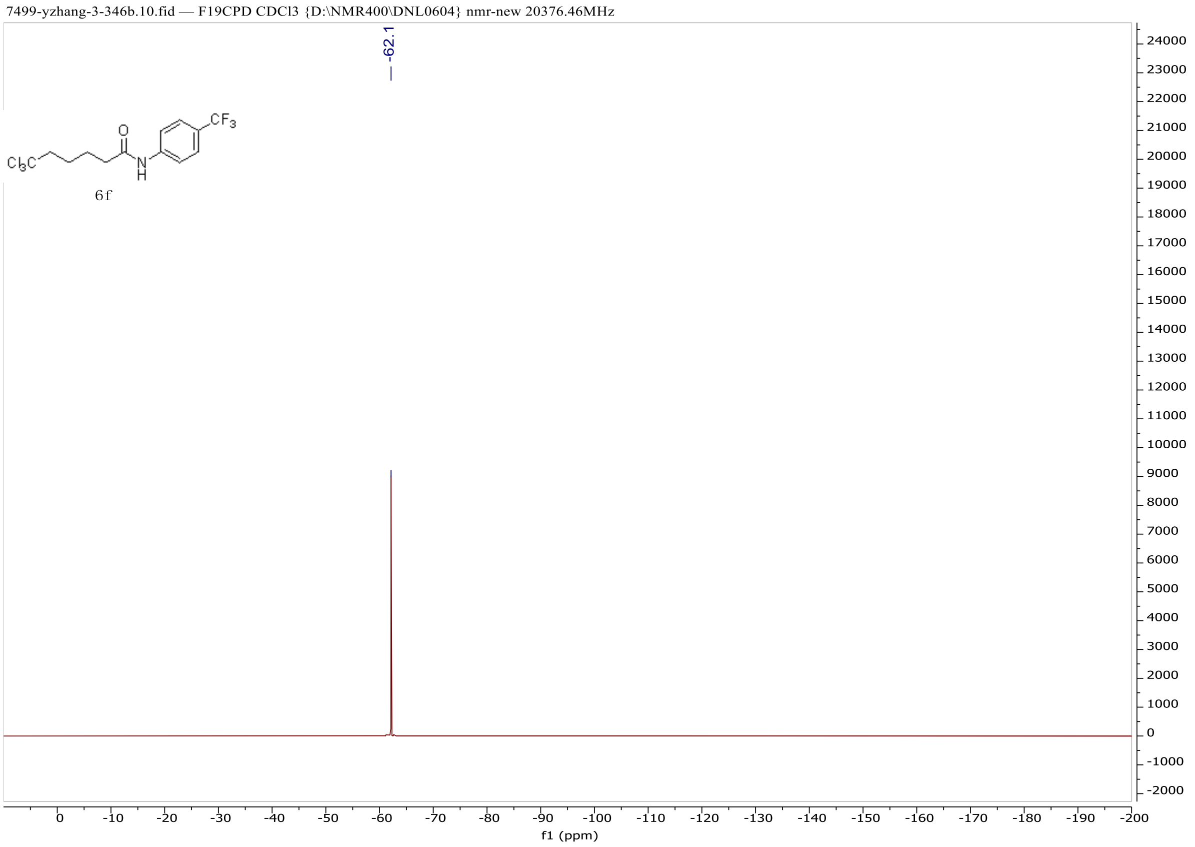
7499-yzhang-3-346b.10.fid — F19CPD CDCl3 {D:\NMR400\DNL0604} nmr-new 20376.46MHz
-62.1
CF3
O
Cl3C
N
H
6f
f1 (ppm)

6g

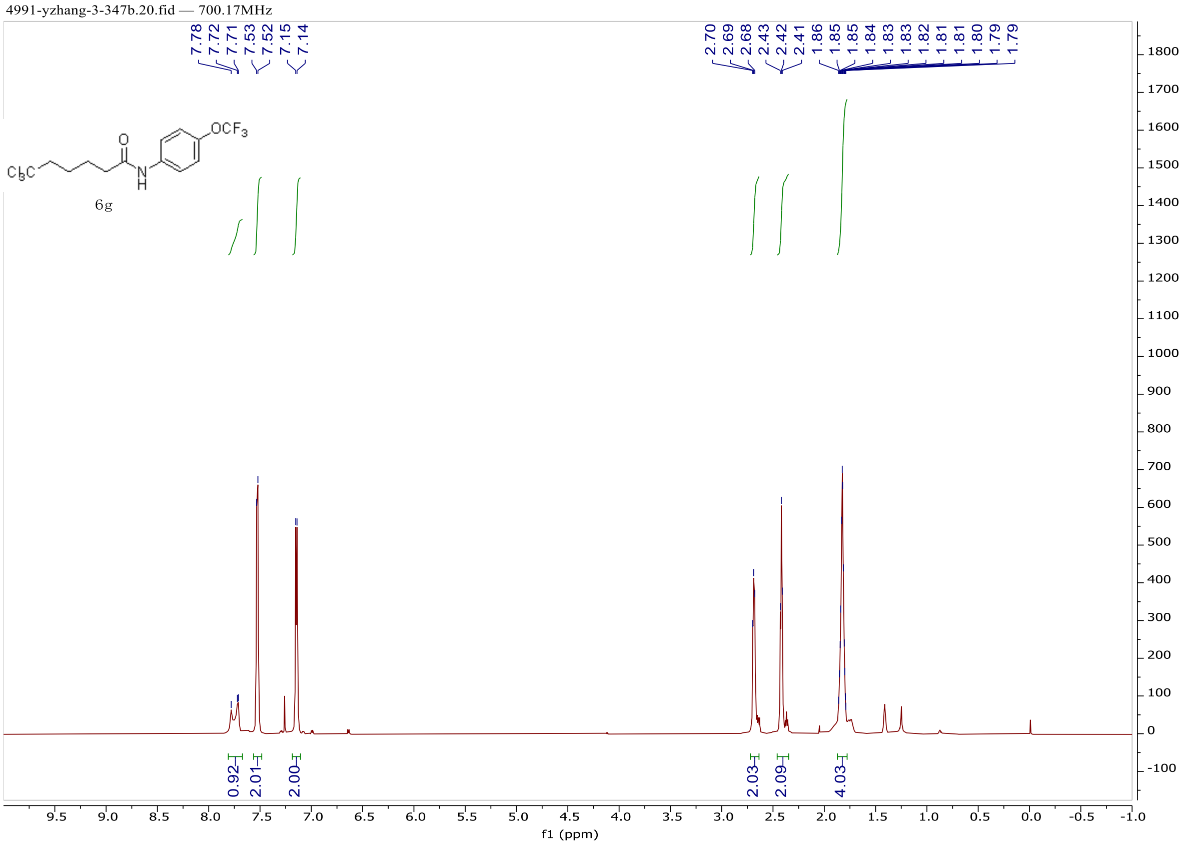
4991-yzhang-3-347b.20.fid — 700.17MHz
7.78 7.72 7.71 7.53 7.52 7.15 7.14
2.70 2.69 2.68 2.43 2.42 2.41 1.86 1.85 1.85 1.84 1.83 1.83 1.82 1.81 1.81 1.80 1.79 1.79
OCF3
Cl3C
O
N
H
6g
0.92 2.01 2.00 2.03 2.09 4.03
f1 (ppm)

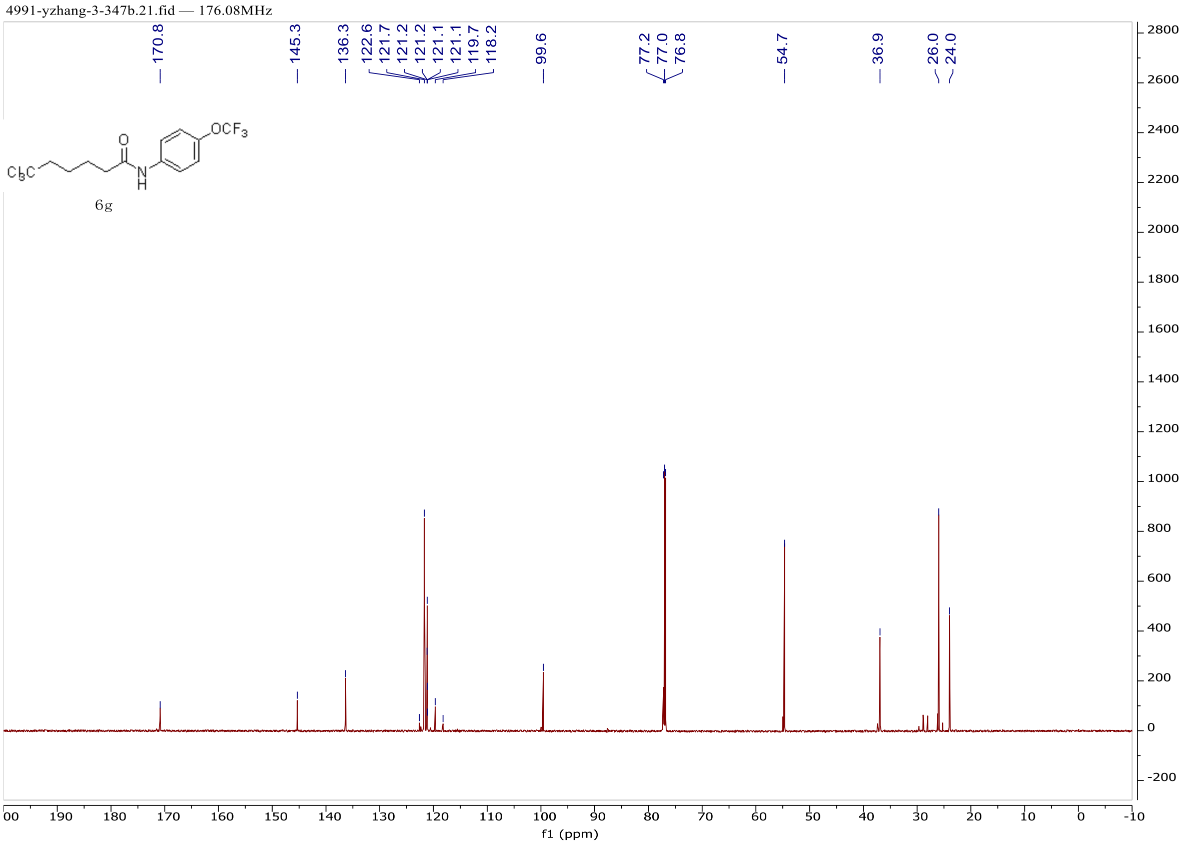
4991-yzhang-3-347b.21.fid — 176.08MHz
170.8
145.3
136.3
122.6 121.7 121.2 121.2 121.1 121.1 119.7 118.2
99.6
77.2 77.0 76.8
54.7
36.9
26.0 24.0
OCF3
Cl3C
O
N
H
6g
f1 (ppm)

7499-yzhang-3-347b.10.fid — F19CPD CDCl3 {D:\NMR400\DNL0604} nmr-new 21376.46MHz

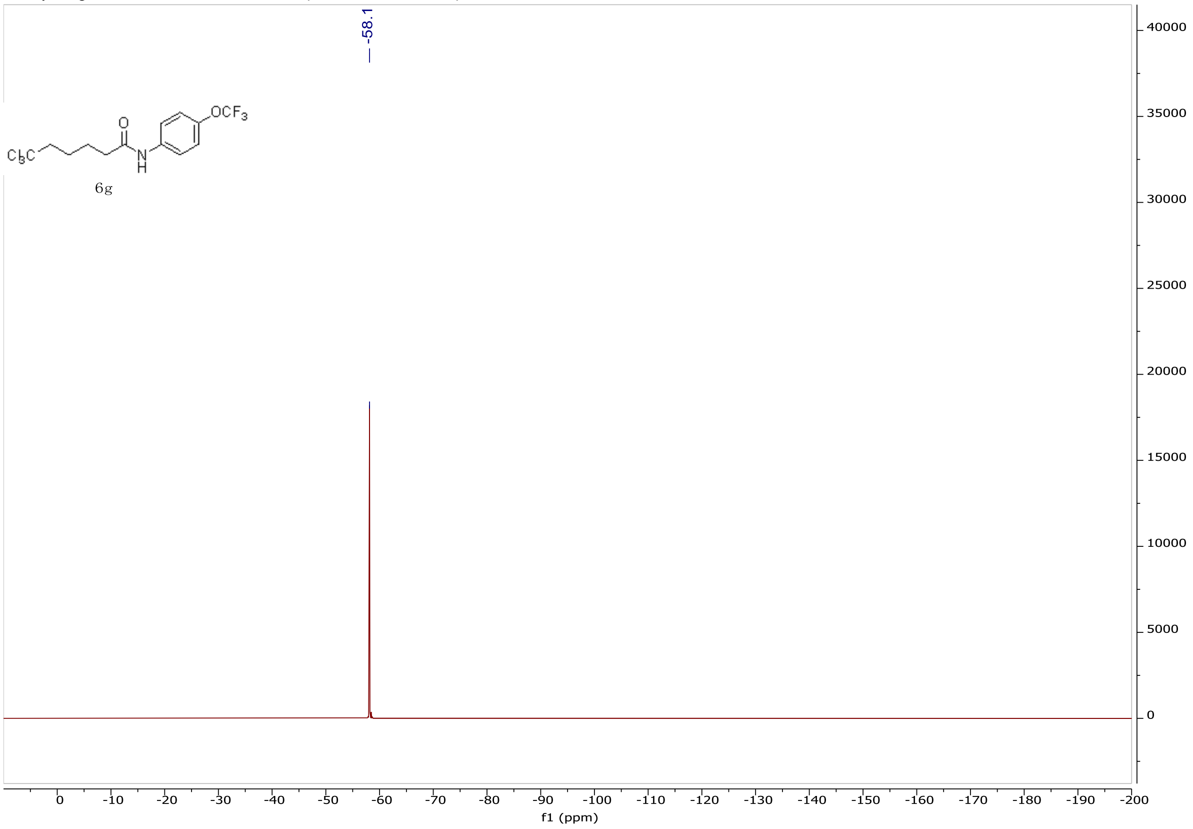

6h

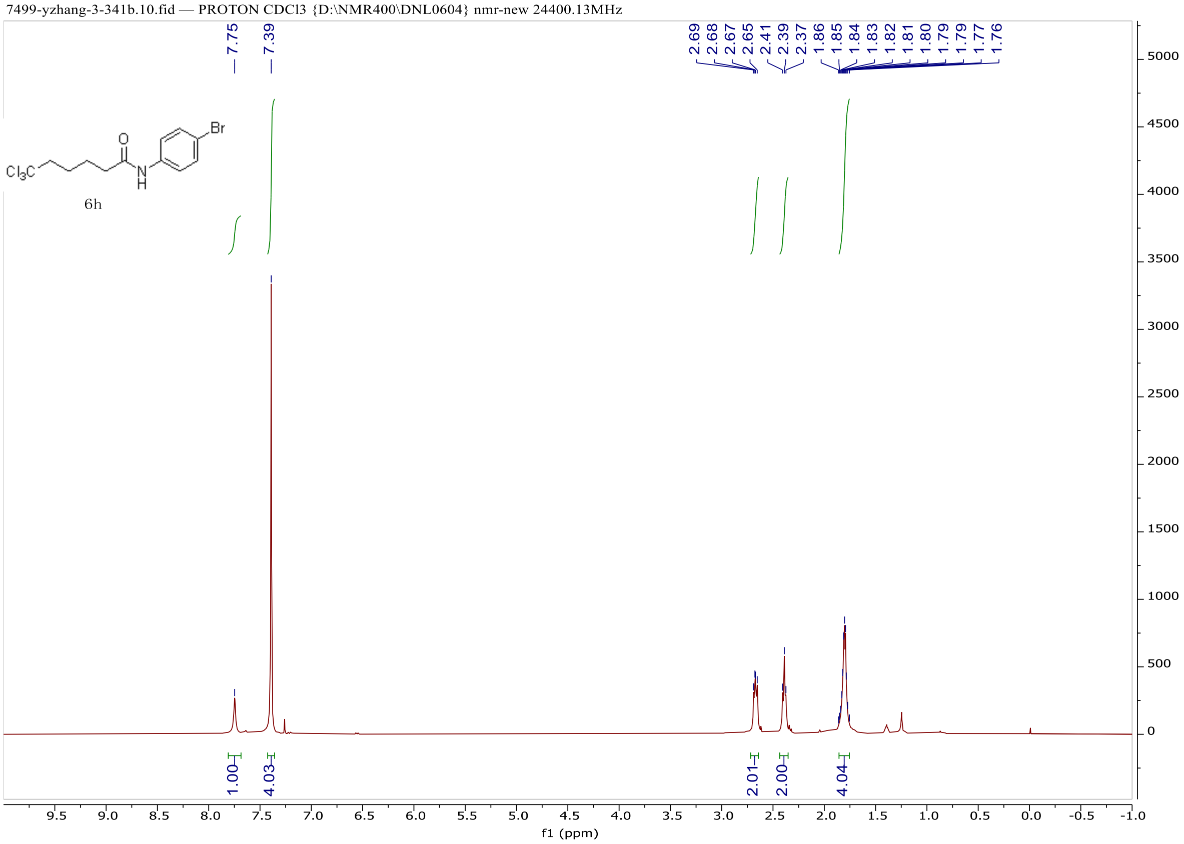
7499-yzhang-3-341b.10.fid — PROTON CDCl3 {D:\NMR400\DNL0604} nmr-new 24400.13MHz
6h
f1 (ppm)

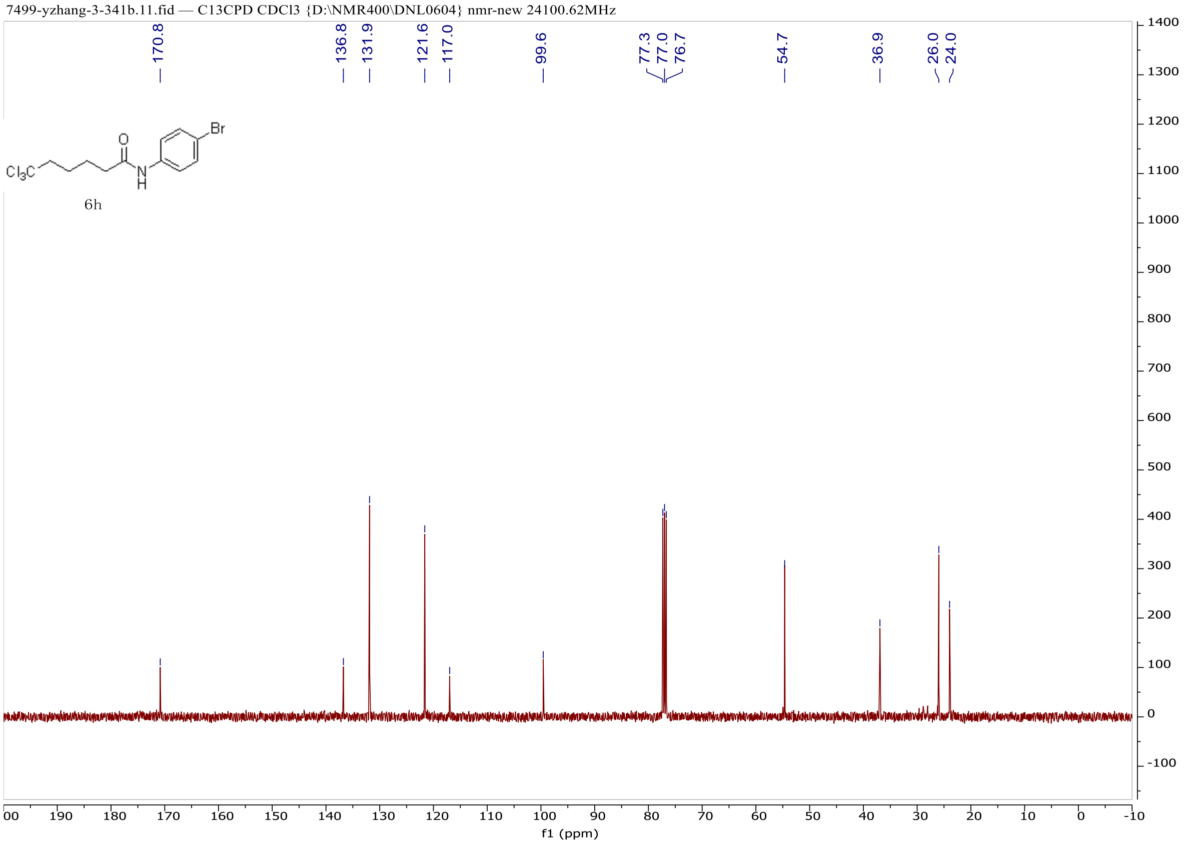
7499-yzhang-3-341b.11.fid — C13CPD CDCl3 {D:\NMR400\DNL0604} nmr-new 24100.62MHz
170.8
136.8
131.9
121.6
117.0
99.6
77.3
77.0
76.7
54.7
36.9
26.0
24.0
6h
f1 (ppm)

6i

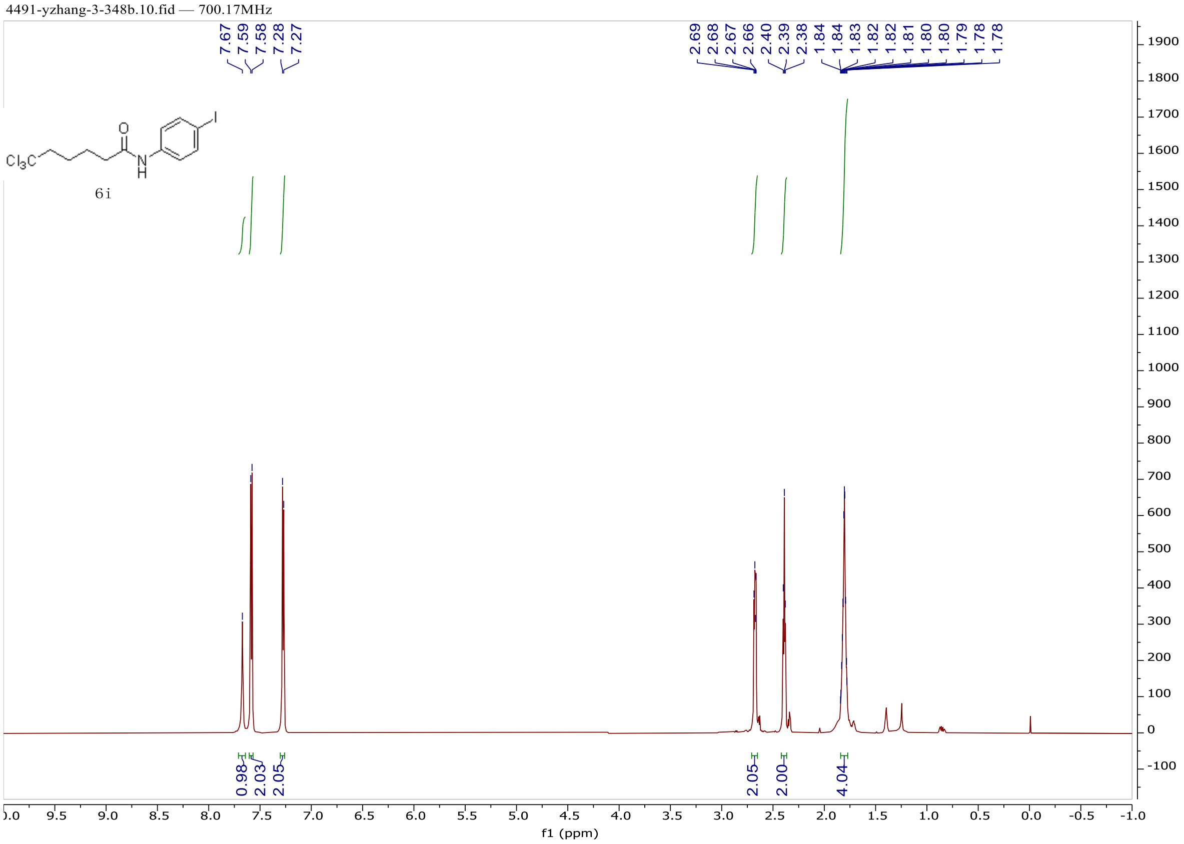
4491-yzhang-3-348b.10.fid — 700.17MHz
7.67 7.59 7.58 7.28 7.27
2.69 2.68 2.67 2.66 2.40 2.39 2.38 1.84 1.84 1.83 1.82 1.81 1.80 1.80 1.79 1.78 1.78
O
N
H
I
Cl3C
6i
0.98 2.03 2.05
2.05 2.00 4.04
f1 (ppm)

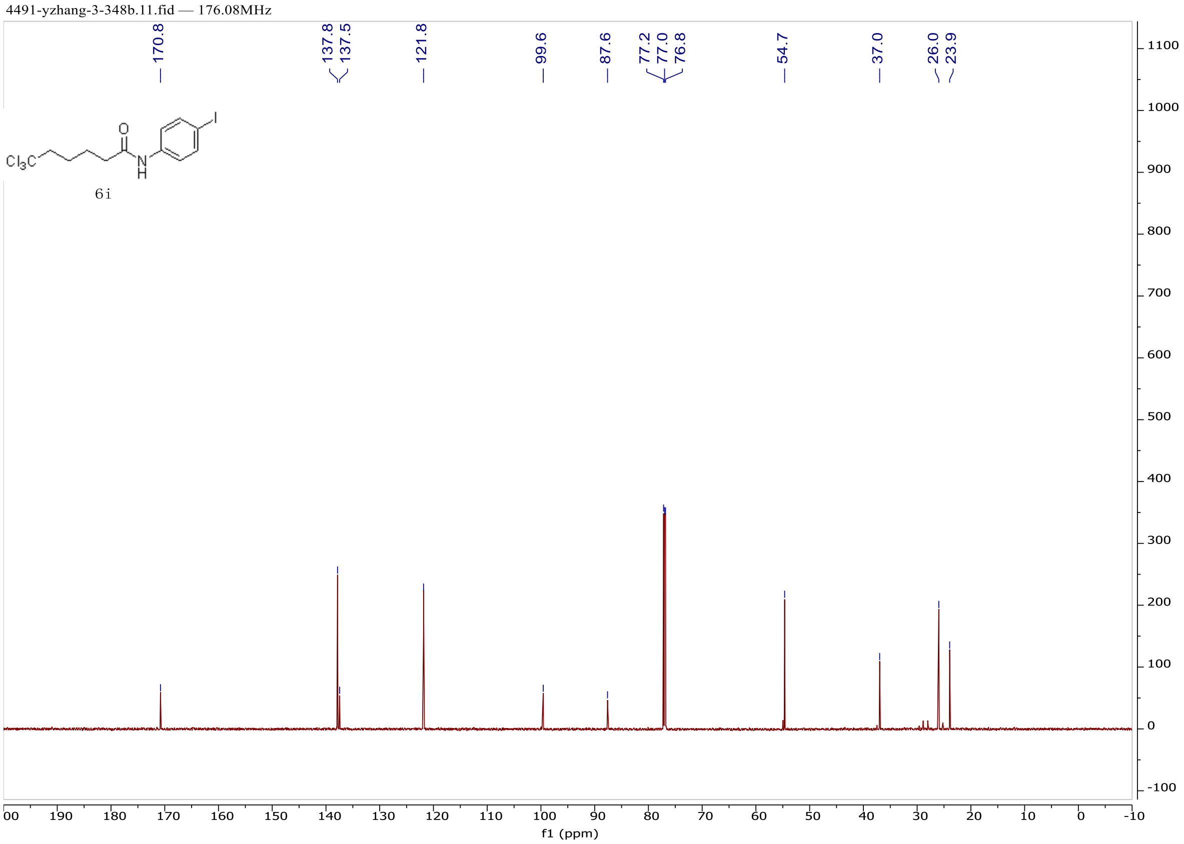
4491-yzhang-3-348b.11.fid — 176.08MHz
170.8
137.8 137.5
121.8
99.6
87.6
77.2 77.0 76.8
54.7
37.0
26.0 23.9
O
N
H
I
Cl3C
6i
f1 (ppm)
